# A Physiologically-Based Pharmacokinetic (PBPK) Model Network for the Prediction of CYP1A2 and CYP2C19 Drug–Drug–Gene Interactions with Fluvoxamine, Omeprazole, S-mephenytoin, Moclobemide, Tizanidine, Mexiletine, Ethinylestradiol, and Caffeine

**DOI:** 10.3390/pharmaceutics12121191

**Published:** 2020-12-08

**Authors:** Tobias Kanacher, Andreas Lindauer, Enrica Mezzalana, Ingrid Michon, Celine Veau, Jose David Gómez Mantilla, Valerie Nock, Angèle Fleury

**Affiliations:** 1 SGS-Exprimo, 2800 Mechelen, Belgium; tobias.kanacher@pharmetheus.com (T.K.); andreas.lindauer@gmail.com (A.L.); enrica.mezzalana@pharmetheus.com (E.M.); Ingrid.Michon@certara.com (I.M.); 2 Boehringer Ingelheim Pharma GmbH & Co. KG, Birkendorfer Str. 65, 88397 Biberach an der Riß, Germany; celine.veau@boehringer-ingelheim.com (C.V.); jose_david.gomez_mantilla@boehringer-ingelheim.com (J.D.G.M.); Valerie.nock@boehringer-ingelheim.com (V.N.)

**Keywords:** physiologically-based pharmacokinetic (PBPK) modeling, drug–drug interactions, drug–gene interactions

## Abstract

Physiologically-based pharmacokinetic (PBPK) modeling is a well-recognized method for quantitatively predicting the effect of intrinsic/extrinsic factors on drug exposure. However, there are only few verified, freely accessible, modifiable, and comprehensive drug–drug interaction (DDI) PBPK models. We developed a qualified whole-body PBPK DDI network for cytochrome P450 (CYP) CYP2C19 and CYP1A2 interactions. Template PBPK models were developed for interactions between fluvoxamine, S-mephenytoin, moclobemide, omeprazole, mexiletine, tizanidine, and ethinylestradiol as the perpetrators or victims. Predicted concentration–time profiles accurately described a validation dataset, including data from patients with genetic polymorphisms, demonstrating that the models characterized the CYP2C19 and CYP1A2 network over the whole range of DDI studies investigated. The models are provided on GitHub (GitHub Inc., San Francisco, CA, USA), expanding the library of publicly available qualified whole-body PBPK models for DDI predictions, and they are thereby available to support potential recommendations for dose adaptations, support labeling, inform the design of clinical DDI trials, and potentially waive those.

## 1. Introduction

Cytochrome P450 (CYP) proteins CYP1A2 and CYP2C19 are important enzymes involved in the metabolism of about 15% and 10% of therapeutic drugs, respectively [1,2,3], the inhibition or induction of which can result in clinically relevant drug–drug interactions (DDIs). Furthermore, genetic polymorphisms for functional CYP2C19 expression may result in drug–gene interactions (DGIs), which can significantly impact drug plasma exposure; approximately 3–5% of European and 15–20% of Asian populations are poor metabolizers (PMs) with no CYP2C19 activity [1,4]. In current clinical practice, DDIs and DGIs are often considered separate entities. However, interactions between them can cause synergistic effects, and thus ignoring drug–drug–gene interactions (DDGIs) can jeopardize patient safety. 

During drug development, the investigation of DDGIs in clinical trials can be challenging or impossible for reasons including ethical and feasibility restrictions due to the complexity of DDGIs and practical restrictions based on the frequency of polymorphisms in the study population. Physiologically-based pharmacokinetic (PBPK) modeling is a well-recognized method to quantitatively predict the effect of patients’ extrinsic and intrinsic factors on drug exposure [5,6,7]. Thus, PBPK modeling is recognized by regulatory agencies, the pharmaceutical industry, and academia as a useful tool to investigate DDGIs. Current guidelines from the European Medicines Agency [8] and the U.S. Food and Drug Administration (FDA) [9] recommend the use of carefully qualified PBPK models to dynamically evaluate DDIs that are involved in metabolic and transport processes. Based on an evaluation of the predictive performance of the drug model, these can subsequently be used to support alternative dosing regimens, clinical study design, or labeling. Guidelines require the PBPK platform to be qualified for its intended use, e.g., the prediction of a DDI for a CYP1A2 substrate [8]. For this purpose, a prespecified qualification dataset using reference substrates and inhibitors is needed to evaluate the PBPK platform before DDI prediction and verification in the specified scenario. Even though many perpetrator and victim drug models are available to explain certain study designs and conditions, further models and more comprehensive DDI networks suitable for general use are needed. 

The aim of this study was to develop a qualified PBPK DDI network for CYP2C19 and CYP1A2 interactions, thereby expanding the library of publicly available models for DDI predictions with qualified whole-body PBPK models, by building template perpetrator/victim PBPK models for fluvoxamine, S-mephenytoin, moclobemide, omeprazole, mexiletine, tizanidine, and ethinylestradiol. Then, the PBPK models were validated by describing or predicting published clinical data and previously undisclosed clinical data. Additionally, we describe and predict DGIs between victim and perpetrator drugs for patients with genetic polymorphisms affecting CYP2C19 metabolism.

## 2. Methods

### 2.1. Data Sources and Software

**Figure 1:**
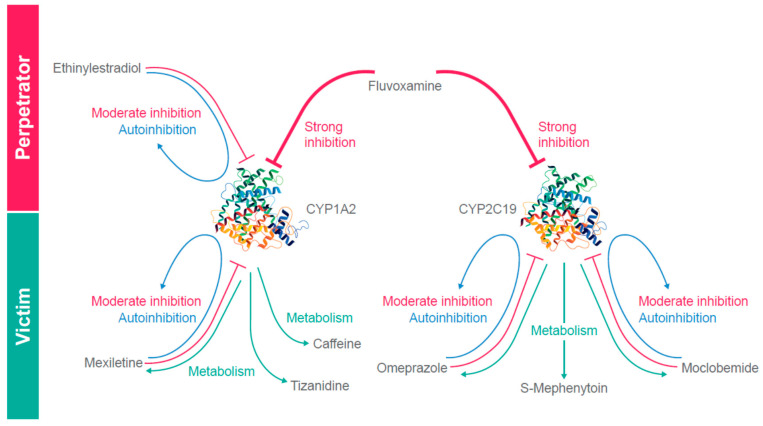
Graphical representation of the CYP2C19/CYP1A2 DDI network.

The published literature was searched to select CYP1A2 and CYP2C19 substrates and inhibitors for the DDI network and to identify and evaluate the available concentration–time data needed for model development and qualification. Recommendations for reference index substrates and inhibitors by the FDA [10] were also considered to ensure the inclusion of a range of strong and moderate CYP inhibition. Each substrate or inhibitor was validated by at least two independent clinical drug inhibition combination studies if data were available. A summary of the CYP2C19/CYP1A2 DDI network is shown in Figure 1. Qualification of the network was accomplished by prediction of the observed clinical interactions for the pairs listed in Table 1.

Concentration–time data from relevant publications were digitized using DigitizeIt version 2.0.5 (Digitizeit, Braunschweig, Germany) (www.digitizeit.de), supplemented with Boehringer Ingelheim study data, and collated by compound, dose, formulation, route of administration, and key patient characteristics. Additionally, studies sponsored by Boehringer Ingelheim were added to those data (see Supplementary Material S1).

PBPK analyses were performed using the open source software Open Systems Pharmacology (OSP) suite version 7.2.2 or higher (http://www.open-systems-pharmacology.org), and a re-qualification work was conducted in version 9.1 in order to upload the models as templates on OSP. In vitro/in vivo extrapolation (using data sources summarized in Supplementary Material S1) was attempted for relevant processes or, if available, in vivo information was directly derived from literature/unpublished internal sources. A middle-out modeling approach was also performed to estimate parameters with iterations of prediction–optimization cycles. 

### 2.2. Model Development and Evaluation

Several iterations of prediction–optimization cycles were performed, visually comparing predictions to digitized concentration–time curves found in publications or optimizing selected model parameters. Key endpoints used in modeling were the predicted perpetrator/victim drug concentration–time profiles, area under the plasma concentration–time curve (AUC), and maximum plasma concentration (C_max_) for each evaluated DDI pair. In addition to the visual comparison of predicted and observed data, model evaluations for some compounds included the precision of parameter estimates and correlations between them. In general, model development proceeded step-wise (Table 2). A detailed summary of the model development steps for each drug can be found along with further methodological details in Supplementary Material S2a,b.

### 2.3. Sensitivity Analyses

In the OSP suite, the sensitivity of a pharmacokinetic (PK) parameter of a certain output (*PK_j_*) to an input parameter (*p_i_*) is calculated by varying the input parameter around the value in the simulation by a (small) change (Δ*p_i_*) and performing a new simulation for the changed input parameter value (keeping all other input values unchanged). The change of *PK_j_* (Δ*PK_j_*) was calculated as the difference between the values in the new simulation and the original simulation. Sensitivity coefficients (*S_ij_*) were calculated as the ratio of the relative change of that PK Parameter [= (Δ*PK_j_*)/*PK_j_*] and the relative variation of the input parameter [= (Δ*p_i_*)/*p_i_*]:

$$S_{ij}=\left(\frac{\Delta PK_{j}}{\Delta p_{i}}\right)\times \frac{p_{i}}{PK_{j}}.$$

For reasons of numerical stability, sensitivity is calculated as the average of several sensitivities based on different variations Δ*_k_*:

$$S_{i,j}=\frac{\sum_{k=1}^{n}\left(\frac{\Delta_{k}PK_{j}}{\Delta_{k}p_{i}}\right)\times \frac{p_{i}}{PK_{j}}}{n}.$$

Sensitivities were not calculated for input parameters that had an initial value of 0 or for parameters of the model that should not be changed, so as to prevent accidental structural model changes. In this analysis, the standard set of PK-Sim model parameters visible in simple view (including e.g., solubility, metabolizing enzymes, organ volumes, and blood flows and all parameters included in the sensitivity analysis) were investigated, and a list of the input parameters with the most impact on either AUC or C_max_ ranked by their respective sensitivity were generated. Parameters that contributed to 90% of the cumulated sensitivity were further investigated. 

The supplementary documents to this paper are compiled as a transparent and comprehensive documentation and reference manual, providing detailed information on all PBPK models and modeled DDI studies. Model files will be freely available on GitHub. 

## 3. Results

### 3.1. *Fluvoxamine*

A PBPK model for fluvoxamine was developed with two parallel metabolic pathways via CYP2D6 as a saturable and CYP1A2 as a linear process. For model development and qualification, data from 21 clinical studies after intravenous and oral administration using different formulations were used, with seven studies assigned to the training set (see Supplementary Material S1.1). For DDI prediction with fluvoxamine as a perpetrator, the in vivo competitive inhibition constant (*K_i_*) corrected for protein binding was used.

**Figure 2:**
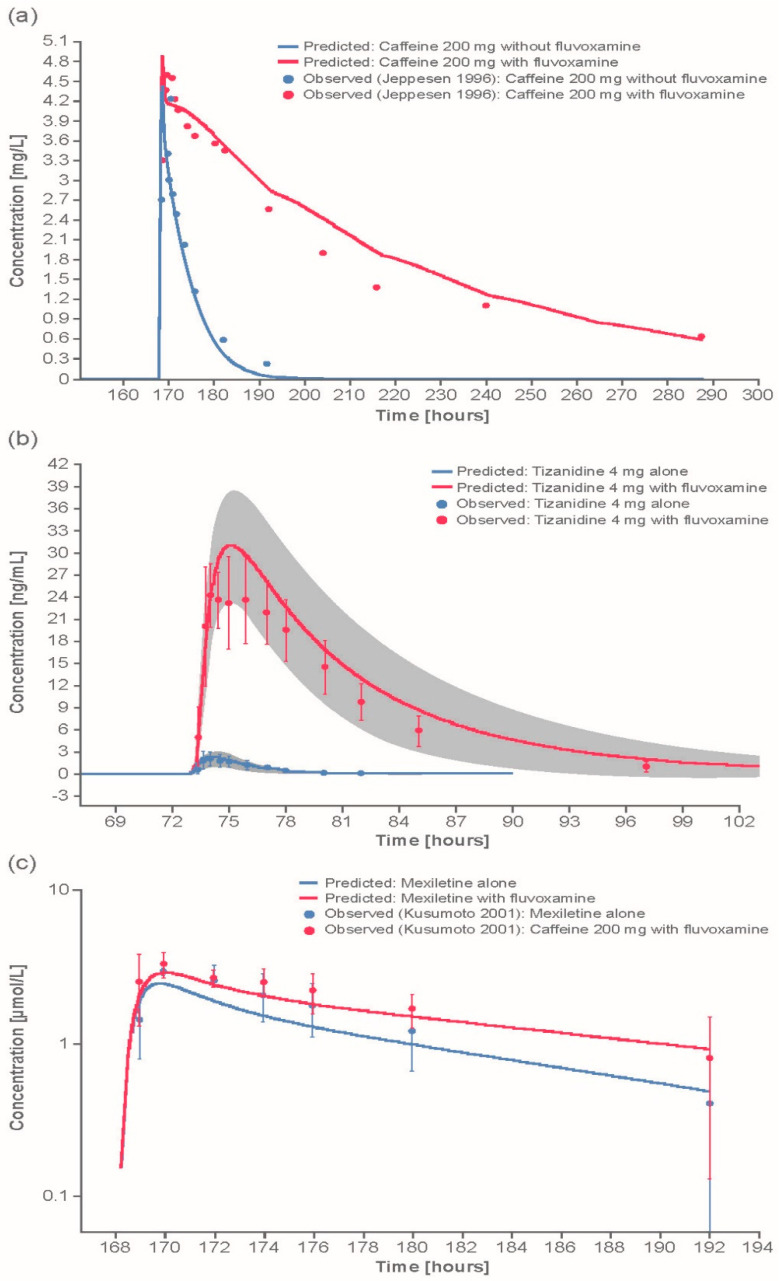
Example comparisons of model-predicted and observed CYP1A2 DDI outcomes: (**a**) Predicted and observed caffeine concentrations, with and without coadministration of fluvoxamine; (**b**) predicted and observed mean (±standard deviation) concentrations of tizanidine after 4 mg dose given with or without fluvoxamine using the optimized *Ki* value; gray area encompasses the standard deviation interval of the predicted profiles; (**c**) predicted and observed mean (±standard deviation) mexiletine concentrations with (red) and without (blue) fluvoxamine. Additional comparisons of model-predicted vs. observed DDI outcomes can be found in Supplementary Material S3. CYP, cytochrome P450.

The model predictions described the observed concentration–time profiles well after single and multiple dosing. DDI simulations with fluvoxamine as a CYP1A2 inhibition perpetrator, and caffeine, tizanidine, or mexiletine as victim substrates, demonstrated good to excellent predictions of the inhibitory potential of fluvoxamine on CYP1A2, with mean predicted/observed concentration ratios of around 1 (range 1.09–1.40 for AUC and 0.97–1.36 for C_max_; Figure 2a,b, and Supplementary Material S3.1).

**Figure 3:**
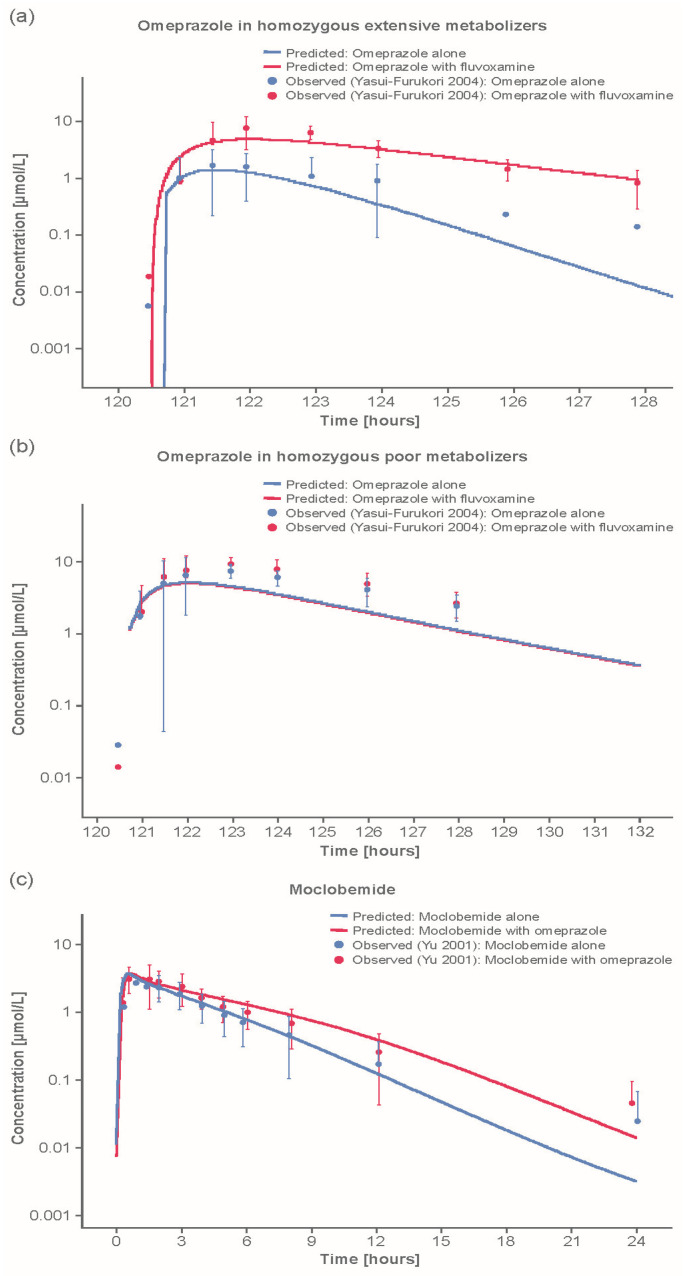
Example comparisons of model-predicted and observed CYP2C19 DDI outcomes: (**a**) observed and predicted omeprazole concentrations with and without fluvoxamine coadministration in homozygous extensive metabolizers; (**b**) observed and predicted omeprazole concentrations with and without fluvoxamine coadministration in homozygous poor metabolizers; and (**c**) simulated and observed concentration-time course for moclobemide with and without omeprazole coadministration. Additional comparisons of model-predicted vs. observed DDI outcomes can be found in Supplementary Material S3.

Good predictions were also obtained in DDI simulations with fluvoxamine as a CYP2C19 inhibition perpetrator and omeprazole or S-mephenytoin as substrate (Figure 3a–c). Strong inhibition of CYP2C19 by the twice-daily co-administration of fluvoxamine led to an observed 5.3-fold increase and predicted 6.0-fold increase in the AUC of omeprazole in extensive metabolizers (EM), and an observed 21% increase in AUC and predicted 0% increase in PM. The difference between the observed increase of 21% and predicted increase of 0% is within bioavailability limits and thus may be explained by interindividual variability. Predictions of the inhibitory potential of fluvoxamine on CYP2C19 were excellent for both EM and PM subjects (Figure 3a,b): the ratios for mean predicted/observed AUC and C_max_ were all around 1 (range: 0.83–1.13 for AUC and 0.89–1.33 for C_max_; Table 3). 

### 3.2. Omeprazole

Similar to other approaches from the literature [11], a PBPK model for omeprazole as racemate and for the single enantiomers esomeprazole and R-omeprazole was developed with two parallel metabolic pathways via CYP2C19 and CYP3A4 as linear processes. Furthermore, a CYP2C19 autoinhibitory process via time-dependent inhibition was included. The omeprazole model was intended to be used as a substrate or perpetrator. Data from 34 clinical studies or study subgroups after intravenous and oral administration using different formulations were used for model development and qualification, with nine studies or study subgroups assigned to the training set (Supplementary Material S1.2). CYP2C19 expression in gut was lowered to account for the higher-than-expected oral bioavailability of the R-omeprazole with expression levels from PK-Sim databases [12].

Model predictions described well the observed concentration–time profiles after single and multiple doses esomeprazole/R-omeprazole in both CYP2C19 EMs and PMs. In addition, DDI simulations with omeprazole as perpetrator and moclobemide as a victim substrate demonstrated a good prediction of moclobemide levels (Figure 3c). Predicted/observed ratios were in excellent agreement (1.07 for AUC, 0.89 for C_max_; Table 3). 

DDI simulations with omeprazole as victim substrate and moclobemide as perpetrator also demonstrated a good prediction of moclobemide action on CYP2C19 and CYP3A4 (Table 3). Although C_max_ levels for EMs were slightly underpredicted (predicted/observed ratio: 0.77), predictions for PMs were excellent (predicted/observed ratio: 1.02), whereas AUC levels were slightly underpredicted in both patient populations (predicted/observed ratio: 0.79 for EMs and 0.86 for PMs; Table 3).

### 3.3. S-mephenytoin

The classical path of PBPK modeling was not possible, as no PK data were available in humans after intravenous dosing, and there were only limited data following oral administration. Therefore, a PBPK model for S-mephenytoin as a victim substrate was developed with an unspecific liver clearance scaled from an in vivo measured apparent clearance [13,14] as the main clearance process and without further fitting of any other model parameter. For qualification, data from five clinical studies after oral administration using different studies were used (Supplementary Material S1.3).

DDIs with fluvoxamine as perpetrator were simulated, and simulations were used to investigate several values found in the literature for *K_i_*. Using a *K_i_* value reflecting the in vivo unbound situation for fluvoxamine with S-mephenytoin as a victim led to excellent DDI predictions (predicted/observed ratios ranged from 1.0–1.17 for AUC and 1.21–1.33 for C_max_). Default settings for CYP2C19 expression in the gut were used in these DDI simulations, because a reduced CYP2C19 expression in the gut, as used for the omeprazole model, was found to have only minor impact on model DDI predictions.

### 3.4. Moclobemide 

A PBPK model for moclobemide was developed with two metabolic pathways: saturable metabolism via CYP2C19 and an unspecified metabolic route (most likely flavin-containing monooxygenase 3 metabolism) [15,16,17]. In addition, a time-dependent autoinhibition (TDI) pathway was included to improve the description of observed concentrations after multiple dosing. The moclobemide model was intended to be used as both substrate and perpetrator. For model development and qualification, data from nine clinical studies or study subgroups after intravenous and oral administration using different formulations were used, with eight studies assigned to the training set (Supplementary Material S1.4).

DDI simulations with moclobemide as perpetrator and omeprazole as victim substrate demonstrated a good prediction of omeprazole levels for different CYP2C19 phenotypes. As previously noted, C_max_ levels for EMs were slightly underpredicted, while predictions for PMs (ratios: 0.86 for AUC, and 1.02 for C_max_) were excellent. DDI simulations with moclobemide as victim substrate and omeprazole as perpetrator demonstrated a good prediction of moclobemide levels (predicted/observed ratios: 1.07 for AUC, and 0.88 for C_max_). In contrast to R-omeprazole, the impact of reducing CYP2C19 relative expression in gut had a minimal effect on predicting moclobemide exposure.

### 3.5. Tizanidine 

A PBPK model for tizanidine as a substrate with linear CYP1A2 metabolism as the only metabolic pathway was developed that accurately predicted the concentration–time profiles following single and multiple dosing of tizanidine. Data from nine clinical studies/study subgroups after oral administration using different formulations were used for model development and qualification, with seven studies assigned to the training set (Supplementary Material S1.5).

DDI simulations with tizanidine as the victim substrate and fluvoxamine as the perpetrator demonstrated a good prediction of tizanidine concentrations (predicted/observed ratios: 1.18 for AUC, and 1.36 for C_max_) when using an optimized value of fluvoxamine *K_i_* (Figure 2b). However, DDI simulations with tizanidine as the victim substrate and mexiletine as the perpetrator underpredicted tizanidine concentrations (predicted/observed ratios: 0.63 for both AUC and C_max_). DDI simulations with tizanidine as the victim substrate and ethinylestradiol as the perpetrator required the inclusion of a TDI mechanism for ethinylestradiol to describe the observed data accurately (Supplementary Material S2.5), resulting in a good prediction of tizanidine levels (predicted/observed ratios: 0.96 for AUC and 1.09 for C_max_; Table 3). 

### 3.6. Mexiletine

The mexiletine model included three parallel metabolic pathways via CYP2D6, CYP1A2, and an unspecific clearance as linear processes. Data from 17 clinical studies or study subgroups after intravenous and oral administration using different formulations were used for model development and qualification, with 13 studies assigned to the training set (Supplementary Material S1.6).

The model was able to describe concentration–time profiles of CYP2D6 EM and PM, and it was able to predict drug accumulation after multiple doses in both phenotypes, despite a slight underprediction of clearance for EM subjects (predicted/observed AUC ratio: 0.76 in EM, vs. 1.01 in PM); however, C_max_ was well predicted by the model (predicted/observed C_max_ ratio: 1.13 in EMs and 1.10 in PMs). 

DDI simulations with mexiletine as victim substrate and fluvoxamine as perpetrator demonstrated an excellent prediction of mexiletine levels (predicted/observed ratios: 1.12 for AUC and 1.00 for C_max_; Table 3). In contrast, DDI simulations with mexiletine as perpetrator and caffeine or tizanidine as victims demonstrated an underprediction of levels for both victims (predicted/observed ratios for caffeine: 0.56 for AUC, and 0.53 for C_max_; predicted/observed ratios for tizanidine: 0.63 for AUC, and 0.63 for C_max_).

### 3.7. Ethinylestradiol 

The model comprised five parallel metabolic pathways via CYP3A4, 1A2, 2C9, and 2C19 as linear processes and uridine 5′-diphosphoglururonosyl-transferase 1A1 as a saturable process. For model development and qualification, data from 20 clinical studies or study subgroups after intravenous and oral administration using different formulations were used with 13 studies assigned to the training set (Supplementary Material S1.7).

The ethinylestradiol model was able to accurately predict the concentration–time profiles following single and multiple dosing. In addition, during the model evaluation, a TDI mechanism on CYP1A2 was introduced to describe tizanidine DDI study data. 

DDI simulations with ethinylestradiol as perpetrator and caffeine or tizanidine as victims demonstrated a good prediction of both substrates (predicted/observed ratios for caffeine: 1.75 for AUC and 0.94 for C_max_ and predicted/observed ratios for tizanidine: 0.96 for AUC, and 1.09 for C_max_; Table 3). 

## 4. Discussion

In this study, PBPK modeling was used to develop a qualified DDI network for CYP2C19 and CYP1A2 interactions. For this purpose, template models of fluvoxamine, S-mephenytoin, moclobemide, omeprazole, mexiletine, tizanidine, and ethinylestradiol were built and qualified with preclinical and clinical data from literature and clinical trial data when available. Predicted concentration–time profiles for perpetrator and victim drugs, as well as DDI AUC and C_max_ ratios confirmed that the developed PBPK models were well suited to characterize the CYP2C19 and CYP1A2 DDI network over the full range of reported DDI studies (Table 3). These models integrate the current knowledge on relevant pharmacokinetic mechanisms, including the impact of different CYP2C19 phenotypes. However, the focus of this manuscript was to build an inhibitory network, and therefore, modeling of CYP2C19 and CYP1A2 induction was not within the intended scope. CYP induction is a complex process and so consequently difficult to model. Therefore, it is common practice to gain confidence in CYP substrates models by first studying and modeling the inhibitory interactions. In the future, this work could be expanded to include an induction network, as was done by Britz et al [18].

*K_i_* values have a very high impact on the extent of the predicted DDI network. A range of values from different sources were available for the perpetrator–victim interactions, including measured in vitro values for *K_i_* or 50% inhibitory concentrations from pooled human liver microsomes, supersomes, baculosomes, or bactosomes. For fluvoxamine, *K_i_* values from in vivo DDI studies were converted to represent unbound drug levels, and both corrected and uncorrected in vivo and in vitro *K_i_* were investigated to identify the most suitable ones to describe the DDI. Most *K_i_* values used in the final models were derived from in vitro values reported in the literature. However, in cases where *K_i_* values had to be estimated to improve the description of observed data, resultant *K_i_* values remained generally in range of the reported literature values for *K_i_* (Table 4 and Supplementary Material S1).

### 4.1. Strong CYP1A2 and CYP2C19 Inhibition

Fluvoxamine is listed in the FDA table of strong clinical index inhibitors for both CYP2C19 and CYP1A2 and is commonly used in prospective clinical DDI studies. Concurrently with this investigation, a PBPK model for fluvoxamine that investigated the CYP1A2 inhibition pathway alone was developed using similar data to this investigation [1]. Accordingly, the final model structure and the fitted parameters for both model alternatives were very similar, and the two model alternatives lead to similar results for DDI prediction via CYP1A2. This clearly underlines the robustness of PBPK modeling as a method for extrapolation and prediction based on given datasets and a priori information. Notably, a *K_i_* of 10 nM for the strong inhibition of CYP1A2 by fluvoxamine in the model of Britz et al. [18] was used to describe the impact on caffeine and theophylline as victim substrate. In the fluvoxamine model presented here, a *K_i_* value of 2.97 nM was used for both caffeine and mexiletine as substrate. This *K_i_* value was reported as an in vivo unbound value for *K_i_* [19]. As a comparison, 100 mg of fluvoxamine administered qd exhibits a trough unbound concentration corresponding to around 36 nM. However, for tizanidine, the *K_i_* (0.9 nM) had to be adjusted to capture the data. 

DDI simulations with CYP1A2 substrates caffeine, tizanidine, and mexiletine demonstrated excellent prediction of the inhibitory potential of fluvoxamine on CYP1A2 (predicted/observed AUC and C_max_ ratios of around 1, and within 2-fold). Interestingly, the literature data for the fluvoxamine–caffeine DDI appear to conflict with each other. However, the elevated pre-dose caffeine concentrations in one of the published clinical studies [23] could be explained by assuming non-compliance of the study subjects to the clinical trial protocol by not refraining completely from caffeine-containing beverages; this is an excellent example of how PBPK can be used as tool for hypothesis testing and explaining unforeseen clinical study results.

In addition to confirming assumptions used in the existing fluvoxamine model [18], to our knowledge, this analysis is the first to investigate the strong CYP2C19 inhibition pathway using a whole-body PBPK model and several reference substrates in a DDI network setting. DDI predictions with fluvoxamine as an inhibitor of both CYP2C19 and CYP1A2 metabolism were successful when in vivo *K_i_* values corrected for protein binding were used to predict inhibition. In vitro *K_i_* values for the inhibition of both CYP isoforms corrected for protein binding led to gross underprediction. This is consistent with the literature, where the inhibition potency in vivo was found to be greater than predicted in vitro data alone for both CYP2C19 and CYP1A2 when inhibited by fluvoxamine [19,24]. The ratio of in vitro/in vivo *K_i_* values (both corrected for protein binding) is 40 and 10 for CYP2C19 and CYP1A2, respectively. If this in vitro/in vivo ratio is included as correction factor to calculate the in vivo unbound *K_i_*s as done in Table (Supplementary Material S1.1.2), the in vivo unbound *K_i_*s and in vitro calculated to in vivo unbound *K_i_*s appear to be in the same range. Reasons for this difference between in vivo and in vitro have been discussed in the literature [24,25] and include possible inhibition by metabolites, general environmental differences between in vitro and in vivo enzyme systems, partitioning into organelles (e.g., lysosomal trapping) or cellular membranes, and active uptake processes altering local concentrations. 

### 4.2. Moderate to Weak CYP2C19 Inhibition

For moderate CYP2C19 inhibition, no clinical index inhibitor or example substances are listed in the FDA tables. However, omeprazole is listed as an example of a weak CYP2C19 inhibitor, and in some publications, the CYP2C19 inhibition effect of omeprazole and moclobemide has been described as weak to moderate, depending on the formulation and administration protocol. Thus, this analysis provided an opportunity to cross validate both moclobemide and omeprazole as perpetrators and victims for CYP2C19 metabolism and inhibition in DDI simulations. Moreover, the omeprazole model could be qualified by predictions with fluvoxamine as a strong CYP2C19 inhibitor to foster the definition of the CYP2C19 pathway. 

PBPK models for omeprazole have been described in the literature before. However, these were either only for the racemate [26] or they were using a minimal PBPK modeling approach with its inherent assumptions and limitations [11]. Here, we describe the time-dependent autoinhibition effect of enantiomers and mutual inhibition effect of racemic drug in a whole-body PBPK model for the first time, allowing confirmation of some of the assumptions used in the minimal models and creating a more universal applicable model structure. For moclobemide, a minimal PBPK model was recently developed with its assumptions and limitations [27]. Using this whole-body PBPK model for moclobemide, the inclusion of time-dependent autoinhibition of CYP2C19 metabolism improved the description of the clinical data compared to the minimal model, although it cannot be excluded that this effect was caused by a metabolite of moclobemide. Taken together, this model offers a universally applicable model structure and an improved description of clinical data. For both compounds, *K_i_* values from in vitro experiments corrected for the protein binding could be used to describe the moderate-to-low CYP2C19 inhibition. 

### 4.3. Moderate CYP1A2 Inhibition

For moderate CYP1A2 inhibition, no clinical index inhibitor is listed in the FDA table, although mexiletine is listed as an example of a clinical inhibitor showing moderate CYP1A2 inhibition. As mexiletine is partially metabolized by CYP1A2, it can be used as CYP1A2 victim substrate as well, and the prediction of the extent of this interaction can be used for additional qualification of a strong CYP1A2 inhibitor model such as fluvoxamine. To the best of our knowledge, no whole-body PBPK model for mexiletine has previously been described in the literature. The mexiletine model was qualified as a moderate CYP1A2 inhibitor by predicting the interaction with two CYP1A2 substrates, caffeine and tizanidine. While PBPK models exist for caffeine, which proved to be suitable as a DDI substrate for strong CYP1A2 inhibition [18], to our knowledge, no PBPK model for tizanidine has been described to date. Interestingly, the estimated *K_i_* value for ethinylestradiol as a moderate CYP1A2 inhibitor (0.48 µM) is in the same range as the in vitro corrected value used for mexiletine (0.28 µM) with regard to all tested substrates (caffeine and tizanidine).

Overall, DDI predictions with mexiletine as an inhibitor tend to lead to an underprediction of both AUC and C_max_ values, but all were within 2-fold of observed values. The effect of food on the tizanidine PK when taken as a capsule could be described with an accurate prediction of T_max_ but with a 1.5- to 2-fold underprediction of C_max_. Therefore, any DDI simulations including food and capsule formulation should be interpreted with caution. 

PBPK models for ethinylestradiol have been previously described in the literature for various applications. However, this is the first whole-body PBPK model for ethinylestradiol as perpetrator, demonstrating usefulness to describe DDIs impacting CYP1A2 metabolism.

During model evaluation, a TDI mechanism on CYP1A2 was introduced to describe the clinical interaction with caffeine and tizanidine. Two values for enzyme inactivation rate (*K_inact_*) were tested, 100 min^−1^ and 200 min^−1^; DDI simulations with ethinylestradiol as the inhibitor and caffeine as the victim substrate demonstrated a good prediction using a *K_inact_* of 100 min^−1^, whereas a *K_inact_* of 200 min^−1^ was better for simulations with tizanidine as victim substrate. When using ethinylestradiol as a perpetrator with a new victim drug, the recommendation is to test both of these values and discuss the potential impact, as the value resulting in the best prediction might depend on the substrate. Although the use of TDI for ethinylestradiol has not been reported previously, the TDI mechanism was necessary to describe the observed interaction with tizanidine. 

### 4.4. Impact of Genetic Polymorphism

The ability of PBPK models to adequately predict PK changes associated with genetic polymorphisms is of critical importance for drug safety and represents a major challenge, with more than 19 allelic variants of the human CYP2C19 gene identified, many leading to marked reduction or an absence of enzyme function [28]. The prevalence of PMs among Caucasians is approximately 3% for CYP2C19 and 10% for CYP2D6, and it is considerably higher in some other ethnic groups [28]. Although many PBPK models are available for the individual enzymes, our models were able to account for the impact of polymorphism within a complex PBPK network. DDI simulations demonstrated an excellent predictive ability for the inhibition of CYP2C19 by fluvoxamine as the perpetrator and omeprazole or S-mephenytoin as victims, in both EM and PM subjects; and, also in PM subjects with moclobemide as the perpetrator and omeprazole as the victim, although levels in EM subjects were slightly underpredicted (C_max_ observed/predicted ratio 0.77). Similarly for CYP2D6, the PBPK model provided excellent predictions for mexiletine in both EM and PM subjects, with a slight underprediction of drug clearance with multiple dosing in EM subjects. The improved understanding of the impact of these genetic polymorphisms provided by our model may help inform clinical trial design and enable potential recommendations for dose adaptations.

## 5. Conclusions

In summary, a whole-body PBPK DDI network for CYP2C19 and CYP1A2 interactions was developed based on template models of fluvoxamine, S-mephenytoin, moclobemide, omeprazole, mexiletine, tizanidine, and ethinylestradiol that were transparently built and qualified with data from the literature. Predicted concentration–time profiles of the perpetrator and victim drugs, DDI, AUC, and C_max_ ratios generally corresponded well with observed data, demonstrating that the models characterized the CYP2C19 and CYP1A2 DDI network over the whole range of DDI studies included in this investigation. The presented models are fully documented and are provided as Supplementary Material to this paper (Supplement S3) and will be available in the GitHub repository. These models integrate the current knowledge on relevant pharmacokinetic mechanisms, including the impact of different CYP2C19 phenotypes and genetic polymorphisms, and they will extend our understanding of the DDI potential of investigational drugs and inform the design of clinical DDI trials. 

## Figures and Tables

**Table 1:** Drug–drug interaction (DDI) studies predicted and used for qualification of the network.

**Strong CYP2C19 inhibition**
Fluvoxamine–omeprazole Fluvoxamine–S-mephenytoin
**Strong CYP1A2 inhibition**
Fluvoxamine–caffeine Fluvoxamine–tizanidine Fluvoxamine–mexiletine
**Moderate CYP2C19 inhibition**
Omeprazole–moclobemide Moclobemide–omeprazole
**Moderate CYP1A2 inhibition**
Mexiletine–caffeine Mexiletine–tizanidine Oral contraceptives (ethinylestradiol)–caffeine Oral contraceptives (ethinylestradiol)–tizanidine

**Table 2:** General step-wise workflow for model development.

Predict intravenous profiles based on in vitro data alone. Optimize distribution model and metabolism parameters based on intravenous clinical data if available. Predict oral data following dissolved formulations. Optimize absorption parameters (intestinal permeability, solubility) if necessary to describe oral clinical data. Predict oral data following non-dissolved formulations. Fit dissolution function if necessary, to describe oral clinical data. Model qualification using external dataset not used for model building. Predict DDI with other templates of the network and check with clinical data.

DDI, drug–drug interaction.

**Table 3:** Overview of results of DDI predictions of template models.

Inhibitor Category	Inhibitor	Substrate	Pred AUC_R_/ Obs AUC_R_	Pred C_maxR_/ Obs C_maxR_
Strong CYP2C19	Fluvoxamine	Omeprazole	EM: 1.13 * PM: 0.83	EM: 1.00 * PM: 0.89
		S-mephenytoin	27.5mg Fl: 1.00 64.1mg Fl: 1.03	27.5mg Fl: 1.21 64.1 mg Fl: 1.33
Strong CYP1A2	Fluvoxamine	Caffeine	1.40	1.01
		Tizanidine	1.18 ^a^	1.36 ^a^
		Mexiletine	1.12	1.00
Moderate CYP2C19	Omeprazole	Moclobemide	1.07	0.89
	Moclobemide	Omeprazole	EM: 0.79 PM: 0.86	EM: 0.77 PM: 1.02
Moderate CYP1A2	Mexiletine	Caffeine	0.61	0.53
		Tizanidine	0.63	0.63
	Ethinylestradiol	Caffeine	1.75 ^b^	0.94 ^b^
		Tizanidine	+CI: 0.26 +TDI: 0.96 ^c^	+CI: 0.33 +TDI: 1.09 ^c^

* This ratio changed in the current PK-Sim version 9.1 (see Supplementary Material S3). Fl, fluvoxamine dose; CI, competitive inhibition; EM, extensive metabolizer; PM, poor metabolizer; TDI: time-dependent inhibition ^a^ Using optimized *K_i_* = 0.8697 nM, ^b^ *K_inact_* = 100 min^−1^, ^c^ *K_inact_* = 200 min^−1^.

**Table 4:** Overview of *K_i_* values used in the DDI network.

Inhibitor Category	Inhibitor	Substrate	*K_i_*
**Strong CYP2C19**	Fluvoxamine	Omeprazole	3.6 nM [19]
		S-mephenytoin	2.6 nM [19]
**Strong CYP1A2**	Fluvoxamine	Caffeine	2.97 nM [19]
		Tizanidine	0.8697 nM ^a^
		Mexiletine	2.97 nM [19]
**Moderate CYP2C19**	Moclobemide	Omeprazole	203.83 µM [20] (TDI 94.85 µM)
	Omeprazole	Moclobemide	S-ome: 3.1 µM [11,21] (TDI 0.3 µM) R-ome: 5.3 µM [11,21] (TDI 1.6 µM)
**Moderate CYP1A2**	Mexiletine	Caffeine	0.28 µM [22]
		Tizanidine	0.28 µM [22]
	Ethinylestradiol	Caffeine	0.48 µM ^b^
		Tizanidine	0.48 µM ^b^

*K_i_*, inhibitor constant; TDI, time-dependent inhibition. ^a^ Lowest literature value = 0.9 nM, ^b^ Literature value = 10.6 µM.

## Supplementary Materials

The following are available online at https://www.mdpi.com/1999-4923/12/12/1191/s1, Supplement S1. Summary of data and data sources used in model development and evaluation. Supplement S2a: Model development and evaluation (CYP2C19 predominant): fluvoxamine, omeprazole, moclobemide, S-mephenytoin. Supplement S2b: Model development and evaluation (CYP1A2 predominant): mexiletine, ethinylestradiol, caffeine, tizanidine. Supplement S3: Final parameters and structure of each model and results of DDI predictions.

## References

[1] Goldstein J.A. (2001). Clinical Relevance of Genetic Polymorphisms in The Human Cyp2c Subfamily. Br. J. Clin. Pharmacol..

[2] Desta Z., Zhao X., Shin J.G., Flockhart D.A. (2002). Clinical Significance of The Cytochrome P450 2c19 Genetic Polymorphism. Clin. Pharmacokinet..

[3] Zhou S.F., Yang L.P., Zhou Z.W., Liu Y.H., Chan E. (2009). Insights into The Substrate Specificity, Inhibitors, Regulation, And Polymorphisms and the Clinical Impact of Human Cytochrome P450 1a2. AAPS J..

[4] Bertilsson L. (1995). Geographical/Interracial Differences in Polymorphic Drug Oxidation. Current State of Knowledge of Cytochromes P450 (Cyp) 2d6 and 2c19. Clin. Pharmacokinet..

[5] Zhao P., Zhang L., Grillo J.A., Liu Q., Bullock J.M., Moon Y.J., Song P., Brar S.S., Madabushi R., Wu T.C. (2011). Applications of Physiologically Based Pharmacokinetic (Pbpk) Modeling and Simulation During Regulatory Review. Clin. Pharmacol. Ther..

[6] Shebley M., Sandhu P., Emami Riedmaier A., Jamei M., Narayanan R., Patel A., Peters S.A., Reddy V.P., Zheng M., de Zwart L. (2018). Physiologically Based Pharmacokinetic Model Qualification and Reporting Procedures for Regulatory Submissions: A Consortium Perspective. Clin. Pharmacol. Ther..

[7] Zhao P., Rowland M., Huang S.M. (2012). Best Practice in The Use of Physiologically Based Pharmacokinetic Modeling and Simulation to Address Clinical Pharmacology Regulatory Questions. Clin. Pharmacol. Ther..

[8] European Medicines Agency (2012). Guideline on the Investigation of Drug Interactions 2012.

[9] U.S. Food and Drug Administration (2017). Clinical Drug Interaction Studies—Study Design, Data Analysis, And Clinical Implications. Guidance for Industry. Draft Guidance.

[10] U.S. Food and Drug Administration (2017). Drug Development and Drug Interactions: Table of Substrates, Inhibitors and Inducers.

[11] Wu F., Gaohua L., Zhao P., Jamei M., Huang S.M., Bashaw E.D., Lee S.C. (2014). Predicting Nonlinear Pharmacokinetics of Omeprazole Enantiomers and Racemic Drug Using Physiologically Based Pharmacokinetic Modeling and Simulation: Application to Predict Drug/Genetic Interactions. Pharm. Res..

[12] Hassan-Alin M., Andersson T., Bredberg E., Rohss K. (2000). Pharmacokinetics of Esomeprazole After Oral and Intravenous Administration of Single and Repeated Doses to Healthy Subjects. Eur. J. Clin. Pharmacol..

[13] Olivares-Morales A., Ghosh A., Aarons L., Rostami-Hodjegan A. (2016). Development of A Novel Simplified Pbpk Absorption Model to Explain the Higher Relative Bioavailability of The Oros(R) Formulation of Oxybutynin. AAPS J..

[14] Adedoyin A., Arns P.A., Richards W.O., Wilkinson G.R., Branch R.A. (1998). Selective Effect of Liver Disease on the Activities of Specific Metabolizing Enzymes: Investigation of Cytochromes P450 2c19 and 2d6. Clin. Pharmacol. Ther..

[15] Gram L.F., Guentert T.W., Grange S., Vistisen K., Brosen K. (1995). Moclobemide, A Substrate of Cyp2c19 And an Inhibitor of Cyp2c19, Cyp2d6, And Cyp1a2: A Panel Study. Clin. Pharmacol. Ther..

[16] Mayersohn M., Guentert T.W. (1995). Clinical Pharmacokinetics of the Monoamine Oxidase-A Inhibitor Moclobemide. Clin. Pharmacokinet..

[17] Hoskins J., Shenfield G., Murray M., Gross A. (2001). Characterization of Moclobemide N-Oxidation in Human Liver Microsomes. Xenobiotica.

[18] Britz H., Hanke N., Volz A.K., Spigset O., Schwab M., Eissing T., Wendl T., Frechen S., Lehr T. (2019). Physiologically-Based Pharmacokinetic Models for Cyp1a2 Drug-Drug Interaction Prediction: A Modeling Network of Fluvoxamine, Theophylline, Caffeine, Rifampicin, And Midazolam. CPT Pharmacomet. Syst. Pharmacol..

[19] Iga K. (2016). Dynamic and Static Simulations of Fluvoxamine-Perpetrated Drug-Drug Interactions Using Multiple Cytochrome P450 Inhibition Modeling, And Determination of Perpetrator-Specific Cyp Isoform Inhibition Constants and Fractional Cyp Isoform Contributions to Victim Clearance. J. Pharm. Sci..

[20] Nielsen K.K., Flinois J.P., Beaune P., Brosen K. (1996). The Biotransformation of Clomipramine In Vitro, Identification of the Cytochrome P450s Responsible for the Separate Metabolic Pathways. J. Pharmacol. Exp. Ther..

[21] Liu K.H., Kim M.J., Shon J.H., Moon Y.S., Seol S.Y., Kang W., Cha I.J., Shin J.G. (2005). Stereoselective Inhibition of Cytochrome P450 Forms by Lansoprazole and Omeprazole In Vitro. Xenobiotica.

[22] Wei X., Dai R., Zhai S., Thummel K.E., Friedman F.K., Vestal R.E. (1999). Inhibition of Human Liver Cytochrome P-450 1a2 By the Class Ib Antiarrhythmics Mexiletine, Lidocaine, And Tocainide. J. Pharmacol. Exp. Ther..

[23] Culm-Merdek K.E., Von Moltke L.L., Harmatz J.S., Greenblatt D.J. (2005). Fluvoxamine Impairs Single-Dose Caffeine Clearance Without Altering Caffeine Pharmacodynamics. Br. J. Clin. Pharmacol..

[24] Yao C., Kunze K.L., Trager W.F., Kharasch E.D., Levy R.H. (2003). Comparison of in Vitro And in Vivo Inhibition Potencies of Fluvoxamine Toward Cyp2c19. Drug Metab. Dispos..

[25] Yao C., Kunze K.L., Kharasch E.D., Wang Y., Trager W.F., Ragueneau I., Levy R.H. (2001). Fluvoxamine-Theophylline Interaction: Gap Between In Vitro And In Vivo Inhibition Constants Toward Cytochrome P4501a2. Clin. Pharmacol. Ther..

[26] Lukacova V., Parrot N., Howard M., Woltosz W., Bolger M. (2010). Prediction of Omeprazole’s Disposition and Drug-Drug Interactions Using A Physiologically-Based Pharmacokinetic Model.

[27] Reddy V.P., Jones B.C., Colclough N., Srivastava A., Wilson J., Li D. (2018). An Investigation into The Prediction of The Plasma Concentration-Time Profile and Its Interindividual Variability for A Range of Flavin-Containing Monooxygenase Substrates Using A Physiologically Based Pharmacokinetic Modeling Approach. Drug Metab. Dispos..

[28] Wijnen P.A., Op den Buijsch R.A., Drent M., Kuijpers P.M., Neef C., Bast A., Bekers O., Koek G.H. (2007). Review Article: The Prevalence and Clinical Relevance of Cytochrome P450 Polymorphisms. Aliment. Pharmacol. Ther..

