# Supplementary material for: A Physiologically-Based Pharmacokinetic (PBPK) Model Network for the Prediction of CYP1A2 and CYP2C19 Drug–Drug–Gene Interactions with Fluvoxamine, Omeprazole, S-mephenytoin, Moclobemide, Tizanidine, Mexiletine, Ethinylestradiol, and Caffeine
Source: Pharmaceutics. 2020 Dec 8;12(12):1191. doi: 10.3390/pharmaceutics12121191 (PMC7764797; doi:10.3390/pharmaceutics12121191)
Supplement: Supplementary file 1 [file pharmaceutics-12-01191-s001.zip › pharmaceutics-1010984 supplementary/PBPK_manuscript_supplement S1_Pharmaceutics.docx]

**Electronic Supplementary Material**

# **Supplement S1: Summary of** **data and data sources used in model development and evaluation**

# **Section S1.1. Fluvoxamine**

## **Table S1.1.1: Physicochemical properties of fluvoxamine**

| Parameter | Value | Source | Comment |
| --- | --- | --- | --- |
| MW free base  Fluvoxamine maleate | 318.3 g/mol  434.4 g/mol | Website: drugbank.ca/drugs/DB00176 | Dose of commercial product usually refers to amount of fluvoxamine maleate – conversion factor is 0.733 |
| BCS class | I | Website: ddfint.org/search.cfm (accessed 03 June 2019) |  |
| Aqueous solubility at pH 7.4 | 0.06 mg/mL | Website: pubchem.ncbi.nlm.nih.gov/compound/9560989#section=Chemical-and-Physical-Properties | Experimental |
| LogP | 3.38 | Alqahtani 2016[^1^](#_ENREF_1) | Alqahtani used a value predicted by the ADMET predictor;  Similar values in other sources (e.g DrugBank: 3.2) |
| fu | 0.23 | Alqahtani 2016[^1^](#_ENREF_1)  Perucca 1994[^2^](#_ENREF_2) | Similar value reported in Luvox Label: “approx. 80% bound” |
| pKa | 9.16 | Alqahtani 2016[^1^](#_ENREF_1) |  |
| Ki_CYP1A2 | 2.97 nmol/L | Iga 2016[^3^](#_ENREF_3) | Represents unbound value from in vivo experiments; presumably the most robust source of Ki as it eliminates the need for in vitro-in vivo scaling |

## **Table S1.1.2: Fluvoxamine literature Ki values for CYP inhibition**

| **CYP** | **Substrate** | **Source** | **in vivo Ki [nM]** | **Literature reference** |
| --- | --- | --- | --- | --- |
| 2C19 | S-omeprazole | in vitro HLM calculated to in vivo unbound | 1.63 | Foti et al 2008^[4](#_ENREF_4)^ |
|  | R-omeprazole | in vitro HLM calculated to in vivo unbound | 0.37 | Foti et al 2008^[4](#_ENREF_4)^ |
|  | Omeprazole | recalculated from in vivo data | 3.60 | Iga 2016[^3^](#_ENREF_3) |
|  | S-mephenytoine | in vitro HLM calculated to in vivo unbound | 2.08 | Yao 2003^[5](#_ENREF_5)^ |
|  | S-mephenytoine | in vitro HLM calculated to in vivo unbound | 1.92 | Yao 2003^[5](#_ENREF_5)^ |
|  | S-mephenytoine | in vitro HLM calculated to in vivo unbound | 1.84 | Yao 2003^[5](#_ENREF_5)^ |
|  | S-mephenytoine | in vitro supersomes calculated to in vivo unbound | 2.05 | Yao 2003^[5](#_ENREF_5)^ |
|  | S-mephenytoine | in vitro supersomes calculated to in vivo unbound | 1.77 | Yao 2003^[5](#_ENREF_5)^ |
|  | S-mephenytoine | in vivo calculated to in vivo unbound | 1.89 | Yao 2003^[5](#_ENREF_5)^ |
|  | S-mephenytoine | recalculated from in vivo data | 2.60 | Iga 2016[^3^](#_ENREF_3) |
|  | S-mephenytoine | in vitro supersomes calculated to in vivo unbound | 0.34 | Foti et al 2008^[4](#_ENREF_4)^ |
|  | S-mephenytoine | in vitro HLM calculated to in vivo unbound | 1.87 | Obach 2006^[6](#_ENREF_6)^ |
|  | S-mephenytoine | in vitro bactosomes calculated to unbound | 0.79 | McGinnity 2008^[7](#_ENREF_7)^ |
| 1A2 | Caffeine | in vitro HLM calculated to in vivo unbound | 1.6 | Rasmussen 1998^[8](#_ENREF_8)^ |
|  |  | recalculated from in vivo data | 2.97 | Iga 2016[^3^](#_ENREF_3) |
|  | Theophylline | in vitro HLM calculated to in vivo unbound | 3.6 | Yao 2003^[5](#_ENREF_5)^ |
|  |  | in vitro HLM calculated to in vivo unbound | 3.5 | Yao 2003^[5](#_ENREF_5)^ |
|  |  | in vitro HLM calculated to in vivo unbound | 3.8 | Yao 2003^[5](#_ENREF_5)^ |
|  |  | in vitro supersomes calculated to in vivo unbound | 3.6 | Yao 2003^[5](#_ENREF_5)^ |
|  |  | in vivo calculated to in vivo unbound | 3.6 | Yao 2003^[5](#_ENREF_5)^ |
|  |  | recalculated from in vivo data | 9.8 | Iga 2016[^3^](#_ENREF_3) |
|  | Tizanidine | recalculated from in vivo data | 3.3 | Iga 2016[^3^](#_ENREF_3) |
|  |  | fitted to in vivo data | **0.8697** | **PK-Sim** |
|  | Phenacetin | in vitro HLM calculated to unbound | 15.0 | Von Moltke 1996[^9^](#_ENREF_9) |
|  |  | in vitro HLM calculated to unbound | 12.2 | Brøsen 1993[^10^](#_ENREF_10) |
|  |  | in vitro HLM calculated to unbound | 0.9 | Karjalainen 2008^[11](#_ENREF_11)^ |
| 3A4 | Midazolam | in vitro HLM calculated to unbound | 2.6 | Foti 2010[^12^](#_ENREF_12) |

Items in bold were used as part of the final model.

## **Table S1.1.3: Fluvoxamine clinical data used in model development and evaluation**

| **Source** | **Route** | **Dose [mg]/**  **Schedule*** | **Population** | **Age [yrs] (mean)** | **Weight [kg] (mean)** | **Sex** | **N** | **Formulation** | **Comment** | **Model development step # and purpose** |
| --- | --- | --- | --- | --- | --- | --- | --- | --- | --- | --- |
| Culm-Merdek 2005^[13](#_ENREF_13)^ | p.o. | 100 b.i.d. | HV | 50 | 82 | m/f | 7 | e.c. Tablets | NA | 7. DDI predictions with caffeine as victim drug |
| Carillo 1996^[14](#_ENREF_14)^ | p.o. | 50 | HV | - | - | m/f | 5 | e.c. Tablets | Non-smoking EM + PM / Smoking EM | 6. Model refinement; adding CYP1A2 pathway (PM) |
| Spigset 1997^[15](#_ENREF_15)^ | p.o. | 50 | HV | - | - |  | 5 | e.c. Tablets | PM | 6. Model refinement; adding CYP1A2 pathway |
| Spigset 1997^[15](#_ENREF_15)^ | p.o. | 50 | HV | - | - |  | 5 | e.c. Tablets | EM |  |
| Culm-Merdek 2005^[13](#_ENREF_13)^ | p.o. | 250 | HV | 50 | 82 | m/f | 7 | Capsule | Caffeine with/without fluvoxamine | 7. DDI Predictions with caffeine as victim drug |
| DeVries 1992^[16](#_ENREF_16)^ | p.o. | 50 b.i.d. | HV + elderly | - | - | m/f | 25 | e.c. Tablets | - |  |
| **Iga 2015**^[17](#_ENREF_17)^ | i.v. | 50 | Japanese | - | - | - | - | - | - | **1. Fitting i.v. data and selection distribution model**  **3. Parameter estimation; intestinal permeability**  **6. Model refinement; adding CYP1A2 pathway** |
| **Iga 2015**^[17](#_ENREF_17)^ | p.o. | 50 | Japanese | - | - | - | - | - | - | **4. Fitting enteric coated tablet** |
| **Orlando 2009**^[18](#_ENREF_18)^ | p.o. | 50 | HV | 35 | 79 | male | 10 | - | - | **4. Fitting enteric coated tablet** |
| Spigset 1998^[19](#_ENREF_19)^ | p.o. | 12.5/ 25/ 50 / 100 b.i.d. | HV | 28.9 | 85.6 | male | 10 | e.c. Tablets | - | 5a. Multiple dose predictions preliminary model  6. Model refinement; adding CYP1A2 pathway |
| **DeVries 1993**^[20](#_ENREF_20)^ | p.o. | 25 / 50/ 100 | HV | - | - | male | 12 | solution | - | **2. Propagation from i.v. to oral model**  **3. Parameter estimation; intestinal permeability**  **6. Model refinement; adding CYP1A2 pathway** |
| VanHarten 1994^[21](#_ENREF_21)^ | p.o. | 50 | HV | - | - | male | 17 | capsule | - |  |
| VanHarten 1991^[22](#_ENREF_22)^ | p.o. | 50 | HV | 24 | 73 | m/f | 12 | e.c. Tablets | With / without food |  |
| **Kunii 2005**^[23](#_ENREF_23)^ | p.o. | 50 | HV | 29.7 | 69.3 | m | 10 | e.c. Tablets | - | **4. Fitting enteric coated tablet** |
| **Fukasawa 2006**^[24](#_ENREF_24)^ | p.o. | 50 | HV | 29.6 | 68.3 | m | 12 | e.c. Tablets | - | **4. Fitting enteric coated tablet** |
| Fleishaker 1994^[25](#_ENREF_25)^ | p.o. | 50 / 100 q.d. | HV | 32 | 80 | m | 20 | e.c. Tablets | - | 5b. Multiple dose predictions preliminary model  6. Model refinement; adding CYP1A2 pathway |
| Labellarte 2004^[26](#_ENREF_26)^ | p.o. | 25 / 50 / 100/ 150 b.i.d. | Adolescents | 14 | 60 | m/f | 23 | e.c. Tablets | - | 6. Model refinement; adding CYP1A2 pathway |
| **Spigset 1997**^[15](#_ENREF_15)^ | p.o. | 50 | HV | 34.7 | 66.5 | m/f | 12 | e.c. Tablets | non-smokers / smoker | **4. Fitting enteric coated tablet** |
| FDA_ClinPharmReview LuvoxCR^[27](#_ENREF_27)^ | p.o. | 100 / 200 / 300 q.d. | HV | - | - | - | 20 | e.c. Tablets / CR | - | 6. Model refinement; adding CYP1A2 pathway (s.d. and q.d. dose) |
| Jeppesen 1996^[28](#_ENREF_28)^ | p.o. | 200 | HV | 27 | - | - | 8 | Tablets | Caffeine with / without Fluvoxamine | 7. DDI Predictions with caffeine as victim drug |
| Study c13128239-01^†^[^29^](#_ENREF_29) | p.o. | 50-100 q.d. | HV | 37.4 | 74.6 | m/f | 18 | e.c. Tablets | - | Model Evaluation and Qualification |

*single dose unless otherwise specified; ^†^Study provided data for use in model evaluation/qualification only, not in model development; - respective information was not provided in the literature source.

Items marked in bold were used in the training set for the model.

# **Section S1.2. Omeprazole**

## **Table S1.2.1: Physicochemical properties of omeprazole**

| Parameter S-omeprazole | Value | Source | Comment |
| --- | --- | --- | --- |
| MW | 345.4 g/mol | Website drugbank.ca/drugs/DB00338 |  |
| BCS class | II | Zhou 2016^[30](#_ENREF_30)^ |  |
| Water solubility | 0.36 mg/mL | Website drugbank.ca/drugs/DB00338 |  |
| LogD | 2.23 | Ogilvie 2011[^31^](#_ENREF_31) | Similar values in other sources (e.g ALOGPS: 1.66-2.43, Website drugbank.ca/drugs/DB00338) |
| fu | 0.03 | Website accessdata.fda.gov/scripts/cder/drugsatfda/ [^32^](#_ENREF_32) | Label of esomeprazole obtained from Drugs@FDA |
| pKa | Acid/9.29 Base/4.77 | Website drugbank.ca/drugs/DB00338 |  |
| Kinact of time dependent inhibition on CYP2C19 | 5 L/h | Wu 2014[^33^](#_ENREF_33) | Sensitivity analysis and value of omeprazole from Ogilvie[^31^](#_ENREF_31) |
| Ki of time dependent inhibition on CYP2C19 | 0.3 µM | Wu 2014[^33^](#_ENREF_33) | Sensitivity analysis and value of omeprazole from Ogilvie[^31^](#_ENREF_31) |
| Renal elimination | 0.037 l/h | Wu 2014[^33^](#_ENREF_33) | Assumed same as omeprazole |
| Ki_CYP2C19 (competitive inhibition) | 3.1 µM | Liu 2005[^34^](#_ENREF_34) | The total Ki value reported by Liu was 3.4 umol/L and corrected with an fu_mic of 0.92 |
| **Parameter R-omeprazole** | **Value** | **Source** | **Comment** |
| MW | 345.4 g/mol | Website drugbank.ca/drugs/DB00338 |  |
| Water solubility | 0.36 mg/mL | Website drugbank.ca/drugs/DB00338 |  |
| LogD | 2.23 | Ogilvie 2011[^31^](#_ENREF_31) | Similar values in other sources (e.g ALOGPS: 1.66-2.43, website drugbank.ca/drugs/DB00338) |
| fu | 0.04 | Ogilvie 2011[^31^](#_ENREF_31) | Assumed same as omeprazole |
| pKa | Acid/9.29 Base/4.77 | Website drugbank.ca/drugs/DB00338 |  |
| Kinact of time dependent inhibition on CYP2C19 | 4 L/h | Wu 2014[^33^](#_ENREF_33) | Sensitivity analysis and value of omeprazole from Ogilvie[^31^](#_ENREF_31) |
| Ki of time dependent inhibition on CYP2C19 | 1.6 µM | Wu 2014[^33^](#_ENREF_33) | Sensitivity analysis and value of omeprazole from Ogilvie[^31^](#_ENREF_31) |
| Renal elimination | 0.037 L/h | Wu 2014[^33^](#_ENREF_33) | Assumed same as omeprazole |
| Ki_CYP2C19 (competitive inhibition) | 5.3 µM | Liu 2005[^34^](#_ENREF_34) | The total Ki value reported by Liu was 5.7 umol/L and corrected with an fu_mic of 0.92 |

## **Table S1.2.2: Omeprazole literature Ki values for CYP2C19 inhibition**

| **Substrate** | **Source** | **in vivo Ki [µM]** | **Literature reference** |
| --- | --- | --- | --- |
| S-mephenytoine | in vitro HLM calculated to unbound | 6.07 | Furuta 2001^[35](#_ENREF_35)^ |
| S-mephenytoine | in vitro HLM calculated to unbound | 2.86 | Ko 1997^[36](#_ENREF_36)^ |
| S-mephenytoine | S-omperazole - in vitro HLM calculated to unbound | **3.14** | Liu 2005^[34](#_ENREF_34)^ |
| S-mephenytoine | Rac-Omeprazole - in vitro HLM calculated to unbound | 3.23 | Liu 2005^[34](#_ENREF_34)^ |
| S-mephenytoine | R-Omeprazole - in vitro HLM calculated to unbound | **5.26** | Liu 2005^[34](#_ENREF_34)^ |
| S-mephenytoine | S-omperazole - in vitro HLM calculated to unbound | 8.19 | Li 2004^[37](#_ENREF_37)^ |
| S-mephenytoine | Rac-Omeprazole - in vitro HLM calculated to unbound | 5.90 | Li 2004^[37](#_ENREF_37)^ |
| S-mephenytoine | R-Omeprazole - in vitro HLM calculated to unbound | 5.52 | Li 2004^[37](#_ENREF_37)^ |

Items in bold were used as part of the final model.

## **Table S1.2.3: Omeprazole clinical data used in model development and evaluation**

| **Source** | **Route** | **Dose (mg) / Schedule*** | **Pop.** | **Sex** | **N** | **Formulation** | **Comment** | **Model development step # and purpose** |
| --- | --- | --- | --- | --- | --- | --- | --- | --- |
| **Omeprazole racemate** | | | | | | | | |
| **Andersson 1990**[^38^](#_ENREF_38) | i.v. | 40–80 | HV | m | 10 | solution | - | **6. Refine CYP2C19 metabolism on CYP2C19 EM data** |
| Andersson 1990^[38](#_ENREF_38)^ | p.o. | 40–80 | HV | m | 10 | Oral solution | - | Model qualification (Figure S2.11) |
| Andersson 1991^[39](#_ENREF_39)^ | p.o. | 10/20/40 q.d. | HV | m | 12 | e.c. granules | - | Model qualification (Figure S2.11) |
| **Andersson 1991**^[39](#_ENREF_39)^ | i.v. | 10/20/40 | HV | m | 12 | solution | - | **6. Refine CYP2C19 metabolism on CYP2C19 EM data** |
| Andersson 1998^[40](#_ENREF_40)^ | p.o. | 20 q.d. | HV | m | 12 | capsule | EM | Model qualification (Figure S2.11) |
| Andersson 1998^[40](#_ENREF_40)^ | p.o. | 20 q.d. | HV | m | 2 | capsule | PM | Model qualification (Figure S2.11) |
| **Oosterhuis 1992**^[41](#_ENREF_41)^ | i.v. | 40–80 | HV | m | 8 | solution | - | **6. Refine CYP2C19 metabolism on CYP2C19 EM data** |
| **Uno 2007**^[42](#_ENREF_42)^ | i.v. | 20 | HV Japanese | m/f | 6 | solution | hmEM | **6. Refine CYP2C19 metabolism on CYP2C19 EM data** |
| **Uno 2007**^[42](#_ENREF_42)^ | i.v. | 20 | HV Japanese | m/f | 6 | solution | PM | **5. Refine CYP3A4 metabolism on CYP2C19 PM data** |
| Uno 2007^[42](#_ENREF_42)^ | p.o. | 40 | HV japanese | m/f | 6 | tablet | hmEM | Model qualification (Figure S2.11) |
| Uno 2007^[42](#_ENREF_42)^ | p.o. | 40 | HV Japanese | m/f | 6 | tablet | PM | Model qualification (Figure S2.11) |
| **Regårdh 1990**^[43](#_ENREF_43)^ | i.v. | 10 | HV | m | 8 | solution | - | **5. Refine CYP3A4 metabolism on CYP2C19 PM data (ID2; PM)   6. Refine CYP2C19 metabolism on CYP2C19 EM data**  **(median and ID7; EM)** |
| Regårdh 1990^[43](#_ENREF_43)^ | p.o. | 20 | HV | m | 8 | oral solution | - |  |
| Andersson 2000^[44](#_ENREF_44)^ | p.o. | 15 q.d. | HV | - | 4 | oral solution | EM | Model qualification |
| Andersson 2000^[44](#_ENREF_44)^ | p.o. | 60 q.d. | HV | - | 5 | oral solution | PM | Model qualification |
| Hassan-Alin 2005^[45](#_ENREF_45)^ | p.o. | 20–40 q.d. | HV | - | - | oral solution | - | Model qualification (Figure S2.11) |
| Cho 2002^[46](#_ENREF_46)^ | p.o. | 20 | HV Asian | - | - | capsule | EM +/- moclobemide | Model qualification ((Figure S2.11) DDI predictions with moclobemide (Figure S3.5) |
| Cho 2002^[46](#_ENREF_46)^ | p.o. | 20 | HV Asian | - | - | capsule | PM +/- moclobemide | Model qualification (Figure S2.11) |
| Yasui-Furukori 2004^[47](#_ENREF_47)^ | p.o. | 40 | HV Japanese | m/f | 6 | omepral | hmEM +/- fluvoxamine | Model qualification (Figure S3.4)  DDI predictions with fluvoxamine (Figure S3.4) |
| Yasui-Furukori 2004^[47](#_ENREF_47)^ | p.o. | 40 | HV Japanese | m/f | 6 | omepral | PM +/- fluvoxamine | Model qualification (Figure S3.4)  DDI predictions with fluvoxamine (Figure S3.4) |
| Study c02327040^[48](#_ENREF_48)^ | p.o. | 40 q.d. | HV Caucasian and Japanese | m/f | 10 | gastro-resistant hard capsule | - | Model qualification (Figure S2.12) |
| Study c01959611^[49](#_ENREF_49)^ | p.o. | 40 q.d. | HV Caucasian | m/f | 15 | gastro-resistant hard capsule | - | Model qualification (Figure S2.12) |

*single dose unless otherwise specified; - respective information was not provided in the literature source.

Items in bold were used as part of the training set to refine the model.

# **Section S1.3. S-Mephenytoin**

## **Table S1.3.1: Physicochemical properties of S-mephenytoin**

| Parameter | Value | Source | Comment |
| --- | --- | --- | --- |
| MW | 218.2 | Website drugbank.ca/drugs/DB00532 |  |
| BCS class | I |  | Assumption that compound is Caco-2 permeable; prediction by DrugBank (drugbank.ca/drugs/DB00532); solubility in water is high |
| Aqueous solubility at pH 7 | 1.27 mg/mL | Website drugbank.ca/drugs/DB00532 |  |
| LogP | 1.69 | Website drugbank.ca/drugs/DB00532 | Experimental properties[^50^](#_ENREF_50) |
| fu | 0.70 | Steere 2015[^51^](#_ENREF_51) |  |
| pKa | 8.51 | Website drugbank.ca/drugs/DB00532 | Experimental properties^[52](#_ENREF_52)^ |

## **Table S1.3.2: S-Mephenytoin clinical data used in model development and evaluation**

| **Source** | **Route** | **Dose / Schedule** | **Population** | **Age [yrs] (mean) /range** | **Weight [kg] (mean) /range** | **Sex** | **N** | **Formulation** | **Comment** | **Model development step # and purpose** |
| --- | --- | --- | --- | --- | --- | --- | --- | --- | --- | --- |
| Adedoyin 1998[^53^](#_ENREF_53) | p.o. | 100 mg s.d. | HV, all EM | 54.7 / 32-73 | - | m/f | 8 | tablet | RS-mephenytoin used, | Model qualification |
| Jacqz 1986[^54^](#_ENREF_54) | p.o. | 100 mg s.d. | HV, 6 EM, 1 IM and 1 PM | 25-76 | - | m/f | 8 | tablet | RS-mephenytoin used | Model qualification |
| Yao 2003[^5^](#_ENREF_5) | p.o. | 100 mg s.d. | HV | 23-49 | - | m/f | 12 | - | S-mephenytoin, with and without Fluvoxamine m.d. of 37.5, 62.5 and 87.5 mg/day | Model qualification (Figure S3.6) |
| Iga 2016[^3^](#_ENREF_3) | p.o. | 100 mg s.d. | - | - | - | - | - | - |  | Model qualification |
| Wedlund 1985[^55^](#_ENREF_55) | p.o. | 300 mg s.d. | HV | 21-76 | 54-108 | M | 8 | tablet | S-mephenytoin | Model qualification |

- respective information was not provided in the literature source

# **Section S1.4. Moclobemide**

## **Table S1.4.1: Physicochemical properties of moclobemide**

| Parameter | | Value | Source | Comment |
| --- | --- | --- | --- | --- |
| MW | 268.7 g/mol | | Website drugbank.ca/drugs/DB01171 |  |
| BCS class | II | |  | Caco-2 permeability is high[^56^](#_ENREF_56); insoluble in water |
| Solubility in intestinal fluid pH 6.8 | 3 mg/mL | | Roche in-house data [^57^](#_ENREF_57) | Measured |
| LogD | 1.79 | | Pons 1990[^58^](#_ENREF_58) | Similar values in other sources (e.g. ALOGPS: 1.56, Website drugbank.ca/drugs/DB01171) |
| fu | 0.50 | | MHRA Label Moclobemide[^59^](#_ENREF_59) |  |
| pKa | 6.2 | | IPCS ICHEM  Website: inchem.org/documents/pims/pharm/pim151.htm#SectionTitle:3.3%20%20Physical%20properties | Weak base |
| Km_FMO (microsomes) | 0.77 mmol/L | | Hoskins 2001[^60^](#_ENREF_60) |  |
| Vmax_FMO (microsomes) | 1.39 nmol/min/mg prot. | | Hoskins 2001[^60^](#_ENREF_60) |  |
| Renal elimination | 0.03 mL/min/kg | | Derived from Schoerlin 1987[^61^](#_ENREF_61) | Schoerlin reports 2.6 mL/min/76kg |
| Ki_CYP2C19 (free) | 203.8 umol/l | | Kramer-Nielsen 1996[^62^](#_ENREF_62) | The Ki value reported for total concentrations was 210 umol/L and corrected with an fu_mic of 0.97 |

## **Table S1.4.2: Moclobemide literature Ki values for CYP2C19 inhibition**

| **Substrate** | **Source** | **in vivo Ki [µM]** | **Literature reference** |
| --- | --- | --- | --- |
| clomipramine | in vitro HLM calculated to unbound | 203.82 | Kramer Nielsen, 1996^[62](#_ENREF_62)^ |

## **Table S1.4.3: Moclobemide clinical data used in model development and evaluation**

| **Source** | **Route** | **Dose [mg] /Schedule*** | **Population** | **Age [yrs] (mean or range)** | **Weight [kg] (mean or range)** | **Sex** | **N** | **Formulation** | **Comment** | **Model development step # and purpose** |
| --- | --- | --- | --- | --- | --- | --- | --- | --- | --- | --- |
| **Gram 1995**^[63](#_ENREF_63)^ | p.o | 300 s.d. / b.i.d. | HV | 26 | - | m/f | 8 | tablet | EM + PM | **2. Estimate non-CYP2C19 metabolism**  **3. Estimate CYP2C19 metabolism**  **4. Multiple dose predictions with autoinhibition**  5. Model evaluation |
| **Yu 2001**^[64](#_ENREF_64)^ | p.o. | 300 s.d. | HV-Asian | - | 60.3 | m | 8 | tablet | EM, PM and EM+OMP40 | **2. Estimate non-CYP2C19 metabolism**  **3. Estimate CYP2C19 metabolism**  6. DDI predictions |
| **Wiesel 1985**^[65](#_ENREF_65)^ | p.o. | 50, 100, 200 s.d. | HV or patient | 26.3 | 75.8 | m | 6 | tablet |  | **3. Estimate CYP2C19 metabolism**  5. Model Evaluation |
| Ignjatovic 2009^[66](#_ENREF_66)^ | p.o. | 150 t.i.d. | Patient | - | - | m/f | 6 | tablet |  | 4. Multiple dose predictions with autoinhibition |
| **Schoerlin 1987**^[61](#_ENREF_61)^ | p.o. & i.v. infusion | 150 t.i.d. /s.d. | HV | 27 | 76 | m | 12 | tablet/ solution |  | **1. Selection of distribution model**  **3. Estimate CYP2C19 metabolism**  **4. Multiple dose predictions with autoinhibition** |
| **Guentert 1990**^[67](#_ENREF_67)^ | p.o. | 150 t.i.d. | HV | 19-29 | 59-86 | m/f | 14 | tablet |  | **3. Estimate CYP2C19 metabolism** |
| **Raaflaub 1984**^[68](#_ENREF_68)^ | p.o. & i.v. infusion | 50 s.d. | HV | 42 | 74 | m | 6 | tablet/ solution |  | **1. Selection of distribution model**  **3. Estimate CYP2C19 metabolism**  5. Model Evaluation |

*single dose unless otherwise specified; - indicates respective information was not provided in the literature source

Items in bold were used as part of the training set to refine the model.

# **Section S1.5. Tizanidine**

## **Table S1.5.1: Physicochemical properties of tizanidine**

| Parameter | Value | Source | Comment |
| --- | --- | --- | --- |
| MW free base:  Tizanidine-HCl: | 253.7 g/mol  289.7 g/mol | Website drugbank.ca/drugs/DB00697  Website drugbank.ca/drugs/DBSALT000550 | Dose of commercial product usually refers to amount of tizanidine free base – no conversion is necessary |
| BCS Class | II | Bhakay 2018[^69^](#_ENREF_69) |  |
| Aqueous Solubility at pH 7.4 | 0.133 mg/mL | Website drugbank.ca/drugs/DB00697 | Calculated by ALOGPS |
| LogP | 1.4 | Website drugbank.ca/drugs/DB00697 |  |
| fu | 0.7 | SmPC tizanidine[^70^](#_ENREF_70) |  |
| pKa (Base) | 7.49 | Website drugbank.ca/drugs/DB00697 |  |
| Intrinsic CL | 17 mL/min/kg | Granfors 2004[^71^](#_ENREF_71) | Predicted from microsomal assay |

## **Table S1.5.2: Tizanidine clinical data used in model development and evaluation**

| **Source** | **Dose [mg] / Schedule*** | **Popu-lation** | **Age [yrs] (mean or range)** | **Weight [kg] (mean or range)** | **Sex** | **N** | **Form.** | **Fasted**  **or Fed** | **Comment** | **Model development step # and Purpose** |
| --- | --- | --- | --- | --- | --- | --- | --- | --- | --- | --- |
| **Momo 2010**^[72](#_ENREF_72)^ | 2 | HV | 29 | 70 | m | 12 | Tablet | Fed | With/ without mexiletine | **2. Food and formulation effect** |
| **Granfors 2004**^[73](#_ENREF_73)^ | 4 | HV | 21-31 | 65-83 | m | 10 | Tablet | Fasted | With / without fluvoxamine | **1. Fitting the CYP1A2 elimination and absorption**  **4. DDI prediction** |
| Schellenberger  1999^[74](#_ENREF_74)^ | 4 t.i.d. | HV | 19-37 | 70-97 | m | 12 | Tablet | Fasted |  | 3. Model evaluation |
| **Henney 2007**^[75](#_ENREF_75)^ | 4 | HV | 26 | 71 | m12/f6 | 18 | Tablet / Capsule | Fed | fed | **2. Food and formulation effect** |
| **Backman 2008**^[76](#_ENREF_76)^ | 4 | HV | 23 | 78 | m | 71 | Tablet | Fasted | Male and female non-smokers and male smokers | **1. Fitting the CYP1A2 elimination and absorption** |
| **Backman 2006**^[77](#_ENREF_77)^ | 4 | HV | 21 | 71 | m6/f4 | 10 | Tablet | Fasted | Only control group used | **1. Fitting the CYP1A2 elimination and absorption** |
| **Shah 2006**^[78](#_ENREF_78)^ | 8 | HV | 18-52 | 46-102 | m54/f42 | 96 | Tablet / Capsule | Fed/  Fasted |  | **2. Food and formulation effect** |
| Tse 1987^[79](#_ENREF_79)^ | 4 t.i.d. | HV | 21-48 | 57-86 | m | 6 | Tablet | Fasted |  | 3. Model evaluation |
| **Al-Ghazawi 2013**^[80](#_ENREF_80)^ | 4 | HV | 28 | 75 | m | 36 | Tablet | Fasted |  | **1. Fitting the CYP1A2 elimination and absorption** |

*single dose unless otherwise specified; - indicates respective information was not provided in the literature source

Items in bold were used as part of the training set to refine the model.

# **Section S1.6. Mexiletine**

## **Table S1.6.1: Physicochemical properties of mexiletine**

| Parameter | Value | Source | Comment |
| --- | --- | --- | --- |
| MW | 179.3 g/mol | Website drugbank.ca/drugs/DB00379 |  |
| BCS class | I | ddfint.net/search.cfm (accessed 03 June 2019) |  |
| Water solubility | 0.54 mg/mL | Website drugbank.ca/drugs/DB00379 | ALOGPS |
| LogD | 2.15-2.46 | Website drugbank.ca/drugs/DB00379 |  |
| fu | 0.50-0.40 | Website drugbank.ca/drugs/DB00379 |  |
| pKa | Base/9.2 | Website drugbank.ca/drugs/DB00379 | Strong base |
| CYP1A2 CL | 0.5-11 L/h | Labbé 2000[^81^](#_ENREF_81) | Partial metabolic clearance of mexiletine to N-hydroxymexiletine. |
| CYP2D6 CL | 12-13 L/h | Labbé 2000[^81^](#_ENREF_81) | Difference in non-renal CL between CYP2D6 extensive and poor metabolizers |
| Unspecific liver CL | 12-24 L/h | Labbé 2000[^81^](#_ENREF_81) | Non-renal CL – CYP2D6 CL – CYP1A2 CL |
| Renal elimination | 1.8-2.1 L/h | Labbé 2000[^81^](#_ENREF_81) |  |
| Ki_CYP1A2 | 0.28 umol/l | Wei 1991[^82^](#_ENREF_82) |  |

## **Table S1.6.2: Mexiletine literature Ki values for CYP1A2 inhibition**

| **Substrate** | **Source** | **in vivo Ki [µM]** | **Literature reference** |
| --- | --- | --- | --- |
| Methoxyresofurin | in vitro HLM calculated to unbound | **0.28** | Wei, 1999^[82](#_ENREF_82)^ |

Items in bold were used as part of the final model.

## **Table S1.6.3: Mexiletine clinical data used in model development and evaluation**

| **Source** | **Route** | **Dose (mg) / Schedule*** | **Pop.** | **Sex** | **N** | **Form.** | **Comment** | **Model development step # and purpose** |
| --- | --- | --- | --- | --- | --- | --- | --- | --- |
| **Campbell 1978**^[83](#_ENREF_83)^ | i.v. | 200 | HV | m | 5 | solution |  | **1. Define distribution and metabolism based on i.v.** |
| **Campbell 1978**^[83](#_ENREF_83)^ | p.o. | 200 | HV | m | 5 | - |  | **2. Define mexiletine absorption based on p.o.** |
| **Campbell 1978**^[83](#_ENREF_83)^ | i.v. | 200 | patients | - | 10 | solution |  | **1. Define distribution and metabolism based on i.v.** |
| **Begg 1982**^[84](#_ENREF_84)^ | p.o. | 333.24 | HV | m/f | 6 | tablet |  | **2. Define mexiletine absorption based on p.o.** |
| Labbé 2000[^81^](#_ENREF_81) | p.o. | 83.31 b.i.d. | HV | m/f | 1 | - | EM/PM | Model qualification (Figure S2.29) |
| **Pringle 1986**^[85](#_ENREF_85)^ | p.o. | 83.31 - 166.62 - 249.9 - 333.24 - 499.9 | HV | m | 12 | capsule | -- | **2. Define mexiletine absorption based on p.o.** Model qualification (Figure S2.30) |
| **Kusumoto 1998**^[86](#_ENREF_86)^ | p.o. | 166.62 | HV | m | 9 | capsule | -- | **2. Define mexiletine absorption based on p.o.** |
| Kusumoto 2001^[87](#_ENREF_87)^ | p.o. | 166.62 | HV japanese | m | 6 | - | +/- Fluvoxamine | Model qualification (Figure S3.14) |
| **Pentikäinen 1984**^[88](#_ENREF_88)^ | i.v. | 166.62 | acute myocardial infarction | -- | 18 | solution | acute myocardial infarction | **1. Define distribution and metabolism based on i.v.** |
| **Joeres 1987**^[89](#_ENREF_89)^ | p.o. | Mexiletine: 200  Caffeine: 366 | HV | -- | 1 | -- | Caffeine +/- Mexiletine | **2. Define mexiletine absorption based on p.o.** Model qualification (Figure S3.12) |
| Momo 2010^[72](#_ENREF_72)^ | p.o. | Mexiletine: 50 b.i.d.  Tizanidine: 2 | HV | m | 12 | tablet |  | Model qualification (Figure S3.13) |

*single dose unless otherwise specified; - indicates respective information was not provided in the literature source

Items in bold were used for model development.

# **Section S1.7. Ethinylestradiol**

## **Table S1.7.1: Physicochemical properties of ethinylestradiol**

| Parameter | Value | Source | Comment |
| --- | --- | --- | --- |
| MW | 296.4 g/mol | Website drugbank.ca/drugs/DB00977 | -- |
| BCS class | I | ddfint.org/search.cfm (accessed 03 June 2019) |  |
| Water solubility | 6.77E-3 mg/mL | Website drugbank.ca/drugs/DB00977 | -- |
| LogD | 3.63 – 3.9 | Website drugbank.ca/drugs/DB00977 | -- |
| fu | 0.03 | Website drugbank.ca/drugs/DB00977 | -- |
| pKa | Acid/10.33 | Website drugbank.ca/drugs/DB00977 | -- |
| CYP1A2 CL | 0.51 µL/min/pmol | Ezuruike 2018[^90^](#_ENREF_90) | -- |
| CYP2C8 CL | 0.13 µL/min/pmol | Ezuruike 2018[^90^](#_ENREF_90) | -- |
| CYP2C9 CL | 0.51 µL/min/pmol | Ezuruike 2018[^90^](#_ENREF_90) | -- |
| CYP3A4 CL | 0.5 µL/min/pmol | Ezuruike 2018[^90^](#_ENREF_90) | -- |
| Km_UGT1A1 | 19.22 µmol/L | Ezuruike 2018[^90^](#_ENREF_90) | -- |
| Vmax_ UGT1A1 | 408.5 pmol/min/mg prot. | Ezuruike 2018[^90^](#_ENREF_90) | -- |
| Renal Elimination | 2.079 L/h | Stanczyk 2013[^91^](#_ENREF_91) | -- |
| Ki_CYP1A2 | 10.6 µmol/L | Karjalainen 2008[^11^](#_ENREF_11) | -- |

## **Table S1.7.2: Ethinylestradiol literature Ki values for CYP1A2 inhibition**

| **Substrate** | **Source** | **in vivo Ki [µM]** | **Literature reference** |
| --- | --- | --- | --- |
| phenacetin | in vitro HLM calculated to unbound | 10.6 | Karjalainen, 2008^[11](#_ENREF_11)^ |

## **Table S1.7.3: Ethinylestradiol clinical data used in model development and evaluation**

| **Source** | **Route** | **Dose (mg) / Schedule*** | **Population** | **Sex** | **N** | **Form.** | **Comment** | **Model development step # and Purpose** |
| --- | --- | --- | --- | --- | --- | --- | --- | --- |
| **Back 1981**^[92](#_ENREF_92)^ | i.v. | 0.03 | HV | f | 5 | Solution | Mean | **1. Define distribution and metabolism based on i.v. and p.o.** |
| **Back 1981**^[92](#_ENREF_92)^ | p.o. | 0.03 | HV | f | 5 | Tablet | Mean | **1. Define distribution and metabolism based on i.v. and p.o.** |
| **Back 1979**^[93](#_ENREF_93)^ | i.v. | 0.05 | HV | f | 6 | Solution | Mean | **1. Define distribution and metabolism based on i.v. and p.o.** |
| **Back 1979**^[93](#_ENREF_93)^ | p.o. | 0.05 | HV | f | 6 | NA | Mean | **1. Define distribution and metabolism based on i.v. and p.o.** |
| **Back 1987**[^94^](#_ENREF_94) | i.v. | 0.05 q.d. | HV | f | 5 | Solution | Mean |  |
| Back 1987^[94](#_ENREF_94)^ | p.o. | 0.05 q.d. | HV | f | 5 | Tablet | Mean | Model qualification |
| **Orme 1991**^[95](#_ENREF_95)^ | i.v. | 0.03 | HV | f | 10 | Solution | Mean | **1. Define distribution and metabolism based on i.v. and p.o.** |
| **Orme 1991**^[95](#_ENREF_95)^ | p.o. | 0.03 | HV | f | 10 | Tablet | Mean | **1. Define distribution and metabolism based on i.v. and p.o.** |
| **Kuhnz 1996**^[96](#_ENREF_96)^ | i.v. | 0.06 | HV | f | 19 | Solution | Mean | **1. Define distribution and metabolism based on i.v. and p.o.** |
| **Goebelsmann 1986**^[97](#_ENREF_97)^ | p.o. | 0.03 | HV | f | 24 | Solution and tablet | Mean | **1. Define distribution and metabolism based on i.v. and p.o.** |
| **Stanczyk 1983**^[98](#_ENREF_98)^ | p.o. | 0.12 | HV | f | 24 | Solution and tablet | Mean | **1. Define distribution and metabolism based on i.v. and p.o.** |
| **Zhang 2017**^[99](#_ENREF_99)^ | p.o. | 0.03 | HV | f | 12 | Tablet | Mean | **1. Define distribution and metabolism based on i.v. and p.o.** |
| Martin 2016^[100](#_ENREF_100)^ | p.o. | 0.03 q.d. | HV | f | 27 | Tablet | Mean | Model qualification |
| Stockis 2014^[101](#_ENREF_101)^ | p.o. | 0.03 q.d. | HV | f | 24 | Tablet | Mean | Model qualification |
| Sidhu 2006^[102](#_ENREF_102)^ | p.o. | 0.03 q.d. | HV | f | 16 | Tablet | Mean | Model qualification |
| **Kothare 2012**^[103](#_ENREF_103)^ | p.o. | 0.03/0.03 q.d. | HV | f | 20 | Tablet | Mean | **1. Define distribution and metabolism based on i.v. and p.o.** |
| **Timmer 2000**^[104](#_ENREF_104)^ | p.o. | 0.03 | HV | f | - | Tablet | Mean | **1. Define distribution and metabolism based on i.v. and p.o.** |
| Balogh 1995^[105](#_ENREF_105)^ | p.o. | EE: 0.03 q.d.  Caffeine: 200 | HV | - | 20 | Tablet | Caffeine +/- EE | Model qualification |
| **Granfors 2005**^[106](#_ENREF_106)^ | p.o. | EE: 0.02 – 0.03 q.d.  Tizanidine: 4 | HV | - | 15 | Tablet | Tizanidine +/- EE | **2. Estimate TDI on CYP1A2** |
| Study c13608215-02^[107](#_ENREF_107)^ | p/o. | 0.03 q.d. | HV | f | 16 | Tablet | Individual data | Model qualification |

*single dose unless otherwise specified; - indicates respective information was not provided in the literature source

Items in bold were used for model development.

;

# **Section S1.8. Glossary of abbreviations used in this document**

| ADMET | Absorption, Distribution, Metabolism, Excretion and Toxicity |
| --- | --- |
| ALOGPs | Atomic LogP |
| b.i.d. | Twice daily (bis in diem) |
| BCS | Biopharmaceutics Classification System |
| CL | Clearance |
| CR | Controlled release |
| CYP | Cytochrome P450 oxidase |
| CYP1A2 | Cytochrome P450 1A2 oxidase |
| CYP2C19 | Cytochrome P450 2C19 oxidase |
| CYP2D6 | Cytochrome P450 2D6 oxidase |
| CYP3A4 | Cytochrome P450 3A4 oxidase |
| DDI | Drug-drug interaction |
| e.c. | Enteric coated |
| EE | Ethinylestradiol |
| EM | Extensive metabolizers |
| FDA | Food and Drug Administration |
| FMO | Flavin-containing monooxygenase |
| fu | Fraction unbound |
| FDA | Food and Drug administration |
| HLM | Human liver microsomes |
| hm | Homozygous |
| HV | Healthy volunteers |
| IDs | Individuals |
| IM | Intermediate metabolizers |
| i.v. | Intravenous |
| Ka | Absorption rate constant |
| Ki | Inhibitor constant |
| Kinact | Rate of enzyme inactivation |
| Km | Michaelis–Menten constant |
| logD | Water:octanol partition coefficient for the ionized and un-ionized form of the compound at a specific pH |
| logP | Water:octanol partition coefficient for the un-ionized form of the compound |
| m.d. | Multiple dose |
| m/f | Male/female |
| MW | Molecular weight |
| NA | Not applicable/not available |
| PK | Pharmacokinetics |
| pKa | Negative logarithm of acid dissociation constant |
| PM | Poor metabolizers |
| p.o. | Orally (per os) |
| q.d. | Once daily (quaque diem) |
| s.d. | Single dose |
| TDI | Time dependent inhibition |
| t.i.d. | Three times a day (ter in die) |
| UGT | Uridine 5'-diphospho-glucuronosyltransferase |
| Vmax | Maximum enzymatic rate achieved by the system at saturating substrate concentration |

# **Section S1.9. REFERENCES**

1. Alqahtani S., Kaddoumi A. Development of a Physiologically Based Pharmacokinetic/Pharmacodynamic Model to Predict the Impact of Genetic Polymorphisms on the Pharmacokinetics and Pharmacodynamics Represented by Receptor/Transporter Occupancy of Central Nervous System Drugs. *Clin Pharmacokinet* **55** 957-969. (2016)

2. Perucca E., Gatti G., Spina E. Clinical pharmacokinetics of fluvoxamine. *Clin Pharmacokinet* **27** 175-190. (1994)

3. Iga K. Dynamic and Static Simulations of Fluvoxamine-Perpetrated Drug-Drug Interactions Using Multiple Cytochrome P450 Inhibition Modeling, and Determination of Perpetrator-Specific CYP Isoform Inhibition Constants and Fractional CYP Isoform Contributions to Victim Clearance. *J Pharm Sci* **105** 1307-1317. (2016)

4. Foti R.S., Wahlstrom J.L. CYP2C19 inhibition: the impact of substrate probe selection on in vitro inhibition profiles. *Drug Metab Dispos* **36** 523-528. (2008)

5. Yao C., Kunze K.L., Trager W.F., Kharasch E.D., Levy R.H. Comparison of in vitro and in vivo inhibition potencies of fluvoxamine toward CYP2C19. *Drug Metab Dispos* **31** 565-571. (2003)

6. Obach R.S., Walsky R.L., Venkatakrishnan K. Mechanism-based inactivation of human cytochrome p450 enzymes and the prediction of drug-drug interactions. *Drug Metab Dispos* **35** 246-255. (2007)

7. McGinnity D.F., Waters N.J., Tucker J., Riley R.J. Integrated in vitro analysis for the in vivo prediction of cytochrome P450-mediated drug-drug interactions. *Drug Metab Dispos* **36** 1126-1134. (2008)

8. Rasmussen B.B., Nielsen T.L., Brosen K. Fluvoxamine is a potent inhibitor of the metabolism of caffeine in vitro. *Pharmacol Toxicol* **83** 240-245. (1998)

9. von Moltke L.L.*, et al.* Phenacetin O-deethylation by human liver microsomes in vitro: inhibition by chemical probes, SSRI antidepressants, nefazodone and venlafaxine. *Psychopharmacology (Berl)* **128** 398-407. (1996)

10. Brosen K., Skjelbo E., Flachs H. Proguanil metabolism is determined by the mephenytoin oxidation polymorphism in Vietnamese living in Denmark. *Br J Clin Pharmacol* **36** 105-108. (1993)

11. Karjalainen M. Inhibition of CYP1A2-mediated drug metabolism in vitro and in humans: With special emphasis on rofecoxib and other NSAIDs. University of Helsinki, Finland, 2008.

12. Foti R.S., Rock D.A., Wienkers L.C., Wahlstrom J.L. Selection of alternative CYP3A4 probe substrates for clinical drug interaction studies using in vitro data and in vivo simulation. *Drug Metab Dispos* **38** 981-987. (2010)

13. Culm-Merdek K.E., von Moltke L.L., Harmatz J.S., Greenblatt D.J. Fluvoxamine impairs single-dose caffeine clearance without altering caffeine pharmacodynamics. *Br J Clin Pharmacol* **60** 486-493. (2005)

14. Carrillo J.A.*, et al.* Disposition of fluvoxamine in humans is determined by the polymorphic CYP2D6 and also by the CYP1A2 activity. *Clin Pharmacol Ther* **60** 183-190. (1996)

15. Spigset O., Granberg K., Hagg S., Norstrom A., Dahlqvist R. Relationship between fluvoxamine pharmacokinetics and CYP2D6/CYP2C19 phenotype polymorphisms. *Eur J Clin Pharmacol* **52** 129-133. (1997)

16. de Vries M.H., Raghoebar M., Mathlener I.S., van Harten J. Single and multiple oral dose fluvoxamine kinetics in young and elderly subjects. *Ther Drug Monit* **14** 493-498. (1992)

17. Iga K. Use of three-compartment physiologically based pharmacokinetic modeling to predict hepatic blood levels of fluvoxamine relevant for drug-drug interactions. *J Pharm Sci* **104** 1478-1491. (2015)

18. Orlando R., De Martin S., Andrighetto L., Floreani M., Palatini P. Fluvoxamine pharmacokinetics in healthy elderly subjects and elderly patients with chronic heart failure. *Br J Clin Pharmacol* **69** 279-286. (2010)

19. Spigset O., Granberg K., Hagg S., Soderstrom E., Dahlqvist R. Non-linear fluvoxamine disposition. *Br J Clin Pharmacol* **45** 257-263. (1998)

20. De Vries M.H., Van Harten J., Van Bemmel P., Raghoebar M. Pharmacokinetics of fluvoxamine maleate after increasing single oral doses in healthy subjects. *Biopharm Drug Dispos* **14** 291-296. (1993)

21. Van Harten J., Kok F.A., Lönnebo A., Grahnén A. Pharmacokinetics of fluvoxamine after intravenous and oral administration. Poster P-1-58. 1994. p. 331.

22. Van Harten J., Van Bemmel P., Dobrinska M.R., Ferguson R.K., Raghoebar M. Bioavailability of fluvoxamine given with and without food. *Biopharm Drug Dispos* **12** 571-576. (1991)

23. Kunii T.*, et al.* Interaction study between enoxacin and fluvoxamine. *Ther Drug Monit* **27** 349-353. (2005)

24. Fukasawa T.*, et al.* Effects of caffeine on the kinetics of fluvoxamine and its major metabolite in plasma after a single oral dose of the drug. *Ther Drug Monit* **28** 308-311. (2006)

25. Fleishaker J.C., Hulst L.K. A pharmacokinetic and pharmacodynamic evaluation of the combined administration of alprazolam and fluvoxamine. *Eur J Clin Pharmacol* **46** 35-39. (1994)

26. Labellarte M.*, et al.* Multiple-dose pharmacokinetics of fluvoxamine in children and adolescents. *J Am Acad Child Adolesc Psychiatry* **43** 1497-1505. (2004)

27. U.S. Food and Drug Administration. FDA review Luvox. 2008.

28. Jeppesen U., Loft S., Poulsen H.E., Brsen K. A fluvoxamine-caffeine interaction study. *Pharmacogenetics* **6** 213-222. (1996)

29. Boehringer Ingelheim. Study c13128239-01: Influence of fluvoxamine on the pharmacokinetics of BI 409306 after oral administration (randomized, open-label, two-treatment, two-sequence, two-period crossover study).

30. Zhou J.*, et al.* Effect of esomeprazole, a proton pump inhibitor on the pharmacokinetics of sonidegib in healthy volunteers. *Br J Clin Pharmacol* **82** 1022-1029. (2016)

31. Ogilvie B.W.*, et al.* The proton pump inhibitor, omeprazole, but not lansoprazole or pantoprazole, is a metabolism-dependent inhibitor of CYP2C19: implications for coadministration with clopidogrel. *Drug Metab Dispos* **39** 2020-2033. (2011)

32. U.S. Food and Drug Administration. Nexium prescribing information. 2014 [cited 26 November 2019]Available from: <https://www.accessdata.fda.gov/drugsatfda_docs/label/2014/022101s014021957s017021153s050lbl.pdf>

33. Wu F.*, et al.* Predicting nonlinear pharmacokinetics of omeprazole enantiomers and racemic drug using physiologically based pharmacokinetic modeling and simulation: application to predict drug/genetic interactions. *Pharm Res* **31** 1919-1929. (2014)

34. Liu K.H.*, et al.* Stereoselective inhibition of cytochrome P450 forms by lansoprazole and omeprazole in vitro. *Xenobiotica* **35** 27-38. (2005)

35. Furuta T.*, et al.* Effects of genotypic differences in CYP2C19 status on cure rates for Helicobacter pylori infection by dual therapy with rabeprazole plus amoxicillin. *Pharmacogenetics* **11** 341-348. (2001)

36. Ko J.W., Sukhova N., Thacker D., Chen P., Flockhart D.A. Evaluation of omeprazole and lansoprazole as inhibitors of cytochrome P450 isoforms. *Drug Metab Dispos* **25** 853-862. (1997)

37. Li X.Q., Weidolf L., Simonsson R., Andersson T.B. Enantiomer/enantiomer interactions between the S- and R- isomers of omeprazole in human cytochrome P450 enzymes: major role of CYP2C19 and CYP3A4. *J Pharmacol Exp Ther* **315** 777-787. (2005)

38. Andersson T., Regårdh C.G. Pharmacokinetics of Omeprazole and Metabolites Following Single Intravenous and Oral Doses of 40 and 80mg. *Drug Investig* **2** 255-263. (1990)

39. Andersson T., Regårdh C.G. The Pharmacokinetics of Single and Repeated Once-Daily Doses of 10, 20 and 40mg Omeprazole as Enteric-Coated Granules. *Drug Investig* **3** 45-52. (1991)

40. Andersson T., Holmberg J., Rohss K., Walan A. Pharmacokinetics and effect on caffeine metabolism of the proton pump inhibitors, omeprazole, lansoprazole, and pantoprazole. *Br J Clin Pharmacol* **45** 369-375. (1998)

41. Oosterhuis B., Jonkman J.H., Andersson T., Zuiderwijk P.B. No influence of single intravenous doses of omeprazole on theophylline elimination kinetics. *J Clin Pharmacol* **32** 470-475. (1992)

42. Uno T.*, et al.* Absolute bioavailability and metabolism of omeprazole in relation to CYP2C19 genotypes following single intravenous and oral administrations. *Eur J Clin Pharmacol* **63** 143-149. (2007)

43. Regardh C.G., Andersson T., Lagerstrom P.O., Lundborg P., Skanberg I. The pharmacokinetics of omeprazole in humans--a study of single intravenous and oral doses. *Ther Drug Monit* **12** 163-172. (1990)

44. Andersson T., Rohss K., Hassan-alin M. Pharmacokinetics (PK) and effect on pentagastrin stimulated peak acid output (PAO) of omeprazole (O) and its 2 optical isomers, S-omeprazole/esomeprazole (E) and R-omeprazole (R-O). **118** A1210. (1990)

45. Hassan-Alin M., Andersson T., Niazi M., Rohss K. A pharmacokinetic study comparing single and repeated oral doses of 20 mg and 40 mg omeprazole and its two optical isomers, S-omeprazole (esomeprazole) and R-omeprazole, in healthy subjects. *Eur J Clin Pharmacol* **60** 779-784. (2005)

46. Cho J.Y.*, et al.* Omeprazole hydroxylation is inhibited by a single dose of moclobemide in homozygotic EM genotype for CYP2C19. *Br J Clin Pharmacol* **53** 393-397. (2002)

47. Yasui-Furukori N.*, et al.* Different inhibitory effect of fluvoxamine on omeprazole metabolism between CYP2C19 genotypes. *Br J Clin Pharmacol* **57** 487-494. (2004)

48. Boehringer Ingelheim. Study c02327040: Investigation of the effect of food and of increased gastric pH on the relative bioavailability of deleobuvir following single oral administration in healthy Caucasian and Japanese subjects (an open label, randomised, four-way crossover study).

49. Boehringer Ingelheim. Study c01959611: Investigation of the effect of food and of increased gastric pH on the relative bioavailability of a single oral dose of 240 mg faldaprevir in an open-label, randomised, three-way crossover trial in healthy subjects).

50. Sangster J. A databank of evaluated octanol-water partition coefficients (Log P) on microcomputer diskette: Sangster Res Lab.; 1994.

51. Steere B., Baker J.A., Hall S.D., Guo Y. Prediction of in vivo clearance and associated variability of CYP2C19 substrates by genotypes in populations utilizing a pharmacogenetics-based mechanistic model. *Drug Metab Dispos* **43** 870-883. (2015)

52. Tomlinson E., Hafkenscheid T.l. Aqueous solubility and partitition coefficient estimation from HPLC data. In: Dunn III WJ, Block JH, Plearlman RS (eds). *Partition coefficient, determination and estimation*. Pergamon Press: New York, 1986, pp 101-141.

53. Adedoyin A., Arns P.A., Richards W.O., Wilkinson G.R., Branch R.A. Selective effect of liver disease on the activities of specific metabolizing enzymes: investigation of cytochromes P450 2C19 and 2D6. *Clin Pharmacol Ther* **64** 8-17. (1998)

54. Jacqz E., Hall S.D., Branch R.A., Wilkinson G.R. Polymorphic metabolism of mephenytoin in man: pharmacokinetic interaction with a co-regulated substrate, mephobarbital. *Clin Pharmacol Ther* **39** 646-653. (1986)

55. Wedlund P.J.*, et al.* Phenotypic differences in mephenytoin pharmacokinetics in normal subjects. *J Pharmacol Exp Ther* **234** 662-669. (1985)

56. Reddy V.P.*, et al.* An Investigation into the Prediction of the Plasma Concentration-Time Profile and Its Interindividual Variability for a Range of Flavin-Containing Monooxygenase Substrates Using a Physiologically Based Pharmacokinetic Modeling Approach. *Drug Metab Dispos* **46** 1259-1267. (2018)

57. INCHEM. Moclobemide. [cited 26 November 2019]Available from: www.inchem.org/documents/pims/pharm/pim151.htm#PartTitle:3.%20%20PHYSICO-CHEMICAL%20PROPERTIES

58. Pons G.*, et al.* Moclobemide excretion in human breast milk. *Br J Clin Pharmacol* **29** 27-31. (1990)

59. MHRA label. Moclobemide film-coated tablets. [cited 26 November 2019]Available from: <http://www.mhra.gov.uk/home/groups/par/documents/websiteresources/con097060.pdf>

60. Hoskins J., Shenfield G., Murray M., Gross A. Characterization of moclobemide N-oxidation in human liver microsomes. *Xenobiotica* **31** 387-397. (2001)

61. Schoerlin M.P., Mayersohn M., Korn A., Eggers H. Disposition kinetics of moclobemide, a monoamine oxidase-A enzyme inhibitor: single and multiple dosing in normal subjects. *Clin Pharmacol Ther* **42** 395-404. (1987)

62. Nielsen K.K., Flinois J.P., Beaune P., Brosen K. The biotransformation of clomipramine in vitro, identification of the cytochrome P450s responsible for the separate metabolic pathways. *J Pharmacol Exp Ther* **277** 1659-1664. (1996)

63. Gram L.F., Guentert T.W., Grange S., Vistisen K., Brosen K. Moclobemide, a substrate of CYP2C19 and an inhibitor of CYP2C19, CYP2D6, and CYP1A2: a panel study. *Clin Pharmacol Ther* **57** 670-677. (1995)

64. Yu K.S.*, et al.* Effect of omeprazole on the pharmacokinetics of moclobemide according to the genetic polymorphism of CYP2C19. *Clin Pharmacol Ther* **69** 266-273. (2001)

65. Wiesel F.A., Raaflaub J., Kettler R. Pharmacokinetics of oral moclobemide in healthy human subjects and effects on MAO-activity in platelets and excretion of urine monoamine metabolites. *Eur J Clin Pharmacol* **28** 89-95. (1985)

66. Rakic Ignjatovic A., Miljkovic B., Todorovic D., Timotijevic I., Pokrajac M. Moclobemide monotherapy vs. combined therapy with valproic acid or carbamazepine in depressive patients: a pharmacokinetic interaction study. *Br J Clin Pharmacol* **67** 199-208. (2009)

67. Guentert T.W.*, et al.* Pharmacokinetics of moclobemide after single and multiple oral dosing with 150 milligrams 3 times daily for 15 days. *Acta Psychiatr Scand Suppl* **360** 91-93. (1990)

68. Raaflaub J., Haefelfinger P., Trautmann K.H. Single-dose pharmacokinetics of the MAO-inhibitor moclobemide in man. *Arzneimittelforschung* **34** 80-82. (1984)

69. Bhakay A., Rahman M., Dave R.N., Bilgili E. Bioavailability Enhancement of Poorly Water-Soluble Drugs via Nanocomposites: Formulation(-)Processing Aspects and Challenges. *Pharmaceutics* **10**. (2018)

70. Accorda Therapeutics Inc. Zanaflex prescribing information. 2013 [cited 2019 25 November 2019]Available from: <https://www.accessdata.fda.gov/drugsatfda_docs/label/2013/021447s011_020397s026lbl.pdf>

71. Granfors M.T., Backman J.T., Laitila J., Neuvonen P.J. Tizanidine is mainly metabolized by cytochrome p450 1A2 in vitro. *Br J Clin Pharmacol* **57** 349-353. (2004)

72. Momo K.*, et al.* Effects of mexiletine, a CYP1A2 inhibitor, on tizanidine pharmacokinetics and pharmacodynamics. *J Clin Pharmacol* **50** 331-337. (2010)

73. Granfors M.T., Backman J.T., Neuvonen M., Ahonen J., Neuvonen P.J. Fluvoxamine drastically increases concentrations and effects of tizanidine: a potentially hazardous interaction. *Clin Pharmacol Ther* **75** 331-341. (2004)

74. Shellenberger M.K., Groves L., Shah J., Novack G.D. A controlled pharmacokinetic evaluation of tizanidine and baclofen at steady state. *Drug Metab Dispos* **27** 201-204. (1999)

75. Henney H.R., 3rd, Shah J. Relative bioavailability of tizanidine 4-mg capsule and tablet formulations after a standardized high-fat meal: a single-dose, randomized, open-label, crossover study in healthy subjects. *Clin Ther* **29** 661-669. (2007)

76. Backman J.T., Schroder M.T., Neuvonen P.J. Effects of gender and moderate smoking on the pharmacokinetics and effects of the CYP1A2 substrate tizanidine. *Eur J Clin Pharmacol* **64** 17-24. (2008)

77. Backman J.T., Granfors M.T., Neuvonen P.J. Rifampicin is only a weak inducer of CYP1A2-mediated presystemic and systemic metabolism: studies with tizanidine and caffeine. *Eur J Clin Pharmacol* **62** 451-461. (2006)

78. Shah J., Wesnes K.A., Kovelesky R.A., Henney H.R., 3rd. Effects of food on the single-dose pharmacokinetics/pharmacodynamics of tizanidine capsules and tablets in healthy volunteers. *Clin Ther* **28** 1308-1317. (2006)

79. Tse F.L., Jaffe J.M., Bhuta S. Pharmacokinetics of orally administered tizanidine in healthy volunteers. *Fundam Clin Pharmacol* **1** 479-488. (1987)

80. Al-Ghazawi M., Alzoubi M., Faidi B. Pharmacokinetic comparison of two 4 mg tablet formulations of tizanidine. *Int J Clin Pharmacol Ther* **51** 255-262. (2013)

81. Labbe L.*, et al.* Pharmacokinetic and pharmacodynamic interaction between mexiletine and propafenone in human beings. *Clin Pharmacol Ther* **68** 44-57. (2000)

82. Wei X.*, et al.* Inhibition of human liver cytochrome P-450 1A2 by the class IB antiarrhythmics mexiletine, lidocaine, and tocainide. *J Pharmacol Exp Ther* **289** 853-858. (1999)

83. Campbell N.P., Kelly J.G., Adgey A.A., Shanks R.G. Mexiletine in normal volunteers. *Br J Clin Pharmacol* **6** 372-373. (1978)

84. Begg E.J., Chinwah P.M., Webb C., Day R.O., Wade D.N. Enhanced metabolism of mexiletine after phenytoin administration. *Br J Clin Pharmacol* **14** 219-223. (1982)

85. Pringle T.*, et al.* Dose independent pharmacokinetics of mexiletine in healthy volunteers. *Br J Clin Pharmacol* **21** 319-321. (1986)

86. Kusumoto M.*, et al.* Lack of pharmacokinetic interaction between mexiletine and omeprazole. *Ann Pharmacother* **32** 182-184. (1998)

87. Kusumoto M.*, et al.* Effect of fluvoxamine on the pharmacokinetics of mexiletine in healthy Japanese men. *Clin Pharmacol Ther* **69** 104-107. (2001)

88. Pentikainen P.J., Halinen M.O., Helin M.J. Pharmacokinetics of intravenous mexiletine in patients with acute myocardial infarction. *J Cardiovasc Pharmacol* **6** 1-6. (1984)

89. Joeres R., Klinker H., Heusler H., Epping J., Richter E. Influence of mexiletine on caffeine elimination. *Pharmacol Ther* **33** 163-169. (1987)

90. Ezuruike U.*, et al.* Risk-Benefit Assessment of Ethinylestradiol Using a Physiologically Based Pharmacokinetic Modeling Approach. *Clin Pharmacol Ther* **104** 1229-1239. (2018)

91. Stanczyk F.Z., Archer D.F., Bhavnani B.R. Ethinyl estradiol and 17beta-estradiol in combined oral contraceptives: pharmacokinetics, pharmacodynamics and risk assessment. *Contraception* **87** 706-727. (2013)

92. Back D.J.*, et al.* The pharmacokinetics of levonorgestrel and ethynylestradiol in women - studies with Ovran and Ovranette. *Contraception* **23** 229-239. (1981)

93. Back D.J.*, et al.* An investigation of the pharmacokinetics of ethynylestradiol in women using radioimmunoassay. *Contraception* **20** 263-273. (1979)

94. Back D.J., Grimmer S.F., Rogers S., Stevenson P.J., Orme M.L. Comparative pharmacokinetics of levonorgestrel and ethinyloestradiol following intravenous, oral and vaginal administration. *Contraception* **36** 471-479. (1987)

95. Orme M., Back D.J., Ward S., Green S. The pharmacokinetics of ethynylestradiol in the presence and absence of gestodene and desogestrel. *Contraception* **43** 305-316. (1991)

96. Kuhnz W., Humpel M., Biere H., Gross D. Influence of repeated oral doses of ethinyloestradiol on the metabolic disposition of [13C2]-ethinyloestradiol in young women. *Eur J Clin Pharmacol* **50** 231-235. (1996)

97. Goebelsmann U., Hoffman D., Chiang S., Woutersz T. The relative bioavailability of levonorgestrel and ethinyl estradiol administered as a low-dose combination oral contraceptive. *Contraception* **34** 341-351. (1986)

98. Stanczyk F.Z.*, et al.* Plasma levels and pharmacokinetics of norethindrone and ethinylestradiol administered in solution and as tablets to women. *Contraception* **28** 241-251. (1983)

99. Zhang C.*, et al.* An open-label, two-period comparative study on pharmacokinetics and safety of a combined ethinylestradiol/gestodene transdermal contraceptive patch. *Drug Des Devel Ther* **11** 725-731. (2017)

100. Martin P.*, et al.* Effects of Fostamatinib on the Pharmacokinetics of Oral Contraceptive, Warfarin, and the Statins Rosuvastatin and Simvastatin: Results From Phase I Clinical Studies. *Drugs R D* **16** 93-107. (2016)

101. Stockis A., Watanabe S., Fauchoux N. Interaction between brivaracetam (100 mg/day) and a combination oral contraceptive: a randomized, double-blind, placebo-controlled study. *Epilepsia* **55** e27-31. (2014)

102. Sidhu J., Job S., Singh S., Philipson R. The pharmacokinetic and pharmacodynamic consequences of the co-administration of lamotrigine and a combined oral contraceptive in healthy female subjects. *Br J Clin Pharmacol* **61** 191-199. (2006)

103. Kothare P.A.*, et al.* Effect of exenatide on the pharmacokinetics of a combination oral contraceptive in healthy women: an open-label, randomised, crossover trial. *BMC Clin Pharmacol* **12** 8. (2012)

104. Timmer C.J., Mulders T.M. Pharmacokinetics of etonogestrel and ethinylestradiol released from a combined contraceptive vaginal ring. *Clin Pharmacokinet* **39** 233-242. (2000)

105. Balogh A.*, et al.* Influence of ethinylestradiol-containing combination oral contraceptives with gestodene or levonorgestrel on caffeine elimination. *Eur J Clin Pharmacol* **48** 161-166. (1995)

106. Granfors M.T., Backman J.T., Laitila J., Neuvonen P.J. Oral contraceptives containing ethinyl estradiol and gestodene markedly increase plasma concentrations and effects of tizanidine by inhibiting cytochrome P450 1A2. *Clin Pharmacol Ther* **78** 400-411. (2005)

107. Boehringer Ingelheim. Study c13608215. A study to investigate the pharmacokinetic drug-drug interaction following oral administration of ethinylestradiol/levonorgestrel (Microgynon®) and BI 409306 in healthy Korean premenopausal female subjects (an open-label, two-period, fixed-sequence study).
