# Supplementary material for: A Physiologically-Based Pharmacokinetic (PBPK) Model Network for the Prediction of CYP1A2 and CYP2C19 Drug–Drug–Gene Interactions with Fluvoxamine, Omeprazole, S-mephenytoin, Moclobemide, Tizanidine, Mexiletine, Ethinylestradiol, and Caffeine
Source: Pharmaceutics. 2020 Dec 8;12(12):1191. doi: 10.3390/pharmaceutics12121191 (PMC7764797; doi:10.3390/pharmaceutics12121191)
Supplement: Supplementary file 1 [file pharmaceutics-12-01191-s001.zip › pharmaceutics-1010984 supplementary/PBPK_manuscript_supplement S2a_Pharmaceutics.docx]

**Electronic Supplementary Material**

# **Supplement S2****a: Model development and evaluation (CYP2C19 predominant): fluvoxamine, omeprazole, moclobemide, S-mephenytoin**

- 1. Fluvoxamine model
     1. Model Development Strategy

Several iterations of prediction-optimization cycles were performed, visually comparing predictions to digitized concentration time-curves found in publications or optimizing certain model parameters with a fitting algorithm. In general, the following step-wise workflow was followed:

1. Predict intravenous (i.v.) profiles based on in vitro data alone and optimize distribution model and metabolism parameters
2. Predict single dose data following oral solution
3. Optimize intestinal permeability and revisit metabolism model
4. Predict single dose data following enteric-coated tablet
5. Multiple dose predictions with preliminary model
6. Refine model and optimize permeability and metabolism parameters
7. Predict DDI with caffeine (fluvoxamine as CYP1A2 inhibition)

The main metabolic route of fluvoxamine is via CYP2D6. A linear CYP2D6 elimination mechanism was explored first. As shown later, predictions of (multiple) high dose data were not captured well with a sole linear pathway, and a saturable CYP2D6 Michaelis-Menten elimination was therefore considered, which better matched the observed data. Finally, a second pathway via CYP1A2 was implemented as suggested by other groups.[^1^](#_ENREF_1)^,^[^2^](#_ENREF_2)

Table S2.1 Model development steps – fluvoxamine model

| **Step** | **Figure and Table in text** | **Purpose** | **Data** |
| --- | --- | --- | --- |
| **1** | Figure S2.1 | Selection distribution model and CL | Iga 2015[^3^](#_ENREF_3) |
| **2** |  | Propagation from i.v. to oral model | Iga 2015[^3^](#_ENREF_3)  De Vries 1993[^4^](#_ENREF_4) |
| **3** | Table S2.2  Figure S2.2 | Parameter estimation intestinal permeability | Iga 2015[^3^](#_ENREF_3)  De Vries 1993[^4^](#_ENREF_4) |
| **4** | Figure S2.3 | Fitting enteric coated tablet | All at 50 mg p.o.: Iga 2015^[3](#_ENREF_3)^  Orlando 2009[^5^](#_ENREF_5), Kunii 2005[^6^](#_ENREF_6)  Fukasawa 2006[^7^](#_ENREF_7)  Spigset 1997[^8^](#_ENREF_8) |
| **5** |  | Multiple dose predictions preliminary model | Spigset 1998[^9^](#_ENREF_9)  Fleishaker 1994[^10^](#_ENREF_10) |
| **6** | Figure S2.4a -  Figure S2.4d  Table S2.3  Table S2.4 | Model refinement; adding CYP1A2 pathway single and multiple daily dose) | Iga 2015[^3^](#_ENREF_3), Carillo 1996[^11^](#_ENREF_11)  Spigset 1997[^8^](#_ENREF_8), Spigset 1998[^9^](#_ENREF_9), De Vries 1993[^4^](#_ENREF_4)  FDA_ClinPharmReview LuvoxCR^[12](#_ENREF_12)^  Labellarte 2004[^13^](#_ENREF_13)  Fleishaker 1994[^10^](#_ENREF_10) |
| **7** |  | DDI Predictions with caffeine as victim drug | Jeppesen 1996[^14^](#_ENREF_14) |

- - 1. Model Development Fluvoxamine
       1. **Selecting a distribution model using i.v. data only**

Different distribution models are available and were tested in PK-Sim. In line with previous PBPK models, the Rodgers and Rowland distribution model seemed best suited to describe the distribution after i.v. administration. Fitting the linear CYP2D6 clearance (single pathway) to the observed data from Iga et al. 2015[^3^](#_ENREF_3) using the Rodgers & Rowland model, the fit was quite adequate (Figure S2.1):


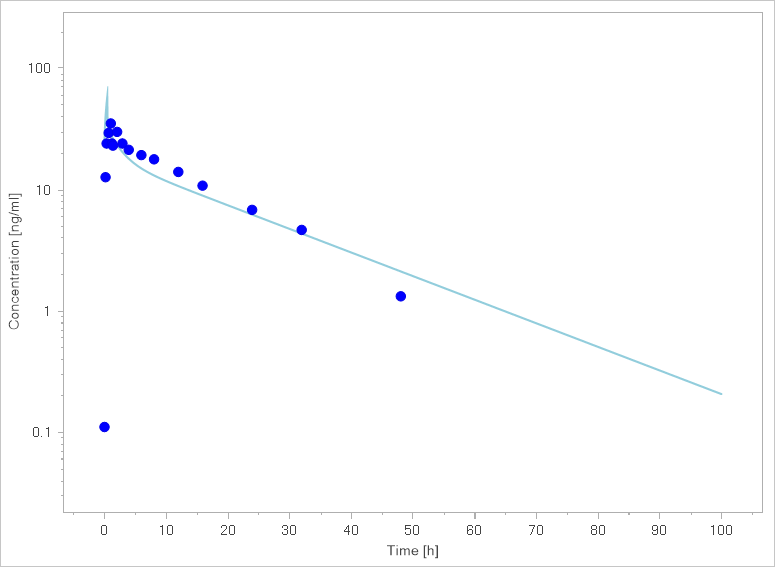


Figure S2.1 Observed (dots) versus predicted (line) fluvoxamine plasma concentrations using the Rodgers & Rowland distribution model considering recombinant CYP2D6 clearance. Observed mean data from Iga et al.2015^[3](#_ENREF_3)^

The estimated in vitro clearance was: 9.01 µl/min/pmol rec. CYP2D6 enzyme. Renal clearance was set equal to the glomerular filtration rate (GFR) as was done by Alqahtani et al.[^1^](#_ENREF_1). This results in less than 2% of parent fluvoxamine being excreted in urine, comparable to that seen in the literature (e.g. 4% in de Vries et al 1993[^4^](#_ENREF_4)).

- - - 1. **Predicting p.o. profiles following oral solution**

As a next step, the intestinal permeability and CYP2D6 saturable clearance process (Michaelis-Menten kinetics) were fitted simultaneously. The fit was performed simultaneously on the oral data from De Vries et al 1993[^4^](#_ENREF_4) and the i.v. data from Iga et al 2015.[^3^](#_ENREF_3)


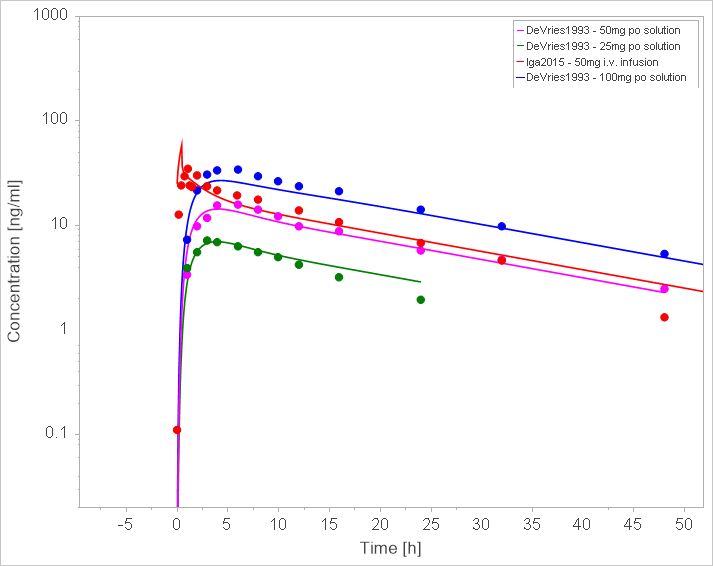


Figure S2.2 Observed (dots) versus predicted (line) fluvoxamine plasma concentrations after oral and intravenous administration with optimized intestinal permeability and non-linear elimination pathway of CYP2D6.

The estimated parameters were as follows:

Table S2.2 Intestinal permeability and CYP2D6 saturable clearance estimates for fluvoxamine

| **Identification Parameter** | **Estimate** |
| --- | --- |
| V_max_ (liver tissue) | 4.95 µmol/min/kg tissue |
| Km | 0.80 µmol/L |
| Intestinal permeability (transcellular) | 1.02E-5 cm/min  (start value: 4.58E-5 cm/min) |

- - - 1. **Predicting p.o. profiles following oral enteric-coated tablets**

In order to predict the profile following oral administration of an enteric-coated tablet (the most common formulation of fluvoxamine), a Weibull dissolution model was used. A rather fast dissolution time of 10 min was used together with a lag time of 30 min, based on the standard gastric emptying time. As shown in Figure S2.3 the predictions described the profile very well, especially t_max_ (time to maximal concentration).


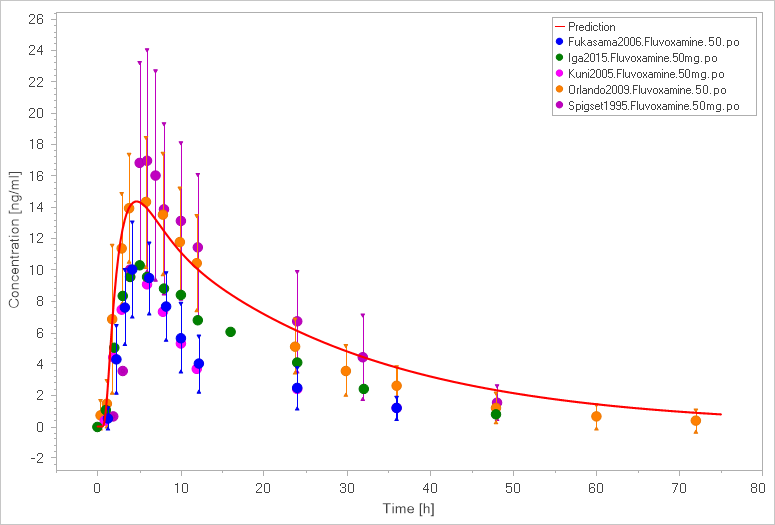


Figure S2.3 Concentration-time profiles following oral administration of 50 mg fluvoxamine maleate as an enteric-coated tablet.

- - - 1. **Model refinement – introducing the CYP1A2 pathway**

To identify a second elimination pathway, the model was fitted to multiple datasets simultaneously to ensure that enough information to identify such pathway is contained in the data. This included data from CYP2D6 poor metabolizers (PM) where the CYP2D6 pathway was switched-off, and including the fraction metabolized via each pathway (29.5% via CYP1A2, 66.5% CYP2D6) as it was employed by Alqahtani et al.[^1^](#_ENREF_1) A brief overview of the data used in the fitting process is shown in Table S2.3.

Table S2.3 Data sources for the final parameter optimization of the fluvoxamine model

| **Source** | **Dose (mg)** | **Route** | **Frequency** | **Individual** | **Comment** |
| --- | --- | --- | --- | --- | --- |
| Iga2015[^3^](#_ENREF_3) | 50 | i.v. | s.d. | Japanese |  |
| Carillo 1996[^11^](#_ENREF_11) | 50 | p.o. | s.d. | European | CYP2D6 PM |
| De Vries 1993[^4^](#_ENREF_4) | 25, 50, 100 | p.o. | s.d. | European | Oral solution |
| Spigset 1997[^8^](#_ENREF_8) | 50 | p.o. | s.d. | European | CYP2D6 PM |
| FDA review Luvox[^12^](#_ENREF_12) | 100 | p.o. | s.d. | European |  |
| FDA review Luvox[^12^](#_ENREF_12) | 100 | p.o. | q.d. | European |  |
| Labellarte 2004[^13^](#_ENREF_13) | 25-50-75-100-125-150 weekly increments | p.o. | b.i.d. | European children and adolescents |  |
| Spigset 1998[^9^](#_ENREF_9) | 50 🡪 100 | p.o. | b.i.d. | European |  |
| Fleishaker 1994[^10^](#_ENREF_10) | 12.5-25-50-100 weekly increments | p.o. | b.i.d. | European |  |

The following parameters were optimized:

- Specific intestinal permeability
- K_m_ of CYP2D6 metabolism
- K_cat_ of CYP2D6 metabolism (corresponding to V_max_)
- Metabolic (linear) clearance of CYP1A2

The final parameter estimates, and their 95% confidence interval are shown Table 2.4. All the other parameters were set to default PK-Sim standard values.

Table S2.4 Final parameter estimates of the fluvoxamine model

| **Identification Parameter** | **95% Confidence Interval** |
| --- | --- |
| Specific intestinal permeability (transcellular) | 1.23E-5 +- 4.99E-6 [cm/min] |
| Km_2D6 | 9.78E-4 +- 1.81E-3 [µmol/l] |
| kcat_2D6 | 0.04 +- 0.01 [1/min] |
| In vitro CL_CYP1A2 | 0.54 +- 0.05 [µl/min/pmol rec. enzyme] |

The parameters were estimated with a reasonable precision, albeit K_m_ and K_cat_ (i.e V_max_) were correlated as shown in the correlation matrix below (Table S2.5). This indicates the need for more data to estimate the saturable process with a better precision.

Table S2.5 Correlation matrix for km_2D6, kcat_2D6 and specific 1A2 clearance

|  | **Specific intestinal permeability (transcellular** | **Km_2D6** | **CL_1A2** | **Kcat_2D6** |
| --- | --- | --- | --- | --- |
| **Specific intestinal permeability (transcellular)** | 1.0000 | 0.0084 | 0.0054 | 0.0024 |
| **Km_2D6** | 0.0084 | 1.0000 | -0.5174 | 0.9296 |
| **CL_1A2** | 0.0054 | -0.5174 | 1.0000 | -0.7275 |
| **Kcat_2D6** | 0.0024 | 0.9296 | -0.7275 | 1.0000 |

Simulations were run with a Standard European / Caucasian male individual set to PK-Sim default biometrics and including CYP1A2 and CYP2D6 expression. Single dose data were very well predicted, as shown in Figure S2.4; the predictions aligned closer to the studies where higher concentrations were observed, with the most likely explanation being the smoking status of the subjects in these studies. Cigarette smoke is known to induce the CYP1A2 enzyme system,[^15^](#_ENREF_15) thus lower fluvoxamine concentrations would be expected in smokers due to the higher metabolism. Although there is no built-in smoking status in PK-Sim, this could be modelled by adapting CYP1A2 expression.

Low concentrations:

- - Fukasawa: healthy Japanese males; 6 **smokers**/ 6 non-smokers; no PM
  - Iga: from Japanese drug information - no details given
  - Kuni: male Japanese volunteers, 5 **smokers**/5 non-smokers; no PM
  - Spigset -smokers: healthy young Europeans; **12 smokers**; no PM

High concentrations:

- - Spigset - **non-smokers**: healthy young Europeans; 12 non-smokers; no PM
  - Orlando: 10 young healthy European **non-smokers**; genotype unknown
  - De Vries: 12 young male European volunteers; **smoking status unknown,** genotype unknown

| 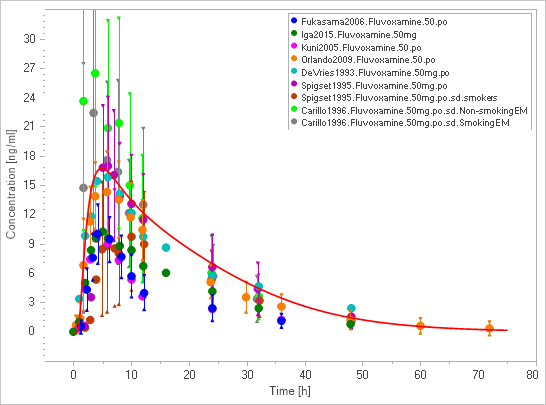 |
| --- |

Figure S2.4 Oral administration of 50 mg fluvoxamine maleate

For unknown reasons, concentrations reported by Carillo were much higher and could not be described by the model. The simulated concentration–time profile following i.v. administration is shown in Figure S2.5. The shape of the curve follows the observed course well.


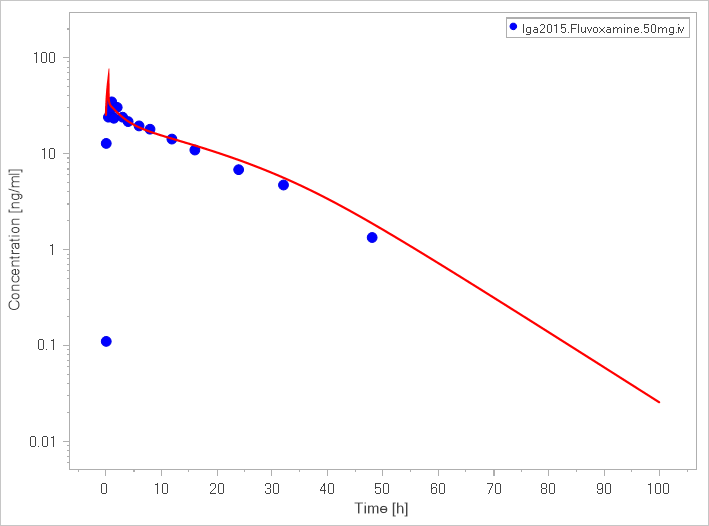


Figure S2.5 Simulation of 50 mg fluvoxamine given as 30 min short infusion.

The predicted time profiles for the multiple dose schedules also matched well with observed data, as can be seen in Figure S2.6 a to d.

| **A**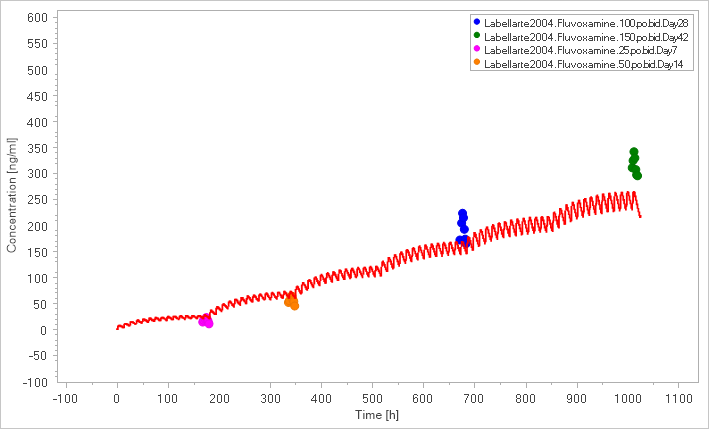 | **B** 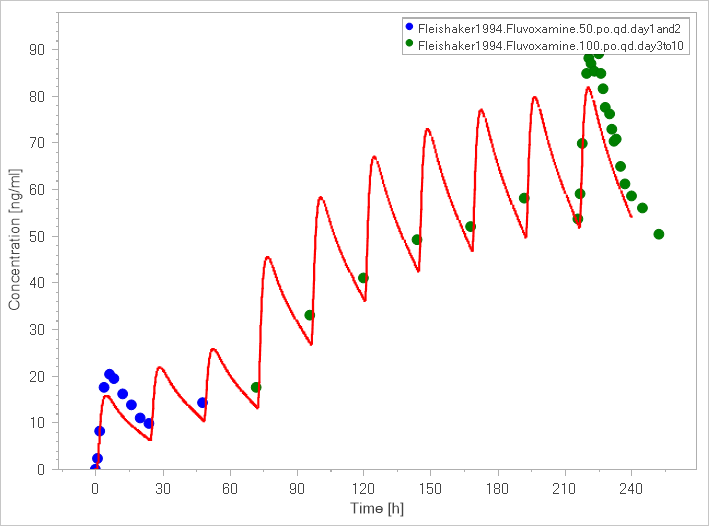 |
| --- | --- |
| **C** 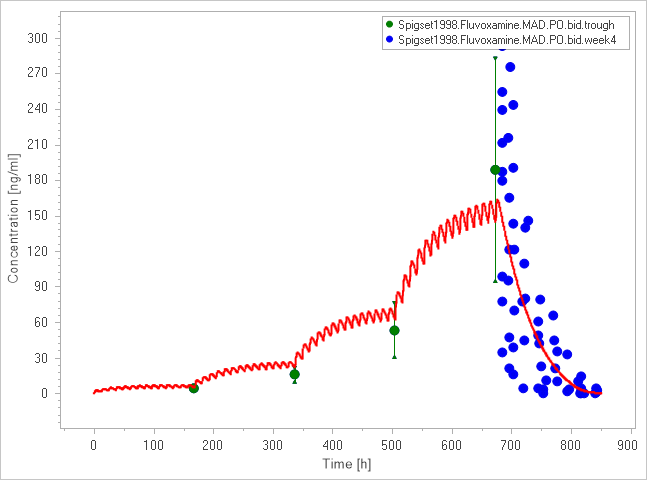 | **D**  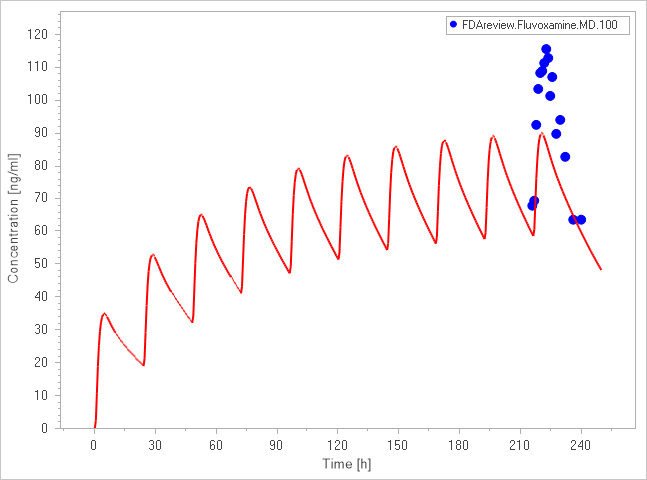 |

Figure S2.6a Simulated ascending multiple doses profile of fluvoxamine. Observed data from Labellarte 2004^[13](#_ENREF_13)^

Figure S2.6b Simulated multiple doses profile of fluvoxamine. Observed data from Fleishaker 1994^[10](#_ENREF_10)^

Figure S2.6c Simulated multiple doses profile of fluvoxamine. Observed data from Spigset 1998^[9](#_ENREF_9)^

Figure S2.6d Simulated multiple doses profile of fluvoxamine. Observed data from FDA review of Luvox^[12](#_ENREF_12)^

- - 1. Assumptions and Limitations for the Fluvoxamine Model

The developed fluvoxamine model included the following assumption:

| **Assumption** | **Justification/impact** |
| --- | --- |
| Renal elimination accounts only for a minor part of total clearance and is driven by passive glomerular filtration | No active renal excretion described in literature. Renal clearance is minor[^16^](#_ENREF_16), and can be described by GFR in PK-Sim. |

- - 1. Fluvoxamine Model Evaluation and Qualification

The final model was qualified by comparing predicted concentration-time profiles to observed data obtained from an internal clinical study (NCT02853136).[^17^](#_ENREF_17) These data were not used during model development in any parameter estimation procedures.

In the clinical study, fluvoxamine-maleate was given to 18 healthy volunteers on 4 consecutive days with the following dosing: 50-100-100-100 mg. A virtual Caucasian population (N=2000) with age
(18–50 y) and BMI (18.5–29.90 kg/m^2^) similar to those of the study participants was used for qualification of the fluvoxamine model. Ontogeny and variability of CYP1A2 and CYP2D6 were taken from the PK-Sim database.

Overall, the model that was build using external data well described the internal fluvoxamine data, both in terms of average profile and variability (Figure S2.7).


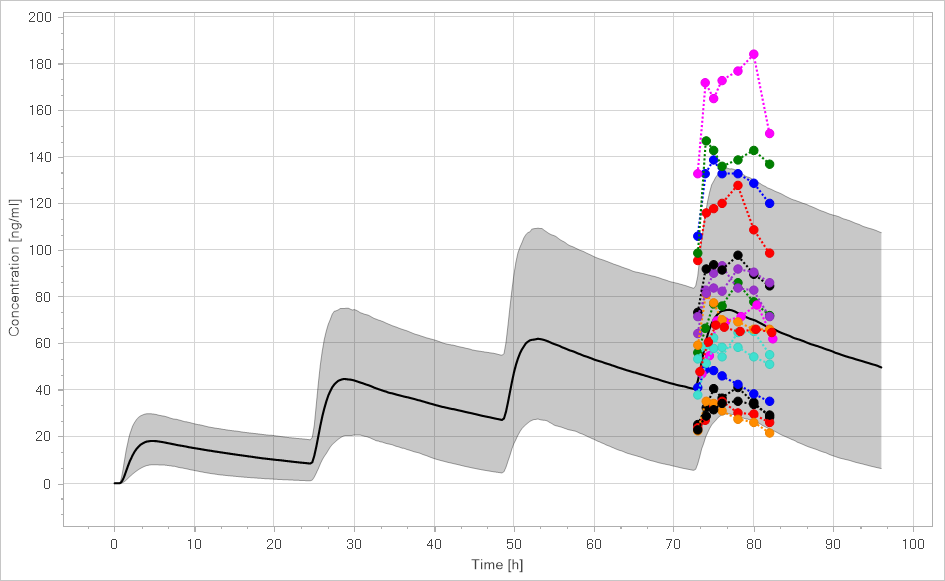


Figure S2.7 Simulated and observed plasma concentrations of fluvoxamine over 4 days of dosing

The black line shows the median concentration from 2000 simulated individuals, the gray area encompasses the 5th and 95th percentile of the concentrations. The colored symbols are observed concentrations from 18 healthy volunteers in study NCT02853136.

- - 1. Sensitivity Analysis for Fluvoxamine Model

The results of one-way sensitivity analysis with AUC and C_max_ as outcome parameters are shown in Figure S2.8 and Figure S2.9, respectively. The standard set of PK-Sim model parameters visible in simple view (N=94, including solubility, metabolism by enzyme, organ volumes and blood flows) were investigated.


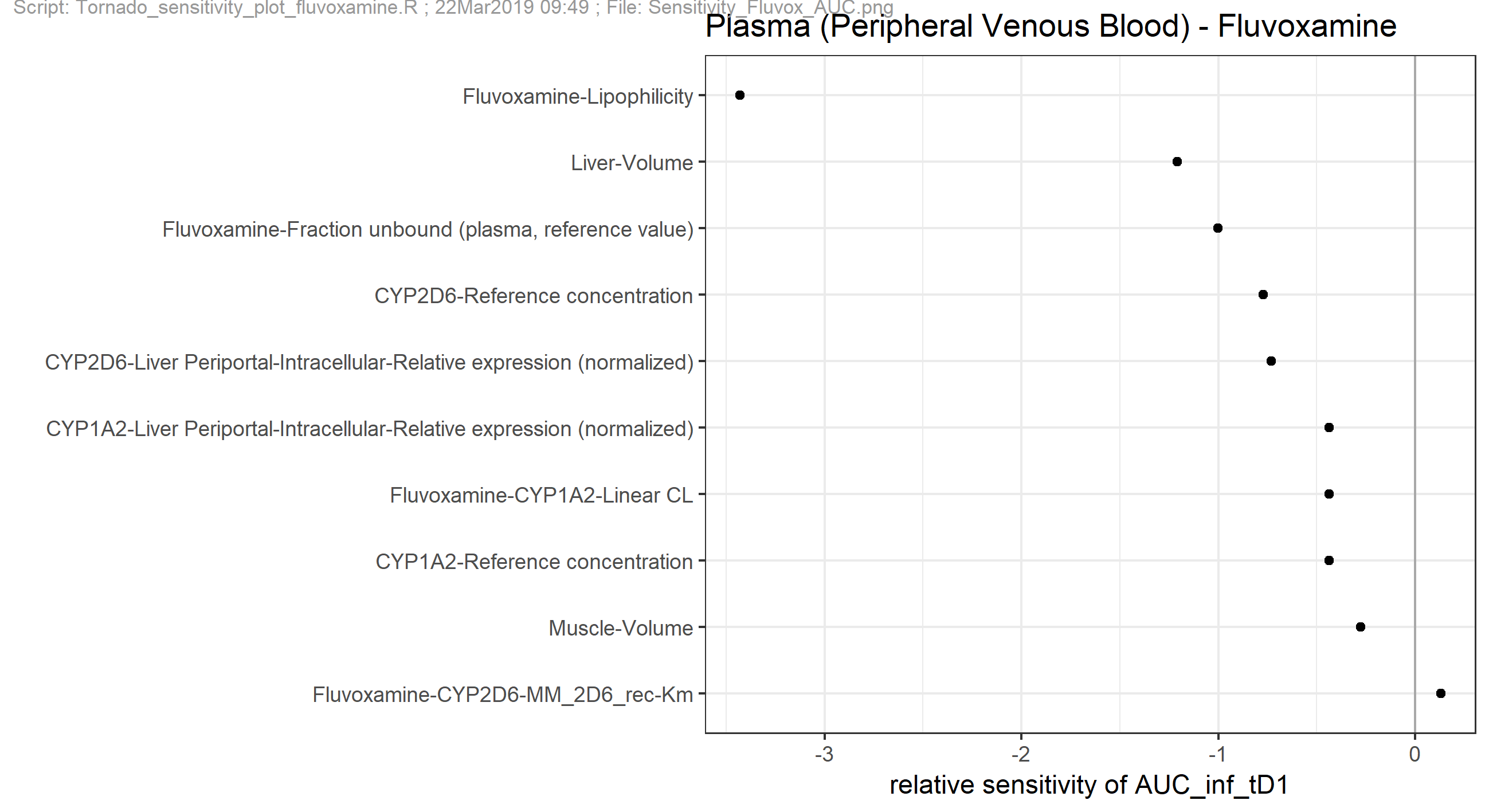


Figure S2.8 Sensitivity Analysis Fluvoxamine AUC (0 to infinity on day 1)


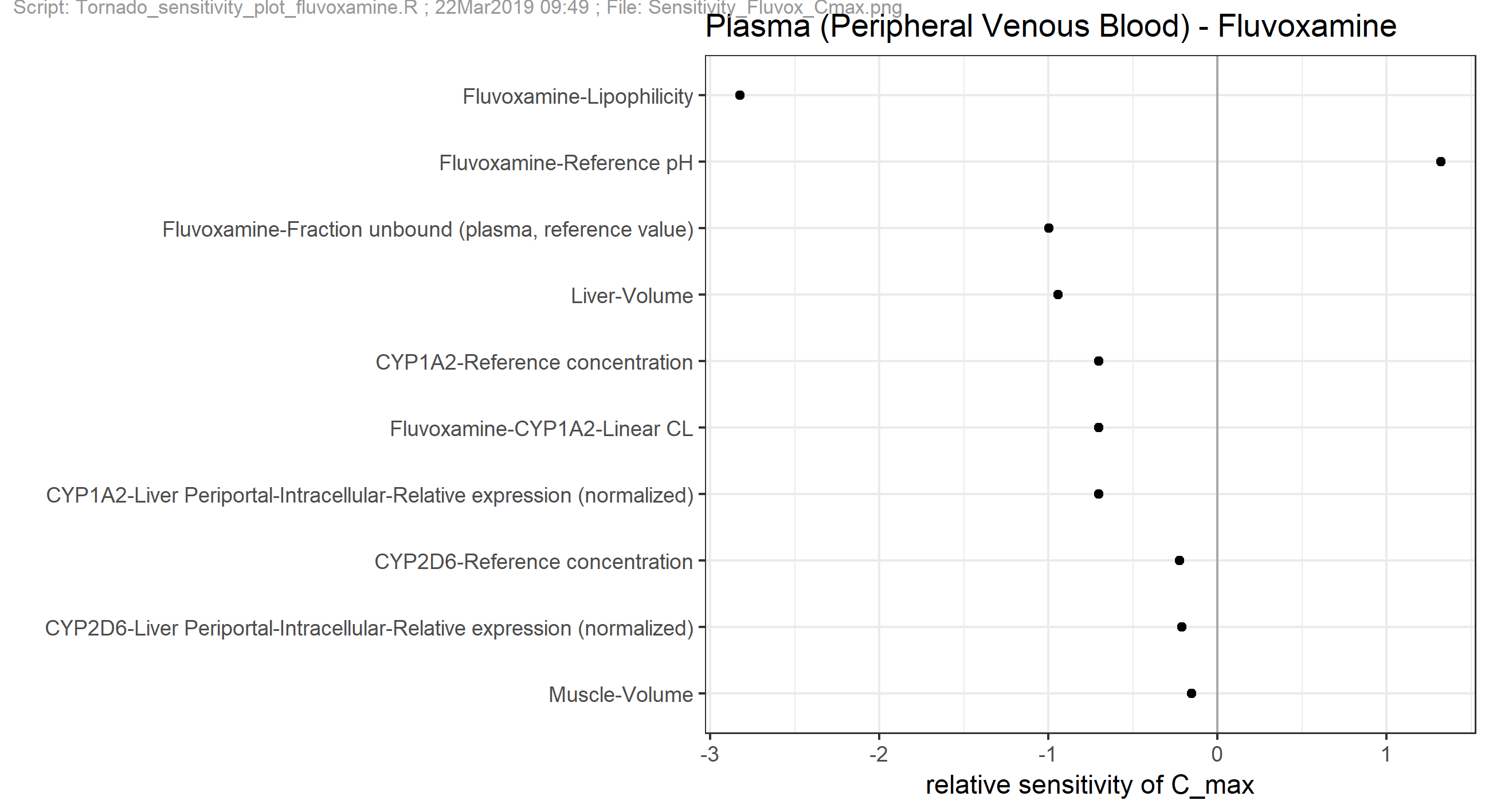


Figure S2.9 Sensitivity Analysis Fluvoxamine C_max_

Lipophilicity (logP) has the highest impact on the PK predictions for fluvoxamine. In the current model we used the same logP (3.38) value that Alqahtani et al.[^1^](#_ENREF_1) also used for their model. Other sources report a very similar value (3.2 DrugBank, 3.61 previous preliminary modelling). Therefore, it may be concluded that the lipophilicity used in the PBPK model is unlikely to be substantially different from reality.

The liver volume in PK-Sim determines the amount of metabolizing enzymes and hence directly impacts the total body clearance (and thus the AUC). The value used for the liver volume is a function of body weight in PK-Sim.

Given that fluvoxamine is a basic drug with a pK_a_ of 9.16, solubility is pH-dependent, and hence the reference pH at which the aqueous solubility was determined impacts the absorption (speed) and C_max_. However, devices to determine the pH in an aqueous medium are fairly accurate and the reported values can be considered invariant.

For the fraction unbound we used the value as reported by Alqahtani et al. (0.23); a similar value is reported in DrugBank for fluvoxamine (0.20).

Most other parameters relate to the relative expression or reference concentration of the CYP enzymes involved in fluvoxamine’s metabolism. These parameters are expected to influence fluvoxamine levels.

In summary, findings from the sensitivity analysis were in line with expectations, i.e. that these parameters influence the predicted AUC and C_max_. Attention should be paid to the reference concentration of CYP2C19 and CYP1A2.

- 1. OMEPRAZOLE MODEL
     1. Model Development Strategy

Similar to other approaches from literature,[^18^](#_ENREF_18) two models for S-omeprazole and R-omeprazole respectively were built and then combined in MoBi^[19](#_ENREF_19)^ to a new observer for racemic omeprazole.

Several iterations of prediction-optimization cycles were performed, visually comparing predictions to digitized concentration time-curves found in publications or optimizing certain model parameters with a fitting algorithm.

In general, the following step-wise workflow was followed:

1a. Define distribution and metabolism for S-omeprazole

1b. Mechanism-based inactivation of CYP2C19 by omeprazole

2. Define S-omeprazole absorption based on p.o. dosing

3. Capsule formulation

3a. Building racemic omeprazole

3b. Adjust CYP2C19 expression in gut

4. Define metabolism for R-omeprazole

5. Refine CYP3A4 metabolism on CYP2C19 PM data

6. Refine CYP2C19 metabolism on CYP2C19 EM data

7. DDI predictions

Table S2.6 Model development steps – omeprazole model

| **Step** | **Figure and Table in text** | **purpose** | **Data** |
| --- | --- | --- | --- |
| **1a** | Table S2.7 | Define distribution and metabolism for S-omeprazole | Hassan-Alin 2000[^20^](#_ENREF_20) S-omeprazole  Wilder-Smith 2005[^21^](#_ENREF_21) S-omeprazole i.v. |
| **1b** | Figure S2.10 | Mechanism-based inactivation of CYP2C19 by omeprazole |  |
| **2** |  | Define S-omeprazole absorption based on p.o. dosing | Hassan-Alin 2005^[22](#_ENREF_22)^ S-omeprazole |
| **3** | Table S2.8 | Capsule formulation | Wilder-Smith 2005[^21^](#_ENREF_21) S-omeprazole capsule |
| **3a** |  | Building racemic omeprazole | Hassan-Alin 2005^[22](#_ENREF_22)^ omeprazole  Andersson 1990^[23](#_ENREF_23)^ omeprazole |
| **3b** |  | Adjust CYP2C19 expression in gut | Hassan-Alin 2005[^22^](#_ENREF_22) omeprazole  Olivares-Morales 2016[^24^](#_ENREF_24) |
| **4** | Table S2.9 | Define metabolism for R-omeprazole | Andersson 2000^[25](#_ENREF_25)^ R-omeprazole  Hassan-Alin 2005^[22](#_ENREF_22)^ R-omeprazole  (weights for timepoints after 6h from dose were set to zero, due to uncertainty in digitization from y-linear scale plots) |
| **5** |  | Refine CYP3A4 metabolism on CYP2C19 PM data | Uno 2007^[26](#_ENREF_26)^ omeprazole PM  Andersson 2000^[25](#_ENREF_25)^ R-/S-omeprazole PM  Regårdh 1990^[27](#_ENREF_27)^ omeprazole ID2=PM  FDA[^28^](#_ENREF_28) S-omeprazole PM |
| **6** | Table S2.10  Table S2.11 | Refine CYP2C19 metabolism on CYP2C19 EM data | Hassan-Alin 2000[^20^](#_ENREF_20)  S-omeprazole Wilder-Smith 2005[^21^](#_ENREF_21) (i.v.) S-omeprazole  Andersson 1990[^23^](#_ENREF_23) omeprazole  Andersson 1991[^29^](#_ENREF_29) omeprazole  Oosterhuis 1992[^30^](#_ENREF_30)  omeprazole  Uno 2007[^26^](#_ENREF_26) omeprazole EM  Regårdh 1990[^27^](#_ENREF_27) omeprazole  Röhss 2007[^31^](#_ENREF_31) S-omeprazole |
| **7** |  | 7. DDI predictions | Yasui-Furukori 2004[^32^](#_ENREF_32)  Cho et al. 2002[^33^](#_ENREF_33) |

- - 1. Model Development Omeprazole
       1. **Define distribution model and lipophilicity for S-omeprazole**

As a first step, only i.v. data after single and multiple doses were used to identify the most suitable distribution method, lipophilicity and metabolic clearances (CYP2C19, CYP3A4). Time dependent autoinhibition (TDI) mechanism as reported by Wu et al.[^18^](#_ENREF_18) was included throughout. The predefined “Standard European Male for DDI” individual was used (age=30y, weight=73kg, height=176cm, BMI=23.57kg/m^2^). CYP2C19 expression from the PK-Sim in-built RT-PCR database[^34^](#_ENREF_34)^,^[^35^](#_ENREF_35)^,^[^36^](#_ENREF_36) was added.

The Rodgers & Rowland model appeared to best describe the distribution phase. Model parameters were estimated with good precision as shown in Table S2.7.

Table S2.7 Lipophilicity, CYP2C19 and CYP3A4 clearance estimates for S-omeprazole based on i.v. data

| **Identification Parameter** | **95% Confidence Interval** |
| --- | --- |
| Lipophilicity | 1.68 +- 0.35 [Log Units] |
| CYP2C19 Specific clearance | 11.98 +- 1.85 [1/min] |
| CYP3A4 Specific clearance | 0.25 +- 0.06 [1/min] |

- - - 1. **Mechanism based inactivation of CYP2C19 by omeprazole**

To illustrate (R-/S-) omeprazole time-dependent autoinhibition (TDI) on CYP2C19, simulations including or excluding TDI were compared.

The implementation in PK-Sim of mechanism-based inactivator follows the equation shown below:

$$\frac{dE_{cat}(t)}{dt}=k_{deg}\cdot E_{0}-\left( k_{deg}+\frac{k_{inact}\cdot I\left( t \right)}{K_{I}+I\left( t \right)} \right)*E_{cat}(t)$$

Where, the two principal kinetic constants are Kinact (the maximum rate of inactivation) and Kinact_half (or K_I_, the concentration at which the inactivation rate is half-maximal). The inclusion of mechanism-based inactivation process was necessary to describe multiple dose (oral solution) data, which are characterized by a lower CL( Figure S2.10).


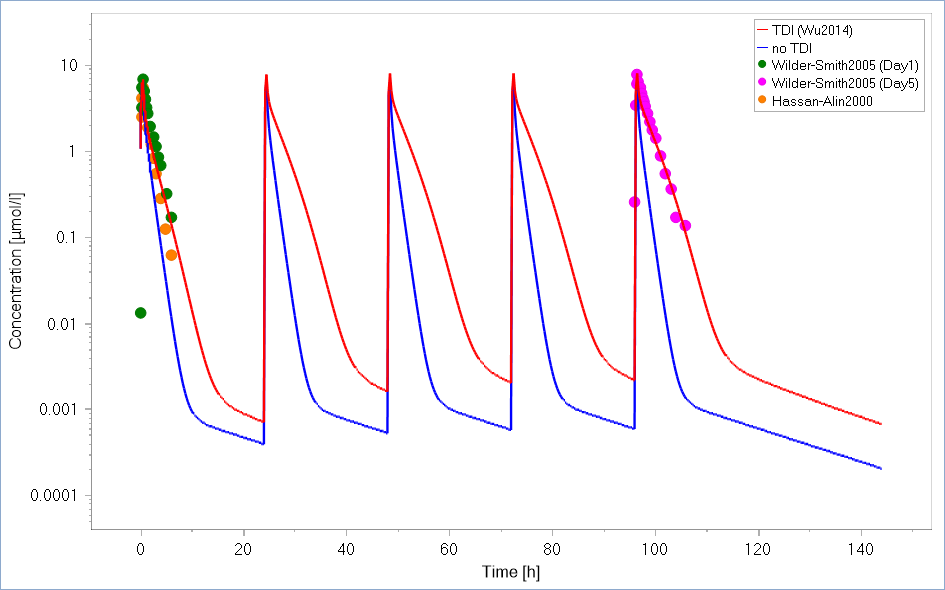


Figure S2.10 S-omeprazole concentration-time profile following 40 mg single and multiple oral solution administrations. Simulations were performed including (red) or excluding (blue) mechanism-based inactivation of CYP2C19.

- - - 1. **Predicting S-omeprazole p.o. profiles following oral solution**

As a next step, data obtained after oral solution administration were used to adjust gastrointestinal permeability.[^37^](#_ENREF_37) Clearance, lipophilicity and distribution model were fixed from the previous step. The intestinal permeability (transcellular) was estimated with reasonable precision: 9.79E-5 +- 4.69E-5 [cm/min] (value +- 95% confidence interval). The predefined “Standard European Male for DDI” individual was used (age=30y, weight=73kg, height=176cm, BMI=23.57kg/m^2^) with CYP2C19 expression.

- - - 1. **Predicting S-omeprazole p.o. profiles following capsules**

A Weibull dissolution model was used to describe the capsule formulation and its parameters were fitted to the data after single dose S-omeprazole 40 mg capsule[^21^](#_ENREF_21) (Table S2.8). The predefined “Standard European Male for DDI” individual was used (age=30 y, weight=73 kg, height=176 cm, BMI=23.57 kg/m^2^) with CYP2C19 expression from PK-Sim database. A lag time of 30 min was used to account for the gastric emptying time. A relatively slow dissolution profile was estimated, with the capsule being 50% dissolved after ~40 minutes. The predictions were able to accurately describe the profile.

Table S2.8 Parameter estimates for capsule formulation based on S-omeprazole p.o. data

| **Identification Parameter** | **95% Confidence Interval** |
| --- | --- |
| Dissolution time (50% dissolved) | 41.65 +- 11.44 [min] |
| Dissolution shape | 1.02 +- 0.23 |

- - - 1. **Predicting R-omeprazole p.o. profiles following oral solution**

As no i.v. data were available for R-omeprazole, the same distribution model and lipophilicity as for S-omeprazole were assumed. For consistency, the same intestinal permeability was also used. As a first step, CYP3A4- and CYP2C19-mediated clearances for R-omeprazole were estimated on the available oral solution data[22](#_ENREF_22)^,^[25](#_ENREF_25) as shown in Table S2.9. Time dependent autoinhibition was derived from literature[^18^](#_ENREF_18) and the predefined “Standard European Male for DDI” individual was used with CYP2C19 expression from PK-Sim database.

Table S2.9 CYP3A4 and CYP2C19 clearance estimates for R-omeprazole based on p.o. data

| **Identification Parameter** | **95% Confidence Interval** |
| --- | --- |
| CYP3A4 specific CL | 0.22 +- 0.04 [1/min] |
| CYP2C19 specific CL | 13.13 +- 1.77 [1/min] |

Note, the estimation weights for R-omeprazole data points after 6 h from dose were set to 0 due to high uncertainty in terminal data obtained from digitization on linear y-scale.

R-omeprazole concentration-time profile following single and multiple q.d. doses in both CYP2C19 EM and PM were well predicted by the model with estimated metabolic clearances.

- - - 1. **Building racemic omeprazole model**

The omeprazole PBPK model was constructed by combining in Mobi the models for the R- and S-enantiomers, assuming that each isomer corresponds to half of the omeprazole dose. The model included both auto inhibition (as derived from Wu[^18^](#_ENREF_18)) as well as mutual inhibition of CYP2C19 pathway (Liu[^38^](#_ENREF_38)). Omeprazole concentrations at any specific time points were obtained by adding the simulated concentrations of two enantiomers together at the corresponding time points to generate the omeprazole PK profiles, according to the following formula:

*fQ_art*(C_pls_art_Eso+C_pls_art_R_O) +fQ_bon*(C_pls_bon_Eso+C_pls_bon_R_O)+fQ_fat*(C_pls_fat_Eso + C_pls_fat_R_O)+fQ_mus*(C_pls_mus_Eso + C_pls_mus_R_O) +fQ_skn*(C_pls_skn_Eso + C_pls_skn_R_O)*

where *fQ_* are fraction of blood flow and *C_pls_* concentrations in plasma compartment respectively in the arterial (*art*), bone (*bon*), fat (*fat*), muscle (*mus*) or skin (*skn*) tissues. *Eso* and *R_O* stand for S- and R-omeprazole respectively.

The plasma concentration in the “Standard European Male for DDI” individual of racemic omeprazole after administration of 10 mg S-omeprazole and 10 mg R-omeprazole as oral solution was well captured both after single and multiple doses. However, the overall clearance was underestimated for (racemic) omeprazole after i.v. administration.

Metabolic clearances were then re-estimated by including also i.v. data of racemic omeprazole. In particular, clearances for S-omeprazole remained very similar to those estimated on S-omeprazole data alone, while R-omeprazole CYP2C19 clearance increased up to 10-fold.

- - - 1. **Adjust CYP2C19 expression in gut**

The implemented relative expression profile of CYP3A4 and CYP2C19 was based on RT-PCR data in the OSP suite human gene expression database. Initially the default reference concentration in the OSP suite given for CYP2C19 was used. However, expression of CYP2C19 isoenzymes in the GI tract significantly prevents R-omeprazole from entering the circulation, and the measured data was under predicted by the model. The relative expression of CYP2C19 in gut was therefore reduced according to Olivares-Morales et al. 2016[^24^](#_ENREF_24) for the final model.

- - - 1. **Refine CYP3A4 mediated metabolism for R-/S-omeprazole on PM data**

To best describe racemic omeprazole data, as much data as possible were included for the parameter optimization.

First, CYP3A4 metabolic clearances for both enantiomers were identified on CYP2C19 PM data where the CYP2C19 pathway was switched off. For this purpose i.v. and p.o. (oral solution) data for either esomeprazole[^25^](#_ENREF_25)^,^[^28^](#_ENREF_28), R-omeprazole[^25^](#_ENREF_25) and omeprazole[^26^](#_ENREF_26)^,^[^27^](#_ENREF_27) were used (Table S2.10). Lipophilicity and intestinal permeabilities were kept from previous steps.

Table S2.10 CYP3A4 clearance estimate for R- and S- omeprazole based on CYP2C19 PM data

| **Identification Parameter** | **95% Confidence Interval** |
| --- | --- |
| CYP3A4 Specific clearance for S-omeprazole | 0.37 +- 0.02 [1/min] |
| CYP3A4 Specific clearance for R-omeprazole | 0.16 +- 0.02 [1/min] |

- - - 1. **Refine CYP2C19 mediated metabolism for R-/S-omeprazole on extensive metabolizers data**

As a next step, CYP2C19 clearance for both enantiomers was optimized on esomeprazole[^21^](#_ENREF_21)^,^[^22^](#_ENREF_22) and omeprazole[^23^](#_ENREF_23)^,^[^26^](#_ENREF_26)^,^[^27^](#_ENREF_27)^,^[^29^](#_ENREF_29)^,^[^30^](#_ENREF_30) i.v. data (Table S2.11).

Table S2.11 CYP2C19 clearance estimate for R- and S- omeprazole based on CYP2C19 EM data after i.v. administration

| **Identification Parameter** | **95% Confidence Interval** |
| --- | --- |
| CYP2C19 Specific clearance for S-omeprazole | 13.98 +- 1.88 [1/min] |
| CYP2C19 Specific clearance for R-omeprazole | 50.00 +- 63.45 [1/min] |

CYP2C19 clearance for S-omeprazole was very similar to the preliminary estimate (11.98 1/min), while that for R-omeprazole hit the upper boundary (> 10-fold). Hence, the estimated range was limited to 50 1/min for this parameter. This value gave a 2C19/3A4 CL ratio similar to the ratio of R-omeprazole intrinsic clearances from recombinant CYPs experiments[^39^](#_ENREF_39). The model describes i.v. data reasonably well in the 10–80 mg dose range, albeit with some overprediction of the 10 mg i.v. infusion and some underprediction for the 40 mg continuous infusion data (graphics not shown).

- - 1. Assumptions and Limitations for the Omeprazole Model

The developed model for R-/S-omeprazole model included the following assumptions:

| **Assumption** | **Justification/impact** |
| --- | --- |
| The same distribution model and lipophilicity was assumed for R-/S-omeprazole. | No i.v. data were available for R-omeprazole. |
| CYP2C19 expression in gut was reduced according to Olivares-Morales et al[^24^](#_ENREF_24) to better describe R-omeprazole. Absorption and metabolic CL were then estimated based on this modified expression. | CYP2C19 CL for R-omeprazole was poorly estimated given the limited amount of R-omeprazole data.  As CYP2C19 CL and expression are obviously inter-dependent, caution should be used when extrapolating such findings to other CYP2C19 substrates. The impact of the reduced expression of CYP2C19 in gut was therefore investigated for its impact on omeprazole levels. |

- - 1. Omeprazole Model Evaluation and Qualification

Simulations of single/multiple p.o. doses in CYP2C19 EM and PM were conducted to visually compare the predicted concentration-time profile to the mean observed concentrations reported in the literature. The model described very well the observed time course in CYP2C19 EM and PM.

Population simulations of single and multiple i.v. or p.o. doses over a wide range of dose levels were conducted to visually compare the predicted concentration-time profile to the observed concentrations reported in the literature, in terms of mean and variability.

The simulated populations matched the race (European or Asian) and the age-weight ranges reported in the respective clinical studies. A total of 1000 individuals were generated for studies in males only, while 2000 were generated for mixed gender populations. The concentration time profile was simulated for each virtual subject and summarized as geometric mean and 95% confidence interval. The simulation was also done for poor CYP2C19 metabolizers. Figure S2.11 shows that the observations were generally within the simulated ranges, both after i.v. and p.o. dosing and for either CYP2C19 EM and PM.

| ***A***  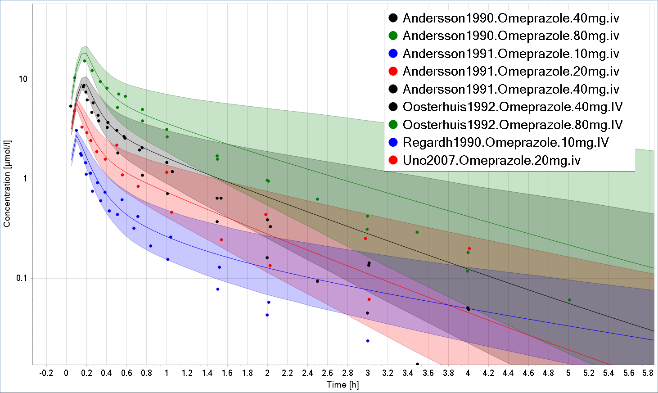 | ***B***  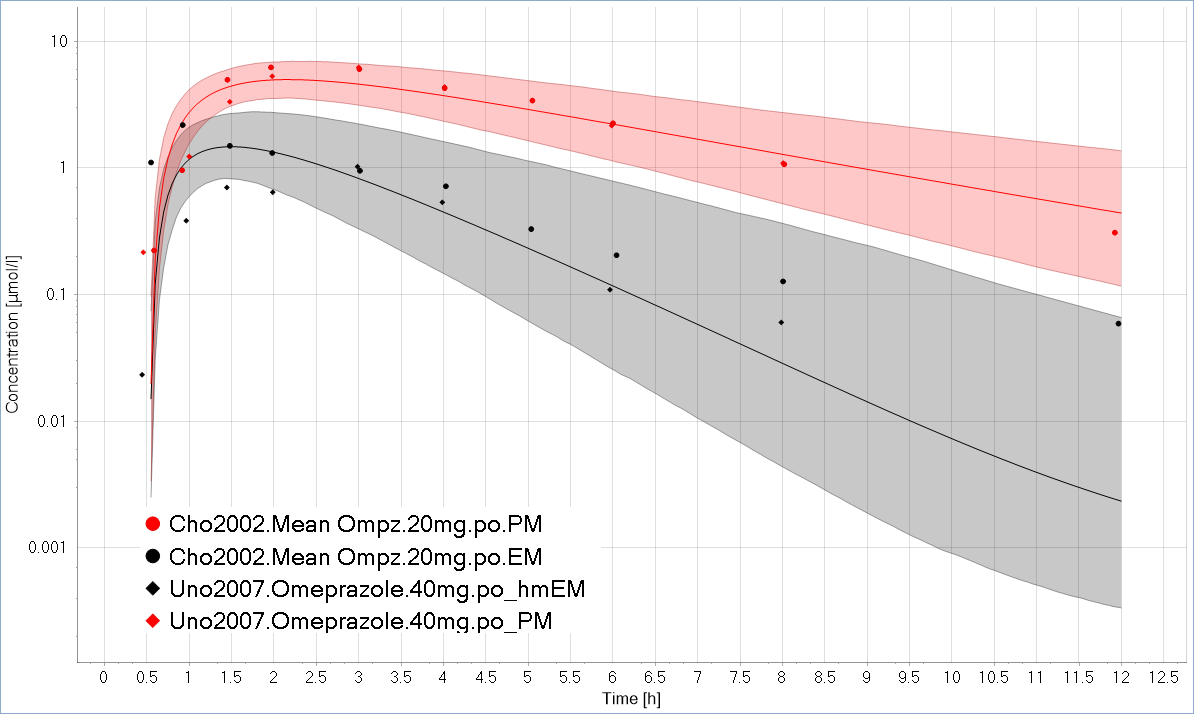 |
| --- | --- |
| ***C***  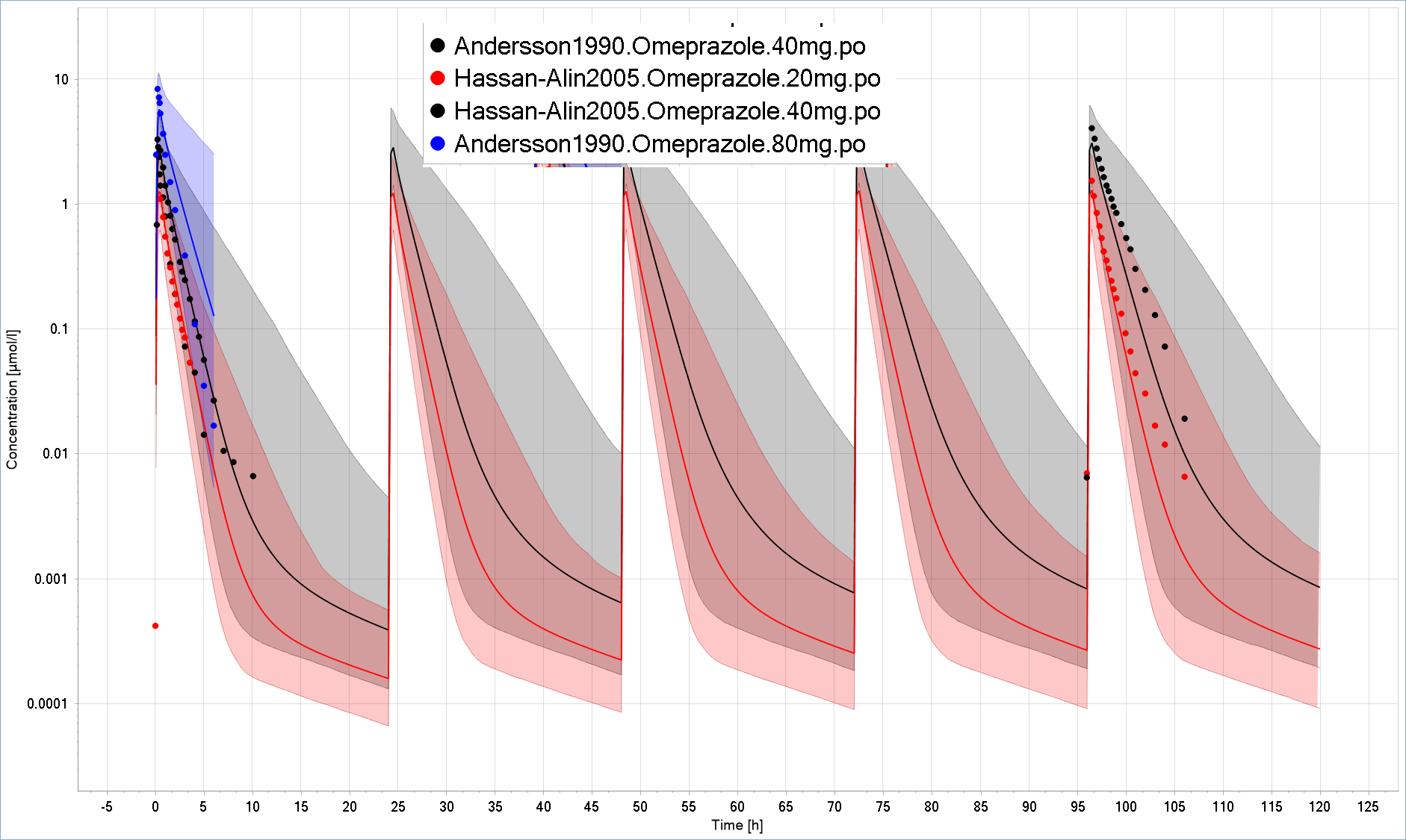 | ***D***  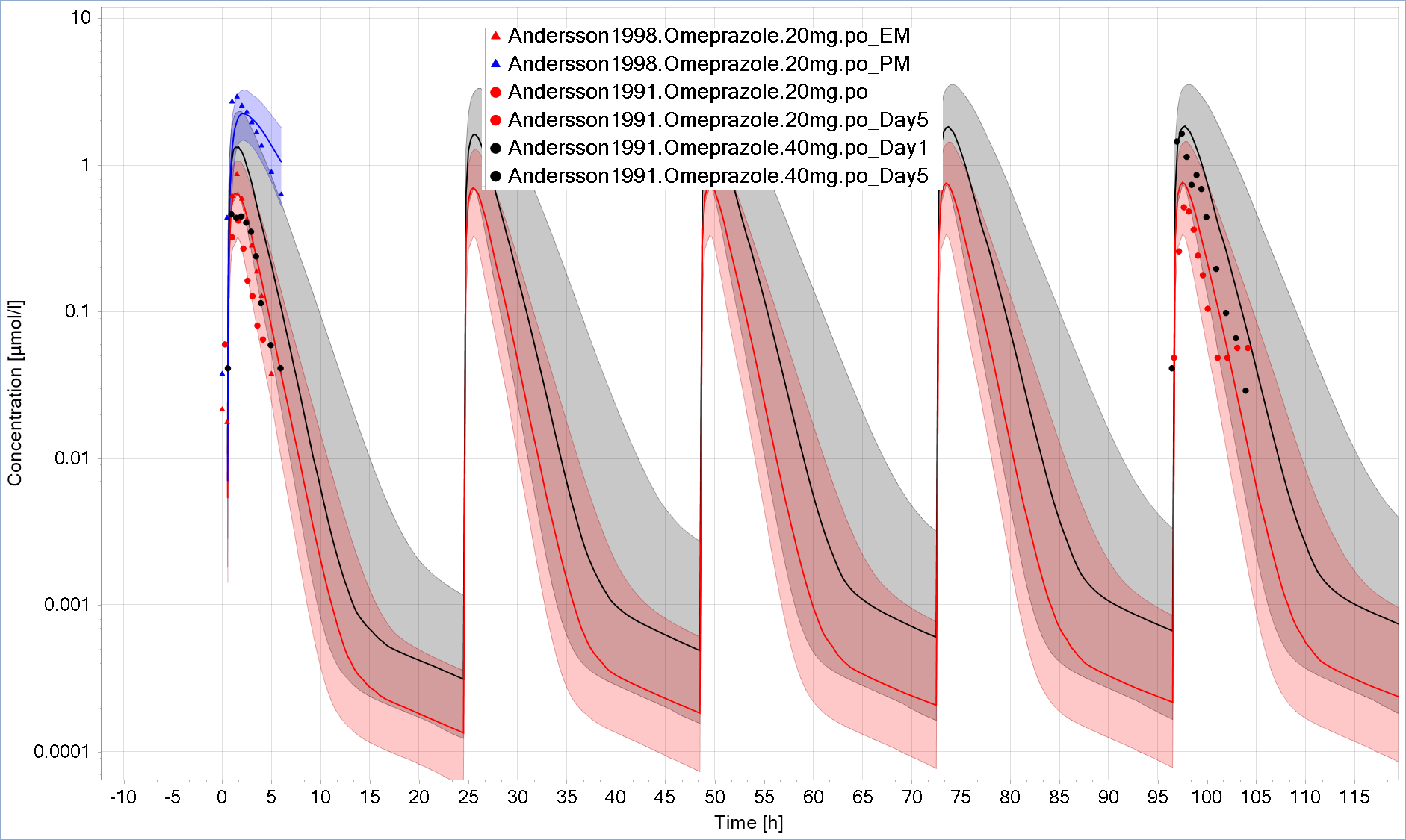 |

Figure S2.11 Population simulations (geometric mean and 95% range) of omeprazole racemate after single i.v. dosing (panel A), single p.o. dosing in CYP2C19 EM and PM (panel B), single and multiple oral doses following solution administration (panel C) or capsule/tablet formulation (panel D).

The final model was further qualified by comparing predicted concentration-time profiles to observed data obtained from two internal clinical studies (NCT01983566, 1220.0059). These data were not used during model development, i.e. they were not used in any parameter estimation procedures.

In study NCT01983566[^40^](#_ENREF_40), 40 mg omeprazole once daily for 4 days was administered to 10 Caucasian and Japanese male/female subjects aged 20 to 35 years, with BMI 18.5 to 25 kg/m^2^. A single PK sample per subject was collected on Day 4 around 4h post dose. In study 1220.0059[^41^](#_ENREF_41), 40 mg omeprazole once daily for 4 days was administered to 15 male/female subjects aged 18 to 50 years, body mass index (BMI) 18.5 to 29.9 kg/m^2^. A full PK profile was collected on Day 5.

A virtual population of N=2000 male/female (50/50%) Caucasian subjects was created in PK-Sim. The population age was ranging 18-50 years and the BMI range was 18.5-29.9 kg/m^2^. Ontogeny and variability of CYP3A4 and CYP2C19 were taken from the PK-Sim database.

Gastrointestinal transit and absorption time of the capsule seem to vary strongly between the individuals and the capsule lag time was adjusted to match individual profiles. The parameters associated to omeprazole capsule administration were the following:

Table S2.12 Parameters associated with omeprazole capsule administration

| **Name** | **Value** | **Value Description** |
| --- | --- | --- |
| Dissolution time (50% dissolved) | 41.65 min | Previously estimated |
| Lag time | 120.00 min | Matched to individual profiles |
| Dissolution shape | 1.02 | Previously estimated |


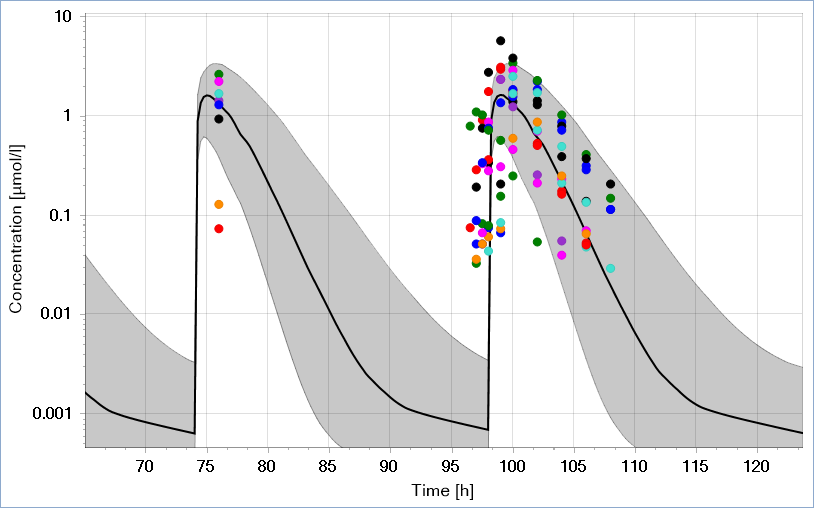


Figure S2.12 Predicted and observed omeprazole concentrations after oral administration in BI internal studies (NCT01983566, 1220.0059).

- - 1. Sensitivity Analysis for Omeprazole Model

The results of the one-way sensitivity analysis with AUC and C_max_ as outcome parameters are shown in Figure S2.13 and Figure S2.14, respectively. The standard set of PK-Sim model parameters visible in simple view (N=203, including e.g. solubility, metabolism by enzyme, organ volumes and blood flows) were investigated.


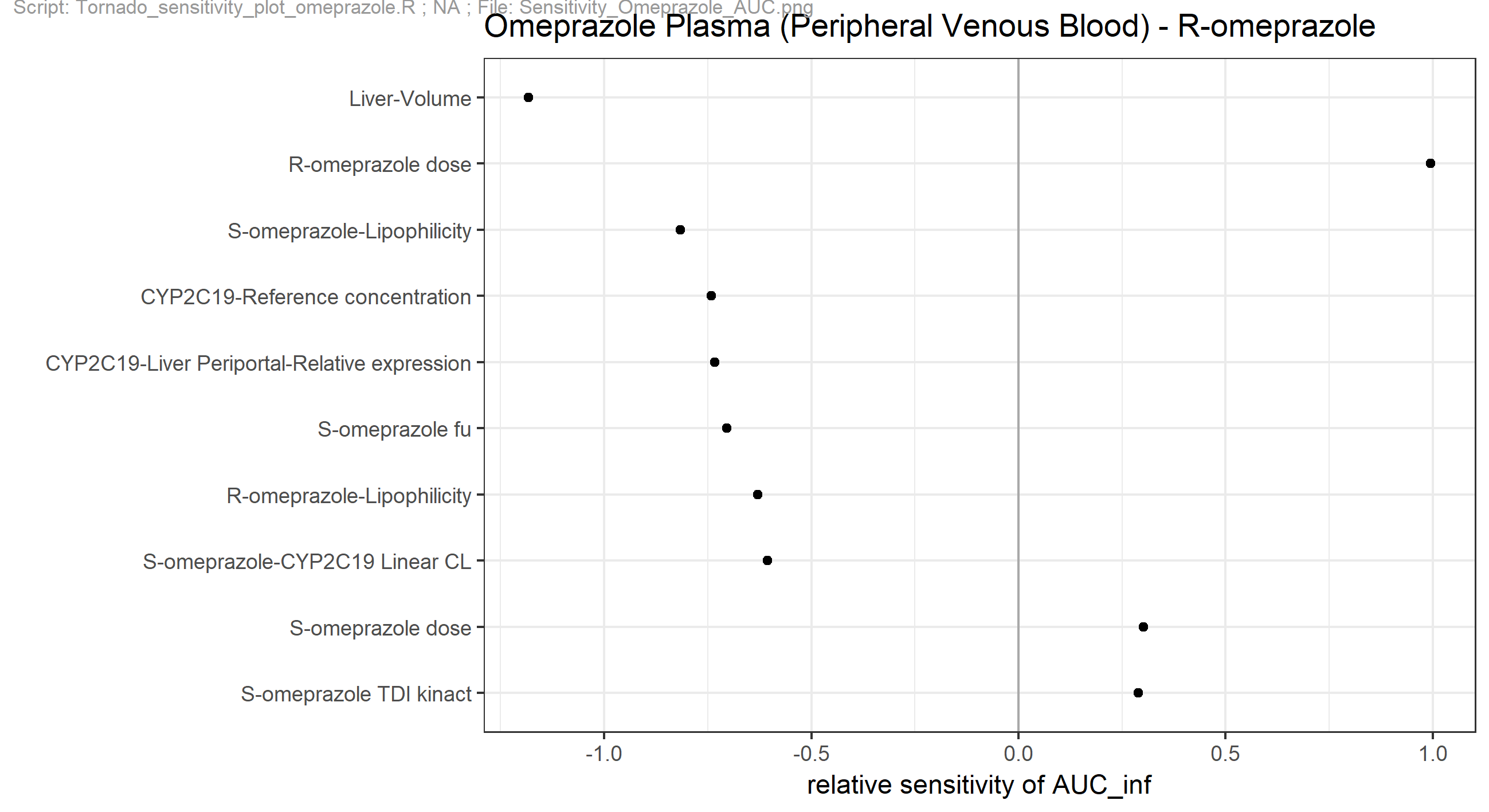


Figure S2.13 Sensitivity Analysis Omeprazole AUC (0 to infinity)


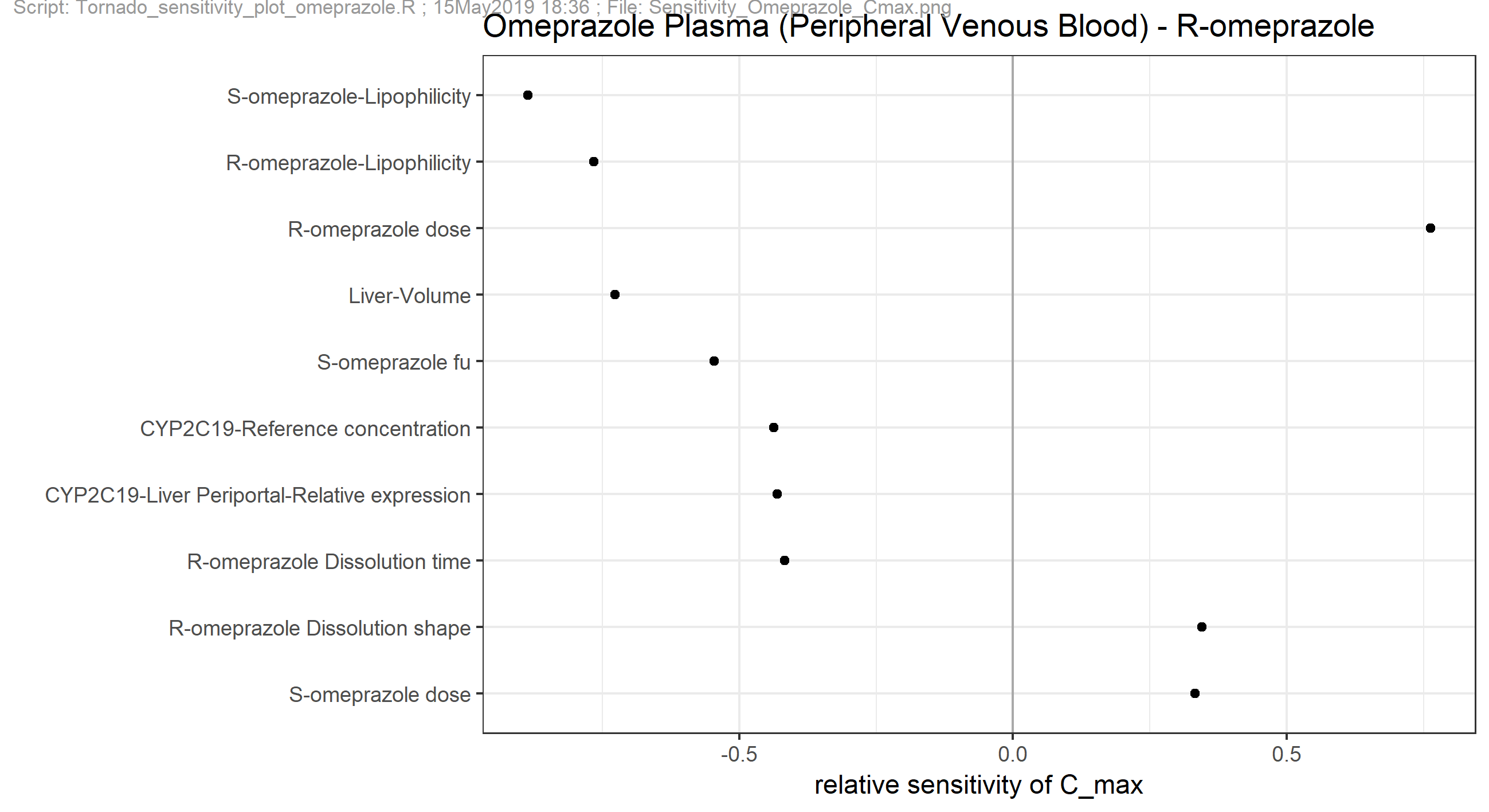


Figure S2.14 Sensitivity Analysis Omeprazole C_max_

Lipophilicity (logP) has a high impact on the PK predictions for omeprazole (mainly on C_max_, but also on AUC). In the current model we estimated logP for S-omeprazole (1.68) and assumed the same for R-omeprazole. Other sources report a very similar value (e.g., DrugBank: 1.66). Therefore, it may be concluded that the lipophilicity used in the PBPK model is unlikely to be substantially different from reality.

The liver volume has the highest impact on AUC. This can be explained by the level of metabolizing enzymes in the liver that directly impacts the total body clearance (and thus the AUC). The value used for the liver volume is a function of body weight in PK-Sim. Given that this is a validated default parameter in PK-Sim, backed by numerous literature sources, substantial bias in this value was not expected.

As can be expected, the dose and the formulation parameters also affect the PK parameters (mainly C_max_). Likewise, parameters relating to CYP2C19 metabolism (reference concentration, intrinsic CL, relative expression in liver periportal) were also very influential on the PK as expected, and TDI Kinact was influential on AUC. Different attempts were made to estimate TDI mechanism with no success and therefore the reported value from Wu et al.[^18^](#_ENREF_18) was used. Note, however, that these parameters are interrelated and that the intrinsic CL was an estimated variable. Hence if the relative expression of CYP enzymes, for example, is changed to a different value, the value of the intrinsic CL would be estimated differently, in order to provide a good fit of the model to the data.

The S-omeprazole fraction unbound was also quite influential. Here the value of 0.03 was used as stated in the label of esomeprazole[^42^](#_ENREF_42). This value was also used in a previous PBPK model using SimCYP^[18](#_ENREF_18)^. No other source was found to confirm this fu value hence it needs to be assumed that the value was unbiased.

In summary, findings from the sensitivity analysis were in line with expectations, i.e. that these parameters influence the predicted AUC and C_max_. Attention should be paid to formulation parameters.

- 1. S-Mephenytoin model
     1. Model Development Strategy

In general, the following step-wise workflow was followed:

1. Development of mean p.o. model (as no i.v. data were available). IVIVE was based on physiochemistry and in vitro metabolization or recalculated in vivo clearance. Alternative middle-out fits to in vivo data were tried but could not improve DDI predictions significantly. Additional limited data led to identifiability problems.
2. Evaluation of p.o. model with virtual PK-Sim population. Range and mean plasma profiles after p.o. administration for the study population were in line with the PK-Sim in-built variability of CYP2C9 and CYP2C19.
3. Qualification with predicted DDI effects. Dynamic DDI predictions with fluvoxamine in range of measured data.

Table S2.13 Model development steps – S-mephenytoin model

| **Step** | **Figure and Table in text** | **Purpose** | **Data** |
| --- | --- | --- | --- |
| **1** |  | IVIVE estimation of CL | Adedoyin 1998[^43^](#_ENREF_43)  Iga 2016[^44^](#_ENREF_44)  Jacqz 1986[^45^](#_ENREF_45)  Wedlund 1985[^46^](#_ENREF_46) |
| **2** | Figure S2.15 | Impact of CYP2C19 expression in gut | Adedoyin 1998[^43^](#_ENREF_43)  Iga 2016[^44^](#_ENREF_44)  Jacqz 1986[^45^](#_ENREF_45)  Wedlund 1985[^46^](#_ENREF_46) |
| **3** |  | DDI predictions | Yao 2003[^47^](#_ENREF_47) |

- - 1. Model Development S-Mephenytoin

Following the classical path of PBPK modelling was not possible[^48^](#_ENREF_48), as no PK data in humans after i.v. dosing and only limited data following p.o. administration to humans were available (See Supplement S1, Table S1.3.2). The predefined “Standard European Male for DDI” individual was used (age=30y, weight=73kg, height=176cm, BMI=23.57kg/m^2^). CYP2C19 expression from the PK-Sim in-built RT-PCR database[^34^](#_ENREF_34)^,^[^35^](#_ENREF_35)^,^[^36^](#_ENREF_36) was added.

IVIVE using CL/F[^43^](#_ENREF_43) recalculated to an intrinsic CYP2C19 clearance and vitro metabolism data from human microsomes CYP2C19 clearance [130] was successful in describing the limited human p.o. data. Measured data were used for lipophilicity[^49^](#_ENREF_49), solubility[^50^](#_ENREF_50) and f_u_ in plasma[^51^](#_ENREF_51). Fraction of GFR was set to 1.

Efforts were made to improve the description of data by fitting lipophilicity, CYP2C19 intrinsic clearance and intestinal permeability. However, the results did not improve the description of data and the parameters were not identifiable. Hence, IVIVE was used in the final model to describe S-mephenytoin pharmacokinetics. The resulting model adequately predicted the limited set of data (Figure S2.15).

- - - 1. **Impact of CYP2C19 expression in gut**

As S-mephenytoin is metabolized via CYP2C19, the impact of reducing CYP2C19 relative expression in gut as in [Section 2.2.2.7](#sec3547) was investigated. This led to an AUC increase of about 30%, with still good predictions of S-mephenytoin data based on IVIVE approach (Figure S2.15). Given the moderate impact overall, the predefined CYP2C19 expression in gut was used which is in line with the other templates of the network (except for omeprazole). The effect of CYP2C19 expression in gut on DDI predictions was also investigated and is discussed in other sections.


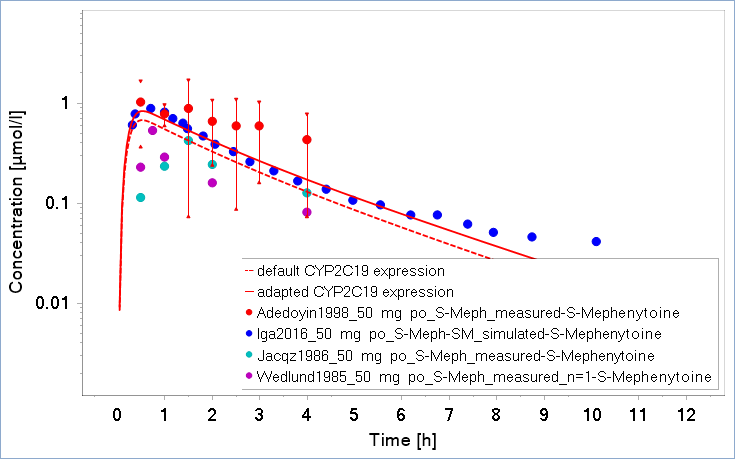


Figure S2.15 Simulation of 50 mg oral S-mephenytoin based on IVIVE approach with default and reduced CYP2C19 expression in gut.

- - 1. Assumptions and Limitations for the S-Mephenytoin Model

The developed model for S-Mephenytoin included the following assumptions:

| **Assumption** | **Justification/impact** |
| --- | --- |
| S-Mephenytoin is mainly metabolised by CYP2C19 | Total intrinsic clearance of S-mephenytoin varies 80 to 100-fold between CYP2C19 EM and PM[^45^](#_ENREF_45). |
| Renal elimination accounts only for a minor part of total clearance and is driven by passive glomerular filtration | No active renal excretion described in literature. Renal clearance is minor compared to overall plasma clearance[^45^](#_ENREF_45) and can be described by GFR in PK-Sim. |
| Intestinal permeability can be calculated by PK-Sim based on MW and lipophilicity | No in vitro measured values for intestinal permeability available. |
| Interindividual variability of clearance is caused by variability of CYP2C19 expression. This variability is larger than PK-Sim default variability | Variability in data depends on clearance via CYP2C19 as studies with CYP2C19 PM and non_PM show interstudy and intrastudy^[45](#_ENREF_45)^ variabilities are higher than expected with ontogeny function. |

- - 1. S-Mephenytoin Model Evaluation and Qualification

A simulation was carried out in a population with 2000 virtual individuals according to the biometrics of the individuals (8 males and females, 32–78 years) used in a study by Adedoyin et al 1998[^43^](#_ENREF_43). Additional variability was included on CYP2C19 among the population, by using the geometric SD as derived from reported CL/F values by Olivares-Morales et al.[^24^](#_ENREF_24). Population simulated data had a comparable variability with the observed data. Moreover, the mean data from other studies[^45^](#_ENREF_45)^,^[^46^](#_ENREF_46) were in the range of expected variability, confirming the adequacy of the model (Figure S2.16).


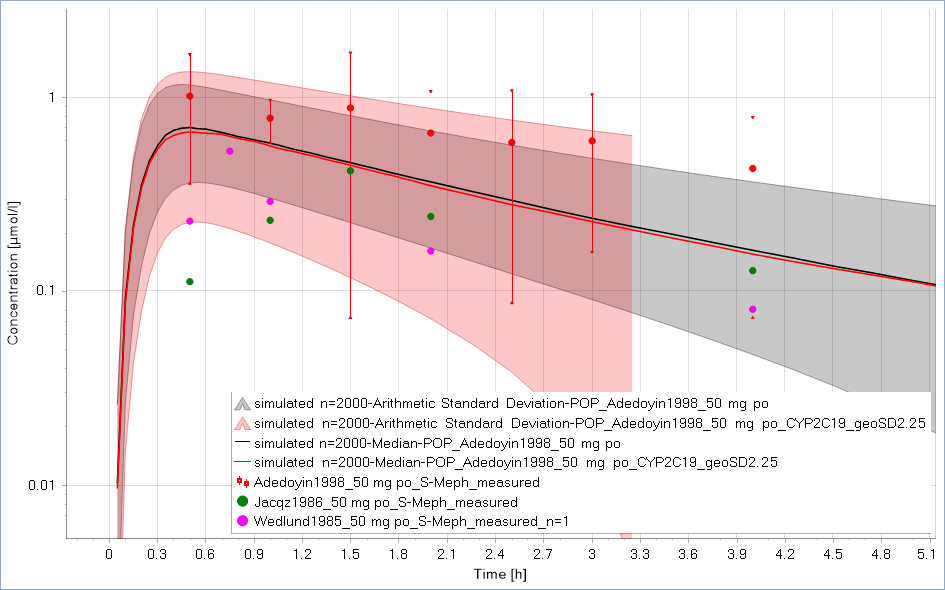


Figure S2.16 Simulated S-Mephenytoin Plasma profiles in 2000 healthy individuals after a 50 mg po dose.

*The black and red lines are the median concentration from 2000 simulated non-PM individuals with biometrics according to a study by Adedoyin et al 1998. The shaded areas encompass +-SD range of the respective concentrations with default variability (grey) and geoSD from Olivares-Morales et al.*[^24^](#_ENREF_24) *(red). The red symbols with error bars are mean and SD data from Adedoyin et al 1997. The green and purple circles are mean data from two other studies found in literature.*

- - 1. Sensitivity Analysis for S-Mephenytoin Model

For the final S-mephenytoin model a sensitivity analysis was run in PK-Sim. The standard set of PK-Sim model parameters visible in simple view (N= 219, e.g. metabolism by enzyme, organ volumes and blood flows) were investigated. The effects of changing a certain parameter value on the relative sensitivity coefficients of AUC (Figure S2.17) and C_max_ (Figure S2.18) are shown.


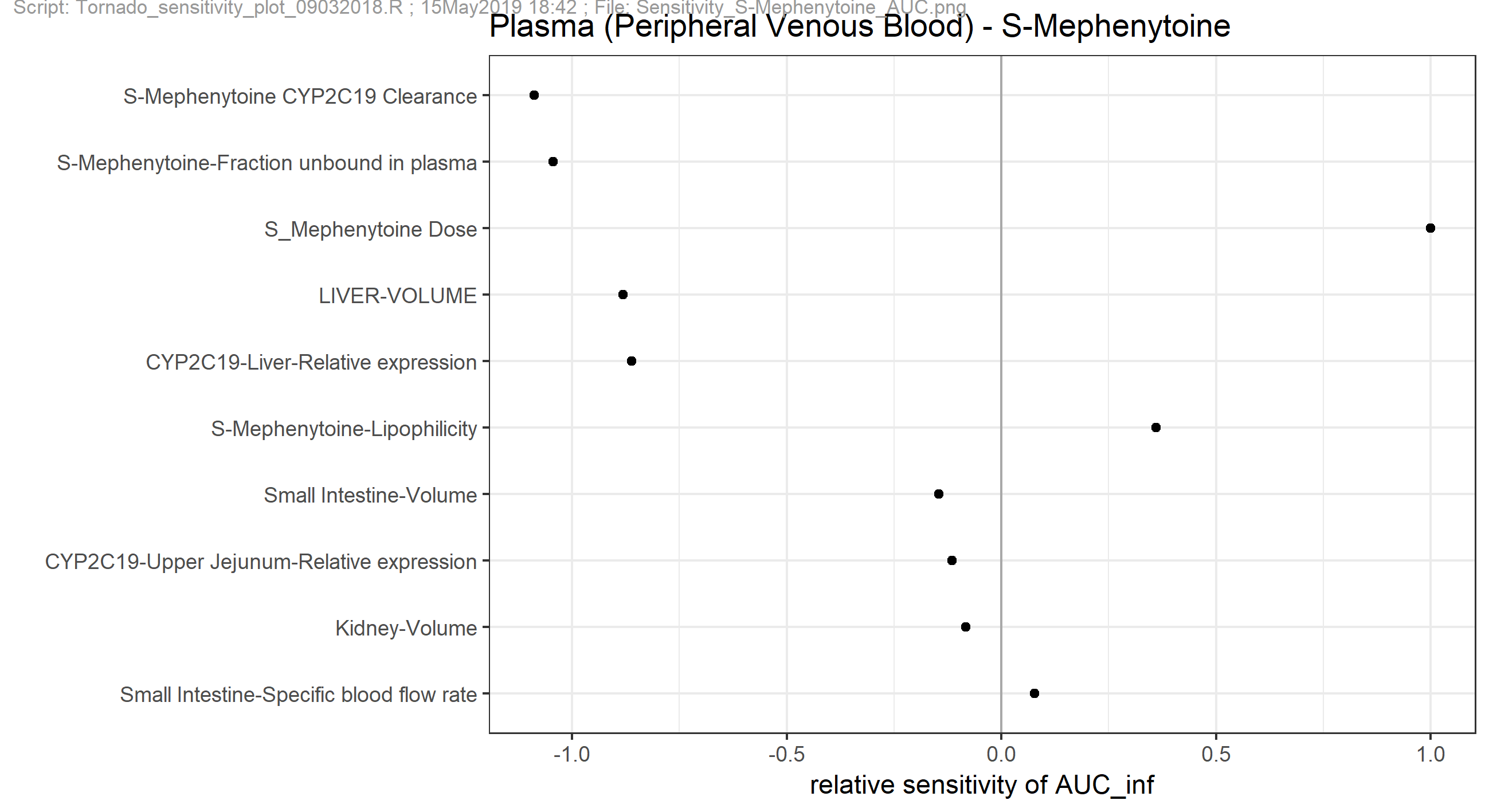


Figure S2.17 Sensitivity Analysis S-Mephenytoin AUC (0 to infinity)


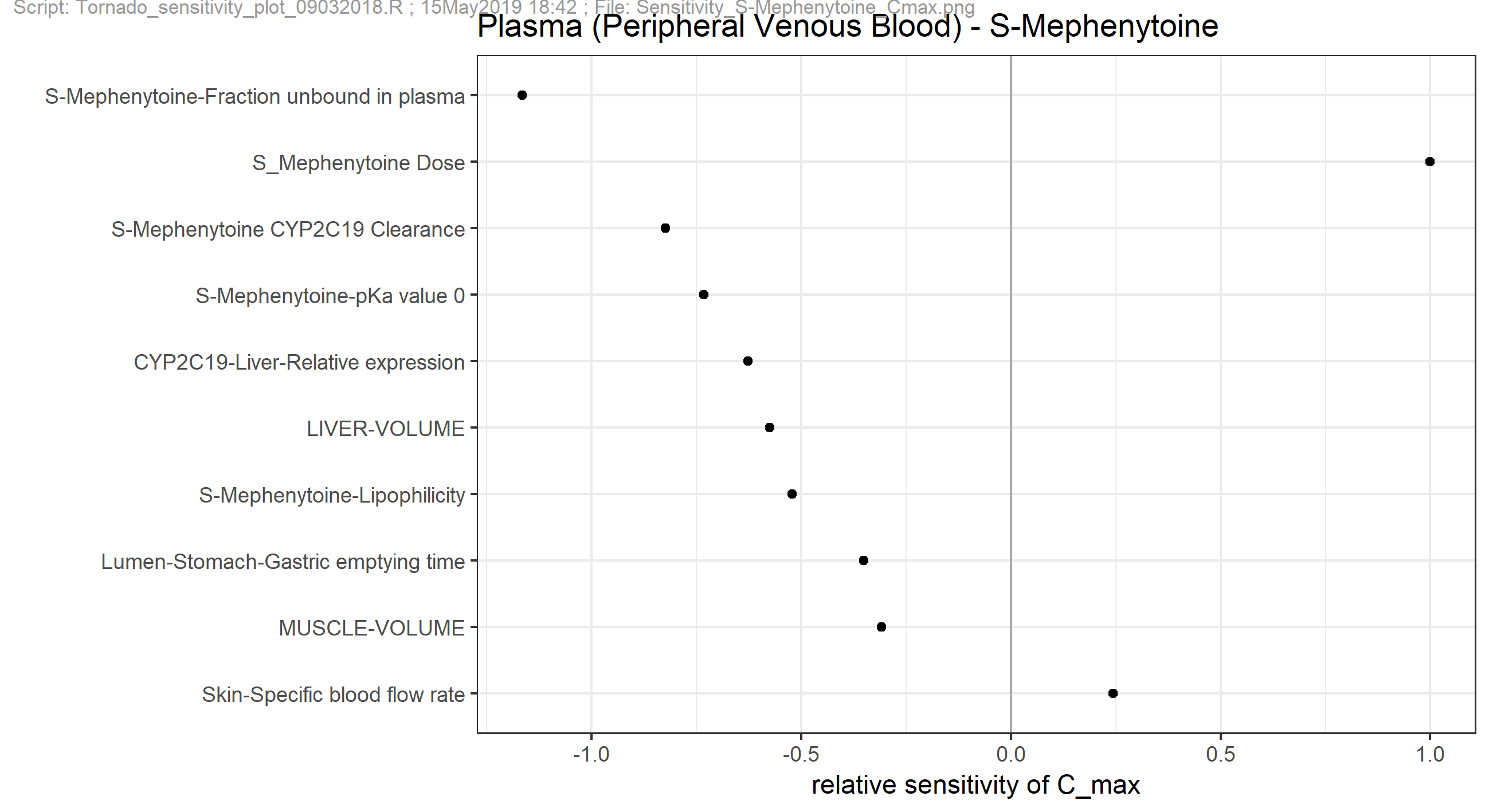


Figure S2.18 Sensitivity Analysis S-Mephenytoin C_max_

CYP2C19 clearance was found to impact both AUC and C_max_, together with CYP2C19 concentration in the liver. This finding was in line with expectations, i.e. that these parameters influence the predicted AUC and C_max_. The intrinsic clearance for CYP2C19 (1986.9 ml/min) was obtained from CL/F in Adedoyin et al.[^43^](#_ENREF_43) and corresponds to a specific clearance of 1.26 min^-1^. The calculated specific clearances based on other in vitro sources lead to similar values, from 0.22 to 2.64 min^-1^.[^44^](#_ENREF_44)

The relative expression of CYP2C19 in upper jejunum was found to influence AUC. The impact of reduced CYP2C19 expression in gut was investigated and showed a slightly higher AUC for adapted CYP2C19 in gut. DDI predictions with the reduced CYP2C19 expression in gut were similar in terms of C_max_ (where we have clinical data), and slightly higher in terms of AUC. Overall, given that this effect was moderate, the standard expression in gut was used. However, the recommendation is to investigate the impact of CYP2C19 expression in gut through sensitivity analysis when CYP2C19 substrates are modelled.

Fraction unbound has a high impact on S-mephenytoin PK, as it determines the fraction of compound available for metabolism. The f_u_ value used in the model was obtained from experimental measurement[^51^](#_ENREF_51), but no other sources were available to compare this value.

Lipophilicity also impacts on AUC. In the current model we used experimental logP (1.69[^49^](#_ENREF_49)); Calculated logP values from ALOGPS and ChemAxon are very close (1.64–1.67). Therefore, it may be concluded that the lipophilicity used in the PBPK model is unlikely to be substantially different from reality.

The liver volume in PK-Sim determines the level of metabolizing enzymes and hence directly impacts the total body clearance (and thus the AUC). The value used for the liver volume is a function of body weight in PK-Sim and sourced back to various literature sources. Organ volumes and blood flow rates are also influential on PK. These are validated default parameter in PK-Sim backed by numerous literature sources and substantial bias in these values are not expected.

For C_max_, the dose has most influence. Given that s-mephenytoin is a weakly acidic drug with a pK_a_ of 8.51 (DrugBank), solubility is pH-dependent, and hence the reference pH at which the aqueous solubility was determined impacts the absorption (speed) and hence C_max_. However, devices to determine the pH in an aqueous medium are considered accurate and the reported values can be considered invariant.

In summary, findings from the sensitivity analysis were in line with expectations, i.e. that these parameters influence the predicted AUC and C_max_.

- 1. Moclobemide model
     1. Model Development Strategy

A step-wise workflow was generally followed consisting of the following key steps:

1. Fit total CL as a placeholder using single dose i.v. data to select the appropriate distribution (i.e. partition coefficient) model.
2. Estimate the contribution of non-CYP2C19 mediated metabolism using data (p.o.) from CYP2C19 PM. This pathway was assumed to be mainly attributed to flavin-containing monooxygenase (FMO). It should be noticed that this includes any other unspecific CL.
3. Use single-dose data (i.v. and p.o.) to estimate V_max_ and K_m_ of CYP2C19.
4. Predict concentrations after multiple oral dosing and compare to literature. Steady state levels were not adequately predicted, as will be shown in the following sections and the model was refined. Adapt model, including time-dependent auto-inhibition to account for a change in CL over time.
5. Predict single and multiple doses profiles (both i.v. and p.o.) with the updated model and compare to published profiles. Qualify model by comparing predicted CL/F and C_max_ to the corresponding parameters in a review across multiple studies. Population prediction to verify the variability components of the model.
6. Prediction of drug-drug interaction with omeprazole as inhibitor.

Initially, attempts were made to also unravel the contribution of the FMO3-specific clearance pathway and the unspecific pathway using the in vitro FMO-CL of moclobemide in a microsomal assay reported by Hoskin et al.[^52^](#_ENREF_52) However, this route was abandoned as predictions were not in line with observations, potentially requiring the need for an in vivo-in vitro scaling factor. For the purpose of DDI predictions, the details of the CYP2C19 pathway only were considered relevant.

Table S2.14 Model development steps – moclobemide model

| **Step** | **Figure and Table in text** | **Purpose** | **Data** |
| --- | --- | --- | --- |
| **1** |  | Selection of Distribution model | Schoerlin 1987 i.v.^[53](#_ENREF_53)^  Raaflaub 1984 i.v.^[54](#_ENREF_54)^ |
| **2** |  | Estimate non-CYP2C19 metabolism | Gram 1995 PM^[55](#_ENREF_55)^  Yu 2001 PM^[56](#_ENREF_56)^ |
| **3** | Table S2.15 | Estimate CYP2C19 metabolism | Gram 1995 EM^[55](#_ENREF_55)^  Yu 2001 EM^[56](#_ENREF_56)^  Wiesel 1985^[57](#_ENREF_57)^  50, 100 and 200 mg  Schoerlin 1987 i.v.^[53](#_ENREF_53)^  Guentert 1990^[58](#_ENREF_58)^  Raaflaub 1984^[54](#_ENREF_54)^ p.o and i.v. |
| **4** | Figure S2.19 | Multiple dose prediction with autoinhibition | Ignjatovic 2009[^59^](#_ENREF_59) |
| **5** | Figure S2.20 | Model evaluation | Gram 1995^[55](#_ENREF_55)^  Wiesel 1985^[57](#_ENREF_57)^  Raaflaub 1984^[54](#_ENREF_54)^ |
| **6** |  | DDI predictions | Yu 2001^[56](#_ENREF_56)^ |

- - 1. Model Development Moclobemide
       1. **Selecting a distribution model using i.v. data only**

To decide between the models described above, the total hepatic clearance was fixed to the value of 0.48 L/h/kg as reported by Schoerlin and the total error of the fit of the various distribution models was evaluated using the i.v. data from Raaflaub (50 mg) and Schoerlin (150 mg). The renal clearance was fixed to a value of 0.034 ml/min/kg as derived from Schoerlin et al.[^53^](#_ENREF_53)

To model these data, a typical European male subject (age=30 y, weight =73 kg, height = 176 cm, BMI=23.57 kg/m^2^) was created in PKsim using the predefined database “European (ICRP, 2002)”, by adding CYP2C19 and FMO (other) expression from the PK-Sim RT-PCR database.

The model according to Rodgers & Rowland and Berezhkovskiy resulted in a similar total error and very similar predictions of the concentration-time profile. The Rodgers & Rowland model was selected as this is the model most commonly used in PBPK modelling.

- - - 1. **Estimate non-CYP2C19 mediated metabolism**

PM data were used to estimate the intrinsic ‘non-CYP2C19’ clearance (occurring in the liver only). The variable in the model was called FMO, as this most likely constitutes the major non-CYP2C19 mediated metabolism, but also covers other non-specified metabolic routes.

The intrinsic CL was estimated based on PM data from Yu et al.[^56^](#_ENREF_56) and Gram et al.[^55^](#_ENREF_55) both after single and multiple oral tablet dosing (default thickness of the water layer and default particle radius). To replicate the time profiles from Yu et al. an Asian typical individual was chosen as the study was carried out in South Korea. For Gram et al. (and all other simulations unless specified otherwise) the standard European male subject was the basis for the simulation. To fit the data from Yu et al.[^56^](#_ENREF_56), a typical Asian subject (age=30 y, weight=60.03 kg, height=170 cm, BMI=20.78 kg/m^2^) was created from the predefined database “Asian (Tanaka, 1996)” by adding CYP2C19 and FMO (other) expression from the PK-Sim RT-PCR database. The observed data from PM were well captured by the model. The intrinsic CL (FMO/unspecific) was estimated to be 0.165 +/- 0.03 L/min.

- - - 1. **Estimate V_max_ and Km of CYP2C19 metabolism using single dose data (i.v. and p.o.)**

The literature clearly indicates a more than proportional increase in plasma concentrations with increasing dose, suggesting a non-linear clearance mechanism. Using the model from the previous step (i.e. with the FMO clearance estimate fixed) and only estimating the parameters of the CYP2C19 clearance the parameter estimates shown in Table S2.15 were obtained. The alternative model with a linear elimination mechanism was also explored. The linear CL model however, despite a more precise estimate of the CL, resulted in a higher total error (3.99 vs. 3.62) and, more importantly, the predicted curves were further from the observations across the dose range. For this reason, the less precise but more plausible non-linear model was selected for further development.

Table S2.15 Parameter estimates of the CYP2C19 clearance pathway for moclobemide

| **Identification Parameter** | **95% Confidence Interval** |
| --- | --- |
| V_max__2C19 | 2.025 +- 1.16 [µmol/min/kg tissue] |
| Km_2C19 | 1.11 +- 1.10 [µmol/l] |
| *Alternative model:* |  |
| CLint_2C19 | 1.43 +- 0.19 [l/min] |

- - - 1. **Prediction of concentration-time profiles following multiple oral dosing and model adaptation with time-dependent autoinhibition**

The model with the parameter estimates from the previous step was used to predict the concentrations of moclobemide at steady state following multiple oral dosing. To fit the data from Ignatovic et al.[^59^](#_ENREF_59), a typical European female subject (age=30 y, weight = 64 kg, height = 163 cm, BMI=24.09 kg/m^2^) was created from the predefined database “European (ICRP, 2002)” by adding CYP2C19 and FMO (other) expression from the PK-Sim RT-PCR database.

Concentrations at steady state were underpredicted, and as the fit of single dose (or day 1) data was good, the misprediction after repeated dosing indicates some sort of time-dependent elimination. The addition of an inhibitory metabolite may be an explanation (see results and discussion). However, it would be very challenging to incorporate the kinetics of such a metabolite in the existing model, considering that no data on IC_50_ or K_i_ are available for such a metabolite. Therefore, the time-dependency was accounted for by simply including a time-dependent autoinhibition function on the CYP2C19 enzyme system.

Time-dependent autoinhibition was included on the CYP2C19 pathway. The mechanism is described in PK-Sim with two parameters: Kinact and Kinact__half._ Given the available data, both parameters could not be estimated together (not separately identifiable). Assuming Kinact is enzyme- but not substance-specific, Kinact was fixed to the value reported by Wu et al. for omeprazole[^18^](#_ENREF_18) and only estimate Kinact__half_. The estimate and the associated confidence interval are shown in Table S2.16:

Table S2.16 Estimated Kinact and Kinact, t_1/2_ for TDI in moclobemide model

| **Identification Parameter** | **95% Confidence Interval** |
| --- | --- |
| Kinact__half_ | 94.85 +- 17.56 [µmol/l] |
| Kinact | 5 1/h (fixed) |

The simulations of the profiles at steady state were repeated and showed a much better agreement of the predictions and observations (Figure S2.19).

| 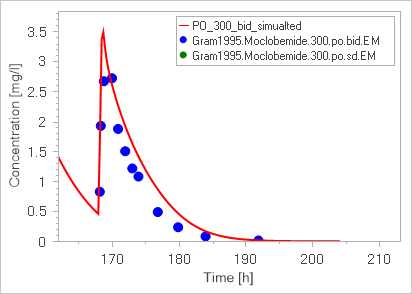 | 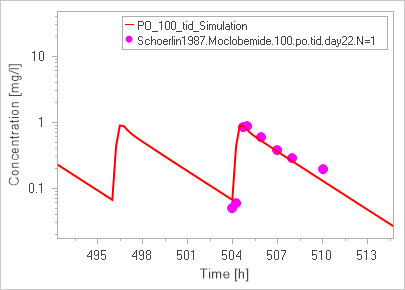 |
| --- | --- |
| 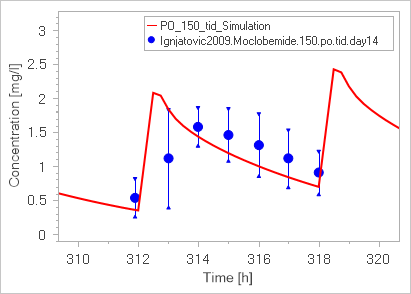 | 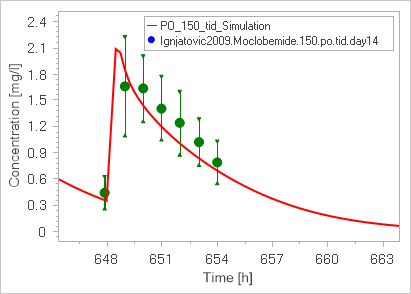 |

Figure S2.19 Prediction of moclobemide concentration at steady state following multiple dosing (b.i.d or t.i.d.) of various dose levels with the model accounting for time-dependent autoinhibition.

- - - 1. **Impact of CYP2C19 expression in gut**

As moclobemide is metabolized via CYP2C19, the impact of reducing CYP2C19 relative expression in gut as in [Section 2.2.2.7](#sec3547) was tested. For this purpose, single and multiple 300 mg p.o. doses were simulated with the standard PK-Sim expression levels and the adapted one. The resulting change in moclobemide plasma profile was minimal, especially around C_max_. Hence, the default expression was used in subsequent evaluation.

- - 1. Assumptions and Limitations for the Moclobemide Model

The developed model for moclobemide included the following key assumptions:

| Assumption | Justification/impact |
| --- | --- |
| Non-CYP2C19 CL occurring in the liver only. | Most of the non-CYP2C19 metabolism is via FMO3 which is mainly expressed in the liver. |
| Time-dependent CL due to autoinhibition of CYP2C19 | Not enough data to evaluate inhibition of emerging moclobemide metabolites. Auto-inhibition of moclobemide appears appropriate approximation. |
| Non-linear CYP2C19 clearance | Data from literature show dose non-proportionality |

- - 1. Moclobemide Model Evaluation and Qualification

Using observed data that were not used for model building[^60^](#_ENREF_60) in combination with some data used for model building (see Supplement 1, Table S1.4.3 for details), simulations of single i.v. or p.o. doses over a wide range of dose levels were conducted to visually compare the predicted concentration-time profiles to the mean observed concentrations reported in the literature. As can be seen in Figure S2.20 the model describes the observed concentration-time courses well.

| 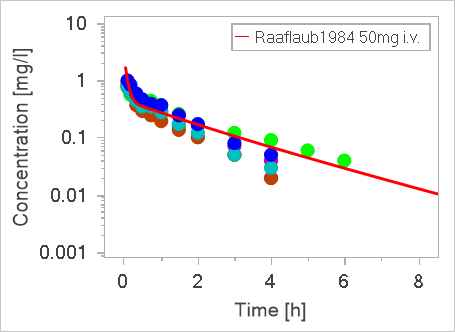 | 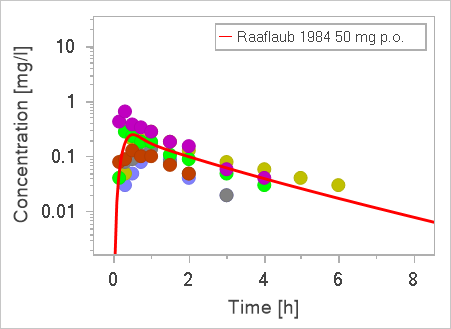 |
| --- | --- |
| 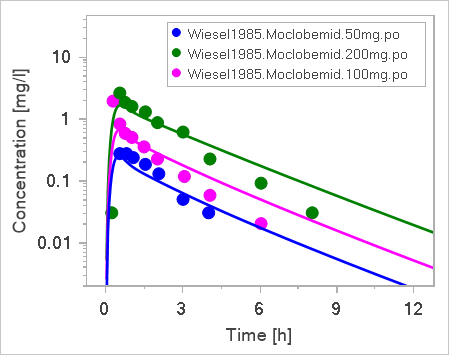 | 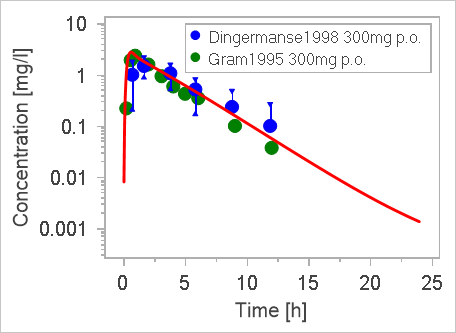 |

Figure S2.20 Simulation of single doses of moclobemide administered i.v. or p.o. over a wide range of doses.

In addition to these simulations for a typical individual, population simulations were carried out to evaluate if the variability incorporated in the model matches the literature reports.

A population of 2000 Asian subjects with age and weight in the same range as reported by Yu et al.[^56^](#_ENREF_56) (age: 20-36 y, weight: 40-120 kg, 13% female) was generated, and the concentration time profile following a single dose of 300 mg p.o. was simulated for each virtual subject and summarized as mean +/- SD. The simulation was also done for poor CYP2C19 metabolizers.

Figure S2.21 shows that the width of the SD band corresponds well with the width of the error bars digitized from Yu et al.[^56^](#_ENREF_56), both for EM and PM.

| *Poor metabolizers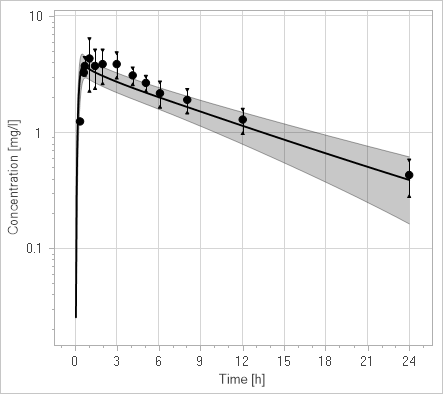* | *Extensive metabolizers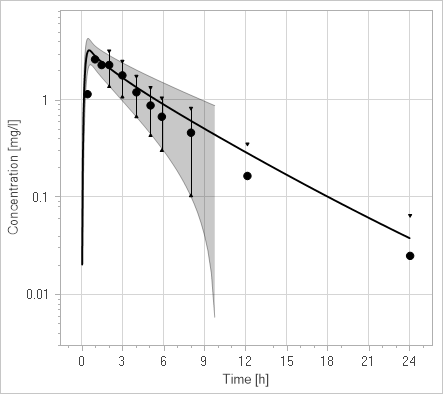* |
| --- | --- |

Figure S2.21 Population simulation for a single p.o. dose of moclobemide (300 mg) administered to Asian subjects who are either poor or extensive metabolizers.

*Circles and bars: observed mean concentration +/- SD as digitized from Yu et al*[^56^](#_ENREF_56)*. Solid line and grey area: mean and SD range of predictions.*

The review paper from Mayersohn et al.[^61^](#_ENREF_61) provides data on mean CL/F and C_max_ (weighted by number of subjects) over a wide range of dose levels and studies. Several population simulations were conducted (N=1000, European subjects, age: 20-50 y, BMI: 18-27 (kg/m^2^), 50% females), one for each dose level, and derived the CL/F and C_max_ from the simulated profiles for the sake of comparison. The observed mean parameters were well in line with the simulations.

- - 1. Sensitivity Analysis for Moclobemide Model

The results of the one-way sensitivity analysis with AUC and C_max_ as outcome parameters are shown in Figure S2.22 and Figure S2.23, respectively. The standard set of PK-Sim model parameters visible in simple view (N=854, including e.g. solubility, metabolism by enzyme, organ volumes and blood flows) were investigated.


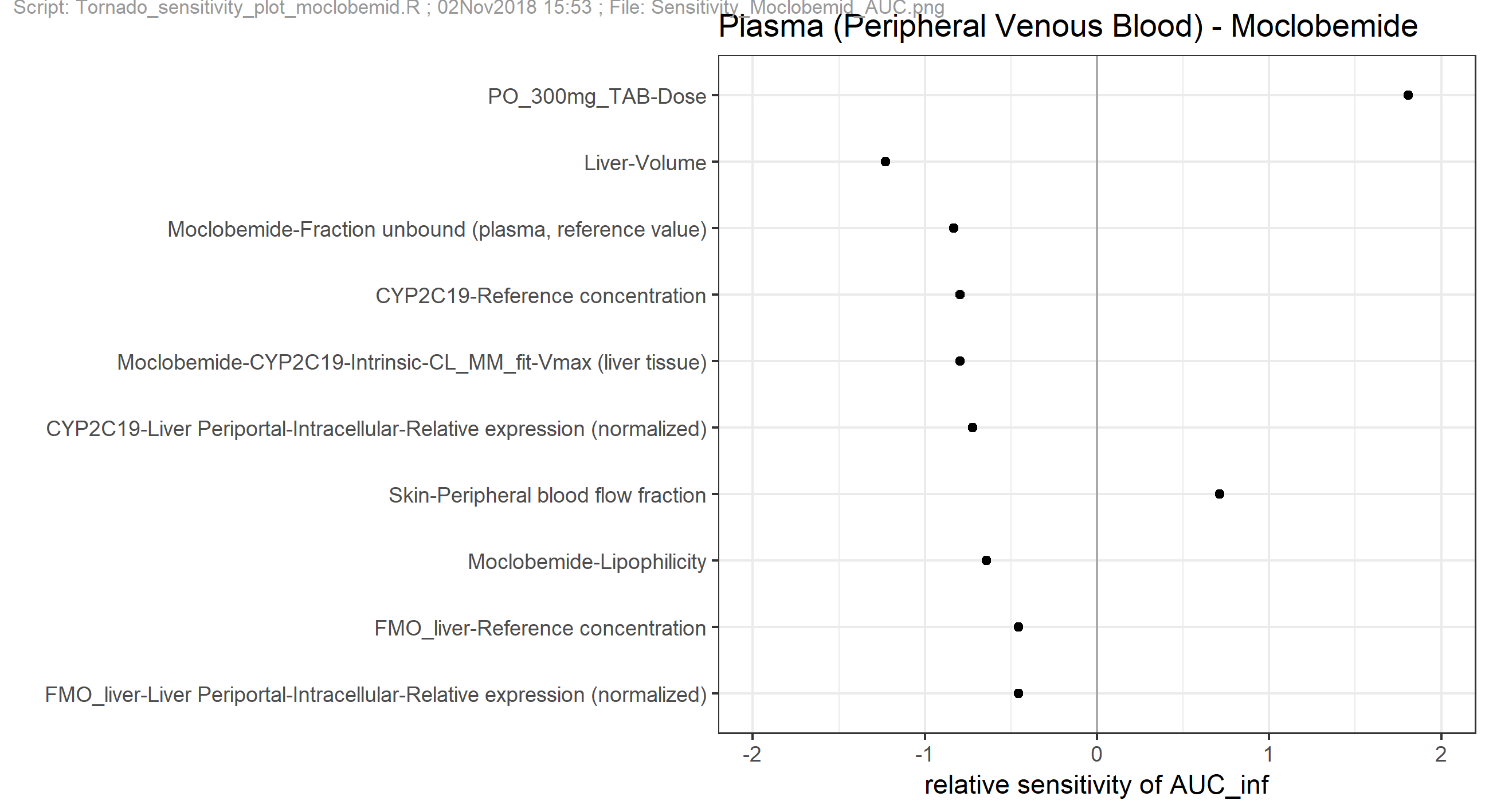


Figure S2.22 Sensitivity Moclobemide AUC


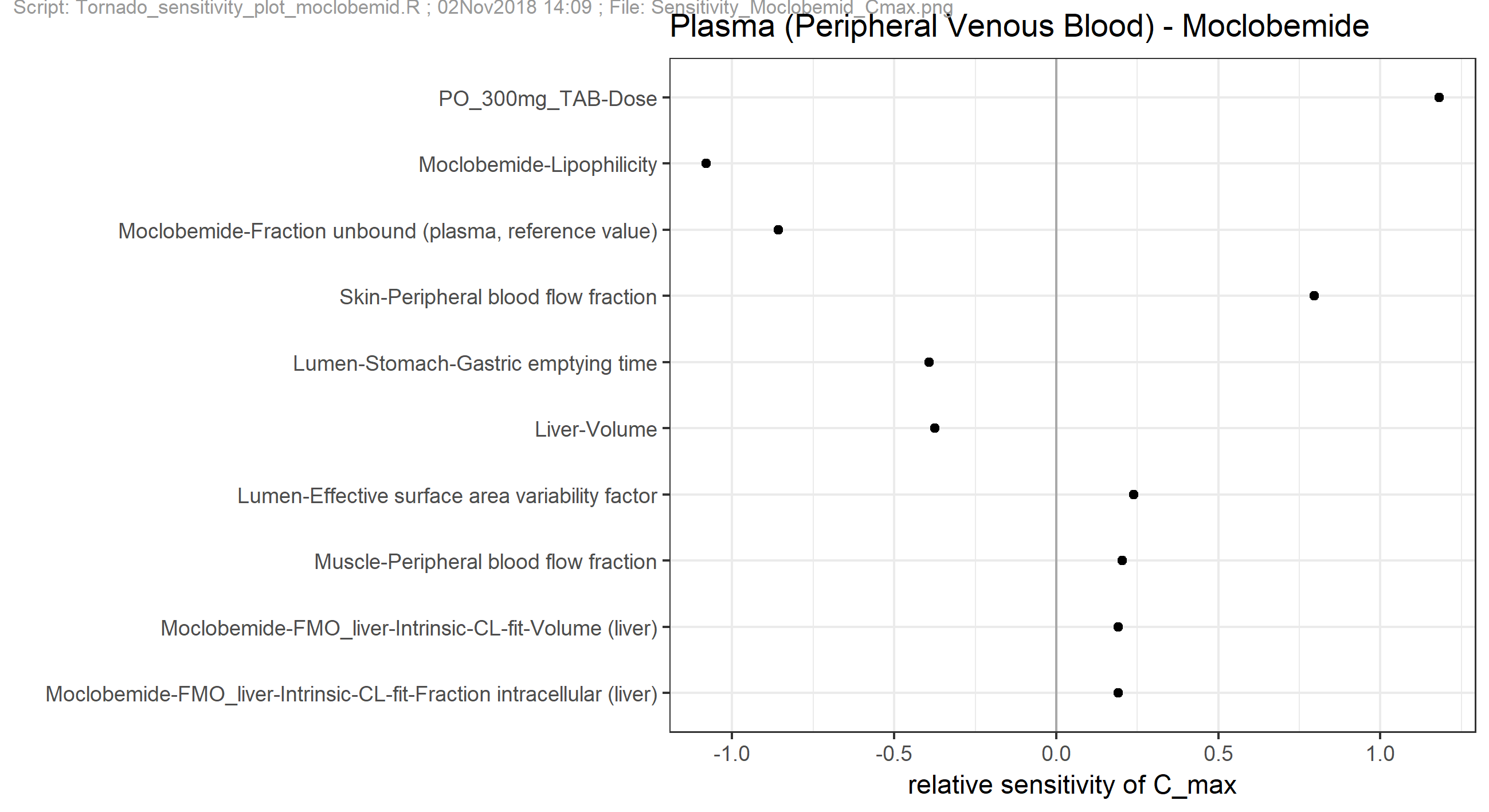


Figure S2.23 Sensitivity Moclobemide C_max_

Due to the non-linear elimination of moclobemide, the dose has an overproportional influence on the PK (relative sensitivity >1).

Liver volume has a high impact on the AUC. This is understandable since moclobemide is almost completely metabolised in the liver and the intrinsic CL value is directly proportional to the organ volume in PK-Sim. Given that this is a validated default parameter in PK-Sim backed by numerous literature sources, substantial bias in this value was not expected. Likewise, the parameters relating to metabolism (CYP2C19/FMO reference concentration, V_max_, intrinsic CL, relative expression cellular volume and fraction) are also very influential on the PK as expected. These parameters are interrelated by definition and that the intrinsic CL and V_max_ were estimated variables. Hence, if the relative expression of CYP enzymes, for example, is changed to a different value, the value of the intrinsic CL would need to be changed as well, to provide a good fit of the model to the data.

The fraction unbound was also quite influential. Here the value of 0.5 was used as stated in the label of moclobemide and also mentioned by Mayersohn et al.[^61^](#_ENREF_61) No other source was found to confirm this value hence it needed to be assumed that the value was unbiased.

Lipophilicity (logP) has also a relatively high impact on the PK predictions of moclobemide, especially C_max_. The value of 1.79 refers to experimental data as reported by Pons et al.[^62^](#_ENREF_62) It was assumed that the value was unbiased.

Muscle volume is the biggest compartment in the body, followed by skin; both impact distribution and thus exposure.

As can be expected, the gastric emptying time influences C_max_. The default value in PK-Sim is 15 min, which appeared to be a good representation of the average value in a healthy population.[^19^](#_ENREF_19) Given that the model was built and qualified on a number of different publications in different healthy populations it seems plausible to assume that the average gastric emptying time across all of subjects in these studies would not deviate very much from the default value.

In summary, findings from the sensitivity analysis were in line with expectations, i.e. that these parameters influence the predicted AUC and C_max_. Attention should be paid to the gastric emptying time for moclobemide.

# **SECTION S2.8. Glossary of abbreviations used in this document**

| ADME | Absorption, Distribution, Metabolism, Excretion |
| --- | --- |
| AUC | Area under the plasma concentration versus time curve |
| AUCR | Area under the plasma concentration versus time curve ratio |
| b.i.d. | Twice daily (bis in diem) |
| BMI | Body mass index |
| CL | Clearance |
| Clint | Intrinsic liver clearance |
| C_max_ | Maximum concentration |
| CmaxR | Maximum concentration Ratio |
| CYP | Cytochrome P450 oxidase |
| CYP1A2 | Cytochrome P450 1A2 oxidase |
| CYP2C19 | Cytochrome P450 2C19 oxidase |
| CYP3A4 | Cytochrome P450 3A4 oxidase |
| DDI | Drug-drug interaction |
| EE | Ethinylestradiol |
| EM | Extensive metabolizers |
| fm | Fraction metabolized |
| FMO | Flavin-containing monooxygenase |
| fu | Fraction unbound |
| FDA | Food and Drug administration |
| GFR | Glomerular filtration rate |
| HLM | Human liver microsomes |
| i.v. | Intravenous |
| IVIVE | In Vitro to In Vivo Extrapolation |
| k_cat_ | Catalyst rate constant |
| K_i_ | Inhibitor constant |
| Kinact | Rate of enzyme inactivation |
| Km | Michaelis Menten constant |
| OSP | Open Systems Pharmacology |
| PBPK | Physiologically-based pharmacokinetics |
| PK | Pharmacokinetics |
| PM | Poor metabolizers |
| RT-PCR | Reverse transcription polymerase chain reaction |
| p.o. | Per os |
| q.d. | Once daily (quaque diem) |
| SD | Single Dose |
| SPC | Summary of Product Characteristics |
| SD | Standard deviation |
| TDI | Time dependent inhibition |
| t.i.d. | Three times a day (ter in die) |
| T_max_ | Time to reach C_max_ |
| UGT | Uridine 5'-diphospho-glucuronosyltransferase |
| V_max_ | Maximum enzymatic rate achieved by the system at saturating substrate concentration |

# **SECTION S2.9. REFERENCES**

1. Alqahtani S., Kaddoumi A. Development of a Physiologically Based Pharmacokinetic/Pharmacodynamic Model to Predict the Impact of Genetic Polymorphisms on the Pharmacokinetics and Pharmacodynamics Represented by Receptor/Transporter Occupancy of Central Nervous System Drugs. *Clin Pharmacokinet* **55** 957-969. (2016)

2. Britz H.*, et al.* Physiologically-Based Pharmacokinetic Models for CYP1A2 Drug-Drug Interaction Prediction: A Modeling Network of Fluvoxamine, Theophylline, Caffeine, Rifampicin, and Midazolam. *CPT Pharmacometrics Syst Pharmacol* **8** 296-307. (2019)

3. Iga K. Use of three-compartment physiologically based pharmacokinetic modeling to predict hepatic blood levels of fluvoxamine relevant for drug-drug interactions. *J Pharm Sci* **104** 1478-1491. (2015)

4. De Vries M.H., Van Harten J., Van Bemmel P., Raghoebar M. Pharmacokinetics of fluvoxamine maleate after increasing single oral doses in healthy subjects. *Biopharm Drug Dispos* **14** 291-296. (1993)

5. Orlando R., De Martin S., Andrighetto L., Floreani M., Palatini P. Fluvoxamine pharmacokinetics in healthy elderly subjects and elderly patients with chronic heart failure. *Br J Clin Pharmacol* **69** 279-286. (2010)

6. Kunii T.*, et al.* Interaction study between enoxacin and fluvoxamine. *Ther Drug Monit* **27** 349-353. (2005)

7. Fukasawa T.*, et al.* Effects of caffeine on the kinetics of fluvoxamine and its major metabolite in plasma after a single oral dose of the drug. *Ther Drug Monit* **28** 308-311. (2006)

8. Spigset O., Granberg K., Hagg S., Norstrom A., Dahlqvist R. Relationship between fluvoxamine pharmacokinetics and CYP2D6/CYP2C19 phenotype polymorphisms. *Eur J Clin Pharmacol* **52** 129-133. (1997)

9. Spigset O., Granberg K., Hagg S., Soderstrom E., Dahlqvist R. Non-linear fluvoxamine disposition. *Br J Clin Pharmacol* **45** 257-263. (1998)

10. Fleishaker J.C., Hulst L.K. A pharmacokinetic and pharmacodynamic evaluation of the combined administration of alprazolam and fluvoxamine. *Eur J Clin Pharmacol* **46** 35-39. (1994)

11. Carrillo J.A.*, et al.* Disposition of fluvoxamine in humans is determined by the polymorphic CYP2D6 and also by the CYP1A2 activity. *Clin Pharmacol Ther* **60** 183-190. (1996)

12. U.S. Food and Drug Administration. FDA review Luvox. 2008.

13. Labellarte M.*, et al.* Multiple-dose pharmacokinetics of fluvoxamine in children and adolescents. *J Am Acad Child Adolesc Psychiatry* **43** 1497-1505. (2004)

14. Jeppesen U., Loft S., Poulsen H.E., Brsen K. A fluvoxamine-caffeine interaction study. *Pharmacogenetics* **6** 213-222. (1996)

15. Raza H., John A., Nemmar A. Short-term effects of nose-only cigarette smoke exposure on glutathione redox homeostasis, cytochrome P450 1A1/2 and respiratory enzyme activities in mice tissues. *Cell Physiol Biochem* **31** 683-692. (2013)

16. Perucca E., Gatti G., Spina E. Clinical pharmacokinetics of fluvoxamine. *Clin Pharmacokinet* **27** 175-190. (1994)

17. Boehringer Ingelheim. Study c13128239-01: Influence of fluvoxamine on the pharmacokinetics of BI 409306 after oral administration (randomized, open-label, two-treatment, two-sequence, two-period crossover study).

18. Wu F.*, et al.* Predicting nonlinear pharmacokinetics of omeprazole enantiomers and racemic drug using physiologically based pharmacokinetic modeling and simulation: application to predict drug/genetic interactions. *Pharm Res* **31** 1919–1929. (2014)

19. Open Systems Pharmacology Suite Community. Open Systems Pharmacology Suite Manual, Version 7.0. 2017.

20. Hassan-Alin M., Andersson T., Bredberg E., Rohss K. Pharmacokinetics of esomeprazole after oral and intravenous administration of single and repeated doses to healthy subjects. *Eur J Clin Pharmacol* **56** 665–670. (2000)

21. Wilder-Smith C.H.*, et al.* Intravenous esomeprazole (40 mg and 20 mg) inhibits gastric acid secretion as effectively as oral esomeprazole: results of two randomized clinical studies. *Eur J Gastroenterol Hepatol* **17** 191-197. (2005)

22. Hassan-Alin M., Andersson T., Niazi M., Rohss K. A pharmacokinetic study comparing single and repeated oral doses of 20 mg and 40 mg omeprazole and its two optical isomers, S-omeprazole (esomeprazole) and R-omeprazole, in healthy subjects. *Eur J Clin Pharmacol* **60** 779-784. (2005)

23. Andersson T., Regårdh C.G. Pharmacokinetics of Omeprazole and Metabolites Following Single Intravenous and Oral Doses of 40 and 80mg. *Drug Investig* **2** 255-263. (1990)

24. Olivares-Morales A., Ghosh A., Aarons L., Rostami-Hodjegan A. Development of a Novel Simplified PBPK Absorption Model to Explain the Higher Relative Bioavailability of the OROS(R) Formulation of Oxybutynin. *AAPS J* **18** 1532-1549. (2016)

25. Andersson T., Rohss K., Hassan-alin M. Pharmacokinetics (PK) and effect on pentagastrin stimulated peak acid output (PAO) of omeprazole (O) and its 2 optical isomers, S-omeprazole/esomeprazole (E) and R-omeprazole (R-O). **118** A1210. (1990)

26. Uno T.*, et al.* Absolute bioavailability and metabolism of omeprazole in relation to CYP2C19 genotypes following single intravenous and oral administrations. *Eur J Clin Pharmacol* **63** 143-149. (2007)

27. Regardh C.G., Andersson T., Lagerstrom P.O., Lundborg P., Skanberg I. The pharmacokinetics of omeprazole in humans--a study of single intravenous and oral doses. *Ther Drug Monit* **12** 163-172. (1990)

28. U.S. Food and Drug Administration. Clinical pharmacology and biopharmaceutics review - Nexium delayed-release capsules - esomeprazole sodium.

29. Andersson T., Cederberg C., Heggelund A., Lundborg P. The pharmacokinetics of single and repeated once-daily doses of 10, 20 and 40mg omeprazole as enteric-coated granules. *Drug Investig* **3** 45-52. (1991)

30. Oosterhuis B., Jonkman J.H., Andersson T., Zuiderwijk P.B. No influence of single intravenous doses of omeprazole on theophylline elimination kinetics. *J Clin Pharmacol* **32** 470-475. (1992)

31. Rohss K., Wilder-Smith C., Kilhamn J., Fjellman M., Lind T. Suppression of gastric acid with intravenous esomeprazole and omeprazole: results of 3 studies in healthy subjects. *Int J Clin Pharmacol Ther* **45** 345-354. (2007)

32. Yasui-Furukori N.*, et al.* Different inhibitory effect of fluvoxamine on omeprazole metabolism between CYP2C19 genotypes. *Br J Clin Pharmacol* **57** 487-494. (2004)

33. Cho J.Y.*, et al.* Omeprazole hydroxylation is inhibited by a single dose of moclobemide in homozygotic EM genotype for CYP2C19. *Br J Clin Pharmacol* **53** 393-397. (2002)

34. Nishimura M., Naito S. Tissue-specific mRNA expression profiles of human ATP-binding cassette and solute carrier transporter superfamilies. *Drug Metab Pharmacokinet* **20** 452-477. (2005)

35. Nishimura M., Naito S. Tissue-specific mRNA expression profiles of human phase I metabolizing enzymes except for cytochrome P450 and phase II metabolizing enzymes. *Drug Metab Pharmacokinet* **21** 357-374. (2006)

36. Nishimura M., Yaguti H., Yoshitsugu H., Naito S., Satoh T. Tissue distribution of mRNA expression of human cytochrome P450 isoforms assessed by high-sensitivity real-time reverse transcription PCR. *Yakugaku Zasshi* **123** 369-375. (2003)

37. Hassan-Alin M., Andersson T., Bredberg E., Rohss K. Pharmacokinetics of esomeprazole after oral and intravenous administration of single and repeated doses to healthy subjects. *Eur J Clin Pharmacol* **56** 665-670. (2000)

38. Liu K.H.*, et al.* Stereoselective inhibition of cytochrome P450 forms by lansoprazole and omeprazole in vitro. *Xenobiotica* **35** 27-38. (2005)

39. Abelo A.*, et al.* Stereoselective metabolism of omeprazole by human cytochrome P450 enzymes. *Drug Metab Dispos* **28** 966-972. (2000)

40. Boehringer Ingelheim. Study c02327040: Investigation of the effect of food and of increased gastric pH on the relative bioavailability of deleobuvir following single oral administration in healthy Caucasian and Japanese subjects (an open label, randomised, four-way crossover study).

41. Boehringer Ingelheim. Study c01959611: Investigation of the effect of food and of increased gastric pH on the relative bioavailability of a single oral dose of 240 mg faldaprevir in an open-label, randomised, three-way crossover trial in healthy subjects).

42. U.S. Food and Drug Administration. Nexium prescribing information. 2014 [cited 26 November 2019]Available from: <https://www.accessdata.fda.gov/drugsatfda_docs/label/2014/022101s014021957s017021153s050lbl.pdf>

43. Adedoyin A., Arns P.A., Richards W.O., Wilkinson G.R., Branch R.A. Selective effect of liver disease on the activities of specific metabolizing enzymes: investigation of cytochromes P450 2C19 and 2D6. *Clin Pharmacol Ther* **64** 8-17. (1998)

44. Iga K. Dynamic and Static Simulations of Fluvoxamine-Perpetrated Drug-Drug Interactions Using Multiple Cytochrome P450 Inhibition Modeling, and Determination of Perpetrator-Specific CYP Isoform Inhibition Constants and Fractional CYP Isoform Contributions to Victim Clearance. *J Pharm Sci* **105** 1307-1317. (2016)

45. Jacqz E., Hall S.D., Branch R.A., Wilkinson G.R. Polymorphic metabolism of mephenytoin in man: pharmacokinetic interaction with a co-regulated substrate, mephobarbital. *Clin Pharmacol Ther* **39** 646-653. (1986)

46. Wedlund P.J.*, et al.* Phenotypic differences in mephenytoin pharmacokinetics in normal subjects. *J Pharmacol Exp Ther* **234** 662-669. (1985)

47. Yao C., Kunze K.L., Trager W.F., Kharasch E.D., Levy R.H. Comparison of in vitro and in vivo inhibition potencies of fluvoxamine toward CYP2C19. *Drug Metab Dispos* **31** 565-571. (2003)

48. Kuepfer L.*, et al.* Applied Concepts in PBPK Modeling: How to Build a PBPK/PD Model. *CPT Pharmacometrics Syst Pharmacol* **5** 516-531. (2016)

49. Sangster J. A databank of evaluated octanol-water partition coefficients (Log P) on microcomputer diskette: Sangster Res Lab.; 1994.

50. Drugbank. Mephenytoin. [cited 2019 18 December 2019]Available from: <https://www.drugbank.ca/drugs/DB00532>

51. Steere B., Baker J.A., Hall S.D., Guo Y. Prediction of in vivo clearance and associated variability of CYP2C19 substrates by genotypes in populations utilizing a pharmacogenetics-based mechanistic model. *Drug Metab Dispos* **43** 870-883. (2015)

52. Hoskins J., Shenfield G., Murray M., Gross A. Characterization of moclobemide N-oxidation in human liver microsomes. *Xenobiotica* **31** 387-397. (2001)

53. Schoerlin M.P., Mayersohn M., Korn A., Eggers H. Disposition kinetics of moclobemide, a monoamine oxidase-A enzyme inhibitor: single and multiple dosing in normal subjects. *Clin Pharmacol Ther* **42** 395-404. (1987)

54. Raaflaub J., Haefelfinger P., Trautmann K.H. Single-dose pharmacokinetics of the MAO-inhibitor moclobemide in man. *Arzneimittelforschung* **34** 80-82. (1984)

55. Gram L.F., Guentert T.W., Grange S., Vistisen K., Brosen K. Moclobemide, a substrate of CYP2C19 and an inhibitor of CYP2C19, CYP2D6, and CYP1A2: a panel study. *Clin Pharmacol Ther* **57** 670-677. (1995)

56. Yu K.S.*, et al.* Effect of omeprazole on the pharmacokinetics of moclobemide according to the genetic polymorphism of CYP2C19. *Clin Pharmacol Ther* **69** 266-273. (2001)

57. Wiesel F.A., Raaflaub J., Kettler R. Pharmacokinetics of oral moclobemide in healthy human subjects and effects on MAO-activity in platelets and excretion of urine monoamine metabolites. *Eur J Clin Pharmacol* **28** 89-95. (1985)

58. Guentert T.W.*, et al.* Pharmacokinetics of moclobemide after single and multiple oral dosing with 150 milligrams 3 times daily for 15 days. *Acta Psychiatr Scand Suppl* **360** 91-93. (1990)

59. Rakic Ignjatovic A., Miljkovic B., Todorovic D., Timotijevic I., Pokrajac M. Moclobemide monotherapy vs. combined therapy with valproic acid or carbamazepine in depressive patients: a pharmacokinetic interaction study. *Br J Clin Pharmacol* **67** 199-208. (2009)

60. Dingemanse J., Wallnofer A., Gieschke R., Guentert T., Amrein R. Pharmacokinetic and pharmacodynamic interactions between fluoxetine and moclobemide in the investigation of development of the "serotonin syndrome". *Clin Pharmacol Ther* **63** 403-413. (1998)

61. Mayersohn M., Guentert T.W. Clinical pharmacokinetics of the monoamine oxidase-A inhibitor moclobemide. *Clin Pharmacokinet* **29** 292-332. (1995)

62. Pons G.*, et al.* Moclobemide excretion in human breast milk. *Br J Clin Pharmacol* **29** 27-31. (1990)
