# Supplementary material for: A Physiologically-Based Pharmacokinetic (PBPK) Model Network for the Prediction of CYP1A2 and CYP2C19 Drug–Drug–Gene Interactions with Fluvoxamine, Omeprazole, S-mephenytoin, Moclobemide, Tizanidine, Mexiletine, Ethinylestradiol, and Caffeine
Source: Pharmaceutics. 2020 Dec 8;12(12):1191. doi: 10.3390/pharmaceutics12121191 (PMC7764797; doi:10.3390/pharmaceutics12121191)
Supplement: Supplementary file 1 [file pharmaceutics-12-01191-s001.zip › pharmaceutics-1010984 supplementary/PBPK_manuscript_supplement S2b_Pharmaceutics.docx]

**Electronic Supplementary Material**

# **Supplement S2****b: Model development and evaluation (CYP1A2 predominant): mexiletine, ethinylestradiol, caffeine, tizanidine**

- 1. Tizanidine model
     1. Model Development Strategy

As concentration-time profiles following intravenous administration are not publicly available, model building was based on data following oral administration.

1. Fit intrinsic CL of CYP1A2, and Weibull absorption parameters (renal elimination fixed to GFR) using data from single dose studies where 4 or 8 mg tablets where given in the fasted state to healthy volunteers. The fitting was done for each of the five tissue distribution models available in PK-Sim.
2. Predictions for fed-state. If adjustments are necessary, only absorption relevant parameters will be fitted.
   - Tablet-fed (2, 4, 8 mg)
   - Capsule-fed (4, 8 mg)
3. Model evaluation: Predict multiple dosing of 4 mg (tablet, fasted) using the best model and parameters from the previous step. If no adjustment of parameters is necessary, move on to next step.
4. Predict drug-drug interaction with fluvoxamine as inhibitor.

Table S2.17 Model development steps – tizanidine model

| **Step** | **Figure and Table in text** | **Purpose** | **Data** |
| --- | --- | --- | --- |
| **1** | Table S2.18  Figure S2.24 | Fitting the CYP1A2 elimination and absorption | Backman 2008[^1^](#_ENREF_1)  Backman 2006[^2^](#_ENREF_2)  Al-Ghazawi 2013[^3^](#_ENREF_3)  Granfors 2004[^4^](#_ENREF_4) |
| **2** | Table S2.19  Figure S2.25 | Food and formulation effect | Momo2010[^5^](#_ENREF_5)  Henney2007[^6^](#_ENREF_6)  Shah2006[^7^](#_ENREF_7) |
| **3** |  | Model evaluation | Tse 1987[^8^](#_ENREF_8)  Schellenberger 1999[^9^](#_ENREF_9) |
| **4** |  | DDI predictions | Granfors 2004[^4^](#_ENREF_4) |

- - 1. Model Development Tizanidine
       1. **Fitting the CYP1A2 elimination and absorption parameters**

Three parameters were optimized using digitized mean concentration time curves from the publications, where the 4 mg or 8 mg tablet was given to healthy volunteers after an overnight fast (Table S2.18). The data were well described with the optimized parameters with a slight tendency of under-predicting the mean profile from the 8-mg studies. One study, Al-Ghazawi et al.[^3^](#_ENREF_3), was excluded from the optimization as 31 of 33 volunteers were smokers and tizanidine concentrations were much lower compared to other studies, probably due to induction of CYP1A2 metabolism with smoking.

Table S2.18 Parameter estimates for tizanidine

| **Identification Parameter** | **95% Confidence Interval** |
| --- | --- |
| Intrinsic CL (CYP1A2) | 7.29 ± 1.20 [l/min] |
| Dissolution shape | 0.96 ±0.18 |
| Dissolution time (50% dissolved) | 38.53 ± 7.17 [min] |

The Rodgers and Rowland model described the data the best, thus this model was selected for all further predictions.

Absolute oral bioavailability was predicted to be approximately 12%. Tse et al.[^8^](#_ENREF_8) reported a bioavailability of 21% derived from a mass balance study. The fraction eliminated in urine was predicted at 0.71%, which is in accordance with the fact that about 95% of an oral dose of tizanidine is metabolized[^8^](#_ENREF_8).

To judge the predicted variability of the model, a population simulation was carried out generating a virtual population of 2000 healthy European male subjects with the weight and age range according to Granfors et al.[^10^](#_ENREF_10) (21–31 y, 65–83 kg). The width of the standard deviation band of the simulated population was in line with the height of the observed standard deviation error-bars as can be seen in Figure S2.24.

| 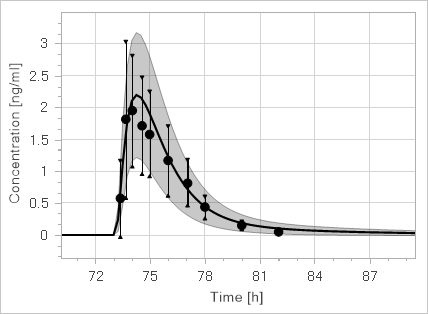 | 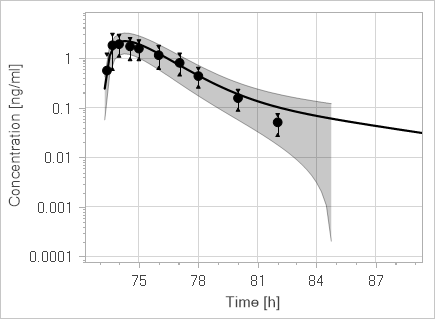 |
| --- | --- |

Figure S2.24 Population prediction of 4 mg tizanidine administered to healthy volunteers in fasted state on linear (left) and semi-log (right) scale.

The grey area encompasses the standard deviation (SD) of the predicted concentration time profiles for the 2000 virtual subjects. The black line is the arithmetic mean of the predictions. The black symbols and error bars correspond to the mean and SD as digitized from the control group in Granfors et al.2004 (single dose at day 4).

- - - 1. **Food and formulation effect**

The concentration–time profiles in the fed-state were predicted by including a ‘high-fat breakfast event’, at time=0 in the simulations. Such a meal is pre-specified in PK-Sim. The food event affects gastric emptying time (longer), gastric pH (higher) and intestinal transit times (longer).

**Tablet with food**

Predictions did not describe the observations well; T_max_ was predicted much later and C_max_ much lower than was observed in the publications; the effect of food on the gastro-intestinal transit time did not impact the absorption speed of the tablet formulation. Hence, the Weibull dissolution parameters were optimized for the fed condition without including a breakfast event in the simulations to descriptively model the observed changes after food intake.

The resulting fits were much closer to the observed concentrations, even though the profiles were not exactly described (Figure S2.25). The estimated Weibull parameters are given in Table S2.19. The estimated dissolution time is much faster as in the original model for the fasting state (3.8 min vs. 38.5 min). This suggests that food may enhance the dissolution of tizanidine released from the tablet and/or accelerates the tablet disintegration.

Table S2.19 Parameter estimates for tizanidine tablets given with food

| **Identification Parameter** | **95% Confidence Interval** |
| --- | --- |
| Dissolution shape | 0.39 ± 0.44 |
| Dissolution time (50% dissolved) | 3.77 ± 10.85 [min] |


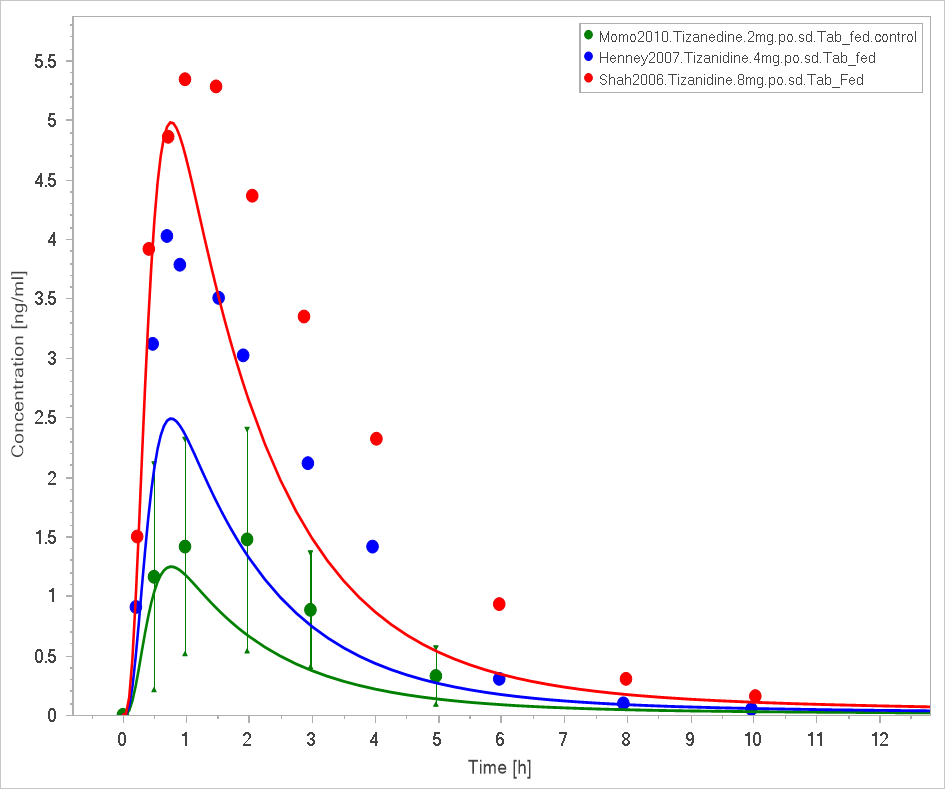


Figure S2.25 Predictions and observations of tizanidine tablets given with food. Weibull dissolution parameters were optimized.

**Capsule with food**

Simulations were performed with a ‘breakfast-event’ at time=0. As was already evident from the exploratory graphs (data not shown), the concentration-time profiles were very different when a capsule or a tablet is taken with food. In fact, the predicted curves with the breakfast event were very similar to the observations for capsules, with a significantly later T_max_, though peak concentration was underpredicted by approximately 2-fold (see assumptions/limitations table below).

- - 1. Assumptions and Limitations for the Tizanidine Model

The developed model for tizanidine included the following key assumptions/limitations:

| **Assumption/Limitation** | **Justification/impact** |
| --- | --- |
| The renal elimination is limited to glomerular filtration only. | The predicted small fraction eliminated in urine (~1%) is in line with literature reports (<5%). Given the minor contribution of the renal pathway no further exploration of active secretion was considered as the impact on DDI predictions is considered likely to be small. |
| CYP1A2 is the only metabolic pathway | No other pathway has been described for tizanidine. The modelling does not suggest that an additional metabolic pathway is necessary to describe the literature data. |
| C_max_ of capsule+food is underpredicted | The effect of food on the capsule could not be described well in terms of peak concentrations. DDI predictions should be restricted to the tablet formulation. The fa estimated by PK-Sim was 1.0 in both fed and fasted condition, indicating that fa was not the limiting factor in this model. |
| Food effect on tablets is due to enhanced dissolution and not affected by slow GI transit due to food. | A slower intestinal transit and delayed gastric emptying are causing a later Tmax, which is not in line with the observed profiles for tablets and food. Enhanced dissolution/disintegration seems a more plausible explanation. |
| Model was developed in non-smoking subjects | Model can only be used in non-smoking subjects |

- - 1. Tizanidine Model Evaluation and Qualification

To evaluate the model, a simulation was conducted giving 4 mg tizanidine three times a day for 4 days in a standard European male expressing CYP1A2 in liver only (age 30 y, weight = 73 kg, height = 176 cm, BMI = 23.57 kg/m^2^). The predicted multiple doses concentration–time profile was then visually compared (Figure S2.26) to mean observations from two studies[^8^](#_ENREF_8)^,^[^9^](#_ENREF_9). Note that these observed data were not used in the parameter optimization procedures.


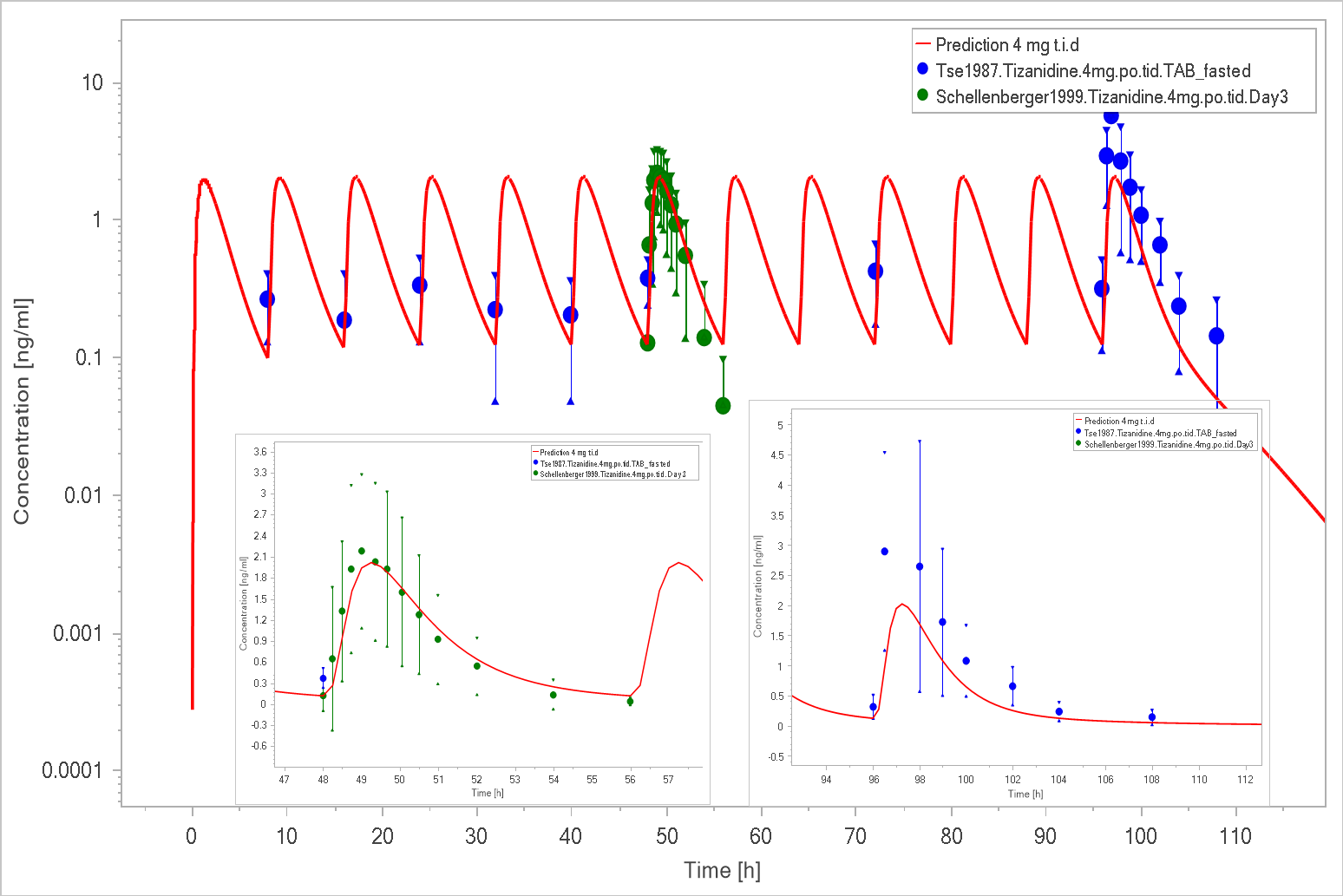


Figure S2.26 Prediction of concentration-time profile of tizanidine following 4 mg t.i.d. to healthy volunteers.

The inlay graphs show the steady state profile for Schellenberger et al.[^9^](#_ENREF_9) and Tse et al.^[8](#_ENREF_8)^

Given that the predictions closely matched the observed mean values, no further model refinement was considered necessary regarding fasting healthy volunteers.

- - 1. Sensitivity Analysis for Tizanidine Model

The results of a one-way sensitivity analysis with AUC and C_max_ as outcome parameters are shown in Figure S2.27 and Figure S2.28, respectively. The standard set of PK-Sim model parameters visible in simple view (N= 78, including e.g. solubility, metabolism by enzyme, organ volumes and blood flows) were investigated.


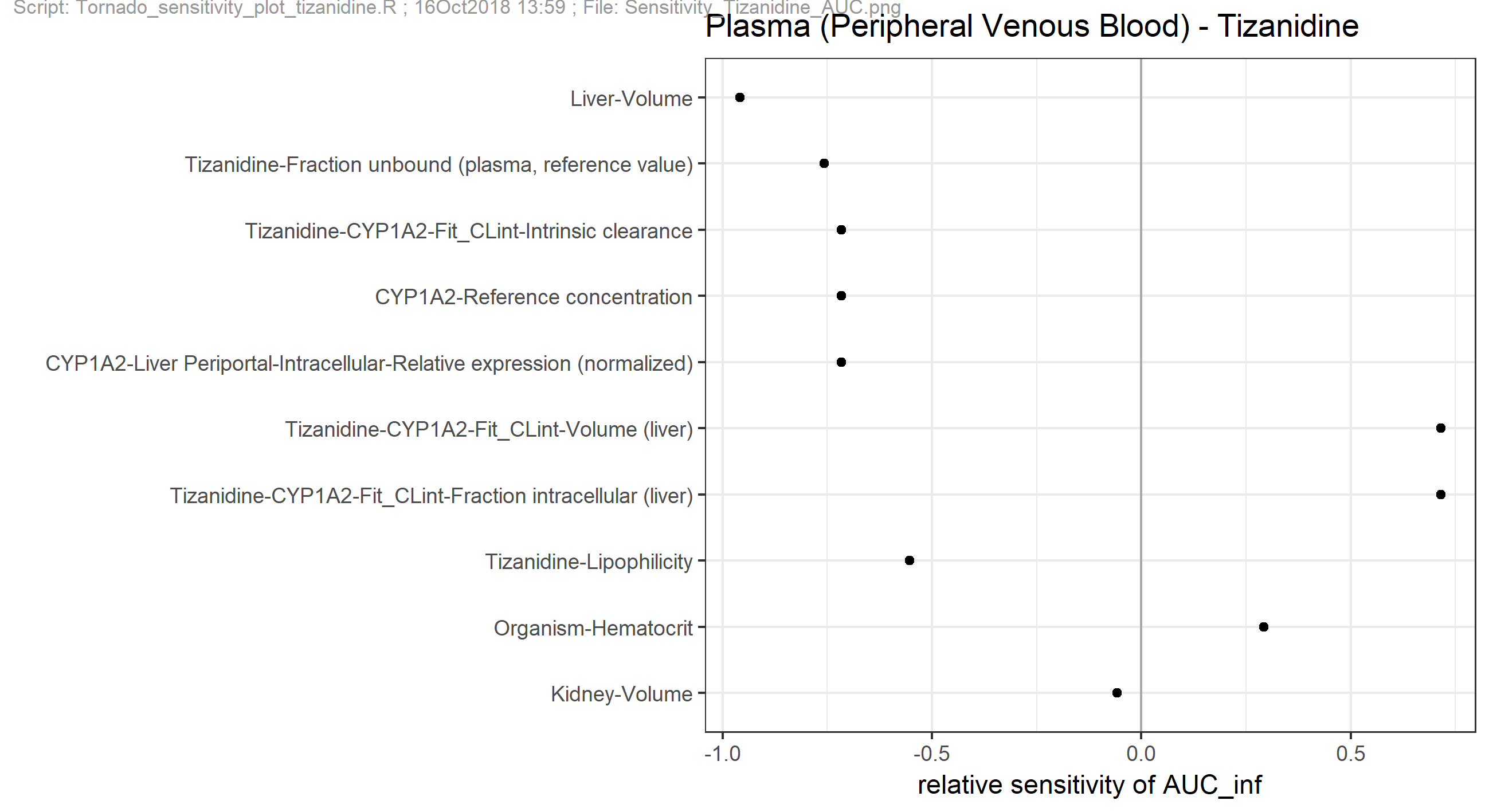


Figure S2.27 Sensitivity analysis tizanidine AUC (0 to infinity)


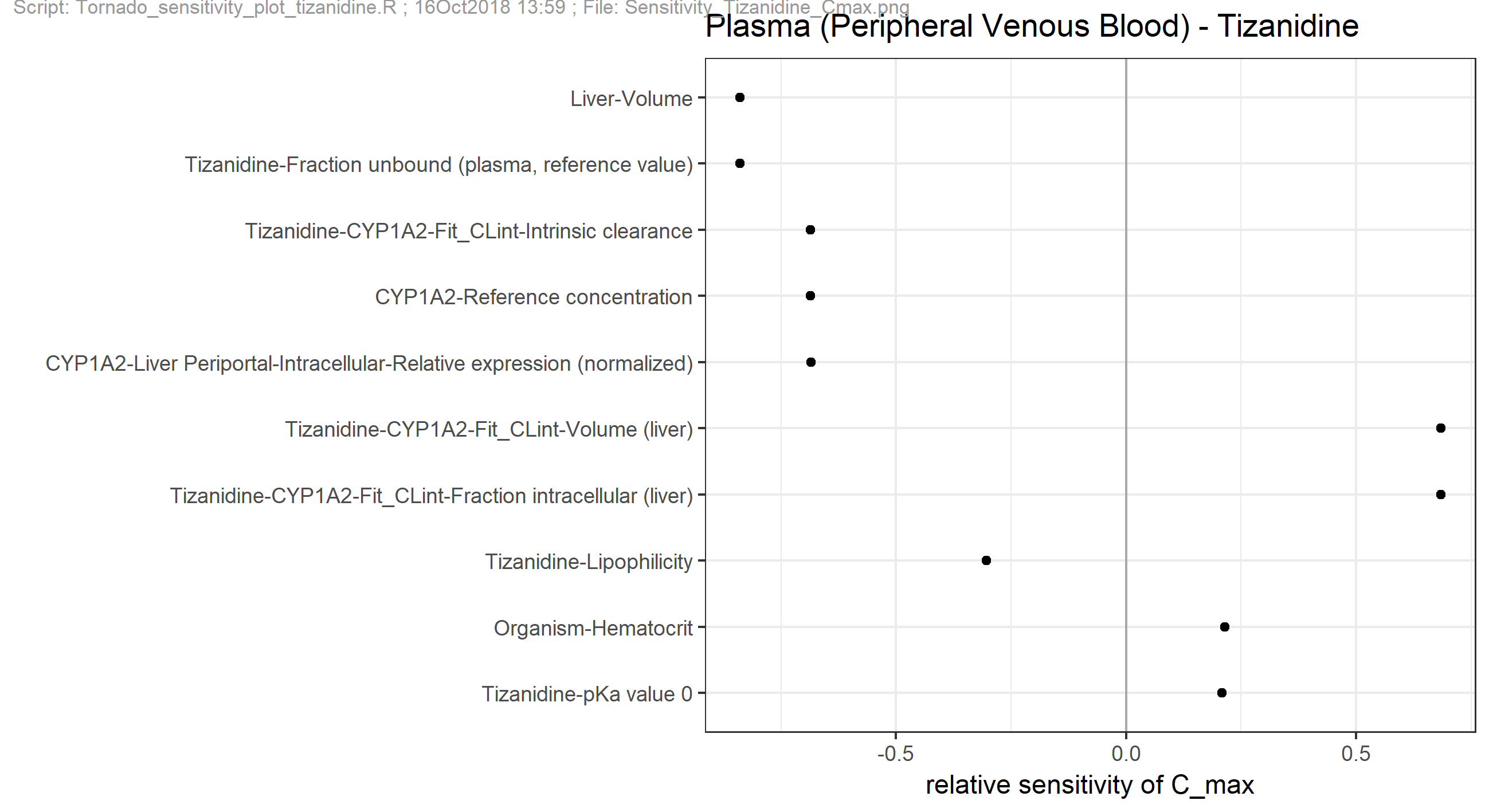


Figure S2.28 Sensitivity analysis tizanidine C_max_

Liver volume had the highest impact on the PK predictions for tizanidine, as could be expected given that tizanidine is almost completely metabolized in the liver, and the intrinsic CL value is directly proportional to the organ volume in PK-Sim. As this is a validated default parameter in PK-Sim backed by numerous literature sources, substantial bias in this value was not expected. Likewise, the parameters relating to CYP1A2 metabolism (reference concentration, intrinsic CL, relative expression cellular volume and fraction) are also very influential on the PK as expected. However, these parameters are interrelated and the intrinsic CL was an estimated variable. If one parameter is changed to a different value, for example the relative expression of CYP enzymes, then the value of the intrinsic CL would also need to be changed to provide a good fit of the model to the data.

The fraction unbound was also quite influential; the value of 0.7 was used as stated in the label of tizanidine[^11^](#_ENREF_11). No other source was found to confirm this value and it was assumed that the value is unbiased.

Lipophilicity (logP) had also a relatively high impact on the PK predictions of tizanidine. The value of 1.4 that was used is cited in DrugBank as an experimental value and is close to 1.6, the value predicted with ALOGPS.

Hematocrit and kidney volume are physiological default parameters in PK-Sim and are not expected to be biased.

No experimental pK_a_ value was available for tizanidine hence the assumption needs to be made that the value calculated by ChemAxon is accurate.

In summary, findings from the sensitivity analysis were in line with expectations, i.e. that these parameters influence the AUC and C_max_ of the predictions.

- 1. CAFFEINE model

The caffeine model utilized for this model was as described previously in PK-Sim documentation. The caffeine model was qualified using caffeine data from internal studies NCT01879371 (1335-0002, 100 mg caffeine) and (1241-00027, 200 mg caffeine).

- 1. Mexiletine model
     1. Model Development Strategy

A stepwise approach was used to fit the model to data.

1. Define distribution model, cellular permeability, renal and metabolic clearance on data after single i.v. administration. For this purpose, literature values from Drugs.com[^12^](#_ENREF_12) were derived for renal CL and CYP2D6 combined with CYP1A2 metabolic clearance, or total hepatic CL, fitted against the data.
2. Define intestinal permeability and fraction absorbed by fitting the model to data after p.o. single dose administration[^13^](#_ENREF_13). Investigate multiple oral doses predictions in CYP2D6 extensive and poor metabolizers[^14^](#_ENREF_14).
3. Qualification of the model with clinical interaction studies with caffeine and tizanidine as CYP1A2 substrates
4. Qualification of the model with clinical interaction study with fluvoxamine as CYP1A2 inhibitor

Table S2.20 Model development steps – mexiletine model

| **Step** | **Figure and Table in text** | **Purpose** | **Data** |
| --- | --- | --- | --- |
| **1** | Table S2.25 | 1. Define distribution and metabolism based on i.v. | Pentikäinen 1984^[15](#_ENREF_15)^  Campbell 1978[^16^](#_ENREF_16) |
| **2** |  | 2. Define mexiletine absorption based on p.o. | Pringle 1986[^13^](#_ENREF_13)  Begg 1982[^17^](#_ENREF_17)  Kusumoto 1998[^18^](#_ENREF_18)  Joeres 1987[^19^](#_ENREF_19)  Campbell 1978[^16^](#_ENREF_16) |
| **3a** |  | 3a. DDI prediction with caffeine | Labbé 2000[^14^](#_ENREF_14) |
| **3b** |  | 3b. DDI prediction with Tizanidine | Joeres 1987[^19^](#_ENREF_19) |
| **4** |  | 4. DDI with fluvoxamine | Kusumoto 2001[^20^](#_ENREF_20) |

- - 1. Model Development Mexiletine
       1. **Define distribution model, cellular permeability and LogP**

As a first step, only i.v. data after single administrations[^15^](#_ENREF_15)^,^[^16^](#_ENREF_16)^,^[^21^](#_ENREF_21) in healthy volunteers and patients were used to identify the most suitable distribution method, cellular permeabilities, lipophilicity and total hepatic clearance at the same time. Renal clearance (R-mexiletine) was obtained from literature[^14^](#_ENREF_14). The predefined “Standard European Male for DDI” individual was used (age=30 y, weight=73 kg, height=176 cm, BMI=23.57 kg/m^2^). The Rodgers & Rowland distribution model together with the PK-Sim standard permeability method together with fitted specific cellular permeability provided the best fit with lowest total error.

An overview of estimated parameters is as shown in Table S2.21. Estimations for lipophilicity were within the ranges found in literature. The estimated hepatic CL was within 2-fold of a value described in literature. The estimated, actual cellular permeability was much lower than the permeability calculated by PK-Sim using only lipophilicity and not taking into account charge, as expected.

Table S2.21 Lipophilicity, hepatic plasma clearance and specific organ permeability estimates based on i.v. data

| **Identification Parameter** | **95% Confidence Interval** | **Literature values (Source)** |
| --- | --- | --- |
| Lipophilicity [Log Units] | 2.38 ± 0.05 [Log Units] | 2.15-2.46 (DrugBank) |
| Hepatic plasma clearance [ml/h/kg] | 265.11 ± 25.13 [ml/h/kg] | 479.45 (Labbe 2000) |
| Specific organ permeability [cm/min] | 1.64E-3 ± 7.39E-4 [cm/min] | 0.12-0.29 (calculated PK-Sim) |

The predicted fraction excreted in urine was similar to the fraction reported in the label (9% vs 10%).

The estimated total hepatic CL was then divided into 1A2, 2D6 and unspecific metabolic clearances according to ratios from Labbe et al. 2000 (R-mexiletine)[^14^](#_ENREF_14), namely 28.6% to CYP1A2, 37.1% to CYP2D6 and the remaining 34.3% to some unspecific liver clearance. For this, in the predefined “Standard European Male for DDI” individual, CYP2D6 and CYP1A2 expressions were obtained from PK-Sim RT-PCR database, and an unspecific metabolizing enzyme was added in intracellular liver (ref. conc=1 µM, t_half=36h).

- - - 1. **Predicting Mexiletine p.o. profiles following oral solution**

As a next step, data from single dose oral administration[^13^](#_ENREF_13)^,^[^17^](#_ENREF_17)^,^[^18^](#_ENREF_18)^,^[^19^](#_ENREF_19)^,^[^21^](#_ENREF_21) were used to adjust gastrointestinal permeability. Clearance, lipophilicity and distribution model were fixed from the previous step. Intestinal permeability (transcellular) was estimated to 4.74E-4 ± 1.57E-4 [cm/min] (estimated value +/- 95% confidence interval). The model could describe the data well.

- - 1. Assumptions and Limitations for the Mexiletine Model

The developed model for mexiletine included the following assumptions:

| Assumption | Justification/impact |
| --- | --- |
| Metabolic clearances have been divided according to the ratios in Labbé et al[^14^](#_ENREF_14) for R-mexiletine | Ratios from S-mexiletine have also been tested and provided similar results. |

- - 1. Mexiletine Model Evaluation and Qualification

Mexiletine plasma concentrations after multiple doses from Labbé et al 2000[14](#_ENREF_14) were predicted in CYP2D6 extensive (EM) and poor metabolizers (PM). The predefined “Standard European Male for DDI” individual (age=30 y, weight=73 kg, height=176 cm, BMI=23.57 kg/m^2^) for both EM and PM was used. CYP2D6-mediated clearance was shut-off (set to 0) in CYP2D6 PMs. The data from Labbé et al[^14^](#_ENREF_14) were not used previously for model building or parameter estimations. The model adequately predicted accumulation after multiple doses in both CYP2D6 phenotypes, despite a slightly lower predicted clearance for EM subjects compared to observed clearance (Figure S2.29).


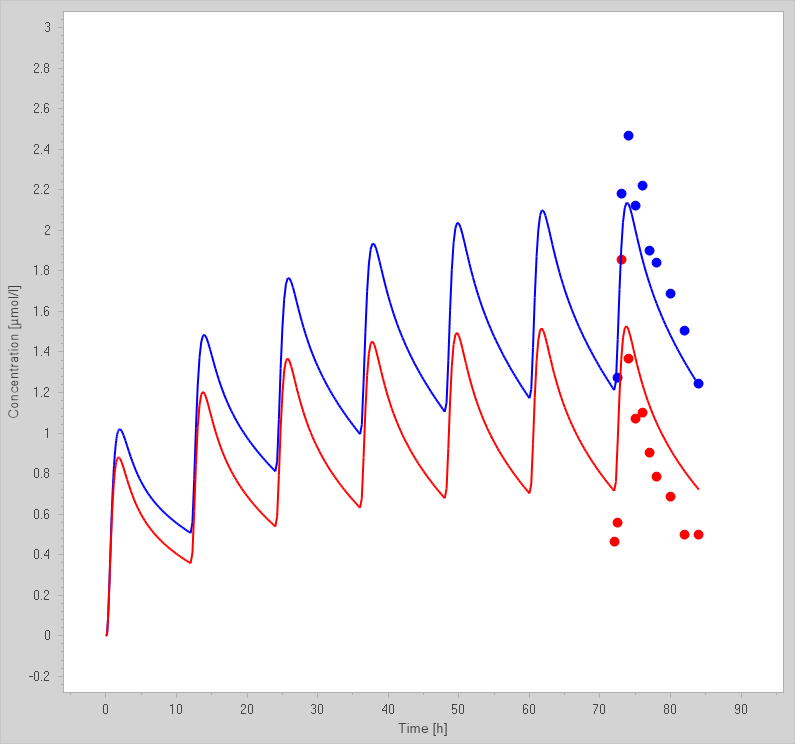


Figure S2.29 Predicted and observed mexiletine concentrations after multiple doses in CYP2D6 poor (blue) and extensive metabolizers (red).

Observed data from Labbé et al^[14](#_ENREF_14)^

Table S2.22 Observed and predicted Mexiletine PK parameters in CYP2D6 extensive and poor metabolizers

|  | **EM observed** | **EM predicted** | **Obs/pred ratio in EM** | **PM observed** | **PM simulated** | **Obs/pred ratio in PM** |
| --- | --- | --- | --- | --- | --- | --- |
| AUC [μg.min/mL] | 110.7 | 136.9 | 0.81 | 226.5 | 210.7 | 1.07 |
| C_max_ [μmol/L] | 1.86 | 1.53 | 1.21 | 2.47 | 2.14 | 1.16 |

Population simulations of single p.o. doses over a wide range of dose levels (83-500 mg) were conducted to visually compare the predicted concentration-time profiles to the observed concentrations reported in the literature, in terms of mean and variability[^22^](#_ENREF_22). A population of 1000 male individuals was generated based on “Standard European Male for DDI”. Age range was limited to 20-40 years. The concentration time profile was simulated for each virtual subject and summarized as mean and +/- SD.

Observed variability was generally larger than predicted variability (Figure S2.30). Depending on the study population, smoking status or variation in CYP-phenotypes may lead to additional variability that might be not included in the PK-Sim ontogeny factor.


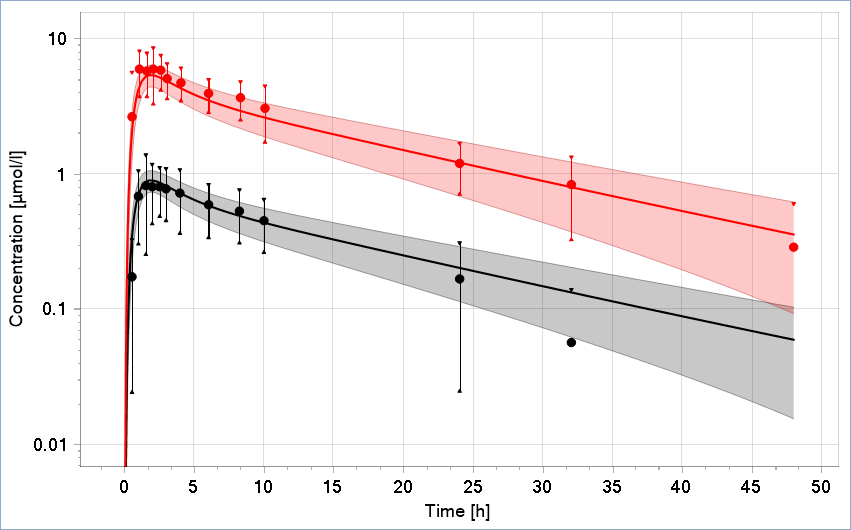


Figure S2.30 Predicted and observed (mean +/-SD) mexiletine concentrations after single 83 mg (black) – 500 mg (red) oral dose

- - 1. Sensitivity Analysis for Mexiletine Model

The results of the one-way sensitivity analysis with AUC and C_max_ as outcome parameters are shown in Figure S2.31 and Figure S2.32, respectively. The standard set of PK-Sim model parameters visible in simple view (N=227, including solubility, metabolism by enzyme, organ volumes and blood flows) were investigated.


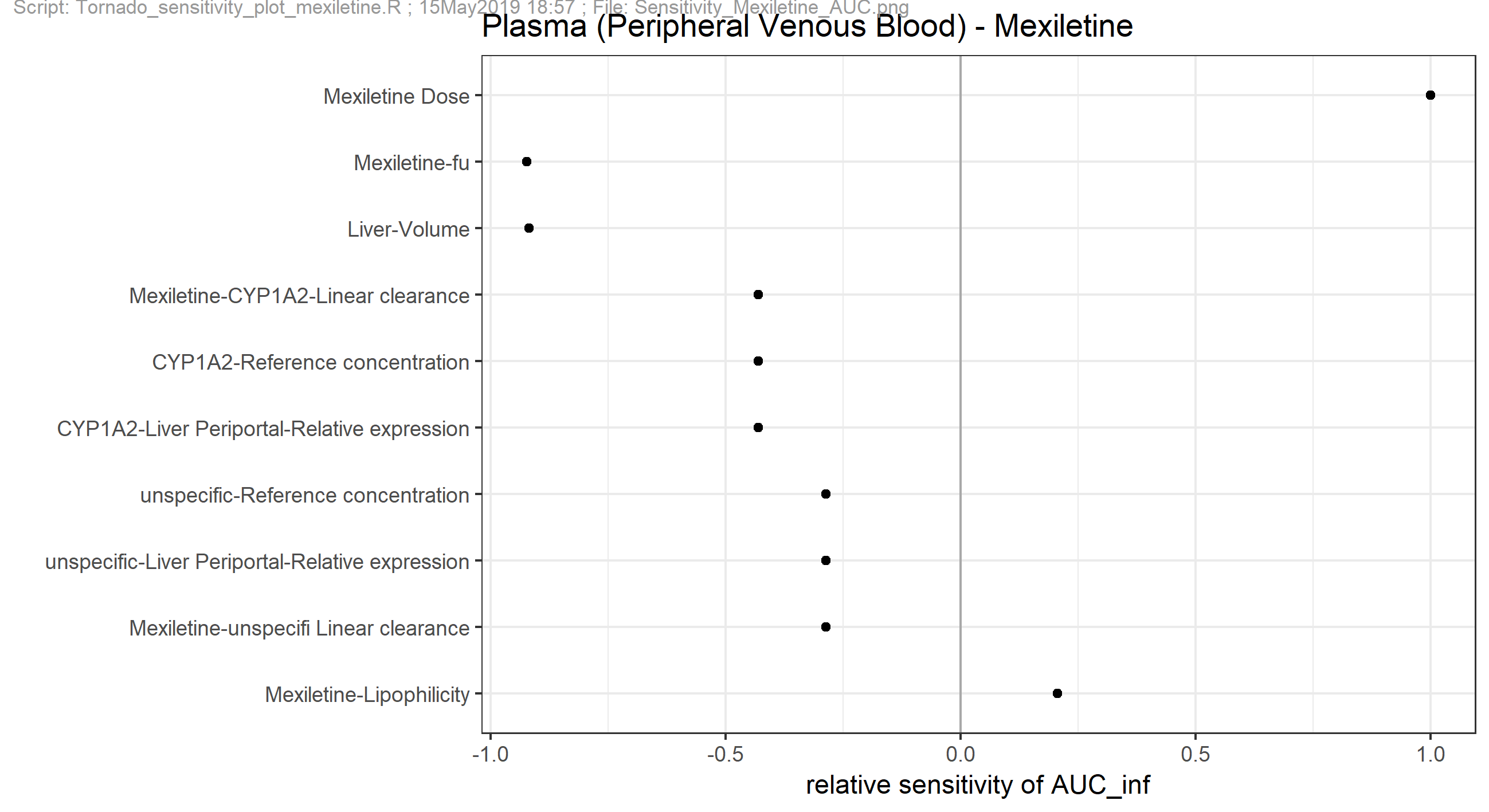


Figure S2.74 Sensitivity analysis mexiletine AUC (0 to infinity)


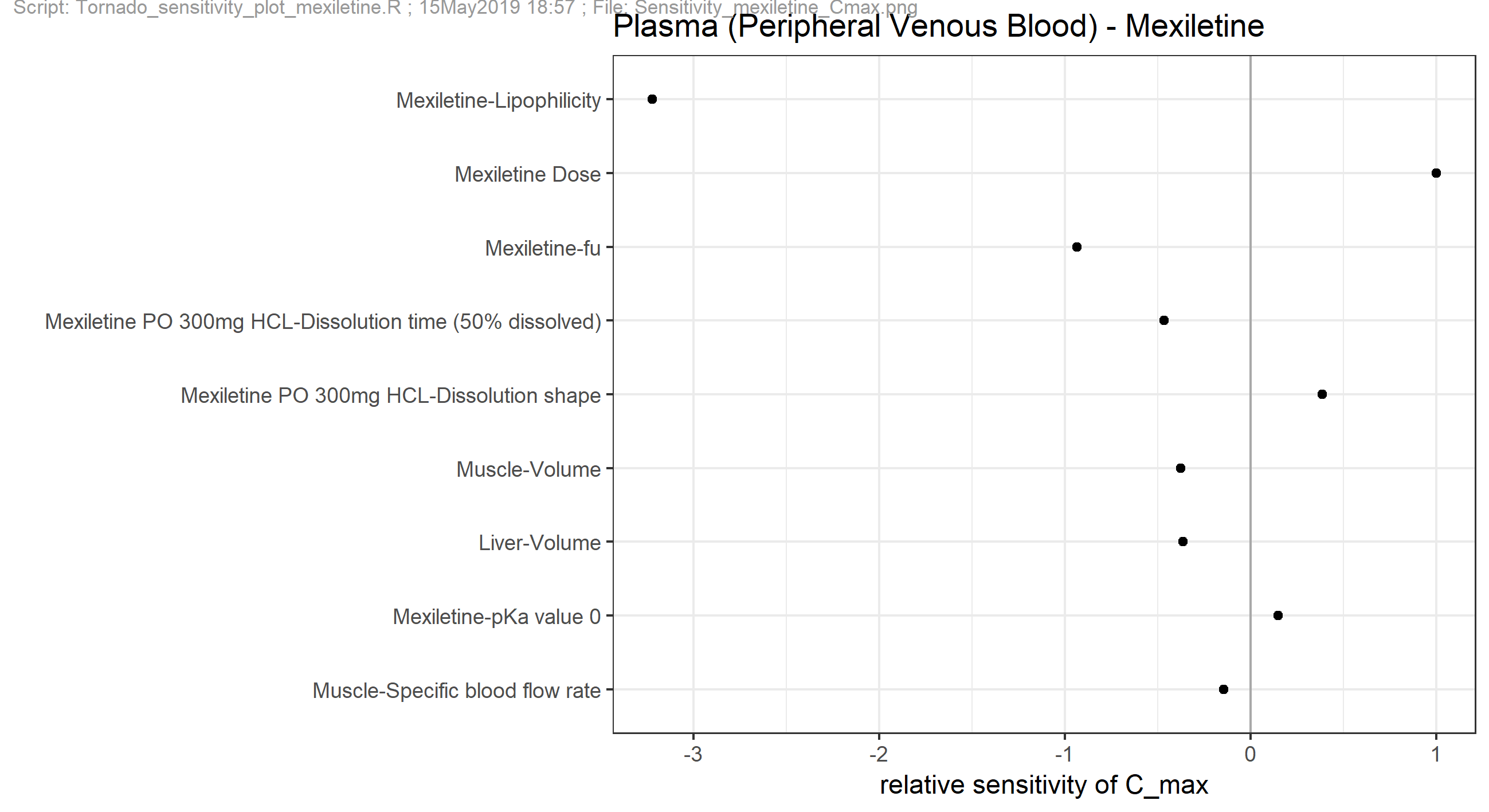


Figure S2.75 Sensitivity analysis mexiletine C_max_

Lipophilicity (logP) has a high impact on the PK predictions for mexiletine (mainly on C_max_, but also on AUC). In the current model, we estimated logP to be 2.38. Other sources report a very similar value (e.g DrugBank: 2.15-2.45). Therefore, it may be concluded that the lipophilicity used in the PBPK model is unlikely to be substantially different from reality.

The liver volume is also very influential on AUC as it impacts the total body clearance. The value used for the liver volume is a function of body weight in PK-Sim. Given that this is a validated default parameter in PK-Sim backed by numerous literature sources, substantial bias in this value is not expected.

The parameters relating to CYP1A2 and liver unspecific metabolism (reference concentration, intrinsic CL, relative expression in liver periportal) are also very influential on the PK. Notice however that these parameters are inter-dependent and that the intrinsic CL was an estimated variable. Hence if one parameter is changed, for example, relative expression of CYP enzymes, the value of the intrinsic CL would be estimated differently, in order to provide a good fit of the model to the data.

As can be expected, the formulation parameters also affect the PK parameters (mainly C_max_). However, the current set of parameters describe properly all the concentration-time profiles after both capsules and tablets, so a different set of parameters is not expected to be needed. Furthermore, metabolic clearances and distribution models have been determined based on i.v. data, where formulation parameters do not play a role, so that a substantial bias for these parameters is not expected.

Fraction unbound influences distribution but also body clearance as fu determines the fraction of compound available for metabolism. The current value (0.50) was obtained from DrugBank and similar values are reported in the label (0.40-0.50).

Muscle volume and blood flow rate are also influential on C_max_. This could be explained by the fact that mexiletine is a blocker of skeletal-muscle sodium channels. As for the liver volume, these are validated default parameters in PK-Sim backed by numerous literature sources, and substantial bias in these values are not expected.

In summary, findings from the sensitivity analysis were in line with expectations, i.e. that these parameters influence the predicted AUC and C_max_.

- 1. Ethinylestradiol model
     1. Model Development Strategy

The following steps were undertaken during ethinylestradiol model development:

1. Define lipophilicity and distribution model on data after i.v. administration
2. Predict p.o. data after single dose and at steady state
3. Detail metabolic contribution of different CYPs and UGTs to total hepatic clearance.
4. Qualification of the model with clinical data
5. Qualification of the model with clinical interaction studies with caffeine and tizanidine as CYP1A2 substrates.

The caffeine model utilized for this model was as described previously in PK-Sim documentation. The caffeine model was qualified using caffeine data from internal studies NCT01879371 (1335-0002, 100 mg caffeine; 1241-00027, 200 mg caffeine).[^23^](#_ENREF_23)^,^[^24^](#_ENREF_24)

Table S2.23 Model development steps – ethinylestradiol model

| **Step** | **Figure and Table in text** | **Purpose** | **Data** |
| --- | --- | --- | --- |
| **1** | Table S2.24  Figure S2.33 | Define absorption, distribution, and metabolism as total hepatic clearance based on i.v. and p.o. data.  Note that for later development the total hepatic clearance process was replaced by detailed enzymatic metabolism but the other disposition parameters were kept | Back 1981[^25^](#_ENREF_25) i.v. and p.o.  Back 1979[^26^](#_ENREF_26) i.v. and p.o.  Orme 1991[^27^](#_ENREF_27) i.v. and p.o.  Goebelsmann 1986[^28^](#_ENREF_28) solution and tablet p.o.  Stanczyck 1983[^29^](#_ENREF_29) solution and tablet  p.o.  Zhang 2017[^30^](#_ENREF_30) p.o.  Kothare 2012[^31^](#_ENREF_31) p.o.  Timmer 2000[^32^](#_ENREF_32) p.o. |
| **2** | Figure S2.34 | Estimate TDI on CYP1A2 | Granfors 2005[^33^](#_ENREF_33) |
| **3a** | Table 3.26 – Table 3.28 | DDI predictions with tizanidine | Granfors 2005[^33^](#_ENREF_33) |
| **3b** |  | DDI predictions with caffeine | Balogh 1995[^34^](#_ENREF_34) |

- - 1. Model Development Ethinylestradiol
       1. **Define a distribution model**

As a first step only i.v. data[^25^](#_ENREF_25)^,^[^26^](#_ENREF_26)^,^[^27^](#_ENREF_27)^,^[^35^](#_ENREF_35) were used to identify the most suitable distribution model. Renal clearance was directly obtained from literature[^36^](#_ENREF_36). A standard female subject was created based on the European (ICRP,2002) PK-Sim database (age=30 y, weight=60 kg, height=163 cm, BMI=22.58 kg/m^2^).

It was then decided to perform a simultaneous fit of i.v. and p.o.[^25^](#_ENREF_25)^,^[^26^](#_ENREF_26)^,^[^27^](#_ENREF_27)^,^[^28^](#_ENREF_28)^,^[^29^](#_ENREF_29)^,^[^30^](#_ENREF_30)^,^[^31^](#_ENREF_31)^,^[^32^](#_ENREF_32) single-dose data to identify the distribution model, total hepatic clearance, intestinal permeability and formulation. The Berezhkovskiy distribution model provided the best fit (Figure S2.33). The estimates for intestinal permeability, formulation and the distribution model were kept in later model versions.

Table S2.24 Lipophilicity, hepatic plasma clearance, intestinal permeability and formulation estimates based on i.v. and p.o. data

| **Identification Parameter** | **95% Confidence Interval** |
| --- | --- |
| Total hepatic plasma clearance | 5.54 ± 0.36 [ml/min/kg] |
| Lipophilicity [Log Units] | 3.48± 0.08 [Log Units] |
| Intestinal permeability (transcellular) | 1.68E-4 ± 1.41E-4 [cm/min] |
| Dissolution time (50% dissolved) | 36.51 ± 15.27 [min] |
| Lag time | 6.77 ± 2.27E-4 [min] |

| 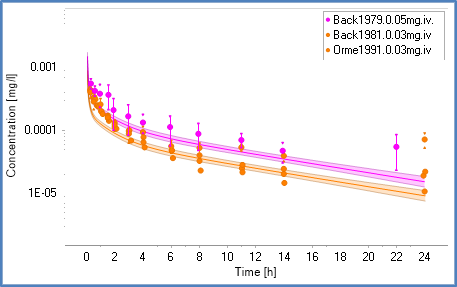 | 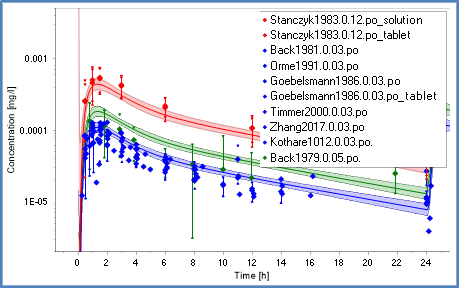 |
| --- | --- |

Figure S2.33 Ethinylestradiol concentration-time profile after single dose i.v. (left) and p.o. (right) dose.

Similarly, the model predicted ethinylestradiol concentrations at steady state well (30 and 50 ug Q.D.)[^25^](#_ENREF_25)^,^[^37^](#_ENREF_37)^,^[^38^](#_ENREF_38)^,^[^39^](#_ENREF_39), although some underprediction of trough concentrations were observed.

- - - 1. **Define the metabolic contribution of different CYPs and UGTs to total hepatic clearance**

For this purpose metabolic and renal clearance values were obtained from Ezuruike et al.[^36^](#_ENREF_36) (see Table S2.25), where in vitro enzyme kinetic parameters were scaled up using physiological data (Clint from HLM, Fm from study with recombinant enzymes) or fitted to the data using a minimal PBPK model with first-order absorption. CYP3A4, CYP2C9, CYP1A2, CYP2C8 and UGT1A1 expressions were obtained from RT-PCR database of PK-Sim and added to the standard female subject created previously. An additional unspecified liver enzyme was also included (reference concentration=1µM; t-half in liver=36h).

Table S2.25 Ethinylestradiol enzyme kinetic parameters from Ezuruike et al.

| **Elimination** | **Enzyme kinetics** | **Comment** |
| --- | --- | --- |
| CYP3A4 Clint [µL/min/pmol] | 0.5 | Optimized with ketoconazole study[^40^](#_ENREF_40) |
| CYP2C9 Clint [µL/min/pmol] | 0.51 | Optimized fm^[41](#_ENREF_41)^ |
| CYP1A2 Clint [µL/min/pmol] | 0.51 | Optimized fm^[41](#_ENREF_41)^ |
| CYP2C8 Clint [µL/min/pmol] | 0.13 | Optimized fm^[41](#_ENREF_41)^ |
| UGT1A1 Vmax [pmol/min/mg prot] | 408.5 | Shiraga et al.[^42^](#_ENREF_42) |
| UGT1A1 Km [µM] | 19.22 | Shiraga et al.[^42^](#_ENREF_42) |
| Additional Clint HLM [µL/min/mg prot] | 118.8 | Retrograde calculation |
| CLR [L/h] | 2.08 | Stanczyk et al[^43^](#_ENREF_43) |

The model with estimated total hepatic clearance (Table S2.24; tot Hep CL) and the one with explicit contribution of various CYP and UGT enzymes (Table S2.25; CYPs and UGTs) perform similarly after both i.v. and p.o. doses (Figure S2.34).

| 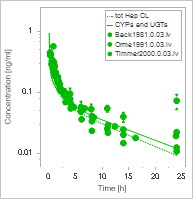 | 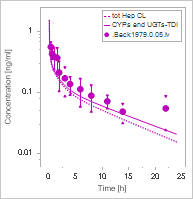 | 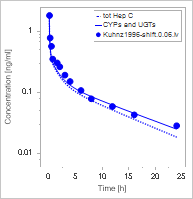 |
| --- | --- | --- |
| 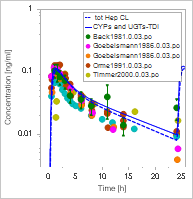 | 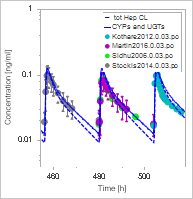 | 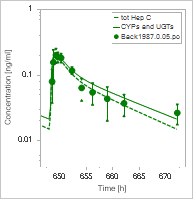 |

Figure S2.34 Ethinylestradiol concentration-time profile after i.v. (top row) and p.o. (last row) administration.

Tot Hep CL = model with fitted total hepatic CL; CYPs and UGTs=model with explicit contribution of CYP3A4, CYP2C9, CYP1A2, CYP2C8 and UGT1A1.

- - 1. Assumptions and Limitations for the Ethinylestradiol Model

The developed model for ethinylestradiol included the following assumptions:

| Assumption | Justification/impact |
| --- | --- |
| Ethinylestradiol was administered together with progestin as combined oral contraceptives. Combination is marketed as Gestodene^®^. Progestin coadministration is assumed to have no impact on the interaction with sensitive CYP1A2 substrates. | Progestin had no effect in vitro on CYP1A2, even at 100 μmol/L (ie, 10,000 times higher than the therapeutic concentration in plasma), as measured by phenacetin O-deethylation^[44](#_ENREF_44)^. |
| TDI mechanism was introduced to describe the observed interactions with tizanidine and caffeine | TDI mechanism for ethinylestradiol was not supported by the literature [^11^](#_ENREF_11)^,^[^44^](#_ENREF_44). However, EE-metabolites having a different half-life than the parent may result in prolonged CYP1A2 inhibition. This hypothesis could not be verified during model development, as no EE-metabolites data were available. |

- - 1. Ethinylestradiol Model Evaluation and Qualification

The final model was qualified by comparing predicted concentration-time profiles to individual observations obtained from a clinical study in Korean female subjects[^45^](#_ENREF_45). The age and BMI range of the study participants were used in the set-up of a virtual Asian population of N=1000 female subjects in PK-Sim.

Figure S2.35 shows overlaid the population simulation from the model including total hepatic clearance and the model including the contribution of the different CYPs and UGTs; the variability of the UGTs is wider, which derives from the variability of different CYP and UGT enzymes as implemented in the PK-Sim ontogeny database.

| 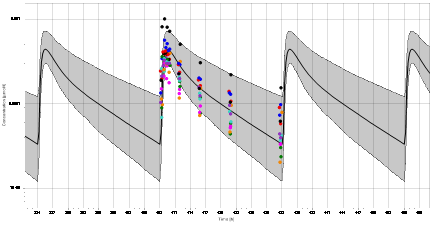 | 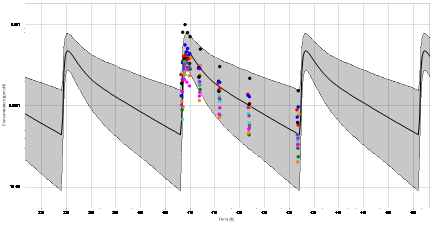 |
| --- | --- |

Figure S2.35 Predicted and observed ethinylestradiol concentrations after multiple dose in female subjects. Dots are observations, shaded areas are 90% CI from the model including total hepatic clearance (left) and the model including different CYPs and UGTs contributions (right).

- - 1. Sensitivity Analysis for Ethinylestradiol Model

The results of the one-way sensitivity analysis with AUC and C_max_ as outcome parameters are shown in Figure S2.36 and Figure S2.37, respectively. The standard set of PK-Sim model parameters visible in simple view (N=225, including solubility, metabolism by enzyme, organ volumes and blood flows) were investigated.


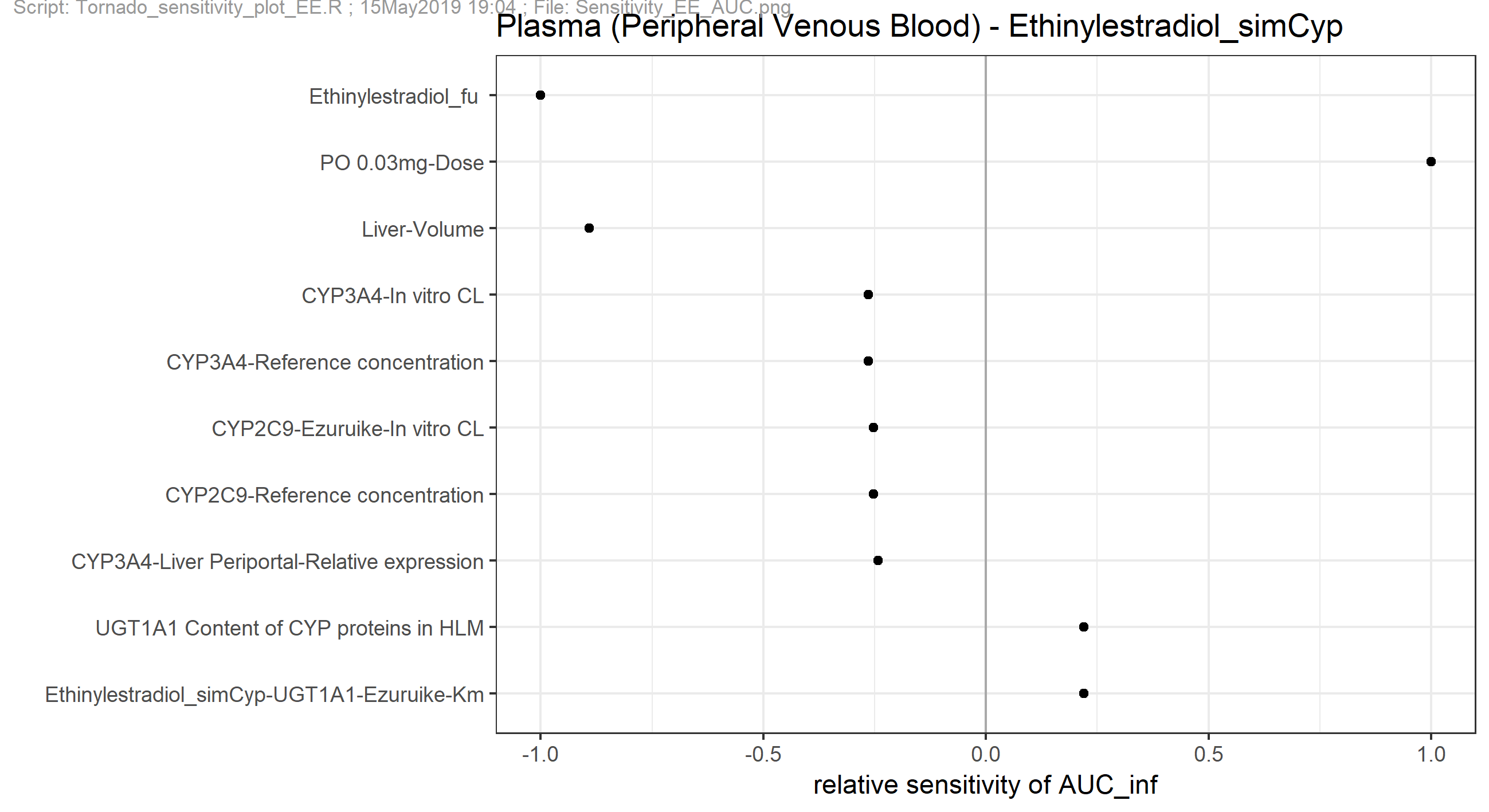


Figure S2.36 Sensitivity analysis ethinylestradiol AUC (0 to infinity)


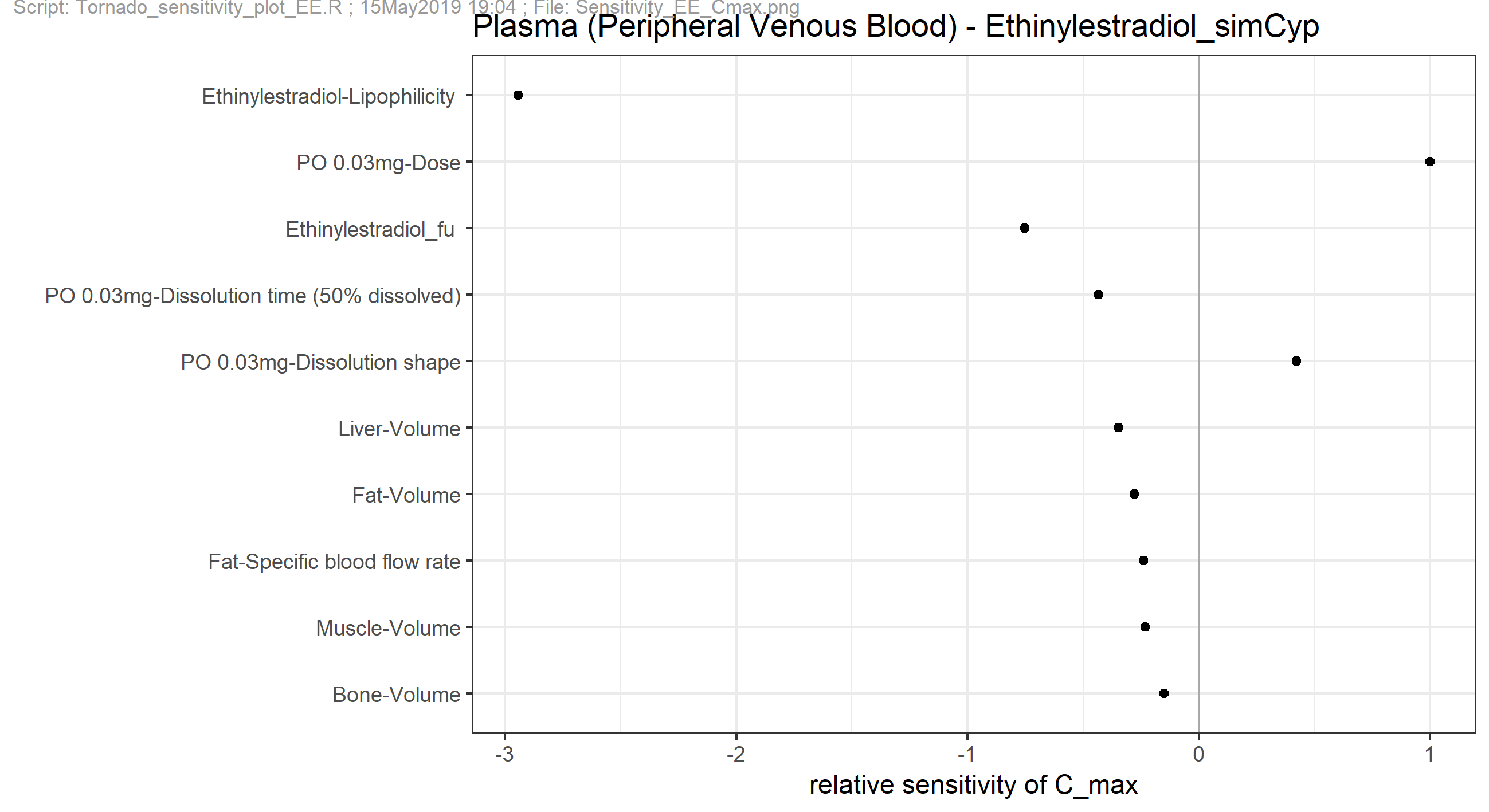


Figure S2.37 Sensitivity analysis ethinylestradiol C_max_

Lipophilicity (logP) has a high impact on the PK predictions for ethinylestradiol C_max_. In the current model we estimated logP to 3.48. Other sources report a very similar value (e.g DrugBank: 3.63 – 3.9). Therefore, it may be concluded that the lipophilicity used in the PBPK model is unlikely to be substantially different from reality.

The liver volume is also very influential on AUC as it impacts the total body clearance. The value used for the liver volume is a function of body weight in PK-Sim. Given that this is a validated default parameter in PK-Sim backed by numerous literature sources, substantial bias in this value is not expected.

As can be expected, the formulation parameters also affect C_max_. These have been estimated in a simultaneous i.v. and p.o. fit, together with total hepatic clearance and intestinal permeability. Note that any change in one of these parameters would lead to different estimates of the other parameters, in order to match the observations.

The parameters relating to CYP3A4, CYP2C9 and UGT1A1 metabolism (reference concentration, intrinsic CL, relative expression in liver periportal) are also influential on the PK. These parameters were derived from a previously developed PBPK model[^36^](#_ENREF_36). Using estimations for total hepatic clearance led to similar predictions.

Fraction unbound influences distribution but also body clearance as fu determines the fraction of compound available for metabolism. The current value (0.03) was obtained from DrugBank and similar values are reported in the label (0.01–0.025).

Muscle, fat, liver volume and blood flow rate are also influential on C_max_. These are validated default parameters in PK-Sim backed by numerous literature sources and substantial bias in these values are not expected.

In summary, findings from the sensitivity analysis were in line with expectations, i.e. that these parameters influence the predicted AUC and C_max_. Attention should be payed to the formulation parameters.

# **SECTION S2.8. Glossary of abbreviations used in this document**

| ADME | Absorption, Distribution, Metabolism, Excretion |
| --- | --- |
| AUC | Area under the plasma concentration versus time curve |
| AUCR | Area under the plasma concentration versus time curve Ratio |
| b.i.d. | Twice daily (bis in diem) |
| BMI | Body mass index |
| CL | Clearance |
| Clint | Intrinsic liver clearance |
| C_max_ | Maximum concentration |
| C_maxR_ | Maximum concentration Ratio |
| CYP | Cytochrome P450 oxidase |
| CYP1A2 | Cytochrome P450 1A2 oxidase |
| CYP2C19 | Cytochrome P450 2C19 oxidase |
| CYP2D6 | Cytochrome P450 2D6 oxidase |
| CYP3A4 | Cytochrome P450 3A4 oxidase |
| DDI | Drug-drug interaction |
| EE | Ethinylestradiol |
| EM | Extensive metabolizers |
| fm | Fraction metabolized |
| FMO | Flavin-containing monooxygenase |
| fu | Fraction unbound |
| FDA | Food and Drug administration |
| GFR | Glomerular filtration rate |
| HLM | Human liver microsomes |
| i.v. | Intravenous |
| IVIVE | In Vitro to In Vivo Extrapolation |
| Kcat | Catalyst rate constant |
| Ki | Inhibitor constant |
| Kinact | Rate of enzyme inactivation |
| Km | Michaelis Menten constant |
| OSP | Open Systems Pharmacology |
| PBPK | Physiologically-based pharmacokinetics |
| PK | Pharmacokinetics |
| PM | Poor metabolizers |
| RT-PCR | Reverse transcription polymerase chain reaction |
| p.o. | Per os |
| q.d. | Once daily (quaque diem) |
| s.d. | Single Dose |
| SPC | Summary of Product Characteristics |
| SD | Standard deviation |
| TDI | Time dependent inhibition |
| t.i.d. | Three times a day (ter in die) |
| T_max_ | Time to reach C_max_ |
| UGT | Uridine 5'-diphospho-glucuronosyltransferase |
| Vmax | Vmax: maximum enzymatic rate achieved by the system at saturating substrate concentration |

# **SECTION S2.9. REFERENCES**

1. Backman J.T., Schroder M.T., Neuvonen P.J. Effects of gender and moderate smoking on the pharmacokinetics and effects of the CYP1A2 substrate tizanidine. *Eur J Clin Pharmacol* **64** 17-24. (2008)

2. Backman J.T., Granfors M.T., Neuvonen P.J. Rifampicin is only a weak inducer of CYP1A2-mediated presystemic and systemic metabolism: studies with tizanidine and caffeine. *Eur J Clin Pharmacol* **62** 451-461. (2006)

3. Al-Ghazawi M., Alzoubi M., Faidi B. Pharmacokinetic comparison of two 4 mg tablet formulations of tizanidine. *Int J Clin Pharmacol Ther* **51** 255-262. (2013)

4. Granfors M.T., Backman J.T., Neuvonen M., Ahonen J., Neuvonen P.J. Fluvoxamine drastically increases concentrations and effects of tizanidine: a potentially hazardous interaction. *Clin Pharmacol Ther* **75** 331-341. (2004)

5. Momo K.*, et al.* Effects of mexiletine, a CYP1A2 inhibitor, on tizanidine pharmacokinetics and pharmacodynamics. *J Clin Pharmacol* **50** 331-337. (2010)

6. Henney H.R., 3rd, Shah J. Relative bioavailability of tizanidine 4-mg capsule and tablet formulations after a standardized high-fat meal: a single-dose, randomized, open-label, crossover study in healthy subjects. *Clin Ther* **29** 661-669. (2007)

7. Shah J., Wesnes K.A., Kovelesky R.A., Henney H.R., 3rd. Effects of food on the single-dose pharmacokinetics/pharmacodynamics of tizanidine capsules and tablets in healthy volunteers. *Clin Ther* **28** 1308-1317. (2006)

8. Tse F.L., Jaffe J.M., Bhuta S. Pharmacokinetics of orally administered tizanidine in healthy volunteers. *Fundam Clin Pharmacol* **1** 479-488. (1987)

9. Shellenberger M.K., Groves L., Shah J., Novack G.D. A controlled pharmacokinetic evaluation of tizanidine and baclofen at steady state. *Drug Metab Dispos* **27** 201-204. (1999)

10. Granfors M.T., Backman J.T., Laitila J., Neuvonen P.J. Tizanidine is mainly metabolized by cytochrome p450 1A2 in vitro. *Br J Clin Pharmacol* **57** 349-353. (2004)

11. Accorda Therapeutics Inc. Zanaflex prescribing information. 2013 [cited 2019 25 November 2019]Available from: <https://www.accessdata.fda.gov/drugsatfda_docs/label/2013/021447s011_020397s026lbl.pdf>

12. Drugs.com. Mexiletine. 2109 1 October 2018 [cited 26 November 2019]Available from: drugs.com/pro/mexiletine.html

13. Pringle T.*, et al.* Dose independent pharmacokinetics of mexiletine in healthy volunteers. *Br J Clin Pharmacol* **21** 319-321. (1986)

14. Labbe L.*, et al.* Pharmacokinetic and pharmacodynamic interaction between mexiletine and propafenone in human beings. *Clin Pharmacol Ther* **68** 44-57. (2000)

15. Pentikainen P.J., Halinen M.O., Helin M.J. Pharmacokinetics of intravenous mexiletine in patients with acute myocardial infarction. *J Cardiovasc Pharmacol* **6** 1-6. (1984)

16. Campbell N.P., Kelly J.G., Adgey A.A., Shanks R.G. The clinical pharmacology of mexiletine. *Br J Clin Pharmacol* **6** 103-108. (1978)

17. Begg E.J., Chinwah P.M., Webb C., Day R.O., Wade D.N. Enhanced metabolism of mexiletine after phenytoin administration. *Br J Clin Pharmacol* **14** 219-223. (1982)

18. Kusumoto M.*, et al.* Lack of pharmacokinetic interaction between mexiletine and omeprazole. *Ann Pharmacother* **32** 182-184. (1998)

19. Joeres R., Klinker H., Heusler H., Epping J., Richter E. Influence of mexiletine on caffeine elimination. *Pharmacol Ther* **33** 163-169. (1987)

20. Kusumoto M.*, et al.* Effect of fluvoxamine on the pharmacokinetics of mexiletine in healthy Japanese men. *Clin Pharmacol Ther* **69** 104-107. (2001)

21. Campbell N.P., Kelly J.G., Adgey A.A., Shanks R.G. Mexiletine in normal volunteers. *Br J Clin Pharmacol* **6** 372-373. (1978)

22. Steere B., Baker J.A., Hall S.D., Guo Y. Prediction of in vivo clearance and associated variability of CYP2C19 substrates by genotypes in populations utilizing a pharmacogenetics-based mechanistic model. *Drug Metab Dispos* **43** 870-883. (2015)

23. Cooper C.*, et al.* Pharmacokinetic interactions of faldaprevir and deleobuvir and their individual and combined effect on selected cytochrome P450 probe substrates in patients infected with genotype-1 HCV. . *Hepatology* **58** 732A, Abstract 1083. (2013)

24. Sabo J.P.*, et al.* Interactions of the hepatitis C virus protease inhibitor faldaprevir with cytochrome P450 enzymes: in vitro and in vivo correlation. *J Clin Pharmacol* **55** 467-477. (2015)

25. Back D.J.*, et al.* The pharmacokinetics of levonorgestrel and ethynylestradiol in women - studies with Ovran and Ovranette. *Contraception* **23** 229-239. (1981)

26. Back D.J.*, et al.* An investigation of the pharmacokinetics of ethynylestradiol in women using radioimmunoassay. *Contraception* **20** 263-273. (1979)

27. Orme M., Back D.J., Ward S., Green S. The pharmacokinetics of ethynylestradiol in the presence and absence of gestodene and desogestrel. *Contraception* **43** 305-316. (1991)

28. Goebelsmann U., Hoffman D., Chiang S., Woutersz T. The relative bioavailability of levonorgestrel and ethinyl estradiol administered as a low-dose combination oral contraceptive. *Contraception* **34** 341-351. (1986)

29. Stanczyk F.Z.*, et al.* Plasma levels and pharmacokinetics of norethindrone and ethinylestradiol administered in solution and as tablets to women. *Contraception* **28** 241-251. (1983)

30. Zhang C.*, et al.* An open-label, two-period comparative study on pharmacokinetics and safety of a combined ethinylestradiol/gestodene transdermal contraceptive patch. *Drug Des Devel Ther* **11** 725-731. (2017)

31. Kothare P.A.*, et al.* Effect of exenatide on the pharmacokinetics of a combination oral contraceptive in healthy women: an open-label, randomised, crossover trial. *BMC Clin Pharmacol* **12** 8. (2012)

32. Timmer C.J., Mulders T.M. Pharmacokinetics of etonogestrel and ethinylestradiol released from a combined contraceptive vaginal ring. *Clin Pharmacokinet* **39** 233-242. (2000)

33. Granfors M.T., Backman J.T., Laitila J., Neuvonen P.J. Oral contraceptives containing ethinyl estradiol and gestodene markedly increase plasma concentrations and effects of tizanidine by inhibiting cytochrome P450 1A2. *Clin Pharmacol Ther* **78** 400-411. (2005)

34. Balogh A.*, et al.* Influence of ethinylestradiol-containing combination oral contraceptives with gestodene or levonorgestrel on caffeine elimination. *Eur J Clin Pharmacol* **48** 161-166. (1995)

35. Kuhnz W., Humpel M., Biere H., Gross D. Influence of repeated oral doses of ethinyloestradiol on the metabolic disposition of [13C2]-ethinyloestradiol in young women. *Eur J Clin Pharmacol* **50** 231-235. (1996)

36. Ezuruike U.*, et al.* Risk-Benefit Assessment of Ethinylestradiol Using a Physiologically Based Pharmacokinetic Modeling Approach. *Clin Pharmacol Ther* **104** 1229-1239. (2018)

37. Martin P.*, et al.* Effects of Fostamatinib on the Pharmacokinetics of Oral Contraceptive, Warfarin, and the Statins Rosuvastatin and Simvastatin: Results From Phase I Clinical Studies. *Drugs R D* **16** 93-107. (2016)

38. Sidhu J., Job S., Singh S., Philipson R. The pharmacokinetic and pharmacodynamic consequences of the co-administration of lamotrigine and a combined oral contraceptive in healthy female subjects. *Br J Clin Pharmacol* **61** 191-199. (2006)

39. Stockis A., Watanabe S., Fauchoux N. Interaction between brivaracetam (100 mg/day) and a combination oral contraceptive: a randomized, double-blind, placebo-controlled study. *Epilepsia* **55** e27-31. (2014)

40. Wiesinger H.*, et al.* Pharmacokinetic interaction between the CYP3A4 inhibitor ketoconazole and the hormone drospirenone in combination with ethinylestradiol or estradiol. *Br J Clin Pharmacol* **80** 1399-1410. (2015)

41. Wang B., Sanchez R.I., Franklin R.B., Evans D.C., Huskey S.E. The involvement of CYP3A4 and CYP2C9 in the metabolism of 17 alpha-ethinylestradiol. *Drug Metab Dispos* **32** 1209-1212. (2004)

42. Shiraga T., Niwa T., Ohno Y., Kagayama A. Interindividual variability in 2-hydroxylation, 3-sulfation, and 3-glucuronidation of ethynylestradiol in human liver. *Biol Pharm Bull* **27** 1900-1906. (2004)

43. Stanczyk F.Z., Archer D.F., Bhavnani B.R. Ethinyl estradiol and 17beta-estradiol in combined oral contraceptives: pharmacokinetics, pharmacodynamics and risk assessment. *Contraception* **87** 706-727. (2013)

44. Karjalainen M. Inhibition of CYP1A2-mediated drug metabolism in vitro and in humans: With special emphasis on rofecoxib and other NSAIDs. University of Helsinki, Finland, 2008.

45. Boehringer Ingelheim. Study c13608215. A study to investigate the pharmacokinetic drug-drug interaction following oral administration of ethinylestradiol/levonorgestrel (Microgynon®) and BI 409306 in healthy Korean premenopausal female subjects (an open-label, two-period, fixed-sequence study).
