# Supplementary material for: A Physiologically-Based Pharmacokinetic (PBPK) Model Network for the Prediction of CYP1A2 and CYP2C19 Drug–Drug–Gene Interactions with Fluvoxamine, Omeprazole, S-mephenytoin, Moclobemide, Tizanidine, Mexiletine, Ethinylestradiol, and Caffeine
Source: Pharmaceutics. 2020 Dec 8;12(12):1191. doi: 10.3390/pharmaceutics12121191 (PMC7764797; doi:10.3390/pharmaceutics12121191)
Supplement: Supplementary file 1 [file pharmaceutics-12-01191-s001.zip › pharmaceutics-1010984 supplementary/PBPK_manuscript_supplement S3_Pharmaceutics.docx]

**Electronic Supplementary Material**

# **Supplement S3:** **Final parameters and structure of each model and results of DDI predictions**

- 1. Fluvoxamine Model
     1. Final Model Parameters and Structure

The final fluvoxamine model consisted of the following key aspects:

- Tissue partition coefficients calculated according to Rodgers & Rowland
- Renal elimination (GFR fraction=1)
- Linear CYP1A2 metabolism
- Non-linear CYP2D6 metabolism

The final parameters of the fluvoxamine PBPK model template are shown in Table S3.1.

Table S3.1 Final parameters of the fluvoxamine model

| **Parameter** | **Value** | **Source** | **Assumptions/ Comment** |
| --- | --- | --- | --- |
| MW (free base // maleate) | 318.3 g/mol // 434.4 g/mol | DrugBank | Dose of commercial product usually refers to amount of fluvoxamine maleate – conversion factor is 0.733 |
| Partition coefficient | Rodgers and Rowland | (estimated) | Various distribution models available in PK-Sim were explored and compared |
| Specific intestinal permeability (transcellular) | 1.23E-5 cm/min | Estimated |  |
| Aqueous solubility at pH 7.4 | 0.062 mg/mL | PubChem |  |
| Dissolution time/ shape (Weibull) | 10 min / 0.92 | Fixed | Fast dissolution; simulations not sensitive to parameter change |
| Dissolution lag-time (for enteric coated tablets) | 30 min | Assumed | Represents gastric emptying time |
| LogP | 3.38 | Alqahtani 2016[^1^](#_ENREF_1) | Similar values in other sources (e.g. DrugBank: 3.2) |
| fu | 0.23 | Alqahtani 2016[^1^](#_ENREF_1) | Similar value in label: 0.2 |
| pKa | Base/9.16 | Alqahtani 2016[^1^](#_ENREF_1) |  |
| Km_2D6 | 9.77E-4 µmol/l | Estimated | Saturable metabolism; supported by literature (dose non-proportionality, other PBPK models) |
| Kcat_2D6 | 0.04 1/min | Estimated | See above |
| Microsomal clearance 1A2 | 0.54 µl/min/pmol rec. enzyme | Estimated | Metabolism via CYP1A2 non-saturable in the relevant concentration range |
| CYP 2D6 / 1A2 expression | - | PK-Sim database |  |
| Renal elimination | 1.0 GFR fraction |  |  |
| Ki_CYP1A2 | 2.97 nmol/L | Iga 2016[^2^](#_ENREF_2) | Unbound value from in vivo experiments; presumably the most robust source of Ki as it eliminates the need for in vitro- in vivo scaling |

- - 1. DDI Predictions with Caffeine as Victim Drug

Fluvoxamine is a strong inhibitor of CYP1A2 and increases the AUC of caffeine by 7- to 14-fold.

A dynamic DDI simulation was conducted with fluvoxamine as CYP1A2 inhibitor and caffeine as victim, and compared to two previously published clinical studies. For caffeine, the template model included in PK-Sim[^3^](#_ENREF_3) was used.

In Jeppesen et al. 1996[^4^](#_ENREF_4), subjects in one group received 50 mg fluvoxamine-maleate on the first 4 days followed by 100 mg q.d. for 8 days. On day 8 they received a single dose of 200 mg caffeine. The other group received caffeine without fluvoxamine co-treatment. The observed mean concentrations in both groups are shown in Figure S3.1 together with the simulations. The model predictions are in agreement with the observations and predicted ratios within 2-fold of the observed ratios. Table S3.2 shows the ratios of caffeine AUC and C_max_ with and without co-administration of fluvoxamine.

Figure S3.1 Predicted and observed caffeine concentrations, with and without co-administration of fluvoxamine.

Observed data: Jeppesen et al. 1996.

Table S3.2 Observed (Jeppesen et al 1996) and predicted AUC and C_max_ ratios for caffeine with/without fluvoxamine

|  |  | **AUCR** | **C_max_R** |
| --- | --- | --- | --- |
| 200 mg Caffeine s.d. + 100 mg Fluvoxamine q.d. | Observed | 7.16 | 1.09 |
|  | Predicted | 10.00 | 1.10 |
| Ratio pred/obs |  | 1.40 | 1.01 |

The second study is reported by Culm-Merdek et al. 2005[^5^](#_ENREF_5), where seven healthy subjects received single 250 mg dose of caffeine (or matching placebo) together with fluvoxamine (four doses of 100 mg over 2 days) or with matching placebo in a cross-over fashion.

As can be seen in Figure S3.2, the caffeine levels with co-administered fluvoxamine were underpredicted. Notice however, that the pre-dose concentrations of caffeine were not 0 in the test group (green dot at 24 h).


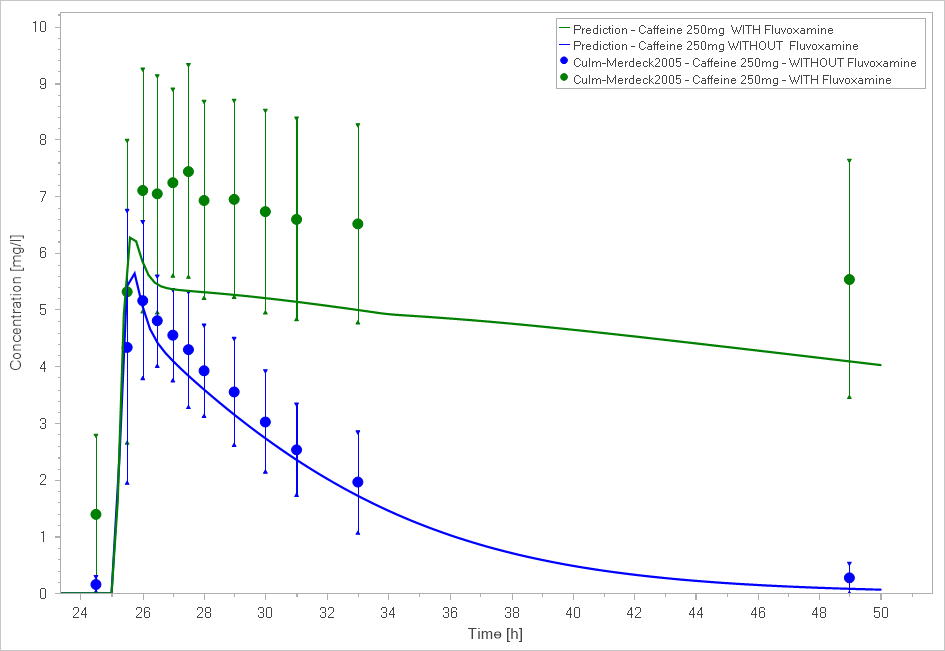


Figure S3.2 Predicted and observed caffeine concentrations, with and without co-administration of fluvoxamine. Observed data: Culm-Merdek et al. 2005.

It may be hypothesised that subjects may not have refrained completely from caffeine-containing beverages before the test period. To investigate this, a simulation was conducted where a dose of 100 mg caffeine (corresponding approximately to the caffeine content of one cup of coffee[^6^](#_ENREF_6)) was given 24 hours before the administration of caffeine-tablets as per the study protocol. The simulations shown in Figure S3.3 support this hypothesis.

Hence it was not deemed necessary to adjust the underlying caffeine or fluvoxamine models but rather conclude that the clinical study reported by Culm-Merdek^[5](#_ENREF_5)^ potentially was facing issues with subjects not compliant with the protocol rules and drinking coffee the morning before the study day.

When assuming this small initial dose of caffeine, the model predicts the interaction between caffeine and fluvoxamine for the Culm-Merdek data within 2-fold as well (Figure S3.3, Table S3.3).


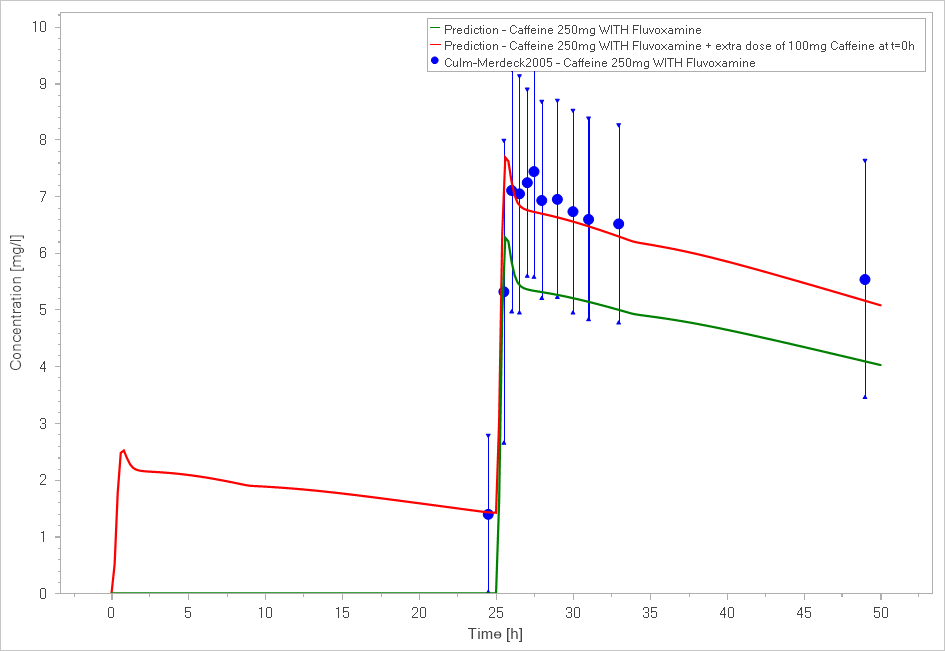


Figure S3.3 Simulation of caffeine levels with co-administration of fluvoxamine and with or without a cup of coffee (100 mg) before the actual dosing.

Table S3.3 Observed (Culm-Merdek 2005) and predicted AUC and C_max_ ratios for caffeine with/without fluvoxamine, with extra cup of coffee

|  |  | **AUCinf**  **(μg.h/mL)** | **AUCR** | **C_max_ (μg/mL)** | **C_max_R** |
| --- | --- | --- | --- | --- | --- |
| 250 mg Caffeine s.d. | Observed | 46.3 |  | 5.81 |  |
|  | Predicted | 34.6 |  | 5.64 |  |
| 250 mg Caffeine s.d. + 100 mg Fluvoxamine b.i.d. | Observed | 635 | 13.71 | 8.14 | 1.40 |
|  | Predicted | 518 | 14.97 | 7.69 | 1.36 |
| Ratio pred/obs |  |  | 1.09 |  | 0.97 |

- 1. Omeprazole Model
     1. Final Model Parameters and Structure

The final R- and S- omeprazole models consisted of the following key aspects:

- Tissue partition coefficients calculated according to Rodgers & Rowland
- Renal elimination (0.000507 L/h/kg)
- Linear CYP2C19 metabolism
- Linear CYP3A4 metabolism
- Time-dependent auto-inhibition of CYP2C19

Adapted CYP2C19 expression in the GI tract

The final parameters for (R-/S-) omeprazole PBPK model template are shown in Table S3.4 and Table S3.5.

Table S3.4 Final parameters for S-omeprazole model

| **Parameter** | **Value** | **Source** | **Assumptions/ Comment** |
| --- | --- | --- | --- |
| MW | 345.4 g/mol | DrugBank |  |
| Partition coefficient model | Rodgers and Rowland | (estimated) | Various distribution models available in PK-Sim were explored and compared |
| Specific intestinal permeability (transcellular) | 9.79E-5 cm/min | Estimated |  |
| Aqueous solubility at pH 7.4 | 0.36 mg/mL | DrugBank |  |
| Dissolution time/ shape (Weibull) | 41.65 min / 1.02 | Estimated |  |
| Dissolution lag-time (for enteric coated tablets) | 30 min | Assumed | Represents gastric emptying time |
| LogP | 1.68 | Estimated | Similar values in other sources (e.g. DrugBank: 1.66) |
| Fu | 0.03 | DrugBank | Similar value in label |
| Compound type/pKa | Acid/9.29  Base/4.77 | DrugBank |  |
| Specific CL_2C19 | 13.98 1/min | Estimated |  |
| Specific CL_3A4 | 0.37 1/min | Estimated | See above |
| 3A4 expression | - | PK-Sim database |  |
| 2C19 expression | Adapted expression in GI tract | Olivares-Morales[^7^](#_ENREF_7) |  |
| Renal elimination | 0.000507 L/h/kg | Wu 2014[^8^](#_ENREF_8) |  |
| CYP2C19 time-dependent autoinhibition | Kinact: 5 1/h  Kinact__half_: 0.3 umol/L | Wu 2014[^8^](#_ENREF_8) | Both parameters not separately identifiable – fixed Kinact value to the one reported by Wu |

Table S3.5 Final parameters for R-omeprazole model

| **Parameter** | **Value** | **Source** | **Assumptions/ Comment** |
| --- | --- | --- | --- |
| MW | 345.4 g/mol | DrugBank |  |
| Partition coefficient model | Rodgers and Rowland | Assumed from S-omeprazole |  |
| Specific intestinal permeability (transcellular) | 9.79E-5 cm/min | Assumed from S-omeprazole |  |
| Aqueous solubility at pH 7.4 | 0.36 mg/mL | DrugBank |  |
| Dissolution time/shape (Weibull) | 41.65 min / 1.02 | Estimated |  |
| Dissolution lag-time (for enteric coated tablets) | 30 min | Assumed | Represents gastric emptying time |
| LogP | 1.68 | Assumed similar to S-omeprazole | Similar values in other sources (e.g DrugBank: 1.66)  LogP is identical between enantiomers |
| Fu | 0.04 | DrugBank | Assumed that of omeprazole |
| Compound type/pKa | Acid/9.29  Base/4.77 | DrugBank |  |
| Specific CL_2C19 | 50 1/min | Estimated |  |
| Specific CL_3A4 | 0.16 1/min | Estimated |  |
| CYP2C19 time-dependent autoinhibition | Kinact: 4 1/h  Kinact__half_: 1.6 umol/L | Wu 2014[^8^](#_ENREF_8) |  |
| 3A4 expression | - | PK-Sim database |  |
| 2C19 expression | Adapted expression in GI tract | Olivares-Morales 2016[^7^](#_ENREF_7) |  |
| Renal elimination | 0.000507 L/h/kg | Wu 2014[^8^](#_ENREF_8) |  |
| TDI CYP2C19 Kinact / Ki | 4.00 1/min / 1.6 umol/L | Wu 2014[^8^](#_ENREF_8) |  |

- - 1. DDI Predictions for Omeprazole Model Qualification
       1. **DDI Predictions with fluvoxamine as inhibitor**

A dynamic DDI simulation was conducted with fluvoxamine as CYP2C19 inhibitor and omeprazole as victim, and compared to literature data. The predefined typical Japanese subject (age=30 y, weight=61.87 kg, height=169 cm, BMI=21.67 kg/m^2^) was used with CYP3A4, CYP2C19, CYP2D6 and CYP1A2 expression from RT-PCR database in PK-Sim and adapted CYP2C19 expression in gut. To note, this adapted expression of CYP2C19 in gut is not affecting fluvoxamine concentrations as fluvoxamine is not metabolized by CYP2C19 (see [Section 3.1.1](#sec411)).

In Yasui-Furukori et al. 2004[^10^](#_ENREF_10), eighteen volunteers, of whom six were homozygous EM (hmEMs), six were heterozygous EM (htEMs) and six were PM for CYP2C19, received two six-day courses of either daily 50 mg fluvoxamine or placebo in a randomized fashion with a single oral 40 mg dose of omeprazole on day six in both cases. Plasma concentrations of omeprazole and its metabolites, 5-hydroxyomeprazole, omeprazole sulphone, and fluvoxamine were monitored up to 8 h after the dosing.

The observed mean concentrations in both hmEM and PM groups are shown in Figure S3.4 together with the simulations. The model predictions are in reasonable agreement with the observations (Table S3.6, Table S3.7) and show the ratios of omeprazole AUC and C_max_ with and without co-administration of fluvoxamine. Despite some model underprediction in both treatment and control groups, the predicted AUC and C_max_ ratios well match the observed ones. Fluvoxamine is a strong inhibitor of CYP2C19 and increases the AUC of omeprazole by ~6-fold in EM.

Table S3.6 Observed and predicted AUC and C_max_ ratios for omeprazole with or without fluvoxamine in EM

|  |  | **AUC**  **(ng.h/mL)** | **AUCR** | **C_max_ (ng/mL)** | **C_max_R** |
| --- | --- | --- | --- | --- | --- |
| 40 mg omeprazole | Observed | 1481 |  | 900 |  |
|  | Predicted | 1189 |  | 468 |  |
| 40 mg omeprazole+ 50 mg fluvoxamine 50 mg b.i.d. | Observed | 7911 | 5.34 | 3131 | 3.48 |
|  | Predicted | 7147 | 6.01 | 1625 | 3.47 |
| Ratio pred/obs |  |  | 1.13 |  | 1.00 |

It has to be noticed that while the fluvoxamine-Omeprazole DDI model re-qualification for OSP was conducted in version 9.1, the model had been built in previous PK-Sim versions where a bug was detected (<https://github.com/Open-Systems-Pharmacology/PK-Sim/issues/1523>) and fixed in version 9.1. This affects substantially the prediction of the interaction ratios in EM for this particular fluvoxamine-omeprazole combination (CmaxR pred/obs = 0.70 in version 9.1 versus 1.13 in previous versions and AUCR pred/obs = 0.58 versus 1.00 respectively). This issue was not found to affect substantially other models in this network.

Table S3.7 Observed and predicted AUC and C_max_ ratios for omeprazole with or without fluvoxamine in PM

|  |  | **AUC**  **(ng.h/mL)** | **AUCR** | **C_max_ (ng/mL)** | **C_max_R** |
| --- | --- | --- | --- | --- | --- |
| 40 mg omeprazole | Observed | 11537 |  | 2991 |  |
|  | Predicted | 7372 |  | 1639 |  |
| 40 mg omeprazole+ 50 mg fluvoxamine 50 mg b.i.d. | Observed | 13940 | 1.21 | 3352 | 1.12 |
|  | Predicted | 7372* | 1.00 | 1639* | 1.00 |
| Ratio pred/obs |  |  | 0.83 |  | 0.89 |

*No CYP2C19 activity in CYP2C19 PMs

| 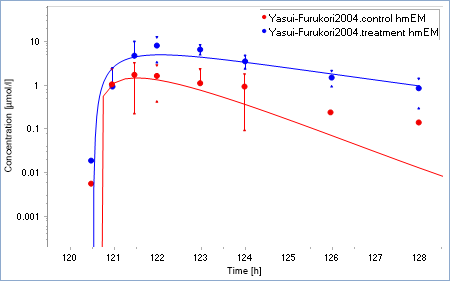 | 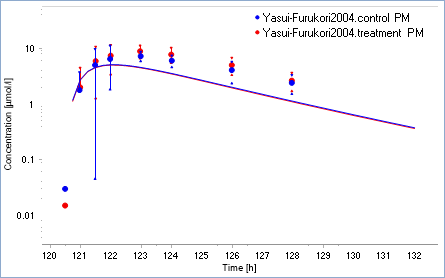 |
| --- | --- |

Figure S3.4 Predicted and observed omeprazole concentrations, with and without co-administration of fluvoxamine. Left: homozygous CYP2C19 extensive metabolizers (hmEM); right: CYP2C19 poor metabolizers (PM)[^11^](#_ENREF_11).

In order to match omeprazole C_max_ best when administered with an inhibitor, different Ki values were tested. As fluvoxamine has been found to be a CYP3A4 inhibitor (in addition to CYP1A2 and CYP2C19) the inclusion of a second competitive inhibition mechanism on CYP3A4 was tested. Initially, the same Ki value for CYP2C19 was assumed, i.e., 3.6 nM^[2](#_ENREF_2)^ (first row of Table S3.8 and Table S3.9). Then, the fluvoxamine-midazolam CYP3A4 Ki, corrected for fu-mic from literature was used[^12^](#_ENREF_12). In particular, a fu-mic of 0.807 was used to convert total to unbound concentrations in HLM. Fu-mic was estimated in PK-Sim as *min(1/(1-M_prot)+10^LogMA*M_prot))* where *M_prot* is the amount of microsomal protein (0.1 mg/mL) as reported in the Open Systems Pharmacology Suite Manual[^13^](#_ENREF_13) and *LogMA* is the estimated lipophilicity value for fluvoxamine.

Lastly, two differential Ki values were used for CYP2C19 inhibition on R- and S-omeprazole, with CYP2C19 Ki of 1.63 and 0.37 nM, respectively. These two parameters have been determined experimentally in Foti 2008[^14^](#_ENREF_14) by incubation of fluvoxamine with (S)-omeprazole and (R)-omeprazole, separately. As shown in Table S3.8 and Table S3.9, none of these approaches led to improvement in model predictions and differences were small. Hence, inhibition by fluvoxamine was kept on CYP2C19 only, which resulted in the AUCR and C_max_R previously presented in Table S3.6 and Table S3.7.

Table S3.8 Observed and predicted C_max_ ratios for omeprazole-fluvoxamine interaction using different Ki values

| Ki nM ^Source^ | R-omeprazole  (ng/mL) | S-omeprazole  (ng/mL) | Omeprazole  (R+S)  (ng/mL) | Ratio C_max_  Current Ki/final Ki | C_max_ obs/pred |
| --- | --- | --- | --- | --- | --- |
| CYP2C19 Ki =3.6[^2^](#_ENREF_2)  CYP3A4 Ki=3.6 [assumed] | 544.3 | 1424.2 | 1969 | **1.21** | 1.59 |
| CYP2C19 Ki =3.6[^2^](#_ENREF_2)  CYP3A4 Ki=2097[^12^](#_ENREF_12) | 827.25 | 923.1 | 1750 | **1.08** | 1.79 |
| CYP2C19 Ki_(S-ome)_=1.63[^14^](#_ENREF_14)  CYP2C19 Ki_(R-ome)_=0.37[^14^](#_ENREF_14) | 792.7 | 829.0 | 1622 | **1.00** | 1.93 |

Table S3.9 Observed and predicted AUC ratios for omeprazole-fluvoxamine interaction using different Ki values

| Ki nM ^Source^ | R-omeprazole  (ng.h/mL) | S-omeprazole  (ng.h/mL) | Omeprazole  (R+S)  (ng.h/mL) | Ratio AUC  Current Ki/final Ki | AUC obs/pred |
| --- | --- | --- | --- | --- | --- |
| CYP2C19 Ki =3.6[^2^](#_ENREF_2)  CYP3A4 Ki=3.6 [assumed] | 8198 | 9047 | 17245 | **2.41** | 0.46 |
| CYP2C19 Ki =3.6[^2^](#_ENREF_2)  CYP3A4 Ki=2097[^12^](#_ENREF_12) | 3543 | 4044 | 7587 | **1.06** | 1.04 |
| CYP2C19 Ki_(S-ome)_=1.63[^14^](#_ENREF_14)  CYP2C19 Ki_(R-ome)_=0.37[^14^](#_ENREF_14) | 3189 | 3107 | 6296 | **0.88** | 1.26 |

- - - 1. **DDI Predictions with moclobemide**

A dynamic DDI simulation was conducted with moclobemide and omeprazole, and compared to literature data. Both compounds act as CYP2C19 inhibitors and victims. The predefined typical Japanese subject (age=30 y, weight=61.87 kg, height=169 cm, BMI=21.67 kg/m^2^) was used with CYP3A4, CYP2C19, CYP2D6 and CYP1A2 expressions from RT-PCR database in PK-Sim and adapted CYP2C19 expression in gut. Although moclobemide is also metabolized by CYP2C19, it has been shown that the adapted expression of CYP2C19 in gut had minimal impact on its PK (see Supplement 2a, Section 2.4.2.5).

In Cho et al. 2002[^15^](#_ENREF_15), sixteen volunteers, of whom eight were EM and eight were PM for CYP2C19, received oral doses of 40 mg omeprazole with or without 300 mg moclobemide coadministration.

The pharmacokinetic change of omeprazole, omeprazole sulphone and 5-hydroxyomeprazole concentrations were assessed to test for an interaction between omeprazole and moclobemide.

The observed mean concentrations in both EM and PM groups are shown in Figure S3.5 together with the simulations. The model predictions are in reasonable agreement with the observations. Table S3.10 and Table S3.11 show the ratios of omeprazole AUC and C_max_ with and without co-administration of moclobemide. Moclobemide is a moderate inhibitor of CYP2C19 and increases the AUC of omeprazole by ~2-fold.

Table S3.10 Observed and predicted AUC and C_max_ ratios for omeprazole after coadministration with moclobemide in EM

|  |  | **AUC**  **(ng.h/mL)** | **AUCR** | **C_max_ (ng/mL)** | **C_max_R** |
| --- | --- | --- | --- | --- | --- |
| 40 mg omeprazole | Observed | 1834 |  | 986.6 |  |
|  | Predicted | 1374 |  | 513.4 |  |
| 40 mg omeprazole + 300 mg moclobemide | Observed | 3760 | **2.05** | 1649 | **1.67** |
|  | Predicted | 2231 | **1.62** | 661.4 | **1.29** |
| Ratio pred/obs |  |  | **0.79** |  | **0.77** |

Table S3.11 Observed and predicted AUC and C_max_ ratios for omeprazole after coadministration with moclobemide in PM

|  |  | **AUC**  **(ng.h/mL)** | **AUCR** | **C_max_ (ng/mL)** | **C_max_R** |
| --- | --- | --- | --- | --- | --- |
| 40 mg omeprazole | Observed | 10425 |  | 2652 |  |
|  | Predicted | 9211 |  | 1738 |  |
| 40 mg omeprazole + 300 mg moclobemide | Observed | 12138 | **1.16** | 2609 | **0.98** |
|  | Predicted | 9211 | **1.00** | 1738 | **1.00** |
| Ratio pred/obs |  |  | **0.86** |  | **1.02** |

| 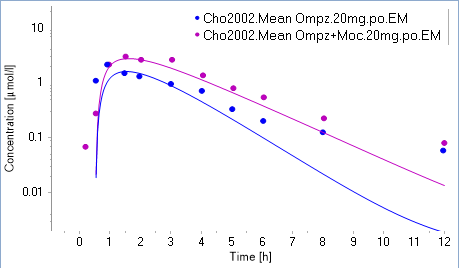 | 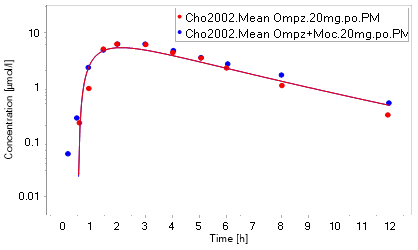 |
| --- | --- |

Figure S3.5 Predicted and observed omeprazole concentrations in CYP2C19 EM (left) and PM (right), with and without co-administration of moclobemide.

Observed data: Cho et al. 2002.

- 1. S-Mephenytoin Model
     1. Final Model Parameters and Structure

The final S-mephenytoin model consisted of the following key aspects:

- Tissue partition coefficients calculated according to Rodgers & Rowland
- Renal elimination (GFR fraction=1)
- Linear CYP2C19 metabolism from IVIVE
- Linear CYP2C9 metabolism from IVIVE

The final parameters of the S-mephenytoin PBPK model template are shown in Table S3.12.

Table S3.12 Final parameters of the S-mephenytoin model

| **Parameter** | **Value** | **Assumption/comment** |
| --- | --- | --- |
| Lipophilicity | 1.69 | LogP measured^[16](#_ENREF_16)^ |
| Distribution partitioning model | Rodgers and Rowland |  |
| CYP2C19 intrinsic clearance [ml/min] | 1986.9 | CL/F from Adedoyin et al.[^17^](#_ENREF_17) |
| CYP2C9 in vitro CL for liver Microsomes [µl/min/mg mic. protein] | 0.390 | Clint CYP2C9 from Steere et al.[^18^](#_ENREF_18) using 96 pmol CYP2C9 per mg mic. protein |
| Renal Elimination | 1.0 GFR fraction |  |
| P intest. (Transcellular) [cm/min] | 2.33E-5 | Default calculated by PK-Sim from LogP |
| Solubility [mg/ml] | 1270 | Water,  Website drugbank.ca/drugs/DB00532 |
| pKa | Acid/8.51 | Website drugbank.ca/drugs/DB00532 |
| fu | 0.702 | Measured^[18](#_ENREF_18)^ |
| Ki [nM] used in DDI predictions | 2.6 | in vivo, unbound from Iga et al.[^2^](#_ENREF_2) |
| geoSD CYP2C19 variability | 2.25 | Derived from CL/F variability from Olivares-Morales[^7^](#_ENREF_7) |

- - 1. DDI Predictions for S-Mephenytoin Model Qualification

A dynamic DDI simulation with coupled fluvoxamine template (as described in Section 3.1.1) was used to predict the effect of a strong CYP2C19 inhibitor. Predictions were compared to clinical results from Yao et al 2003[^19^](#_ENREF_19), where the effect of different fluvoxamine doses in S-mephenytoin was investigated. A simulation in a population with 2000 virtual individuals according to the biometrics of the individuals (12 males and females, 23-49 years) used in the Yao study was run to investigate the expected variability of S-mephenytoin and fluvoxamine plasma concentrations.

Figure S3.6 shows that the measured means and standard deviation of C_max_ for S-mephenytoin and the average concentrations for fluvoxamine are in good agreement with the prediction. Similar results were obtained for the 45.8 and 64.1 mg dose (not shown). Of note, the simulation was done with the free base of fluvoxamine, assuming that the dose value given in the study was for fluvoxamine maleate.

The Ki value is a critical parameter for predicting the extent of DDIs. Ki values can be generated from in vitro systems or in vivo data. However, the in vitro generated Ki is often not predictive for in vivo[^19^](#_ENREF_19). Yao et al 2003 investigated the gaps between in vitro and in vivo Ki values. The predictivity of Ki values from various sources were explored with the S‑mephenytoin-fluvoxamine coupled DDI model. The in vivo, unbound Ki from Yao 2003[^19^](#_ENREF_19) and the Ki recalculated from in vivo data by Iga 2016[^2^](#_ENREF_2) are in a similar nM range and lead both to predictions close to measured AUCR and C_max_ of 9.89 and 2.42, respectively (Table S3.13). Eventually, the Ki from Iga 2016 was chosen for further model qualification.

Table S3.13 Predicted AUC and C_max_ ratios for S-Mephenytoin with 64.1 mg fluvoxamine using various Ki values. Red: selected for further model qualification

| **Source** | **Ki Type** | **Ki [nM]** | **SD** | **AUCR** | **Pred/obs**  **AUCR** | **C_max_R** | **Pred/obs C_max_R** |
| --- | --- | --- | --- | --- | --- | --- | --- |
| Observed |  |  |  | 9.89 |  | 2.42 |  |
| Yao 2003[^19^](#_ENREF_19) | in vitro, total | 235 | 51 | 1.28 | 0.13 | 1.22 | 0.50 |
| Yao 2003[^19^](#_ENREF_19) | in vitro, unbound | 70 | - | 1.76 | 0.18 | 1.53 | 0.63 |
| Yao 2003[^19^](#_ENREF_19) | in vivo, total | 13.5 | 5.6 | 3.95 | 0.40 | 2.35 | 0.97 |
| Yao 2003[^19^](#_ENREF_19) | in vivo, unbound | 1.9 | 1.1 | 11.76 | 1.19 | 3.30 | 1.36 |
| Iga 2016[^2^](#_ENREF_2) | Parameter identification to fit in vivo data | 2.6 | - | 10.17 | 1.03 | 3.21 | 1.33 |

*
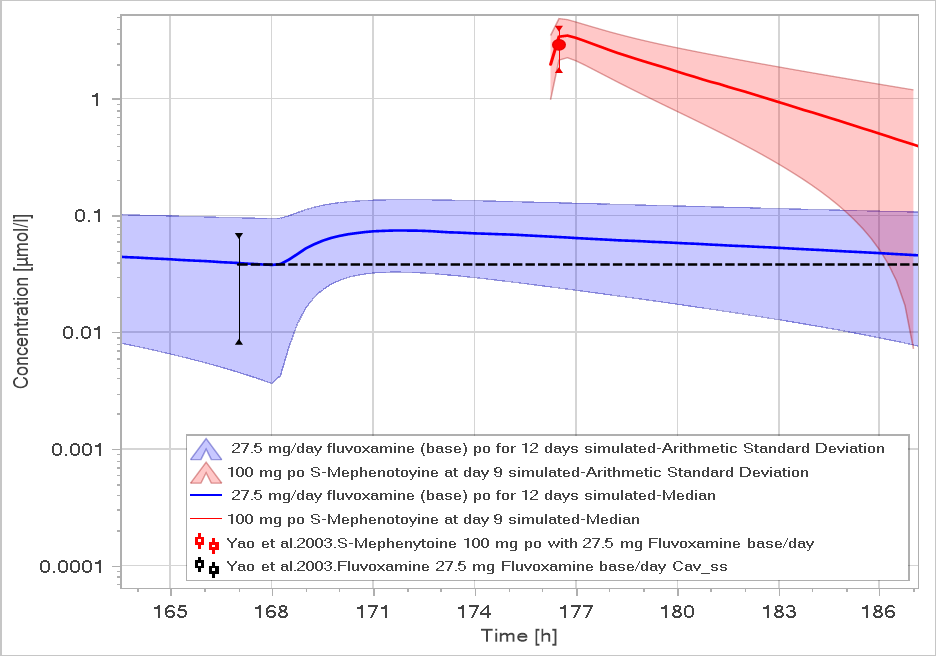
*

Figure S3.6 Simulated S-mephenytoin and Fluvoxamine plasma profiles in 2000 healthy males and females*.*

*The red line is the simulated arithmetic mean after a 100 mg S-Mephenytoin dose given concurrent to multiple doses of 27.5 mg fluvoxamine (blue line). The Ki value from Iga 2016*[*^2^*](#_ENREF_2) *was used for predictions*

As dose-dependent metabolism processes were included in the fluvoxamine model it was important to investigate if the AUCR and C_max_R could be predicted correctly with the PBPK-DDI model over a range of typical used doses for human (27.5 to 64.1 mg calculated as free base). Using the Ki from Iga et al.[^2^](#_ENREF_2), DDI predictions at several doses of fluvoxamine were simulated. As shown in Table S3.14, the predictions of AUC and C_max_ ratios were very close to the observed values, especially for the lower fluvoxamine doses. Predictions tend to get slightly overpredicted at higher doses, at least for C_max_R.

Table S3.14 Comparison of mean predicted AUC and C_max_ ratios for S-mephenytoin in presence of various dose levels of fluvoxamine.

Fluvoxamine was dosed OD for 12 days. At the 9^th^ day a dose of 100 mg S-Mephenytoin was given. The Ki value from Iga 2016[^2^](#_ENREF_2) was used for predictions

| **Dose Fluvoxamine (base) [mg]** | **Parameter** | **observed  Yao et al 2003** | **predicted   with Ki_Iga2016** | **Ratio pred/ obs** |
| --- | --- | --- | --- | --- |
| 27.5 | AUCR | 4.64 | 4.63 | 1.00 |
| 45.8 | AUCR | 6.70 | 7.85 | 1.17 |
| 64.1 | AUCR | 9.89 | 10.17 | 1.03 |
| 27.5 | C_max_R | 2.12 | 2.56 | 1.21 |
| 45.8 | C_max_R | 2.40 | 3.02 | 1.26 |
| 64.1 | C_max_R | 2.42 | 3.21 | 1.33 |

Of note, the impact of reduced CYP2C19 expression in gut was also investigated in DDI settings with fluvoxamine (Figure S3.7). DDI predictions with the reduced CYP2C19 expression were similar in terms of C_max_ (in presence of clinical data), and slightly higher (~20%) in terms of AUC. DDI simulations described above were performed with default CYP2C19 expression levels.


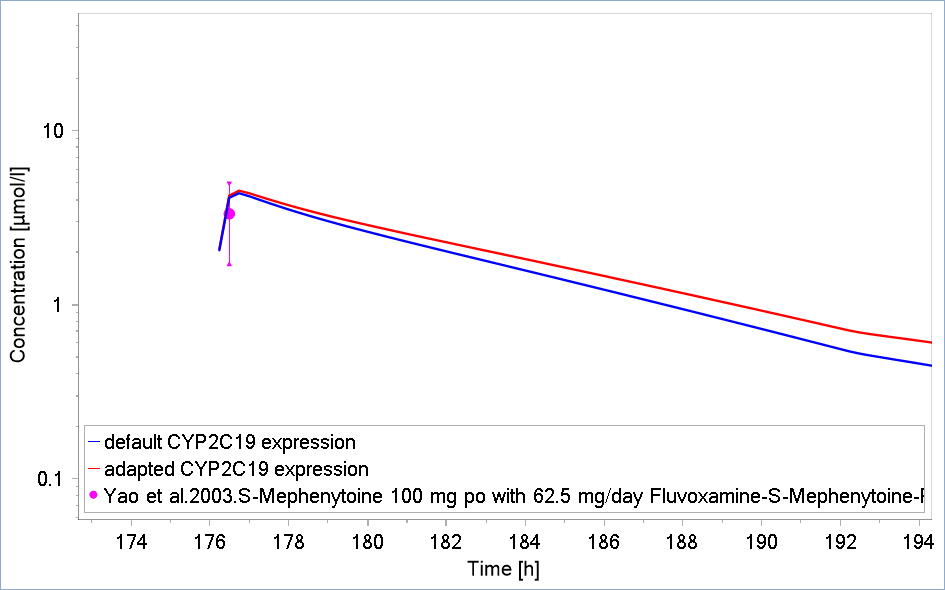


Figure S3.7 Simulated S-mephenytoin plasma profiles when administered together with fluvoxamine in individuals with default or adapted CYP2C19 expression in gut.

- 1. Moclobemide Model
     1. Final Model Parameters and Structure

The final model consisted of the following key aspects:

- Tissue partition coefficients calculated according to Rodgers & Rowland
- Limited renal elimination (0.03 ml/min/kg)
- Saturable CYP2C19 metabolism
- Linear non-CYP2C19 metabolism
- Time-dependent auto-inhibition of CYP2C19.

The table below summarizes the final model parameters:

Table S3.15 Final model parameters for Moclobemide

| **Parameter** | **Value** | **Source** | **Assumptions / Comment** |
| --- | --- | --- | --- |
| MW | 268.7 g/mol | DrugBank |  |
| Partition coefficient model | Rodgers and Rowland | (estimated) | Various distribution models available in PK-Sim were explored and compared |
| Specific intestinal permeability (transcellular) | 1.69E-5 cm/min | Calculated by PK-Sim |  |
| Solubility in intestinal fluid @pH 6.2 | 3 mg/mL | Roche data[^20^](#_ENREF_20) |  |
| Dissolution (particle size) | 20 um (thickness)  10 um (radius) | Fixed | Fast dissolution; simulations not sensitive to parameter change |
| LogD | 1.79 | Pons et al.[^21^](#_ENREF_21) |  |
| fu | 0.50 | Mayersohn et al.[^22^](#_ENREF_22) |  |
| pKa | 6.2 | Raaflaub et al.[^23^](#_ENREF_23) | Weak base |
| Km_2C19 | 1.11 µmol/l | **Estimated** | Saturable metabolism; supported by literature (dose non-proportionality) |
| Vmax_2C19 | 2.03 umol/min/kg | **Estimated** | See above. |
| Intrinisc Clearance FMO  (i.e. non CYP2C19 metabolism) | 0.24 l/min | **Estimated** | Estimated using data of 2C19 poor metabolizers |
| CYP 2C19 Expression | - | PK-Sim database |  |
| FMO expression | Ref. conc. 1 umol/l  half-life (liver) 36 h | Manual | Relative expression in liver set to 1.  Note this does not cover only FMO but also other non-2C19 metabolism |
| Renal Elimination | 0.03 ml/min/kg | Derived from Schoerlin et al.[^24^](#_ENREF_24) | Schoerlin reports 2.6ml/min/76kg |
| Ki_CYP2C19 (free) | 203.83 umol/l | Kramer-Nielsen et al.[^25^](#_ENREF_25) | The total ki value reported by Kramer was 210 umol/L and corrected with an fu_mic of 0.973 |
| CYP2C19 Time-dependent autoinhibition | Kinact: 5.00 1/h  Kinact__half_: 94.85 umol/l | Wu 2014[^8^](#_ENREF_8) (omeprazole)  **Estimated** | Both parameters not separately identifiable – fixed value for Kinact reported by Wu |

- - 1. DDI Predictions for Moclobemide with omeprazole as inhibitor

A dynamic DDI simulation with moclobemide as substrate and omeprazole as perpetrator was simulated. The final omeprazole model described in Section S4.1.1 was used and a single dose of 40 mg omeprazole (racemate, i.e. 20 mg S-omeprazole and 20 mg R-omeprazole) was simulated. To accommodate this simulation, a typical Asian Male subject for DDI (age=30 y, weight=60.03 kg, height=170 cm, BMI=20.78 kg/m^2^) was created from the predefined database “Asian (Tanaka, 1996)” by adding CYP2C19, CYP3A4 and FMO (other) expressions from the PK-Sim RT-PCR database.

Figure S3.8 illustrates how well the predicted time-course matches with the observations from Yu et al[^26^](#_ENREF_26) and Table S3.16 shows the key PK parameters derived from the simulations. The predicted and observed AUC and C_max_ ratios are very close.


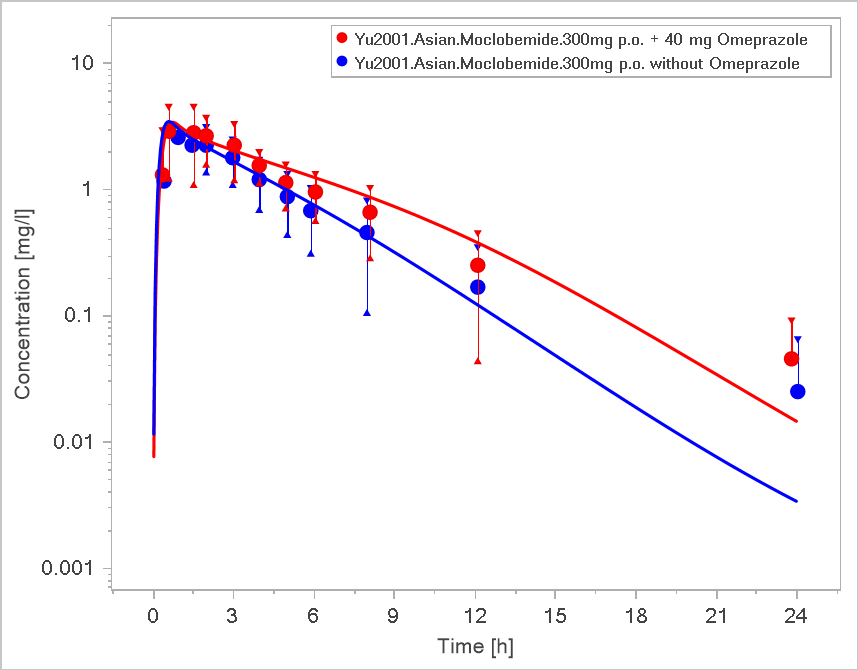


Figure S3.8 Simulated and observed concentration-time course of moclobemide with and without concomitant omeprazole

Table S3.16 Observed and predicted PK parameters and ratios for moclobemide with/without omeprazole

|  |  | **AUCinf (μg.h/mL)** | **AUCR** | **C_max_ (μg/mL)** | **C_max_R** |
| --- | --- | --- | --- | --- | --- |
| 300 mg moclobemide | observed | 12.98 |  | 3.47 |  |
|  | predicted | 12.87 |  | 3.44 |  |
| 300 mg moclobemide + 40 mg omeprazole | observed | 16.95 | **1.31** | 3.84 | **1.11** |
|  | predicted | 18.04 | **1.40** | 3.40 | **0.99** |
| pred/obs ratio |  |  | **1.07** |  | **0.89** |

To note, adapted expression of CYP2C19 in gut had minimal impact on the DDI prediction as shown in Figure S3.23.


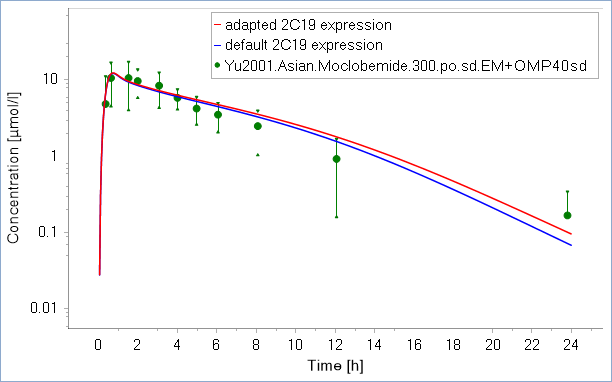


Figure S3.9 Simulated and observed concentration-time course of moclobemide in presence of omeprazole with default and adapted CYP2C19 expression in gut.

- 1. Tizanidine Model
     1. Final Model Parameters and Structure

The final tizanidine model consisted of the following key aspects:

- Tissue partition coefficients calculated according to Berezhkovskiy
- Renal elimination (GFR fraction=1)
- Linear CYP1A2 metabolism
- Dissolution parameters for fasted and fed state

The final parameters of the tizanidine PBPK model template are shown in Table S3.17.

Table S3.17 Final parameters of the tizanidine model

| **Parameter** | **Value** | **Source** | **Assumptions/ Comment** |
| --- | --- | --- | --- |
| MW free base  Tizanidine-HCl | 253.7 g/mol  289.7 g/mol | Website drugbank.ca/drugs/DB00697 | Dose of commercial product usually refers to amount of tizanidine free base – no conversion is necessary |
| Partition coefficient model | Berezhkovskiy | (estimated) | Various distribution models available in PK-Sim were explored and compared |
| Aqueous Solubility at pH 7.4 | 0.133 mg/mL | Website drugbank.ca/drugs/DB00697 | Calculated by ALOGPS |
| LogP | 1.4 | Website drugbank.ca/drugs/DB00697 | Experimental; value calculated by ALOGPS is similar: 1.6 |
| fu | 0.7 | Prescribing information tizanidnine^[27](#_ENREF_27)^ |  |
| pKa (Base) | 7.49 | Website drugbank.ca/drugs/DB00697 | Calculated by ChemAxon |
| Intrinsic Clearance 1A2 | 7.29 L/min | **Estimated** | Metabolism via CYP1A2 non-saturable in the relevant concentration range |
| Weibull Dissolution time/ shape (fasted) | 38.5 min / 0.963 | **Estimated** |  |
| 1A2 Expression | - | PK-Sim database |  |
| Renal Elimination | 1.0 GFR fraction | fixed | To account for a limited elimination via the kidney (<5%) |
| Ki_CYP1A2 | 3.29 nmol/L | Iga 2016[^2^](#_ENREF_2) | Unbound value from in vivo experiments; presumably the most robust source of Ki as it eliminates the need for in vitro- in vivo scaling |
| Ki_CYP1A2 (alternative) | 0.87 nmol/L | **Estimated** | Alternative value estimated based on DDI study of Granfors 2004[^28^](#_ENREF_28) |

- - 1. DDI Predictions for Tizanidine Model Qualification

The final model parameters were used to predict the drug-drug interaction with fluvoxamine as a perpetrator. For this the Ki value reported by Iga et al.[^2^](#_ENREF_2) for CYP1A2 and tizanidine derived from in vivo studies (3.29 nmol/L) was used initially.

In the clinical study reported by Granfors et al.[^29^](#_ENREF_29), a single oral dose of 100 mg tizanidine was given after treatment with fluvoxamine (100 mg fluvoxamine maleate ~73.3 mg free base, q.d. for 4 days).


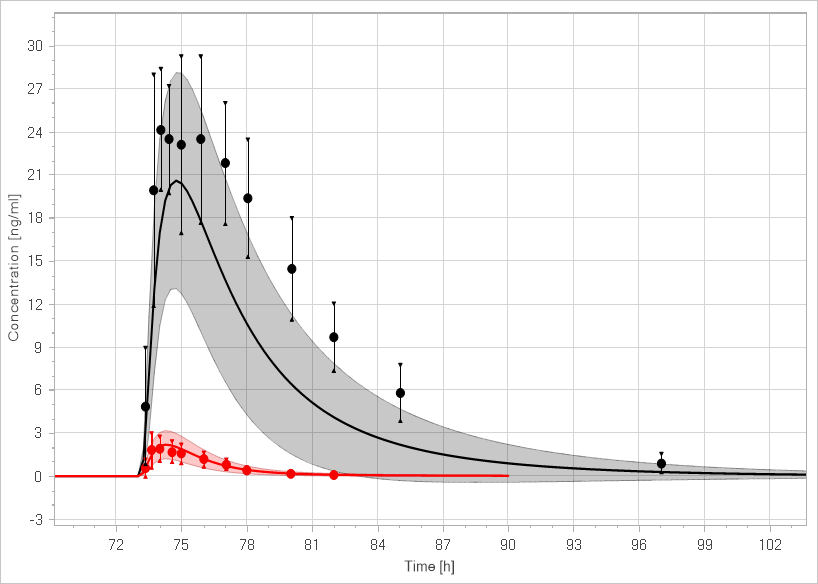


Figure S3.10 Population prediction and mean observations of 4 mg tizanidine given with (black) or without (red) fluvoxamine – Ki value from Iga et al.

A population of 2000 virtual male subjects between 21 and 31 years and weighting 65 to 83 kg was simulated. The area encompasses the standard deviation interval of the predicted profiles while the solid line is the arithmetic mean. Symbols are observed mean values +/- SD digitized from Granfors et al.[^29^](#_ENREF_29)

Table S3.18 Observed and predicted PK parameters and ratios for tizanidine with/without fluvoxamine.

| **Source** | **AUClast  [ng.h/mL]** | **AUCR** | **C_max_  [ng/mL]** | **C_max_R** |
| --- | --- | --- | --- | --- |
| Observed control | 6.6* |  | 1.95* |  |
| Observed +4x100mg fluvoxamine | 223* | 33.8 | 24.1* | 12.4 |
| Predicted control | 7.5 |  | 2.2 |  |
| Predicted  +4x100mg fluvoxamine (Ki from Iga et al.) | 122.9 | 16.4 | 20.6 | 9.4 |
| Pred/obs ratio |  | 0.48 |  | 0.76 |
| Predicted  +4x100mg fluvoxamine (Ki optimized) | 290 | 38.7 | 30.9 | 14 |
| Pred/obs ratio |  | 1.15 |  | 1.14 |

*value calculated with PK-Sim inbuild PK-analysis from scanned measured data.

While the predicted C_max_ ratio reasonably matches closely the observed value, the AUC ratio is underpredicted by approximately twofold (Figure S3.10). As the predictions of the tizanidine profiles without co-administration of fluvoxamine matched the observations very well, it was concluded that the most plausible reason for the under prediction was the value of Ki, as the fraction metabolized via CYP1A2 was already 99% (Figure S3.11). Hence Ki was optimized using the data from Granfors et al. and the simulations were repeated. A Ki value of 0.87 +/- 0.19 nmol/L was estimated, which is still in line with the Ki values derived for other CYP1A2 substrates (see Supplement S1, Table S1.1.2).

As expected, the predicted AUC and C_max_ ratios are now much closer to the observed values (last row in Table S3.18).


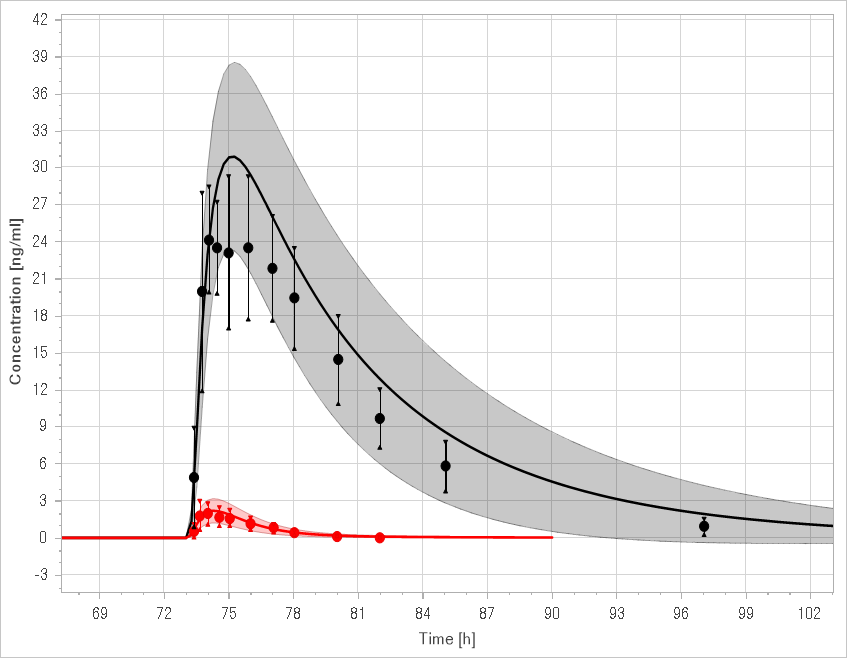


Figure S3.11 Population prediction and mean observations of 4 mg tizanidine given with (black) or without (red) fluvoxamine – optimized Ki value.

A population of 2000 virtual male subjects between 21 and 31 years and weighting 65 to 83 kg was simulated. The area encompasses the standard deviation interval of the predicted profiles while the solid line is the arithmetic mean. Symbols are observed mean values +/- SD digitized from Granfors et al.[^29^](#_ENREF_29)

- 1. Mexiletine Model
     1. Final Model Parameters and Structure

The final mexiletine model consisted of the following key aspects:

- Tissue partition coefficients calculated according to Rodgers & Rowland
- Renal elimination (24.7 mL/h/kg)
- Linear CYP1A2 metabolism
- Linear CYP2D6 metabolism
- Linear unspecific metabolism in liver

The final parameters are shown in Table S3.19.

Table S3.19 Final parameters of the mexiletine model

| **Parameter** | **Value** | **Source** | **Assumption/comment** |
| --- | --- | --- | --- |
| MW | 179.3 g/mol | DrugBank |  |
| Lipophilicity | 2.38 | Estimated | Similar values in other sources (e.g DrugBank: 2.15-2.45) |
| Partition coefficient model | Rodgers and Rowland | Estimated | Various distribution models available in PK-Sim were explored and compared |
| Specific intestinal permeability (transcellular) [cm/min] | 4.74E-4 | Estimated | Estimated on single p.o. data |
| Aqueous Solubility at pH 7.4 | 0.54 mg/mL | DrugBank | Calculated by ALOGPS |
| Dissolution time/ shape (Weibull) | 60.00 min / 0.92 | Default |  |
| fu | 0.50 | DrugBank | Similar value in the label:0.40–0.50 |
| Compound type/pKa | Base/9.2 | DrugBank |  |
| CYP21A2 specific clearance [1/min] | 0.08 | Ratio to total hepatic CL derived from Labbé 2000[^30^](#_ENREF_30) | 28.6% of fitted specific hepatic CL (0.50 1/min) divided by 1.8 µmol/l reference concentration of CYP1A2 |
| CYP2D6 specific clearance [1/min] | 0.46 | Ratio to total hepatic CL derived from Labbé 2000[^30^](#_ENREF_30) | 37.1% of fitted specific hepatic CL (0.50 1/min) divided by 0.4 µmol/l reference concentration of CYP2D6 |
| Unspecific specific clearance [1/min] | 0.17 | Ratio to total hepatic CL derived from Labbe 200[^30^](#_ENREF_30) | 34.3% of fitted specific hepatic CL (0.50 1/min) |
| Renal Elimination [1/min] | 0.14 | Labbé 2000[^30^](#_ENREF_30) - Table II R-mexiletine in EM |  |
| Specific organ permeability [cm/min] | 1.64E-3 | Estimated | Estimated based on i.v. data |
| Ki [nM] fluvoxamine | 2.97 | Iga 2016[^2^](#_ENREF_2) - in vivo, unbound Ki for caffeine | Fluvoxamine as preparator toward victim Mexiletine |
| Ki [uM] mexiletine | 0.28 | Wei 1999[^31^](#_ENREF_31) - calculated in vivo, unbound Ki for Methoxyresofurin |  |

- - 1. DDI Predictions for Mexiletine Model Qualification
       1. **DDI with caffeine as victim drug**

A dynamic DDI simulation was conducted with mexiletine as CYP1A2 inhibitor and caffeine as victim, and compared to literature data.

Clinical observations of mexiletine-caffeine interaction were derived from Joeres et al.[^32^](#_ENREF_32) where 5 healthy volunteers received 366 mg caffeine (400 mg caffeine monohydrate) after an overnight fast, together with 200 mg mexiletine orally. One week later caffeine was administered alone. The in-built caffeine template model included in PK-Sim[^3^](#_ENREF_3) was used for caffeine predictions (see Supplementary file 2b). This model was previously qualified using clinical trial data. Competitive inhibition was assumed on CYP1A2 enzyme between caffeine (substrate) and mexiletine (inhibitor) with an inhibitory constant of 0.28 µM. No reported values were found for mexiletine Ki on CYP1A2 and caffeine as substrate, so the inhibitory constant for methoxyresofurin (151) was used [calculated in vivo, unbound Ki for methoxyresofurin].

The observed caffeine concentrations with/without mexiletine are shown in Figure S3.12 for a representative individual together with the simulated median-90% prediction interval (PI) based on a population of 1000 individuals having similar characteristics to the healthy volunteers of the study. The model predictions are in good agreement with the observations. Table S3.20 shows the simulated and observed caffeine AUC and C_max_ ratios with and without co-administration of mexiletine. Mexiletine is a moderate inhibitor of CYP1A2 and increases the AUC of caffeine by ~2-fold. The built-in model for caffeine seems to overpredict the caffeine levels and subsequent interaction. However, predicted ratios are within 2-fold of observed ratios.

| 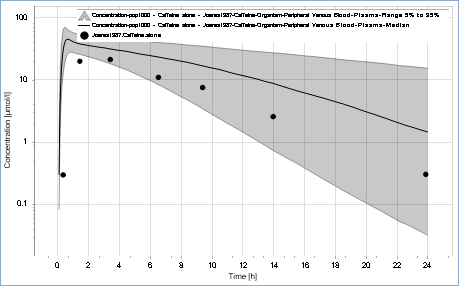 | 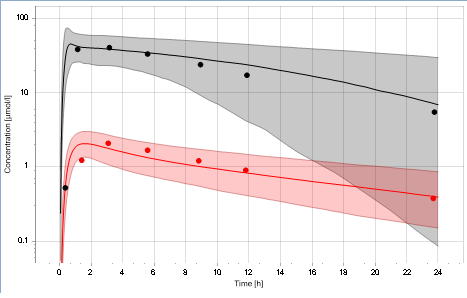 |
| --- | --- |

Figure S3.12 Predicted (median-90%PI) and observed (data from a representative individual) caffeine (black) and mexiletine (red) concentrations. Left-hand side: caffeine alone; right-hand side: caffeine together with mexiletine.

Table S3.20 Observed and predicted caffeine AUC and C_max_ ratios with/without mexiletine

|  |  | **AUC**  **(μg.min/mL)** | **AUCR** | **C_max_ (μg/mL)** | **C_max_R** |
| --- | --- | --- | --- | --- | --- |
| 400 mg caffeine | Observed | 1941 |  | 4.12 |  |
|  | Predicted | 3520 |  | 8.66 |  |
| 400 mg caffeine + 200 mg mexiletine | Observed | 5500 | 2.83 | 7.79 | 1.89 |
|  | Predicted | 5631 | 1.60 | 8.66 | 1.00 |
| Ratio pred/obs |  |  | 0.56 |  | 0.53 |

- - - 1. **DDI with Tizanidine as victim drug**

A dynamic DDI simulation was conducted with mexiletine as CYP1A2 inhibitor and tizanidine as victim, and compared to literature data from Momo et al. 2010[^33^](#_ENREF_33). The same Ki as for the caffeine interaction simulation was used (0.28 µM). The predefined “Standard European Male for DDI” individual (age=30 y, weight=73 kg, height=176 cm, BMI=23.57 kg/m^2^) was used. The pharmacokinetics of tizanidine was examined in an open-label study in 12 healthy participants after a single dose of tizanidine (2 mg) with and without mexiletine coadministration (50 mg, 3 times as a pretreatment for a day and 2 times on the study day)[^33^](#_ENREF_33).

Figure S3.13 shows observed and simulated tizanidine concentrations with and without mexiletine coadministration. Tizanidine is underpredicted both with and without mexiletine, probably due to the fact that a food effect was not taken into account as already observed during tizanidine model development (See Supplement S2b, Section 2.5.4.2). However, the simulated ratios of tizanidine AUC and C_max_ are in reasonable agreement (within 2-fold) with the observations from the clinical study. At the same time, mexiletine PK predictions were in agreement with observations (Table S3.21).

| 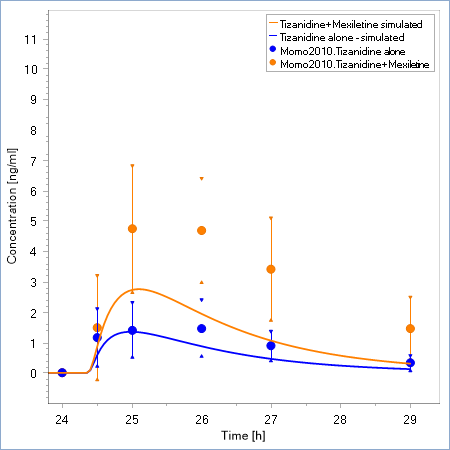 | 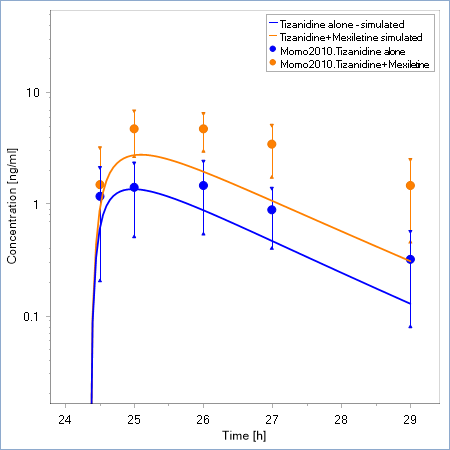 |
| --- | --- |

Figure S3.13 Predicted and observed (mean +/- SD) tizanidine concentrations with (orange) and without (blue) mexiletine. Left-hand side: linear y-scale; right-hand side: log y-scale.

Table S3.21 Observed and predicted tizanidine AUC and C_max_ ratios with/without mexiletine

|  |  | **AUC**  **(ng.h/mL)** | **AUCR** | **C_max_ (ng/mL)** | **C_max_R** |
| --- | --- | --- | --- | --- | --- |
| 2 mg tizanidine | Observed | 4.5 |  | 1.47 |  |
|  | Predicted | 2.9 |  | 1.36 |  |
| 2 mg tizanidine + 50 mg mexiletine tid | Observed | 15.4 | 3.42 | 4.74 | 3.22 |
|  | Predicted | 6.3 | 2.17 | 2.76 | 2.03 |
| Ratio pred/obs |  |  | 0.63 |  | 0.63 |

- - - 1. **DDI with fluvoxamine as CYP1A2 inhibitor**

A dynamic DDI simulation was conducted with fluvoxamine as CYP1A2 inhibitor and mexiletine as victim, and compared to literature data[^34^](#_ENREF_34)^,^[^35^](#_ENREF_35). A typical Japanese subject (age=30 y, weight=61.87 kg, height=170 cm, BMI=21.67 kg/m^2^) was created in PK-Sim from predefined database “Japanese (2015)” by adding CYP2D6 and CYP1A2 expressions from PK-Sim RT-PCR database, and adding an unspecific metabolizing enzyme in intracellular liver (ref. conc=1µM, t_half=36h).

A randomized crossover design with two phases was used. Each subject received an oral dose of mexiletine (200 mg) or fluvoxamine (50 mg twice a day) for 7 days, and on the eighth day they received mexiletine and fluvoxamine concomitantly.

Observed and simulated mexiletine concentrations with and without fluvoxamine treatment are shown in Figure S3.14 and the corresponding ratios are presented in Table S3.22. It can be concluded that mexiletine levels were very well predicted with and without fluvoxamine.

Figure S3.14 Predicted and observed (mean +/- SD) mexiletine concentrations with (red) and without (blue) fluvoxamine.

Table S3.22 Observed and predicted mexiletine AUC and C_max_ ratios with/without fluvoxamine

|  |  | AUCinf  (μg.h/mL) | AUCR | C_max_ (μg/mL) | **C_max_R** |
| --- | --- | --- | --- | --- | --- |
| 200 mg mexiletine | Observed | 6.70 |  | 0.536 |  |
|  | Predicted | 6.05 |  | 0.427 |  |
| 200 mg mexiletine + 50 mg fluvoxamine b.i.d. | Observed | 10.38 | 1.55 | 0.623 | 1.16 |
|  | Predicted | 7.87 | 1.30 | 0.463 | 1.08 |
| Ratio pred/obs |  |  | 0.84 |  | 0.93 |

- 1. Ethinylestradiol Model
     1. Final Model Parameters and Structure

The final ethinylestradiol model consisted of the following key aspects:

- Tissue partition coefficients calculated according to Berezhkovskiy
- Renal elimination
- Linear CYP1A2 metabolism
- Linear CYP2C8 metabolism
- Linear CYP2C9 metabolism
- Linear CYP3A4 metabolism
- Saturable UGT1A1 metabolism
- Linear unspecific metabolism in liver
- Time dependent inhibition of CYP1A2

Final model parameters are listed in Table S3.23.

Table S3.23 Final parameters of the ethinylestradiol model

| **Parameter** | **Value** | **Source** | **Assumption/comment** |
| --- | --- | --- | --- |
| MW | 296.4 g/mol | DrugBank |  |
| Lipophilicity | 3.48 | Estimated | Similar values in other sources (e.g DrugBank: 3.63 – 3.9) |
| Partition coefficient model | Berezhkovskiy | (Estimated) | Various distribution models available in PK-Sim were explored and compared |
| Specific intestinal permeability (transcellular) [cm/min] | 1.68E-4 | Estimated |  |
| Aqueous Solubility at pH 7.4 | 0.00677 mg/mL | DrugBank |  |
| Dissolution time/ shape /lagtime (Weibull) | 36.51 min / 0.92 / 6.77 | Estimated |  |
| fu | 0.03 | DrugBank |  |
| Compound type/pKa | Acid/10.33 | DrugBank |  |
| CYP3A4 Clint [µL/min/pmol] | 0.5 | Ezuruike^[36](#_ENREF_36)^ |  |
| CYP2C9 Clint [µL/min/pmol] | 0.51 | Ezuruike^[36](#_ENREF_36)^ |  |
| CYP1A2 Clint [µL/min/pmol] | 0.51 | Ezuruike^[36](#_ENREF_36)^ |  |
| CYP2C8 Clint [µL/min/pmol] | 0.13 | Ezuruike^[36](#_ENREF_36)^ |  |
| UGT1A1 Vmax [pmol/min/mg prot] | 408.5 | Ezuruike^[36](#_ENREF_36)^ |  |
| UGT1A1 Km [µM] | 19.22 | Ezuruike^[36](#_ENREF_36)^ |  |
| Additional Clint HLM [µL/min/mg prot] | 118.83 | Ezuruike^[36](#_ENREF_36)^ |  |
| Renal clearance ->  Specific renal Clearance | 2.08 L/h  2.78 1/min | Ezuruike^[36](#_ENREF_36)^ |  |
| Ki CYP1A2 TDI | 0.48 µmol/l | Estimated |  |
| Kinact TDI | 100 1/min or 200 1/min | Estimated |  |

- - 1. DDI Predictions for Ethinylestradiol Model Qualification
       1. **DDI with caffeine as victim drug**

A dynamic DDI simulation was conducted with ethinylestradiol as CYP1A2 inhibitor and caffeine as victim, and compared to literature data. No profiles of caffeine under this interaction were available, but Balogh et al 1995[^37^](#_ENREF_37) reported a 55% decrease in caffeine CL due to ethinylestradiol coadministration. The interaction was simulated according to their design. Caffeine clearances in presence of ethinylestradiol are compared in Table S3.24. The time-dependent inhibition (TDI) model with Kinact=100min^-1^ provided the best prediction (Table S3.25).

Table S3.24 Observed and predicted caffeine clearance in presence of ethinylestradiol

| **Scenario** | **Caffeine clearance [ml/min] with ethinylestradiol** | **Pred/obs** |
| --- | --- | --- |
| Observed | 38.8 |  |
| Predicted, Kinact=200 min^-1^ | 18.85 | 0.49 |
| Predicted, Kinact=100 min^-1^ | 28.6 | 0.74 |

Table S3.25 Observed and predicted caffeine AUC and C_max_ ratios with/without ethinylestradiol using Kinact =100 min^-1^

|  |  | **AUCinf**  **(μg.h/mL)** | **AUCR** | **C_max_ (μg/mL)** | **C_max_R** |
| --- | --- | --- | --- | --- | --- |
| 200 mg caffeine | Observed | 40.31 |  | 5.23 |  |
|  | Predicted | 33.63 |  | 5.36 |  |
| 200 mg caffeine + 0.03 mg ethinylestradiol q.d. | Observed | 85.91 | 2.13 | 6.01 | 1.15 |
|  | Predicted | 125.7 | 3.74 | 5.80 | 1.08 |
| Ratio pred/obs |  |  | 1.75 |  | 0.94 |

- - - 1. **DDI with tizanidine as victim drug**

The model including the contribution of different CYPs and UGTs was coupled with the tizanidine model template to run a dynamic DDI simulation with ethinylestradiol as CYP1A2 moderate inhibitor and tizanidine as victim drug.

Clinical observations of ethinylestradiol-tizanidine interaction were derived from Granfors et al.[^38^](#_ENREF_38) in which 15 healthy women using oral contraceptives (OCs) (ethinylestradiol + gestodene) and 15 healthy women without OCs (control subjects) ingested a single dose of 4 mg tizanidine.

The observed tizanidine concentrations are shown in Figure S3.15 when administered together with ethinylestradiol according to the final model.

Initially, competitive inhibition by ethinylestradiol was assumed on CYP1A2 enzyme, with an inhibitory constant Ki of 10.6 µmol/l (ethinylestradiol-phenacetin interaction[^39^](#_ENREF_39)). As can be observed in Figure S3.15, the model with competitive inhibition (green curve) underpredicts the magnitude of the interaction, even when decreasing Ki to the pM range (black curve). Therefore, a TDI function was incorporated in the CYP1A2 enzyme system. Note, a TDI mechanism for ethinylestradiol was not evident in literature[^27^](#_ENREF_27)^,^[^39^](#_ENREF_39). The substantial and prolonged inhibition may result from CYP1A2 inhibition by ethinylestradiol-metabolites having a different half-life from the parent. Chang et al. 2009[^40^](#_ENREF_40) for example found that the 2-hydroxy-ethinylestradiol and 2-methoxy- ethinylestradiol IC50s toward rCYP1A1 and rCYP1A2 are comparable to that of the parent. However, not having the possibility to model the contribution of ethinylestradiol-metabolites, a TDI function on CYP1A2 was used instead to account for this effect. The estimated parameters for TDI are reported in Table S3.26.

Table S3.26 Parameter estimates for time-dependent inhibition on CYP1A2 by ehtinylestradiol

| **Identification Parameter** | **95% Confidence Interval** |
| --- | --- |
| Kinact [1/min] | 200.0 +- 2.46E-4 |
| K_kinact_half [µmol/l] | 0.48 +- 0.10 |

As can be observed, the estimated inhibition process (magenta curve) is well describing the tizanidine concentrations, albeit a slight overprediction of the observations around C_max_. An alternative inhibition model was also attempted by reducing Kinact to 100 1/min to better capture the first time points (blue curve).Table S3.27 and Table S3.28 show the ratios of tizanidine AUC and C_max_ with and without co-administration of ethinylestradiol. When using ethinylestradiol as a perpetrator with a new victim drug, the recommendation is to test both of these values and discuss the potential impact, as the value resulting in the best prediction might depend on the substrate.


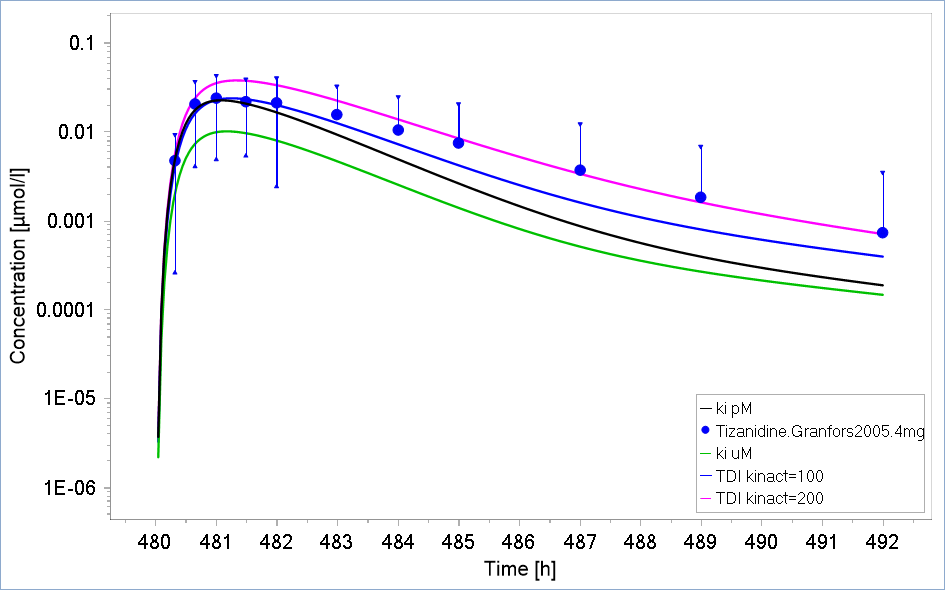


Figure S3.15 Predicted and observed (mean+/-SD) tizanidine concentrations when co-administered with ethinylestradiol.
Ki uM – Competitive inhibition model with a Ki of 10.6 µmol/l.
Ki pM – Competitive inhibition model with Ki in the pM range.

Table S3.27 Observed and predicted tizanidine AUC ratio with and without ethinylestradiol

| **AUC (ng.h/ml)** | **without ethinylestradiol** | **with ethinylestradiol** | **AUCR** | **pred/obs ratio** |
| --- | --- | --- | --- | --- |
| Observed | 6.27 | 24.57 | 3.92 |  |
| Competitive Ki=10.6µM | 7.49 | 7.49 | 1.00 | 0.26 |
| Kinact=200 min^-1^ | 7.49 | 28.04 | 3.74 | 0.96 |
| Kinact=100 min^-1^ | 7.49 | 17.15 | 2.29 | 0.58 |

Table S3.28 Observed and predicted tizanidine C_max_ ratio with and without ethinylestradiol

| **C_max_ (ng/ml)** | **without ethinylestradiol** | **with ethinylestradiol** | **C_max_R** | **pred/obs ratio** |
| --- | --- | --- | --- | --- |
| Observed | 2.12 | 6.4 | 3.02 |  |
| Competitive Ki=10.6µM | 2.54 | 2.55 | 1.00 | 0.33 |
| Kinact=200 min^-1^ | 2.54 | 8.34 | 3.28 | 1.09 |
| Kinact=100 min^-1^ | 2.54 | 5.45 | 2.15 | 0.71 |

- 1. Glossary of abbreviations used in this document

| ALOGPS | Atomic LogP |
| --- | --- |
| AUC | Area under the plasma concentration versus time curve |
| AUCinf | AUC until infinity |
| AUCR | Area under the plasma concentration versus time curve Ratio |
| b.i.d. | Twice daily (bis in diem) |
| BMI | Body mass index |
| CL | Clearance |
| CL/F | Oral clearance |
| Clint | Intrinsic liver clearance |
| C_max_ | Maximum concentration |
| C_max_R | Maximum concentration Ratio |
| CYP | Cytochrome P450 oxidase |
| CYP1A2 | Cytochrome P450 1A2 oxidase |
| CYP2C8 | Cytochrome P450 2C8 oxidase |
| CYP2C9 | Cytochrome P450 2C9 oxidase |
| CYP2C19 | Cytochrome P450 2C19 oxidase |
| CYP2D6 | Cytochrome P450 2D6 oxidase |
| CYP3A4 | Cytochrome P450 3A4 oxidase |
| DDI | Drug-drug interaction |
| EM | Extensive metabolizers |
| FMO | Flavin-containing monooxygenase |
| fu | Fraction unbound in plasma |
| fu-mic | Fraction unbound in the microsomal system |
| GFR | Glomerular filtration rate |
| GI | Gastrointestinal |
| HLM | Human liver microsomes |
| hm | Homozygous |
| Ht | Heterozygous |
| i.v. | Intravenous |
| IVIVE | In Vitro to In Vivo Extrapolation |
| Kcat | Catalyst rate constant |
| Ki | Inhibitor constant |
| Kinact | Rate of enzyme inactivation |
| Km | Michaelis Menten constant |
| logD | Water:octanol partition coefficient for the ionized and un-ionized form of the compound at a specific pH |
| LogP | Water:octanol partition coefficient for the un-ionized form of the compound |
| m.d. | Multiple doses |
| MW | Molecular weight |
| PBPK | Physiologically-based pharmacokinetics |
| PK | Pharmacokinetics |
| pKa | Negative logarithm of acid dissociation constant |
| PM | Poor metabolizers |
| RT-PCR | Reverse transcription polymerase chain reaction |
| p.o. | Per os |
| Pred/obs | Predicted/observed |
| q.d. | Once daily (quaque diem) |
| s.d. | Single dose |
| SD | Standard deviation |
| TDI | Time dependent inhibition |
| t.i.d. | Three times a day (ter in die) |
| UGT | Uridine 5'-diphospho-glucuronosyltransferase |
| V_max_ | V_max_: maximum enzymatic rate achieved by the system at saturating substrate concentration |

REFERENCES

1. Alqahtani S., Kaddoumi A. Development of a Physiologically Based Pharmacokinetic/Pharmacodynamic Model to Predict the Impact of Genetic Polymorphisms on the Pharmacokinetics and Pharmacodynamics Represented by Receptor/Transporter Occupancy of Central Nervous System Drugs. *Clin Pharmacokinet* **55** 957-969. (2016)

2. Iga K. Dynamic and Static Simulations of Fluvoxamine-Perpetrated Drug-Drug Interactions Using Multiple Cytochrome P450 Inhibition Modeling, and Determination of Perpetrator-Specific CYP Isoform Inhibition Constants and Fractional CYP Isoform Contributions to Victim Clearance. *J Pharm Sci* **105** 1307-1317. (2016)

3. GitHub. Caffeine.pksim5. 2017 [cited]Available from: <https://github.com/Open-Systems-Pharmacology/Example_Caffeine>

4. Jeppesen U., Loft S., Poulsen H.E., Brsen K. A fluvoxamine-caffeine interaction study. *Pharmacogenetics* **6** 213-222. (1996)

5. Culm-Merdek K.E., von Moltke L.L., Harmatz J.S., Greenblatt D.J. Fluvoxamine impairs single-dose caffeine clearance without altering caffeine pharmacodynamics. *Br J Clin Pharmacol* **60** 486-493. (2005)

6. U.S. Food and Drug Administration. Spilling the beans: how much caffeine is too much? 2018 [cited 2019 19 December 2019]Available from: <https://www.fda.gov/consumers/consumer-updates/spilling-beans-how-much-caffeine-too-much>

7. Olivares-Morales A., Ghosh A., Aarons L., Rostami-Hodjegan A. Development of a Novel Simplified PBPK Absorption Model to Explain the Higher Relative Bioavailability of the OROS(R) Formulation of Oxybutynin. *AAPS J* **18** 1532-1549. (2016)

8. Wu F.*, et al.* Predicting nonlinear pharmacokinetics of omeprazole enantiomers and racemic drug using physiologically based pharmacokinetic modeling and simulation: application to predict drug/genetic interactions. *Pharm Res* **31** 1919–1929. (2014)

9. Liu K.H.*, et al.* Stereoselective inhibition of cytochrome P450 forms by lansoprazole and omeprazole in vitro. *Xenobiotica* **35** 27-38. (2005)

10. Yasui-Furukori N.*, et al.* Different inhibitory effect of fluvoxamine on omeprazole metabolism between CYP2C19 genotypes. *Br J Clin Pharmacol* **57** 487-494. (2004)

11. Pentikainen P.J., Halinen M.O., Helin M.J. Pharmacokinetics of intravenous mexiletine in patients with acute myocardial infarction. *J Cardiovasc Pharmacol* **6** 1-6. (1984)

12. Foti R.S., Rock D.A., Wienkers L.C., Wahlstrom J.L. Selection of alternative CYP3A4 probe substrates for clinical drug interaction studies using in vitro data and in vivo simulation. *Drug Metab Dispos* **38** 981-987. (2010)

13. Open Systems Pharmacology Suite Community. Open Systems Pharmacology Suite Manual, Version 7.0. 2017.

14. Foti R.S., Wahlstrom J.L. CYP2C19 inhibition: the impact of substrate probe selection on in vitro inhibition profiles. *Drug Metab Dispos* **36** 523-528. (2008)

15. Cho J.Y.*, et al.* Omeprazole hydroxylation is inhibited by a single dose of moclobemide in homozygotic EM genotype for CYP2C19. *Br J Clin Pharmacol* **53** 393-397. (2002)

16. Sangster J. A databank of evaluated octanol-water partition coefficients (Log P) on microcomputer diskette: Sangster Res Lab.; 1994.

17. Adedoyin A., Arns P.A., Richards W.O., Wilkinson G.R., Branch R.A. Selective effect of liver disease on the activities of specific metabolizing enzymes: investigation of cytochromes P450 2C19 and 2D6. *Clin Pharmacol Ther* **64** 8-17. (1998)

18. Steere B., Baker J.A., Hall S.D., Guo Y. Prediction of in vivo clearance and associated variability of CYP2C19 substrates by genotypes in populations utilizing a pharmacogenetics-based mechanistic model. *Drug Metab Dispos* **43** 870-883. (2015)

19. Yao C., Kunze K.L., Trager W.F., Kharasch E.D., Levy R.H. Comparison of in vitro and in vivo inhibition potencies of fluvoxamine toward CYP2C19. *Drug Metab Dispos* **31** 565-571. (2003)

20. INCHEM. Moclobemide. [cited 26 November 2019]Available from: inchem.org/documents/pims/pharm/pim151.htm#PartTitle:3.%20%20PHYSICO-CHEMICAL%20PROPERTIES

21. Pons G.*, et al.* Moclobemide excretion in human breast milk. *Br J Clin Pharmacol* **29** 27-31. (1990)

22. Mayersohn M., Guentert T.W. Clinical pharmacokinetics of the monoamine oxidase-A inhibitor moclobemide. *Clin Pharmacokinet* **29** 292-332. (1995)

23. Raaflaub J., Haefelfinger P., Trautmann K.H. Single-dose pharmacokinetics of the MAO-inhibitor moclobemide in man. *Arzneimittelforschung* **34** 80-82. (1984)

24. Schoerlin M.P., Mayersohn M., Korn A., Eggers H. Disposition kinetics of moclobemide, a monoamine oxidase-A enzyme inhibitor: single and multiple dosing in normal subjects. *Clin Pharmacol Ther* **42** 395-404. (1987)

25. Nielsen K.K., Flinois J.P., Beaune P., Brosen K. The biotransformation of clomipramine in vitro, identification of the cytochrome P450s responsible for the separate metabolic pathways. *J Pharmacol Exp Ther* **277** 1659-1664. (1996)

26. Yu K.S.*, et al.* Effect of omeprazole on the pharmacokinetics of moclobemide according to the genetic polymorphism of CYP2C19. *Clin Pharmacol Ther* **69** 266-273. (2001)

27. Accorda Therapeutics Inc. Zanaflex prescribing information. 2013 [cited 2019 25 November 2019]Available from: <https://www.accessdata.fda.gov/drugsatfda_docs/label/2013/021447s011_020397s026lbl.pdf>

28. Granfors M.T., Backman J.T., Laitila J., Neuvonen P.J. Tizanidine is mainly metabolized by cytochrome p450 1A2 in vitro. *Br J Clin Pharmacol* **57** 349-353. (2004)

29. Granfors M.T., Backman J.T., Neuvonen M., Ahonen J., Neuvonen P.J. Fluvoxamine drastically increases concentrations and effects of tizanidine: a potentially hazardous interaction. *Clin Pharmacol Ther* **75** 331-341. (2004)

30. Labbe L.*, et al.* Pharmacokinetic and pharmacodynamic interaction between mexiletine and propafenone in human beings. *Clin Pharmacol Ther* **68** 44-57. (2000)

31. Wei X.*, et al.* Inhibition of human liver cytochrome P-450 1A2 by the class IB antiarrhythmics mexiletine, lidocaine, and tocainide. *J Pharmacol Exp Ther* **289** 853-858. (1999)

32. Joeres R., Klinker H., Heusler H., Epping J., Richter E. Influence of mexiletine on caffeine elimination. *Pharmacol Ther* **33** 163-169. (1987)

33. Momo K.*, et al.* Effects of mexiletine, a CYP1A2 inhibitor, on tizanidine pharmacokinetics and pharmacodynamics. *J Clin Pharmacol* **50** 331-337. (2010)

34. Kusumoto M.*, et al.* Effect of fluvoxamine on the pharmacokinetics of mexiletine in healthy Japanese men. *Clin Pharmacol Ther* **69** 104-107. (2001)

35. Kusumoto M.*, et al.* Lack of pharmacokinetic interaction between mexiletine and omeprazole. *Ann Pharmacother* **32** 182-184. (1998)

36. Ezuruike U.*, et al.* Risk-Benefit Assessment of Ethinylestradiol Using a Physiologically Based Pharmacokinetic Modeling Approach. *Clin Pharmacol Ther* **104** 1229-1239. (2018)

37. Balogh A.*, et al.* Influence of ethinylestradiol-containing combination oral contraceptives with gestodene or levonorgestrel on caffeine elimination. *Eur J Clin Pharmacol* **48** 161-166. (1995)

38. Granfors M.T., Backman J.T., Laitila J., Neuvonen P.J. Oral contraceptives containing ethinyl estradiol and gestodene markedly increase plasma concentrations and effects of tizanidine by inhibiting cytochrome P450 1A2. *Clin Pharmacol Ther* **78** 400-411. (2005)

39. Karjalainen M. Inhibition of CYP1A2-mediated drug metabolism in vitro and in humans: With special emphasis on rofecoxib and other NSAIDs. University of Helsinki, Finland, 2008.

40. Chang S.Y., Chen C., Yang Z., Rodrigues A.D. Further assessment of 17alpha-ethinyl estradiol as an inhibitor of different human cytochrome P450 forms in vitro. *Drug Metab Dispos* **37** 1667-1675. (2009)
